# Atlantic forest origins and Amazon connections: the evolutionary history of *Adenocalymma*

**DOI:** 10.1093/aob/mcag087

**Published:** 2026-04-13

**Authors:** Luiz Henrique M Fonseca, Lars W Chatrou, Lúcia G Lohmann

**Affiliations:** Departamento de Botânica, Instituto de Biociências, Universidade de São Paulo, São Paulo 05508-090, Brazil; Systematic and Evolutionary Botany Laboratory, Department of Biology, Universiteit Gent, Ghent 9000, Belgium; Institut für Ökologie, Evolution und Diversität, Fachbereich Biowissenschaften, Goethe-Universität Frankfurt, Frankfurt am Main 60438, Germany; Systematic and Evolutionary Botany Laboratory, Department of Biology, Universiteit Gent, Ghent 9000, Belgium; Naturalis Biodiversity Center, Leiden 2300, the Netherlands; Department of Biology, Washington University in St. Louis, St. Louis, MO 63130, USA; Missouri Botanical Garden, St. Louis, MO 63110, USA

**Keywords:** *Adenocalymma*, Bignoniaceae, tropical plants, hybrid-capture, incomplete lineage sorting, introgression, phylogenomics, biogeography

## Abstract

**Background and Aims:**

The Amazon and the Atlantic Forest are the most diverse tropical rainforests globally. They are separated by a ‘dry diagonal’ of mesic forests and savannas. The formation times of these ecoregions and the historical connections between them remain debated, highlighting gaps in our understanding of the continent’s biogeographical history. Here, we used the endemic clade *Adenocalymma* to further understand the formation and connections of current ecoregions in the Neotropics.

**Methods:**

We sampled 68 species of *Adenocalymma*, representing 87 % of species diversity, and used nuclear genomic data to infer their phylogeny. To better characterize the evolutionary history of *Adenocalymma*, we applied supermatrix and species-tree methods, as well as approaches that account for introgression and incomplete lineage sorting (ILS). Divergence times were inferred using a subset of genes. Finally, we applied the resulting phylogenetic hypothesis to infer ancestral ranges and the diversification patterns within the genus.

**Key Results:**

*Adenocalymma* was strongly supported as monophyletic in all analyses. Support for internal clades was generally high. ILS and introgression accounted for a minor proportion of gene-tree incongruences, suggesting that methodological factors may be relevant. The Atlantic Forest was identified as the ancestral area and main source of lineages for adjacent ecoregions. Vicariance was clearly observed in two instances. *Adenocalymma* showed a sharp increase in speciation and net diversification rates during the last 5 Ma.

**Conclusions:**

Our results demonstrate that nuclear genomic data are highly valuable for inferring robust phylogenetic trees and networks in Bignoniaceae. ILS and introgression appear to be less relevant for shaping phylogenetic incongruence. Furthermore, the Amazon Forest may have contributed fewer plant lineages to other ecoregions than previously suggested. Diversification within *Adenocalymma* showed a pronounced accumulation of lineages during the last 5 Ma, especially in the Atlantic Forest.

**Summary:**

This study explored the phylogeny of the Neotropical genus *Adenocalymma* using genomic data and used this evolutionary framework to better understand the biogeographical history of the focal clade and the Neotropics. Here, we showed that the lineage originated in the Atlantic Forest and further colonized other ecoregions. We also recovered a rapid increase in diversification of *Adenocalymma* over the last 5 Ma.

## INTRODUCTION

The Amazon and the Atlantic Forests harbour among the highest numbers of land plants worldwide (Mittermeier *et al*., 2005; Hoorn *et al*., 2010). The Atlantic Forest also stands out for having up to 50 % plant endemism (BFG, 2022), driven largely by *in situ* diversification and isolation (Peres *et al*., 2020). Currently, the Amazon and Atlantic Forests are separated by a large, dry diagonal composed of more open vegetation with pronounced seasonality (Werneck, 2011). However, this landscape configuration has changed drastically over evolutionary time. A continuous forest putatively covered South America during most of the Palaeogene (Morley, 2000), with megafossil evidence of wet forests dating back to the late Palaeocene (Wing *et al*., 2009). Even during the Eocene thermal maximum, a continuous tropical rainforest probably remained stable, and there is no fossil evidence of increased aridity or formation of savannas during this period (Jaramillo *et al*., 2006; Jaramillo and Cárdenas, 2013).

The Dry Diagonal is composed by Seasonally Dry Tropical Forests (SDTFs), especially the Caatinga in northeast Brazil, along with savannas (Cerrado) and Chaco. Together, these biomes form an efficient barrier for biotic exchanges between the Amazon and the Atlantic Forests (Oliveira-Filho and Ratter, 1995). The Caatinga, Cerrado and Chaco experience seasonal drought, and occur across a great variety of climatic, latitudinal and altitudinal ranges. The age of the Dry Diagonal and the formation of two separated and extensive wet forests within South America are matters of discussion but have been hypothesized to have started during the Oligocene (Perret *et al*., 2013; Thode *et al*., 2019; Hoorn *et al*., 2023), which was marked by global climatic cooling and aridification (Westerhold *et al*., 2020). The ages of the ecoregions that currently occupy the Dry Diagonal are hypothesized to be younger, though they date back to the Miocene for the Caatinga and Cerrado (Simon *et al*., 2009; Fernandes *et al*., 2022). The current Chaco flora dates back to the Pliocene or early Pleistocene and was established on saline soils resulting from marine incursions that began during the Oligocene and intensified with Andean uplift (Hernández *et al*., 2005; Hoorn *et al*., 2010; Ruskin *et al*., 2011).

The exact age of formation of the Cerrado is still uncertain. Both the evolution of ungulate grazers and pollen records indicate the emergence of savannas or seasonally dry woodlands in the late Oligocene or middle Miocene (≈30 Ma) (Jacobs *et al*., 1999). This age coincides with the central Andean uplift (Hoorn *et al*., 2010, 2023) and is supported by a few phylogenetic data (e.g. Thode *et al*., 2019) and fossil evidence (Romero, 1993). The physiognomy of these formations and their extensions during the Oligocene and early Miocene are still poorly known. Robust evidence from the fossil record suggests an expansion of savannas and dry forests slightly later, beginning in the middle Miocene (≈15 Ma) (Edwards *et al*., 2010). Major diversification of some iconic Cerrado plants such as Fabaceae or Poaceae dates back to the middle or late Miocene (≈12–8 Ma) (Simon *et al*., 2009; Edwards *et al*., 2010; Huang *et al*., 2022). This period coincides with the emergence of C4 grass-dominated savannas (Jacobs *et al*., 1999; Edwards *et al*., 2010; Huang *et al*., 2022). The current Cerrado, with its fire regime, is highly dependent on C4 grasses, but its past dynamics remain poorly known. It is plausible that savanna-like physiognomies existed before the late Miocene, with mixed C3 and C4 grasses (Jacobs *et al*., 1999).

The existence of seasonally dry forests, as we currently see in northeast Brazil, since the late Miocene or earlier is elusive; the formation of the Caatinga’s geomorphological systems is the result of large-scale processes that began during this period (Fernandes *et al*., 2022). Geological and climatic drivers are responsible for the region’s aridification, and most endemic plant clades originated *in situ*. The plant lineages possibly emerged during the Pliocene/Pleistocene and evolved in response to diverse edaphic conditions (Fernandes *et al*., 2022). However, the stability of a continuous seasonally dry forest core in northeast Brazil through time is controversial, since the pollen record (Oliveira *et al*., 1999) and climatic data (Wang *et al*., 2004; Cheng *et al*., 2013) suggest the replacement of at least part of the Caatinga by wet forests. This is further supported by climate models, which indicate that highly unstable seasonally dry forests in northeast Brazil were present during the last glacial maximum (LGM) (Costa *et al*., 2018).

Even though South America is currently fragmented into distinct ecoregions, evidence from phylogenetics, climatology and geology suggests recurrent spatial reconfigurations and numerous instances of rainforest reconnections. Species range expansions during ancient connections followed by interruptions in gene flow during periods of isolation seem to have represented powerful engines for the diversification of taxa in these ecoregions (Morley, 2000; Batalha-Filho *et al*., 2013; Sobral-Souza *et al*., 2015; Ledo and Colli, 2017; Costa *et al*., 2018). As such, a signature must have been left in the history of organisms that are disjunctly distributed, such as cladogenesis among different organisms concentrated in some time windows (Ledo and Colli, 2017). In South America, the colonization of new ecoregions followed by *in situ* diversification has also been documented, with the Amazon Forest serving as a source of species and lineages for adjacent ecoregions and the Atlantic Forest (Antonelli *et al*., 2018; Nicholls *et al*., 2025).

Here, we inferred the phylogeny of the Neotropical genus *Adenocalymma* Mart. ex Meisn. emend L.G.Lohmann using genomic data obtained using the recently developed bait kit for the plant family Bignoniaceae that targets 762 nuclear genes (Fonseca *et al*., 2023). We also applied the new phylogenetic framework to illuminate the biogeographical and diversification history of this plant group. The genus has two centres of diversity, one in the Amazon Forest and the other in the Atlantic Forest. The remaining species are endemic to the Caatinga, Cerrado, the Pacific Domain (Morrone *et al*., 2022) and Central America. Few species are widespread and occur in up to two different biomes, such as *A. flaviflorum* (Miq.). L.G.Lohmann and *A. validum* (K.Schum.) L.G.Lohmann that occur disjunctly in the Amazon and Atlantic Forests (Fonseca and Lohmann, 2019). Most *Adenocalymma* species share their distribution with congenerics, and clades recovered in previous phylogenetic studies using Sanger-generated and genome skimming molecular data (Fonseca and Lohmann, 2018, 2020) showed strong geographical structure. Most representatives of the former genus *Memora* Miers (now a synonym of *Adenocalymma*) are Amazonian taxa that belong to a well-supported clade nested within *Adenocalymma*. The clade composed by the species of the former *Adenocalymma s.s.* (Fonseca and Lohmann, 2018, 2020) also formed a geographically structured clade, with most species occurring in the Atlantic Forest (Fonseca and Lohmann, 2018, 2020).

The sympatric distribution of many closely related species could favour gene flow and challenge the morphological integrity of individual species. For flowering plants in general, more than 20 % of plant taxa are expected to have a hybrid origin, while hybridization events have been documented in the phylogenetic history of 70 % of all plants (Rieseberg, 1997; Soltis and Soltis, 2009; Payseur and Rieseberg, 2016). Introgression has been explored at relatively recent timescales in various taxa such as roses (*Rosa* L.; up to 20 Ma; Debray *et al*., 2022) and wheat relatives (*Aegilops* L./*Triticum* L.; 9 Ma; Glémin *et al*., 2019). Extreme examples of introgression in recent geological timescales have also been observed in oaks (*Quercus* L.; Cannon and Petit, 2020) and the African Detarioid legumes (Boom *et al*., 2021). The distribution patterns of *Adenocalymma* suggest a hidden, convoluted evolutionary history for the clade, although hybrids have not been formally described or observed in nature or in herbaria. Importantly, independent nuclear regions have not been available for exploring the role of hybridization in the clade. Few examples of introgression have been documented for tropical plant clades (Boom *et al*., 2021; Morales-Briones *et al*., 2021; Gardner *et al*., 2023), and it remains unclear whether introgression has also played an important role in the diversification history of these plants.

In this study, we test the robustness of previous phylogenetic trees, based primarily on chloroplast data (Fonseca and Lohmann, 2018, 2020), using a much more comprehensive dataset that includes hundreds of independent nuclear genes. Given the gene conflict recovered in some nodes of the Bignoniaceae phylogeny (Fonseca *et al*., 2023), we also examined gene tree discordance patterns and inferred the putative processes behind the discordance. To better characterize the context of *Adenocalymma* evolution, we estimated the age of the genus, ancestral areas of distribution for internal lineages and the diversification patterns of the clade. Using ancestral area reconstruction and diversification analysis we explicitly tested the role of wet forest fragmentation and the Dry Diagonal formation on the diversification of the focal genus.

## MATERIALS AND METHODS

### Taxon sampling

We sampled 68 different species of *Adenocalymma* following the concepts presented in Fonseca and Lohmann (2019). This sampling scheme allowed us to include ca. 87 % of the 78 currently recognized species. Specimens from the entire geographical range of *Adenocalymma* were sampled, with a particular focus on species from the Amazon and the Brazilian Atlantic Forest (Fonseca and Lohmann, 2019). We also sampled two individuals of *A. flaviflorum*, *A. mirabile* (Sandwith) L.H.Fonseca & L.G.Lohmann, *A. saulense* A.H.Gentry and *A. schomburgkii* (DC.) L.G.Lohmann. Seventy-one specimens of *Adenocalymma* were sequenced for this study. *Adenocalymma acutissimum* (Cham.) Miers was made available from Fonseca *et al*. (2023). As outgroups, we included *Anemopaegma arvense* (Vell.) Stellfeld ex de Souza, *Callichlamys latifolia* (Rich.) K.Schum., *Crescentia cujete* L., *Dolichandra cynanchoides* Cham., *Fridericia speciosa* Mart., *Jacaranda mimosifolia* D.Don., *Perianthomega vellozoi* Bureau and *Tanaecium jaroba* Sw. from Fonseca *et al*. (2023). Materials for this study were collected in the field and dried in silica gel (61 specimens) or obtained from herbarium specimens (19 specimens) from the MO and SPF herbaria (Supplementary Data Table S1).

### DNA isolation, library preparation, sequencing and gene assembly

Total genomic DNA was extracted from silica-dried or herbarium samples using the Invisorb® Spin Plant Mini Kit (Invitek, Berlin, Germany). Total DNA was quantified using a Qubit BR assay. DNA quality was also evaluated using a NanoDrop 2000 (Thermo Fisher Scientific) and gel electrophoresis. Library assembly and target capture reactions were performed by the QB3 Genomics facility (University of California, Berkeley, CA, USA) using their high-throughput workflow. All samples were prepared into standard-sized libraries using Kapa Biosystems library preparation kits (using a Covaris sonicator to fragment gDNA) and custom Unique Dual Indexes. Samples were multiplexed, and the target genes were captured using the custom Bignoniaceae bait kit (Fonseca *et al*., 2023). The fragments were sequenced on an Illumina NovaSeq 6000 S4 with 150-bp paired-end reads.

Illumina reads were demultiplexed at the sequencing facility, and low-quality reads were trimmed using Trimmomatic 0.35 (Bolger *et al*., 2014) with the SLIDINGWINDOW:10:20 and MINLEN:40 parameters (e.g. remove read segments where average quality drops below Q20 across a 10-bp window; discard reads shorter than 40 bp after trimming). HybPiper 2.3.2 (Johnson *et al*., 2016) was used to obtain the gene sequences using the ‘assemble’ command. As targets, we used the original reference file with 762 genes used to design the baits (Fonseca *et al*., 2023). The pipeline uses BWA 0.7.17 (Li and Durbin, 2009) to filter reads using the targets; SPAdes 3.6.1 (Bankevich *et al*., 2012) to assemble contigs *de novo*; and Exonerate 2.4.0 (Slater and Birney, 2005) to return Supercontigs (exons + introns/intergenic-regions) and Exon-Only sequences per gene. We retrieved FASTA files of Exon-Only and Supercontig sequences containing all sampled species using the ‘retrieve_sequences’ command. Summary statistics were calculated using the ‘stats’ command, and the heatmap plot was obtained using the ‘gene_recovery_heatmap.R’ script.

Paralogue warnings produced by the HybPiper pipeline for samples with multiple long contigs were further investigated using the HybPiper scripts ‘paralog_retriever’ command. In total, 95 genes were flagged as having more than three samples containing paralogues and were discarded from both the Exon-Only and Supercontig datasets. We also applied a threshold criterion of 75 %/75 %, first removing sequences that did not have 75 % of the maximum size of the gene reference and then removing the genes that did not have 75 % of the samples (custom R code available at https://github.com/luizhhziul/Adenocalymma). The final gene set contained 661 putative single-copy loci with a threshold criterion of 75 %/75 % out of the 762 initial targets.

### Gene alignment, quality control and super-matrix analyses

Phylogenomic inferences were performed using a subset of 661 genes. Two datasets were used for analysis in this and the next section: (1) Exon-Only, with just exons; and (2) Supercontig, with exons, complete or partial introns, and complete or partial intergenic regions. Sequences were aligned using MAFFT 7.490 (Katoh and Standley, 2013) with an automatic selection of alignment strategy and a maximum of 1000 iterations. Alignments were concatenated, and a supermatrix was built using AMAS (Borowiec, 2016). To remove poorly aligned regions, we used ClipKIT 1.3.0 (Steenwyk *et al*., 2020) with the parameters -m gappy -g 0.5, retaining other settings at their defaults. The alignments were also cleaned using Spruceup 2024.7.20 (Borowiec, 2019) using default parameters and a cutoff of 3. The number of parsimony-informative sites (PIS) for each gene alignment and combined alignments was obtained using AMAS.

All downstream analyses were based on edited DNA matrices (i.e. after editions with ClipKIT and Spruceup). Genes were analysed separately or combined in super-matrices. Individual gene alignments were used for phylogenetic reconstructions under the maximum-likelihood (ML) criterion in RAxML-NG 1.2.2 (Kozlov *et al*., 2019) with 100 non-parametric bootstraps and the parameter –model GTR+G. For combined matrices, we used the ML criterion in IQ-TREE 2.1.3 (Nguyen *et al*., 2015) with the option -m TESTMERGE to select the best partition scheme and evolutionary models and 1000 UFBoot samples (Hoang *et al*., 2018).

### Species tree inference

To address the statistical inconsistency of supermatrix approaches (Kubatko and Degnan, 2007) and account for incomplete lineage sorting (ILS), we first used Weighted ASTRAL 1.19.3.5 (Mirarab *et al*., 2014; Zhang and Mirarab, 2022) as implemented in ASTER (https://github.com/chaoszhang/ASTER). Given the computational difficulties of jointly estimating gene trees and the species tree, we used wASTRAL as a two-step approach to infer the species tree (Mirarab *et al*., 2014). Low-support branches (i.e. <20 %) were collapsed to improve accuracy, as discussed by Mirarab (2023). Branch support in wASTRAL was calculated using local posterior probabilities (LPP).

We also used the site-based species tree inference implemented in CASTER v.1.19.1.4 (Zhang *et al*., 2025). This method uses site pattern frequencies as input to estimate the species tree under the MSC (Multi-Species Coalescent) model directly. Because site-based methods do not require gene tree estimation, these methods are more robust to some conditions, such as low phylogenetic signal in gene trees resulting in a poor species tree (Zhang *et al*., 2025). Branch support was estimated using local bootstrap support. To evaluate gene tree and site congruence, we used quartet frequencies inferred using wASTRAL, and the gene concordance factor (gCF) and site concordance factor (sCF) implemented in IQ-TREE 2.1.3 (Nguyen *et al*., 2015; Minh *et al*., 2020).

### Reticulated evolution

An explicit tree-based approach was used to infer introgression using maximum pseudolikelihood (MPL). The MPL approach estimates the network, branch lengths and heritability that jointly maximize the pseudolikelihood (Yu and Nakhleh, 2015). MPL optimization was fully implemented in PhyloNet. PhyloNet is widely used for inferring networks, using the pseudolikelihood framework to estimate the extent and direction of introgression using the composed trees in triplets (Yu and Nakhleh, 2015). To reduce the number of species to tractable numbers, we divided the phylogeny of *Adenocalymma* in three subtrees: (1) ‘Early Diverging Lineages’, (2) ‘Amazon Forest Lineages’ and (3) ‘Amazon forest Lineages’ with respectively 15, 15 and 18 terminals sampled. As input, we used the gene trees obtained from the supercontigs to estimate between one and five reticulations. The phylogenetic tree (i.e. without reticulations) was also estimated under PhyloNet for comparison. For each *k* value, trees/reticulations were inferred using 50 runs to maximize the chance to recover the MPL tree/network. Topologies and pseudolikelihood values were used for discussion of the results. Networks were plotted using the package PhyloPlots (https://github.com/JuliaPhylo/PhyloPlots.jl).

We also used the NeighborNet algorithm to infer a phylogenetic network of the genus. The algorithm is loosely based on neighbour-joining, using a distance matrix as input to agglomerate clusters (Bryant and Moulton, 2004). This method is an implicit approach to estimating a network and is implemented in the R package phangorn (Schliep, 2011). To visualize the result, we used the R package tanggle (Schliep *et al*., 2017).

### Divergence time estimation

To reconstruct the historical biogeography and diversification of *Adenocalymma*, we first inferred a time-calibrated tree using one specimen per species, comprising 68 ingroup species and all eight outgroups. For tree calibration, only gene alignments from the Supercontig dataset were used. Due to computational limitations to handle data from hundreds of genes to time-calibrate trees, we used a subset of genes selected using a custom R script (available at https://github.com/luizhhziul/Adenocalymma) and the packages phangorn, adephylo (Jombart *et al*., 2010) and ips (Heibl not published, available at https://github.com/heibl/ips). The selection was based on gene length, molecular variability, clock-like pattern (variability among branch lengths) and topology distance from the Supercontig wASTRAL tree. A weighting scheme of 1:1:2:2 was used, respectively. As constraints for node dating, we used the ages of Bignoniaceae (66.13–58.46 Mya), tribe Bignonieae (54.2–45.7 Mya) and the core Bignonieae clade (37–33.7 Mya; Lohmann *et al*., 2013). No constraints were used for *Adenocalymma*, since the clade composed of *A. pedunculatum* (Vell.) L.G.Lohmann and *A. validum*, sister to the rest of the *Adenocalymma* species (Fonseca and Lohmann, 2018, 2020), was not sampled by Lohmann *et al*. (2013). The tree was calibrated using MCMCtree (Yang, 2007), a software package widely used for Bayesian molecular clock dating of species divergences in the genomics era due to its computational efficiency (Dos Reis *et al*., 2016). The settings of MCMCtree followed recommendations from Dos Reis and Yang (2019), and the topology was fixed following the ASTRAL result. Because fossils are unavailable for the genus, outgroups were used to anchor ages. To visualize posterior ages on trees, we used the R package MCMCtreeR (Puttick, 2019).

### Diversification analyses

We estimated changes in speciation and extinction rates over time and across lineages using Bayesian methods implemented in RevBayes. We first used an episodic birth–death (EBD) model, in which rates are modelled as a piecewise-constant Brownian process changing at discrete points in time, with the mean-variance autocorrelated between time slices (Höhna, 2014; Magee *et al*., 2020). We used as the reference tree the result from MCMCtree, pruned to exclude the outgroup and leaving a single species per genus. We followed the tutorial https://revbayes.github.io/tutorials/divrate/ebd.html, using 100 intervals to discretize rate variation through time, and a chain length of 50 000 generations. To explore whether changes in diversification rates are linked to specific lineages, we implemented a lineage-specific birth–death shift model (LSBS), which assumes a multistate state-dependent speciation and extinction (SSE) model (Maddison *et al.*, 2007), with each state having its own rates, and where shifts in states (e.g. speciation rates) are modelled dynamically over the tree (Höhna *et al*., 2019). With this approach, we are able to estimate the exact position on the phylogeny of putative rate shifts. We followed the tutorial https://revbayes.github.io/tutorials/divrate/branch_specific.html, using six rate categories to discretize the lognormal distribution of the speciation rate variation, and a chain length of 3000 generations. We assumed that the extinction rate was the same for all rate categories. To visualize the results, we used the R package RevGadgets. The custom scripts to run and visualize the results are available at https://github.com/luizhhziul/Adenocalymma.

### Historical biogeography

Distribution data for *Adenocalymma* were compiled from herbaria collections and curated following the procedures described in Narváez-Gómez *et al*. (2021). In total, 4337 records were compiled for the whole genus and used in various analyses; the distribution data ranged from a single population of the recently described *A. apetiolatum* L.H.Fonseca & L.G.Lohmann, *A. cauliflorum* L.H.Fonseca & L.G.Lohmann, *A. darwinii* L.H.Fonseca and *A. fistulosum* L.H.Fonseca & L.G.Lohmann, to 395 entries for the widespread *A. marginatum* (Cham.) DC. Five biogeographical areas were coded using the distribution dataset available for *Adenocalymma*: [A] Atlantic Forest, [B] Caatinga, [C] Cerrado, [D] Amazon Forest, and [E] Pacific Domain and Central America. Area delimitation was based on the proposal of Morrone *et al*. (2022), with adjustments to better reflect the distribution of *Adenocalymma*.

We inferred the evolution of the geographical range under the dispersal–extinction–cladogenesis (DEC) model (Ree and Smith, 2008). DEC models specific ‘range-inheritance’ events at speciation (cladogenesis), such as sympatry, subset speciation (vicariance) or widespread ancestor scenarios, allowing the text of different biogeographical scenarios. We used the Bayesian implementation of the DEC model (Landis *et al*., 2018) in RevBayes 1.4.0 (Höhna *et al*., 2016), which allows us to account for uncertainty in the values of anagenetic parameters and ancestral nodal ranges by estimating marginal posterior probabilities (Landis *et al*., 2018). As input, we used the dated tree from MCMCtree with a species-level sampling. We allowed up to two areas per node, representing the maximum number of areas observed for extant taxa. A total of 10 000 generations were used and a burn-in of 25 % was applied. To run the analysis, we built a RevBayes script using the tutorial https://revbayes.github.io/tutorials/biogeo/biogeo_epoch.html as reference. The custom scripts to run and visualize the results are available in https://github.com/luizhhziul/Adenocalymma. Stochastic Character Mapping (Freyman and Höhna, 2019) was used to infer the timing and direction of geographical range changes along branches, by dividing the phylogeny into 1000 time slices. Results were summarized into a maximum a posteriori (MAP) tree.

To evaluate the role of the Dry Diagonal for the biogeographical history of the plants, we used two approaches: a single dispersal matrix considering the current distribution of the biomes for the whole diversification time of *Adenocalymma*; and time-stratified dispersal matrices – one accounting for the initial formation of the Dry Diagonal and the separation of the putative continuous rain forest over South America until 15 Ma, one dispersal matrix accounting for the establishment of the Cerrado and initial formation of the Caatinga, from 15 to 5 Ma, and a dispersal matrix accounting for the full establishment of the Caatinga from 5 Ma to the present (code scheme available at https://github.com/luizhhziul/Adenocalymma). The time-stratified approach allowed us to reduce the probability of connections between the rainforests while increasing the probability of occupancy of one of the Dry Diagonal biomes over time. The two approaches were compared using marginal likelihoods estimated via stepping-stone sampling. To visualize the results, we used the R package RevGadgets v.1.0 (Tribble *et al*., 2022).

## RESULTS

### Sequencing, gene assembly, and alignment statistics

We recovered an average of 20 124 934 paired-end reads per specimen after trimming, with a maximum of 52 197 438 and a minimum of 6 060 152. Baits showed high accuracy, with most raw reads mapping to a target gene (Supplementary Data Table S2). We assembled 615–737 genes, with an average of 725.7 genes per specimen at 75 % or more (Fig. S1; Table S2). Nine genes failed across all species, consistent with earlier findings for Bignoniaceae as a whole (Fonseca *et al*., 2023). HybPiper flagged 213 genes that might contain paralogues, averaging 42.8 genes per specimen. After removing the 95 genes flagged as having paralogues for more than two individuals, a final array of 661 genes was used for gene alignments and phylogenetic tree searches.

Here, we report on datasets after editions using ClipKIT and Spruceup. The mean length of the Exon-Only alignments for all 661 genes was 1268.4 bp, with sizes ranging between 189 and 6516 bp. The mean PIS value was 98, ranging from 13 to 470 bp across the complete dataset. When outgroups were excluded, the mean PIS was 54.2 bp, ranging from 6 to 271 bp. The combined Exon-Only alignment was 838 418 bp long, with 64 806 bp PIS when outgroups were included or 35 801 bp PIS when outgroups were removed (Table 1). The mean length of the Supercontig alignments for all 661 genes was 3673.5 bp, with sizes ranging between 809 and 11 918 bp. The mean value of PIS was 674.1, ranging between 88 and 2303 bp. When outgroups were excluded, the mean PIS was 414.5 bp, ranging from 59 to 1393 bp. The combined Exon-Only alignment was 2 428 218 bp long, of which 445 584 bp were PIS, including outgroups, and 274 018 bp when outgroups were removed (Table 1).

**Table 1.** Number of aligned and parsimony-informative bases for each dataset with and without outgroups.

	Data	Size	PIS	PIS (non-out)
Exon-Only	Mean	1268.4	98	54.2
	Min.	189	13	6
	Max.	6516	470	271
	Combined	838 418	64 806	35 801
Supercontig	Mean	3673.5	674.1	414.5
	Min.	809	88	59
	Max.	11 918	2303	1393
	Combined	2 428 218	445 584	274 018

### Combined trees

**Fig. 1.**
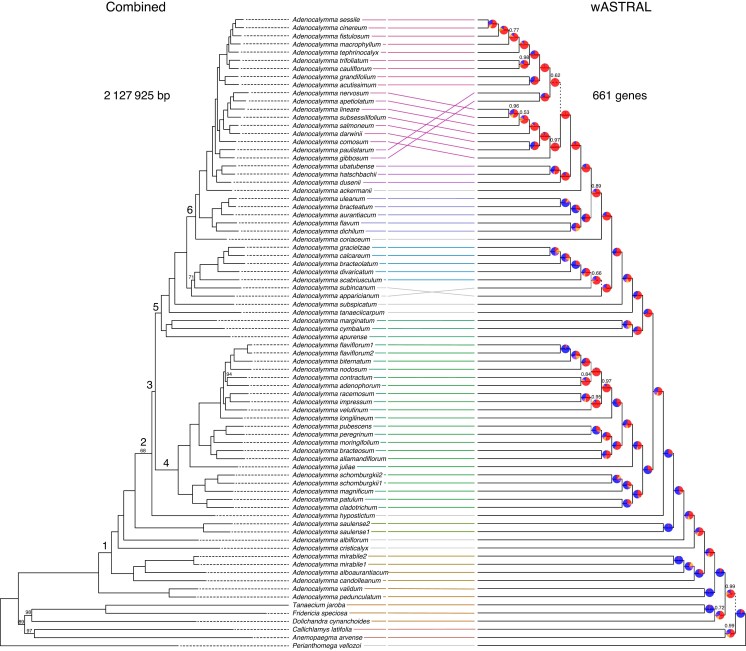
Tanglegram comparisons of phylogenies obtained using a supermatrix approach and IQ-TREE, and a coalescent approach and wASTRAL of the Supercontig dataset. The supermatrix result includes ultra-fast bootstrap proportion (UFBoot) support values. The coalescent result is labelled with local posterior probabilities (LPP). Maximum support of the UFBoot and LPP are not shown. Gene concordance factor (gCF) values are shown as pie charts. For each pie chart, blue represents the proportion of gene trees concordant with that branch, purple represents the proportion of gene trees concordant with the first alternative quartet, orange represents the proportion of gene trees concordant with the second alternative quartet and red represents the gene discordance support due to polyphyly. Branches labelled on the supermatrix tree are discussed in the text.

The single ML tree derived from the concatenated Exon-Only dataset is well supported, with most branches receiving high UFBoot values (>90 %; Supplementary Data Fig. S2), and a mean UFBoot of 94.7 % across the tree. The tree was rooted with *Jacaranda mimosifolia*, following previous phylogenetic reconstructions of Bignoniaceae (Fonseca *et al*., 2023). The genus *Adenocalymma* emerged as monophyletic with maximum UFBoot support. Only 18 nodes within the genus had support values below 100 % (Fig. S2). The ML tree derived from the concatenated Supercontig dataset has the most branches supported by high UFBoot values (>90 %) (Fig. 1), with a mean UFBoot of 99.9 % across the tree. The genus *Adenocalymma* emerged as monophyletic with maximum UFBoot support. Only three branches within the genus received UFBoot values below 100 % (Fig. 1).

Both Exon-Only and Supercontig analyses recovered highly similar topologies, differing only in the position of seven terminals (Fig. 1; Supplementary Data Fig. S2). (1) In the Supercontig tree, *A. hypostictum* Bureau & K.Schum. was sister to Clade 3, whereas in the Exon-Only topology it was sister to Clade 3; (ii) *A. velutinum* (A.H.Gentry ex Hauk) L.G.Lohmann was sister to the clade composed of *A. impressum* (Rusby) Sandwith and *A. racemosum* (A.H.Gentry) L.G.Lohmann in the Supercontig tree, but was sister to *A. contractum* (A.H.Gentry ex Hauk) L.G.Lohmann in the Exon-Only tree; (iii) *A. contractum* was recovered as sister to *A. adenophorum* (Sandwith) L.G.Lohmann in the Supercontig analysis; (iv) *A. coriaceum* DC. was sister to the remainder of Clade 6 in the Supercontig tree but was nested within that clade in the Exon-Only topology; (v) *A. lineare* L.H.Fonseca & L.G.Lohmann was recovered as sister to the *A. nervosum* Bureau & K.Schum. + *A. apetiolatum* L.H.Fonseca & L.G.Lohmann clade in the Supercontig tree, whereas in the Exon-Only tree it was sister to a broader clade composed of *A. subsessilifolium* DC., *A. nervosum* and *A. apetiolatum*; (vi) *A. cauliflorum* L.H.Fonseca & L.G.Lohmann was sister to *A. trifoliatum* (Vell.) R.C.Laroche in the Supercontig tree but sister to a larger clade of five species in the Exon-Only tree; and (vii) finally, *A. tephrinocalyx* Bureau & K.Schum. was sister to *A. macrophyllum* (Cham.) DC. in the Supercontig tree but sister to a clade comprising *A. macrophyllum*, *A. fistulosum* L.H.Fonseca & L.G.Lohmann, *A. sessile* Udulutsch & Assis, and *A. cinereum* Udulutsch & Assis in the Exon-Only topology (Fig. 1; Fig. S2).

### Gene tree and species trees

The Exon-Only tree was recovered using wASTRAL, with a mean LPP support of 0.96, ranging from 0.455 to 1. In total, 23 branches recovered LPP values below 1. The mean quartet support value was 0.587, ranging from 0.344 to 0.996. Mean gCF was 22.455, ranging from 0.303 to 94.2 (Supplementary Data Fig. S3). Mean sCF was 50.67, ranging from 30.3 to 97.7 (Fig. S3). Comparing this tree (Fig. S3) with the tree derived from the combined analysis of the Exon-Only dataset (Fig. S2) resulted in a Robinson–Foulds (RF) distance of 20. Topological differences were observed in seven positions and involved nine different species, including *A. hypostictum*, *A. contractum* and *A. velutinum* (Fig. S3). The Supercontig dataset recovered a species tree using wASTRAL, with a mean LPP support of 0.972, ranging from 0.529 to 1. In total, 18 nodes showed LPP values below 1. The mean quartet support value was 0.622, ranging from 0.356 to 0.994. Mean gCF was 37.197, ranging from 0.605 to 98.6 (Fig. 1). Mean sCF was 51.944, ranging from 33 to 97.5 (Fig. S5). Comparing the Supercontig wASTRAL with the combined Supercontig tree (Fig. 1), the RF distance was 6. Only two internal branches were not shared between the Supercontig wASTRAL tree and the Supercontig combined tree, with differences in the positions of *A. apparicianum* J.C.Gomes, *A. subincanum* Huber and the clade (*A. gibbosum* Udulutsch & Assis, *A. paulistarum* Bureau & K.Schum.) (Fig. 1).

The Exon-Only dataset recovered a species tree using CASTER-site with a mean local bootstrap support value of 86.953, ranging from 50.4 to 94.8. When this tree (Supplementary Data Fig. S4) was compared with the tree derived from the combined analysis of the Exon-Only dataset (Fig. S2), the RF distance was 28. Multiple topological differences were recovered, including differences in the position of *A. hypostictum* and many taxa inside Clade 6 (Figs S2 and S4). The results of the species tree inferences using the Exon-Only dataset through wASTRAL and CASTER-site share some clades, and the RF distance is 22, with nine branches not shared within the genus (Figs S3 and S4). The Supercontig dataset recovered a species tree using CASTER-site, with a mean local bootstrap support of 96.07, ranging from 55.5 to 100. In total, 53 branches recovered BS values below 100 (Fig. S5). When the Supercontig CASTER-site tree (Fig. S5) was compared with the combined Supercontig tree (Fig. 1), the RF distance was 6. Topological differences included the positions of *A. hypostictum*, the clade (*A. gibbosum*, *A. paulistarum*) and the clade (*A. macrophyllum*, *A. tephrinocalyx*) (Fig. 1; Fig. S5). The results of the species tree inferences using the Supercontig dataset through wASTRAL and CASTER-site are very similar and only two branches were not shared between the two trees (Fig. 1; Fig. S5).

Given the considerable difference in molecular variability between the Exon-Only and Supercontig datasets (Table 1) and the topological congruence with the combined and coalescent (i.e. from wASTRAL and CASTER-site) outputs (Fig. 1; Supplementary Data Figs S3–S5), we considered the results obtained from the analyses of the Supercontig dataset as our best result and all the discussion will be based on this dataset. The phylogenetic trees obtained using the Supercontig dataset in a combined matrix, through wASTRAL, and through CASTER-site, are very similar. Considering the overall topology and the support obtained for the branches, we used the wASTRAL tree obtained using the Supercontig dataset as the main coalescent result and all downstream results used this tree as input or reference.

### Reticulated evolution

**Fig. 2.**
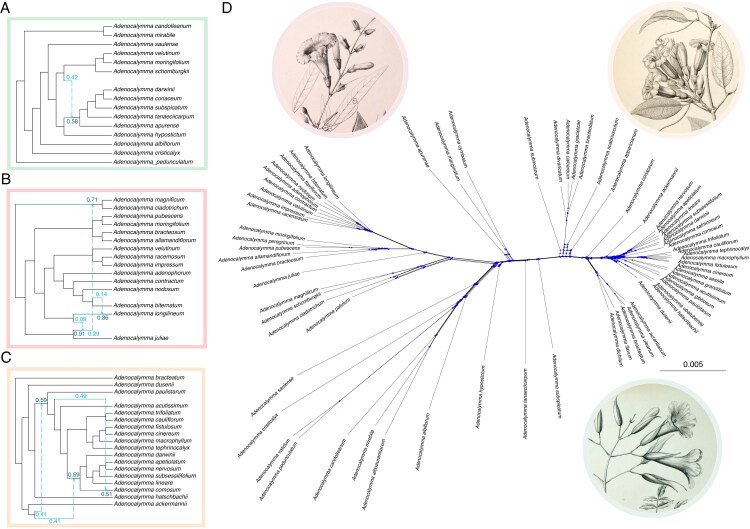
Reticulations inferred for the subtrees: (A) ‘Early Diverging Lineages’ (green), (B) ‘Amazon Forest Lineages’ (red) and (C) ‘Atlantic Forest Lineages’ (orange) using PhyloNetworks. Introgression events <0.1 were omitted. (D) A network sampling all the species of the genus *Adenocalymma* was also inferred, with subclades highlighted following the same colour scheme. Original Ilustrations from Flora Brasiliensis (Schumann,1894).

Three subtrees were tested using the 661 gene trees. For the ‘Early Diverging Lineages’, one reticulation was obtained, with minor hybrid edge (*γ* < 0.5) of 0.42 (Fig. 2). Gene flow was observed between the minor hybrid edge of the lineage leading to Clade 4 (0.42) with the major hybrid edge leading to Clade 5 (Fig. 2A). For the ‘Amazon Forest Lineage’, three reticulations were obtained, with two reticulations showing a minor hybrid edge over 0.1. Gene flow was observed between the minor hybrid edge leading to *A. biternatum* (A.Samp.) L.G.Lohmann (0.14) and a major hybrid edge leading to *A. longilineum* (A.Samp.) L.G.Lohmann, and between the minor hybrid edge leading to *A. juliae* (A.H.Gentry) L.G.Lohmann (0.29) and a major hybrid edge leading to the clade (*A. cladotrichum* (Sandwith) L.G.Lohmann, *A. magnificum* Mart. ex DC.) (Fig. 2B). For the ‘Atlantic Forest Lineage’, three reticulations were found, all showing a minor hybrid edge above 0.1. Two gene flow events were observed between the minor hybrid edge leading to *A. ackermannii* Bureau ex K.Schum. (0.41), and two different comprehensive clades inside Clade 6. Gene flow was also observed between the minor hybrid phantom lineage sister to a clade comprising *A. acutissimum* and nested species, and *A. comosum* DC. (Fig. 2C). The effects of the reticulation events described above are reflected in the broad network-like reticulation in the neighbour-net tree. Many reticulations were observed at the base of all three lineages discussed above, especially at the base of the ‘Atlantic Forest Lineage’ (Fig. 2D).

### Divergence time and diversification

**Fig. 3.**
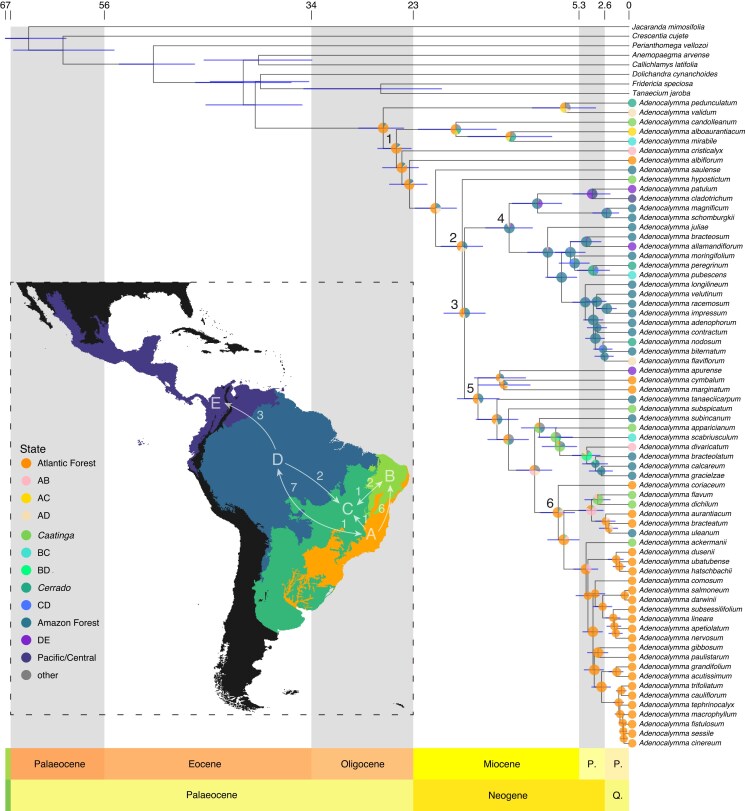
Bayesian dated tree using the wASTRAL tree as topological constraint. Bars over nodes are the 95 % HPD range. Map shows the biogeographical regions used in this study. Coloured circles at tips indicate the geographical range of each extant species. Pie charts over nodes represent the relative probabilities of ancestral ranges inferred using RevBayes and a time-stratified approach.

The time-calibrated tree of *Adenocalymma* and outgroups was inferred using a Bayesian approach in MCMCTree. The stem age of *Adenocalymma* was dated to the Eocene [mean age, 95 % highest posterior density (HPD): 39.89, 34.18–44.67 Ma], and the crown age to the Oligocene (mean, 95 % HPD: 26.23, 24.05–28.85 Ma) (Table 2; Fig. 3). Crown divergences of the major clades occurred between the Late Oligocene and Late Miocene: Clade 1 originated in the Late Oligocene (mean, 95 % HPD: 24.84, 23.26–26.8 Ma); Clade 2 and 3 during the Early Miocene (means 17.80 and 17.56 Ma, respectively; 95 % HPDs: 15.63–20.05 and 15.36–19.73 Ma); Clade 4 in the Middle to Late Miocene (mean 12.79, 95 % HPD: 10.32–15.29 Ma); Clade 5 in the Early Miocene (mean 16.12; 95 % HPD: 11.05–16.48 Ma); and Clade 6 in the Late Miocene (mean 7.55; 95 % HPD: 5.82–9.42 Ma) (Table 2; Fig. 3). Most cladogenetic events within *Adenocalymma* occurred during the Pliocene and Pleistocene, particularly within Clades 4 and 6 (Fig. 3).

**Table 2.** Mean, minimum and maximum ages (Ma) estimated for each key clade of the phylogeny of *Adenocalymma*.

Clade	Age estimated	Min.	Max.
Stem *Adenocalymma*	39.89	34.18	44.67
MRCA of *Adenocalymma*	26.23	24.05	28.85
MRCA of Clade 1	24.84	23.26	26.8
MRCA of Clade 2	17.8	15.63	20.05
MRCA of Clade 3	17.56	15.36	19.73
MRCA of Clade 4	12.79	10.32	15.29
MRCA of Clade 5	16.12	11.05	16.48
MRCA of Clade 6	7.55	5.82	9.42

MRCA, most recent common ancestor.

**Fig. 4.**
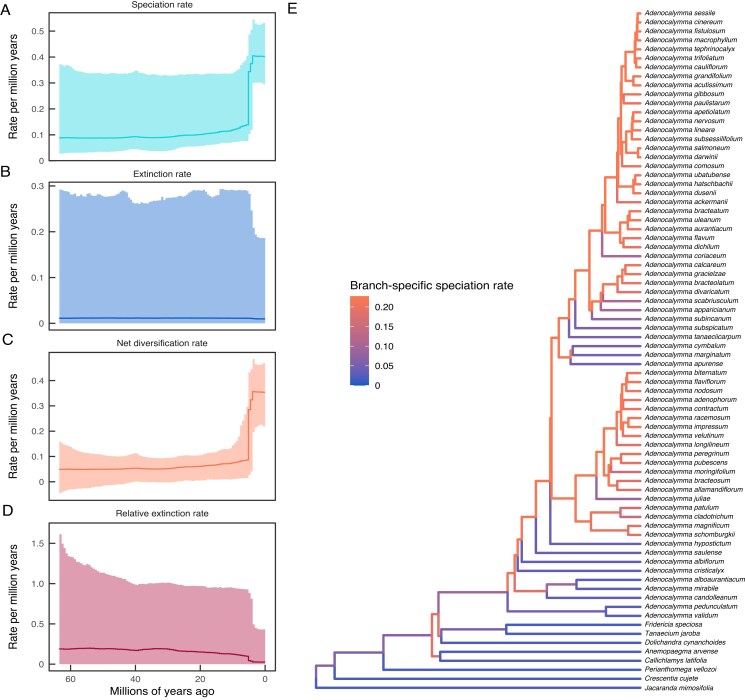
Diversification analysis in *Adenocalymma*. (A–D) Results from the episodic birth–death (EBD) model with 100 time intervals showing changes in speciation and extinction rates, and net diversification (speciation minus extinction) and relative extinction (extinction/speciation ratio) rates over time. Shaded regions show the 90 % credible intervals for the rate. (E) Changes in speciation rates among major lineages within *Adenocalymma*, estimated using a multistate SSE birth–death model (LSBDS).

The EBD analysis (Fig. 4) shows a pattern of increase in diversification rate during the last 5 Ma, mainly because of a sharp increase in the speciation rate, and a corresponding decrease in the relative extinction rate. The LSBDS analysis inferred high rates of net diversification throughout the ‘backbone’ of the *Adenocalymma* phylogeny, with particularly high rates inside Clades 4 and 6. The clade comprising (*A. divaricatum* Miers, (*A. bracteolatum* DC., (*A. calcareum* Udulutsch & P.Dias, *A. gracielzae* A.H.Gentry))) also shows high rates of net diversification. Low rates of net diversification are restricted to branches leading to species or small clades (Fig. 4).

### Historical biogeography

The single matrix and time-stratified approaches were compared using log-likelihoods (−111.1708 and −107.3128, respectively). In the following, we focus on the time-stratified approach (Fig. 3), noting differences with the single-matrix approach where informative. The ancestral area of *Adenocalymma* was inferred as the Atlantic Forest with high support (PP = 0.91). For Clade 1, the probability is lower (PP = 0.78). The results for the remaining early-diverging lineages within *Adenocalymma* recovered most-recent common ancestors (MRCAs) occurring in the Atlantic Forest with PP > 0.5 in all scenarios. If we consider the ancestral ranges inferred using the single-matrix approach, the crown node of *Adenocalymma* and all other early diverging lineages within the genus have probabilities below 0.5 and recovered the Caatinga as the most probably ancestral area for most of the nodes (Supplementary Data Fig. S6). Ancestral areas for Clades 2 and 3 were equivocal, with the highest probability being assigned to either the Amazon Forest or Atlantic Forest. The Amazon Forest was inferred as the ancestral area at the crown node of Clade 4 (PP = 0.76). The ancestral range of the crown node for Clade 5 is equivocal, with probabilities shared among Amazon and Atlantic Forests. The most probable ancestral area of the crown node of Clade 6 is the Atlantic Forest with a PP = 71. The Atlantic Forest also appeared as the most probable ancestral area for most nodes within Clade 6 (Fig. 3).

Clear events of vicariance (e.g. with PP > 0.5 for all elements involved in the range split) were rare within *Adenocalymma* and two examples can be described: (i) the clade comprising (*A. divaricatum*, (*A. bracteolatum*, (*A. calcareum*, *A. gracielzae*))), with a Caatinga + Amazon Forest ancestral area for the crown node (PP = 0.59), while *A. divaricatum* is endemic to the Caatinga, and the clade (*A. bracteolatum*, (*A. calcareum*, *A. gracielzae*)) is endemic to the Amazon Forest (PP = 0.87); and (ii) the ancestral area at the crown node of the clade comprising ((*A. dichilum* A.H.Gentry, *A. flavum* Mart. ex DC.), (*A. aurantiacum* Udulutsch & Assis, (*A. bracteatum* DC., *A. uleanum* Kraenzl.))) was Atlantic Forest + Caatinga (PP = 0.74), while the ancestral areas of the crown nodes of the sister clades, (*A. aurantiacum*, (*A. bracteatum*, *A. uleanum*)) and (*A. dichilum*, *A. flavum*), were the Atlantic Forest and Caatinga, respectively (PP = 0.83 and PP = 0.79). Dispersal and range shifts within *Adenocalymma* were very common. Using stochastic character mapping results (Supplementary Data Fig. S7), we counted the minimum number of events among ecoregions (ignoring episodic transitions within long branches). The most common range transitions are from the Atlantic to the Amazon Forest with seven events, followed by transitions from the Atlantic Forest to Caatinga with six events; the Amazon Forest to the Pacific Domain and Central America with three events; from the Amazon Forest to the Cerrado and from the Cerrado to the Caatinga with two events; and from the Amazon to the Atlantic Forest and from the Caatinga to the Cerrado with a single event (Fig. 3; Fig. S7).

Considering the inferred ranges for the species and clades involved in introgression, the event inferred in the ‘Early Diverging Lineages’ is largely equivocal, and it is impossible to pinpoint the exact area where it occurred. The ‘Amazon Forest Lineages’ and ‘Atlantic Forest Lineages’ clearly involved events between sympatric lineages (Figs 2 and 3).

## DISCUSSION

### The phylogeny of *Adenocalymma*

The phylogeny of *Adenocalymma* recovered here using nuclear data and various methodological approaches reveals largely congruent patterns and many shared clades (Fig. 1; Supplementary Data Fig. S5). Similar topological patterns were observed in the trees derived from the reticulated analysis using PhyloNet, despite introgression (Fig. 2). Additionally, the wASTRAL analysis using hundreds of independent nuclear genes recovered clades that are nearly identical in topology to those obtained from plastome, rDNA and mitochondrial genome data, encompassing both deep and shallow evolutionary relationships (Fig. 1; Fonseca and Lohmann, 2018, 2020). The topological similarities recovered across methods and genomes are striking, corroborating the robustness of the molecular data for inferring the phylogeny of *Adenocalymma* (Fonseca and Lohmann, 2018, 2020) and the Bignoniaceae as a whole (Lohmann, 2006; Olmstead *et al*., 2009; Fonseca *et al*., 2023). Such high congruence is uncommon in other plant groups, including *Buddleja* Houst. ex L. (Yang *et al*., 2023), cotton (*Gossypium* L.; Xu *et al*., 2024), figs (*Ficus* L.; Gardner *et al*., 2023), peatmoss (*Sphagnum* L.; Meleshko *et al*., 2021), strawberries (*Fragaria* L.; Feng *et al*., 2023) and *Polemonium* L. (Rose *et al*., 2021). By contrast, incongruences between genomes and methods have been documented for entire families, such as Brassicaceae (Hendriks *et al*., 2023) and Fagaceae (Zhou *et al*., 2022), reflecting notable cyto-nuclear divergences, often interpreted as evidence of gene flow within lineages. Near-identical cyto-nuclear topologies have been observed in only a few instances, such as in Scrophulariaceae (Villaverde *et al*., 2023).

Detailing the phylogenetic relationships in *Adenocalymma*, the genus was recovered as monophyletic for the Supercontig dataset in the combined and wASTRAL analyses, leading to topologies with respectively maximum values of UFBoot and LPP (Fig. 1). The clade comprising *A. pedunculatum* and *A. validum* always emerged as sister to the rest of the genus, with maximum support in all scenarios (Fig. 1). The placement of taxa located at the tree base was consistent across the trees recovered from the combined, wASTRAL and reticulated analyses (Figs 1 and 2). The only remarkable difference among the nuclear results (combined, ILS and ILS/introgression aware approaches; Figs 1 and 2) and the cytoplasmatic genomes (Fonseca and Lohmann, 2018, 2020) is the position of *A. hypostictum*. The result from PhyloNet clearly show an event of introgression between the lineage leading to Clade 4, or the minor hybrid edge (*γ* = 0.42), and the lineage leading to Clade 5, or the major hybrid edge, explaining the discordance between the nuclear (Fig. 1) and cytoplasmatic trees (Fonseca and Lohmann, 2018, 2020). Relationships within Clade 4 are congruent and highly supported across analyses, with maximum UFBoot and LPP support. Incongruences within Clade 4 are minimal among genome compartments and poorly supported in the tree resulting from the analyses of the plastome or mitochondrial datasets (Fonseca and Lohmann, 2018, 2020). The placement of Clades 5 and 6 is generally congruent across methods and genome compartments (Figs 1 and 2; Fonseca and Lohmann, 2018, 2020). The topological differences within Clade 5 between the position of *A. apparicianum* and *A. subincanum* can be explained by ILS, but is poorly supported in both combined and wASTRAL trees. The position of the clade (*A. gibbosum*, *A. paulistarum*) also varied between the combined and wASTRAL results and is possibly explained by ILS, but is poorly supported in the wASTRAL tree (Fig. 1).

### The processes behind gene tree discordance

The tree-like nature of evolution has been challenged by empirical evidence and methodological advances (Rokas *et al*., 2003; Shen *et al*., 2021), complicating attempts to translate phylogeny into classification when there is conflict among gene trees (e.g. Morales-Briones *et al*., 2021; Jin *et al*., 2023). Recent studies have proposed a set of tools and methods to assist taxonomists/systematists in decision-making in such situations (e.g. inference of ancestral states on networks; Karimi *et al*., 2020). The field of phylogenetics has its roots in systematics (Hennig, 1966), and has been traditionally used to support classifications. Recent phylogenetic studies reveal complicated and intertwined evolutionary processes that have shaped biodiversity (Soltis and Soltis, 2009), challenging phylogeny-based classifications. Even though confounding signals of ILS and introgression were recovered in *Adenocalymma*, we were able to infer a robust phylogenetic hypothesis for the genus using hundreds of nuclear genes (Figs 1 and 2) that corroborated an earlier classification based on plastid data (Fonseca and Lohmann, 2018, 2019).

ILS was fully addressed using wASTRAL, CASTER-site and PhyloNet. The topologies were generally similar, with poorly supported incongruences, minimizing the role of ILS in *Adenocalymma* (Figs 1 and 2, Supplementary Data Fig. S5; Mirarab *et al*., 2014; Mirarab, 2023; Solís-Lemus, 2023). The role of introgression in the evolutionary history of *Adenocalymma* was evaluated using implicit and explicit approaches. Using PhyloNet, we identified introgression with an explicit tree-like tool, which recovered seven reticulation events within the ‘Early Diverging Lineages’, ‘Amazon forest Lineages’ and ‘Atlantic forest Lineages’. These events ranged from 20 Ma to the present (Figs 2 and 3), with most occurring in the Late Miocene or Pliocene. Introgression at relatively recent times has been observed in some plant clades, such as *Rosa* (Debray *et al*., 2022) and wheat relatives (Glémin *et al*., 2019). While deep reticulation is thought to be common in plants (Stull *et al*., 2023), genetic evidence for it relies on high rates of ancient gene flow and potentially on positive selection favouring hybrid genotypes (Suvorov *et al*., 2022). The role of introgression in shaping *Adenocalymma* evolution is also highlighted when an implicit approach is considered (Fig. 3). Reticulations are observed on many branches, including the ‘backbone’ of the tree. However, the result is far less complex than in extreme cases, such as the putative syngameon *Quercus* (Hipp *et al*., 2020).

Multiple processes may drive gene tree discordance simultaneously (Page and Charleston, 1997; Rokas *et al*., 2003; Yu *et al*., 2011; Solís-Lemus *et al*., 2016), especially ILS, introgression and horizontal gene transfer, although the last of these is not generally observed in eukaryotes (Galtier and Daubin, 2008; Keeling and Palmer, 2008). Horizontal gene transfer is also uncommon in plants and mostly associated with intimate species interactions through parasitic, symbiotic, epiphytic, entophytic and grafting interactions (Gao *et al*., 2014). Even though most *Adenocalymma* species are lianas and are in intimate contact with other woody plants, there is no evidence of horizontal gene transfer in the clade. Gene tree conflict can arise from non-biological sources, including stochastic error in gene alignment and tree inference, poorly sequenced or assembled data, or biases from mis-modelled DNA evolution (Pamilo and Nei, 1988; Doyle, 1992; Shen *et al*., 2021). Given the limited roles of ILS or introgression within the focal group, it is likely that non-biological factors contributed substantially to observed gene discordance. High levels of gene-tree incongruence in some branches may reflect other processes, such as low molecular variability in some genes. This pattern may extend to the entire Bignoniaceae, where similarly high levels of gene tree incongruence have been reported (Fonseca *et al*., 2023).

### *Adenocalymma* through space and time

The stem node of *Adenocalymma* was recovered with a mean age of 39.89 Ma (95 % HPD: 34.18–44.67 Ma), while the crown node was recovered with a mean age of 26.23 Ma (95 % HPD: 24.05–28.85 Ma) (Table 2; Fig. 3). The ages inferred for *Adenocalymma* based on a phylogeny of tribe Bignonieae are older (Lohmann *et al*., 2013), but the 95 % HPDs of the crown node in this study (Fig. 3) and in the previous result overlap (Lohmann *et al*., 2013). Here we placed the initial evolution of *Adenocalymma* in the Eocene and the diversification of the crown group of the genus in the late Oligocene. *Adenocalymma* diversified throughout the Oligocene, Miocene, Pliocene and Pleistocene (Fig. 3). The divergence time estimates are based on an extensive sampling of taxa (68 or 87 % of all known species) and markers (661 nuclear genes). A striking result of this study is the relatively recent diversification within the genus, with Clades 4 and 6 showing maximum net diversification rates since the start of the Pliocene (5.3 Ma) and continuing through the Pleistocene (Figs 3 and 4).

The formation of the Dry Diagonal was explicitly tested in the model, and the ancestral range reconstruction using different dispersal matrices through time is clearly favoured compared to the simple model. The biogeographical reconstruction suggests that *Adenocalymma* originated in the Atlantic Forest, later expanding to the Amazon Forest, Caatinga and Cerrado. This result corroborates a previous inference that recovered eastern South America as the possible ancestral area for the clade and the entire tribe (Lohmann *et al*., 2013). Most nodes along the backbone of the genus have the highest probabilities of occurring in the Atlantic Forest, with the exception of Clade 3, whose crown node is more likely to have occurred in the Amazon Forest (Fig. 3). At least seven dispersals from the Atlantic Forest into the Amazon Forest were inferred, with only a single event in the opposite direction (Fig. 3; Supplementary Data Fig. S7). The Amazon Forest is widely recognized as a source biome for many different groups that dispersed to the Atlantic Forest (Antonelli *et al*., 2018), including the plant tribe Protieae (Fine *et al*., 2014), and the genera *Inga* Mill. (Nicholls *et al*., 2025) and *Mansoa* DC. (Sun *et al*., 2025). However, dispersals from the Atlantic to the Amazon Forests were common in angiosperms, such as in the genera *Ficus* L. (Machado *et al*., 2018) and *Leandra* Raddi (Reginato and Michelangeli, 2019). *Adenocalymma flaviflorum* and *A. validum*, two species with disjunct distribution in the Amazon and Atlantic Forests, recovered an ancestral state in the Amazon and Atlantic Forests, respectively (Fig. 3). For these two species, dispersal to the new biome probably involved recent connections between both rainforests or ‘stepping stones’ of humid forest patches and gallery forests through northeast Brazil. This route is common across many groups of organisms and is considered recent in geological time (Batalha-Filho *et al*., 2013; Ledo and Colli, 2017).

Direct transitions from the Atlantic Forest to the Caatinga were also common within the focal genus, corroborating previous findings across multiple plant clades (Fernandes *et al*., 2022). Occupations of the Pacific Domain (Morrone *et al*., 2022) and Central America from the Amazon Forest occurred at least three times inside *Adenocalymma*, a pattern commonly found in other plant groups (e.g. *Inga*; Nicholls *et al*., 2025). The dispersals to Central America possibly occurred during the Pliocene around 3.5 Ma (Supplementary Data Fig. S7). This age is before full formation of the Panama Isthmus and the Great American Biotic Exchange (around 2.8 Ma; O’Dea *et al*., 2016), but is in line with dispersal dates inferred for other plant groups (Bacon *et al*., 2015). Clade 4 includes most species previously included in *Memora* (Lohmann and Taylor, 2014). For many years, the refugia hypothesis (Haffer, 1969) was the most widely discussed explanation for diversification in the Amazonian region. It proposes that Pleistocene dry periods fragmented forests into areas of open dry vegetation, such as savannas, creating isolated refugia where new species could arise from former widespread ancestral species. However, this hypothesis has not been supported by empirical data and modelling studies (Nelson *et al*., 1990; Rull, 2011; Rocha and Kaefer, 2019). The diversification of Amazonian *Adenocalymma* lineages adds to this body of evidence, occurring primarily from the Late Miocene to the Pliocene, with no cladogenesis observed during the Pleistocene (Fig. 3).

*In situ* diversification of Clade 4 in the Amazon Domain, along with other lineages of the early diverging species and Clade 5, matches the age of the establishment of the Pebas and Acre lacustrine systems. Orogenesis of the central and northern Andes led to the formation of swamps and lakes in the eastern portion of the Amazon Domain during the Miocene and Pliocene. River formation and capture was intense during this period (Hoorn *et al*., 2010). These new and heterogeneous landscapes would have fragmented populations and clades, boosting the diversification of the biota during the Miocene (Antonelli and Sanmartín, 2011). This explanation matches well with the current geographical distribution and temporal diversification of Amazonian lineages of *Adenocalymma* and reveals the putative role of Andean orogenesis for the diversification of the genus. As most of the species of *Adenocalymma* that were not sampled occur in the Andean regions of Central America, and/or Colombia (e.g. *A. chocoense* A.H.Gentry), increased sampling would probably contribute additional evidence for the role of Andean orogenesis to the history of *Adenocalymma* (Fonseca and Lohmann, 2019). Amazonian diversification was previously observed in other Bignonieae clades such as *Amphilophium* Kunth emend L.G.Lohmann (Thode *et al*., 2019), *Anemopaegma* Mart. ex Meisn. (Calió *et al*., 2022) and *Tynanthus* Miers (Medeiros and Lohmann, 2015). Dispersals or range shifts from the Amazon Forest to the Dry Diagonal occurred at least twice within *Adenocalymma*. These events occurred during the Pliocene, when the Cerrado was probably well established, corroborating results obtained with other plant groups (Simon *et al*., 2009).

Clade 5 comprises species found across all areas, with most endemic to the Atlantic Forest. Vicariance was inferred twice in this clade, one involving the Atlantic Forest and the Caatinga and the other involving the Amazon Forest and the Caatinga (Fig. 3). The HPDs of the nodes involved were within the Pliocene, and the ages match the putative establishment of the current Caatinga flora around 5–3 Ma (Fernandes *et al*., 2022). Clade 6 comprises species that are restricted to the Atlantic Forest, with the exceptions of three species endemic to the Caatinga and *A. uleanum* from the Amazon Forest. Diversification in this clade occurred during the Pliocene and Pleistocene, leading to a sharp increase in speciation and net diversification rates over the last 5 Ma (Figs 3 and 4). It is the only internal clade of *Adenocalymma* with cladogenesis during the Pleistocene, and many species are narrowly endemic (Supplementary material S2). Combined, these results suggest that refugia were important for the diversification of this clade. Endemism in the Atlantic Forest has often been related to forest refugia during the Pleistocene (Carnaval and Moritz, 2008) and proposed forest refugia are supported by climatic and genetic variability (e.g. Turchetto-Zolet *et al*., 2016; Leal *et al*., 2018; Mäder *et al*., 2021). Vicariant processes probably played a role in the diversification of many plant groups, including within the Atlantic Forest endemic clade of *Adenocalymma*, However, our sampling at the species level does not allow us to infer signatures of vicariant processes within the Atlantic Forest and a population-level sampling and further regionalization will be necessary to formally test hypotheses of refugia. Biological drivers of diversification putatively played a role within Clade 6, given the variability in floral morphologies (Fonseca and Lohmann, 2018) and putative pollination strategies within the clade.

## CONCLUSION

The genomic revolution started with the development of high-throughput sequencing platforms, which allowed the comparison of thousands to millions of independent bases for phylogenomic purposes. New challenges were raised by the new data, going beyond the obvious accumulation of data and the increased time for analyses. ILS and introgression, among other processes, were widespread throughout the Tree of Life. Empirical evidence accumulated, showing the importance of ILS and, in particular, hybridization/introgression in plants. However, this evidence concentrates on the temperate flora and few tropical lineages were sampled. Here, we used the successful Bignoniaceae bait kit to explore the phylogeny of the Neotropical clade *Adenocalymma* and showed the robust phylogenetic signal present in the data. Similar topologies were obtained through different genome compartments and methods. ILS and introgression were recovered, but with a minor role inside the genus. This is a striking result and should be explored in other tropical plant lineages, notably the flora of tropical America. Even though the Neotropics harbour the most diverse flora in the world, phylogenomic studies for Neotropical plants remain elusive.

The timing and spatial distribution of *Adenocalymma* evolution were also explored here, and the results are congruent with those from other plant lineages. The genus emerged in the Atlantic Forest, with multiple dispersals and range shifts into the Amazon Forest and the Caatinga. The timing corroborates previous findings on the age of the Dry Diagonal, with occupations of this region during the Late Miocene and Pliocene. Diversification *in situ* in the Amazon and Atlantic Forests was also observed within the two major internal clades. These biogeographical results should be explored for other tropical plant lineages from South America, comparing the ages of diversification of these clades and the congruence with geological and model-based evidence already available.

## Supplementary Material

mcag087_Supplementary_Data

## Data Availability

Scripts for nuclear locus assembly and processing are available on GitHub (https://github.com/luizhhziul/Adenocalymma). Raw reads have been deposited in the NCBI Sequence Read Archive (BioProject PRJNA909066).

## References

[mcag087-B1] Antonelli A, Sanmartín I. 2011. Why are there so many plant species in the Neotropics? Taxon 60: 403–414. doi:10.1002/tax.602010

[mcag087-B2] Antonelli A, Zizka A, Carvalho FA, et al 2018. Amazonia is the primary source of Neotropical biodiversity. Proceedings of the National Academy of Sciences of the United States of America 115: 6034–6039. doi:10.1073/pnas.1713819115 29760058 PMC6003360

[mcag087-B3] Bacon CD, Silvestro D, Jaramillo C, Smith BT, Chakrabarty P, Antonelli A. 2015. Biological evidence supports an early and complex emergence of the Isthmus of Panama. Proceedings of the National Academy of Sciences of the United States of America 112: 6110–6115. doi:10.1073/pnas.1423853112 25918375 PMC4434730

[mcag087-B4] Bankevich A, Nurk S, Antipov D, et al 2012. SPAdes: a new genome assembly algorithm and its applications to single-cell sequencing. Journal of Computational Biology 19: 455–477. doi:10.1089/cmb.2012.0021 22506599 PMC3342519

[mcag087-B5] Batalha-Filho H, Fjeldså J, Fabre PH, Miyaki C. 2013. Connections between the Atlantic and the Amazonian forest avifaunas represent distinct historical events. Journal of Ornithology 154: 41–50. doi:0.1007/s10336-012-0866-7

[mcag087-B6] BFG [The Brazil Flora Group] . 2022. Brazilian Flora 2020: leveraging the power of a collaborative scientific network. Taxon 71: 178–198. doi:10.1002/tax.12640

[mcag087-B7] Bolger AM, Lohse M, Usadel B. 2014. Trimmomatic: a flexible trimmer for Illumina sequence data. Bioinformatics 30: 2114–2120. doi:10.1093/bioinformatics/btu170 24695404 PMC4103590

[mcag087-B8] Boom AF, Migliore J, Kaymak E, Meerts P, Hardy OJ. 2021. Plastid introgression and evolution of African miombo woodlands: new insights from the plastome-based phylogeny of *Brachystegia* trees. Journal of Biogeography 48: 933–946. doi:10.1111/jbi.14051

[mcag087-B9] Borowiec ML . 2016. AMAS: a fast tool for alignment manipulation and computing of summary statistics. PeerJ 4:. doi:10.7717/peerj.1660 PMC4734057 26835189

[mcag087-B10] Borowiec ML . 2019. Spruceup: fast and flexible identification, visualization, and removal of outliers from large multiple sequence alignments. Journal of Open Source Software 4:1635. doi:10.21105/joss.01635

[mcag087-B11] Bryant D, Moulton V. 2004. Neighbor-net: an agglomerative method for the construction of phylogenetic networks. Molecular Biology and Evolution 21: 255–265. doi:10.1093/molbev/msh018 14660700

[mcag087-B12] Calió MF, Thode VA, Bacon CD, Silvestro D, Antonelli A, Lohmann LG. 2022. Spatio-temporal evolution of the catuaba clade in the Neotropics: morphological shifts correlate with habitat transitions. Journal of Biogeography 49: 1086–1098. doi:10.1111/jbi.14368

[mcag087-B13] Cannon CH, Petit RJ. 2020. The oak syngameon: more than the sum of its parts. New Phytologist 226: 978–983. doi:10.1111/nph.16091 31378946

[mcag087-B14] Carnaval AC, Moritz C. 2008. Historical climate modelling predicts patterns of current biodiversity in the Brazilian Atlantic forest. Journal of Biogeography 35: 1187–1201. doi:10.1111/j.1365-2699.2007.01870.x

[mcag087-B15] Cheng H, Sinha A, Cruz FW, et al 2013. Climate change patterns in Amazonia and biodiversity. Nature Communications 4: 1411. doi:10.1038/ncomms2415 23361002

[mcag087-B16] Costa CC, Hampe A, Ledru MP, et al 2018. Biome stability in South America over the last 30 kyr: inferences from long-term vegetation dynamics and habitat modelling. Global Ecology and Biogeography 27: 285–297. doi:10.1111/geb.12694

[mcag087-B17] Debray K, Le Paslier MC, Bérard A, et al 2022. Unveiling the patterns of reticulated evolutionary processes with phylogenomics: hybridization and polyploidy in the genus *Rosa*. Systematic Biology 71: 547–569. doi:10.1093/sysbio/syab064 34329460

[mcag087-B18] Dos Reis M, Donoghue PC, Yang Z. 2016. Bayesian molecular clock dating of species divergences in the genomics era. Nature Reviews: Genetics 17: 71–80. doi:10.1038/nrg.2015.8 26688196

[mcag087-B19] Dos Reis M, Yang Z. 2019. Bayesian molecular clock dating using genome-scale datasets. In: Anisimova M. ed. Evolutionary genomics: statistical and computational methods. Methods in molecular biology, vol. 1910. New York: Humana Press, 309–330. doi:10.1007/978-1-4939-9074-0_10 31278669

[mcag087-B20] Doyle JJ . 1992. Gene trees and species trees: molecular systematics as one-character taxonomy. Systematic Botany 17: 144–163. doi:10.2307/2419070

[mcag087-B21] Edwards EJ, Osborne CP, Strömberg CA, et al 2010. The origins of C_4_ grasslands: integrating evolutionary and ecosystem science. Science 328: 587–591. doi:10.1126/science.117721 20431008

[mcag087-B22] Feng C, Wang J, Liston A, Kang M. 2023. Recombination variation shapes phylogeny and introgression in wild diploid strawberries. Molecular Biology and Evolution 40:msad049. doi:10.1093/molbev/msad049 36864629 PMC10015625

[mcag087-B23] Fernandes MF, Cardoso D, Pennington RT, de Queiroz LP. 2022. The origins and historical assembly of the Brazilian Caatinga seasonally dry tropical forests. Frontiers in Ecology and Evolution 10:723286. doi:10.3389/fevo.2022.723286

[mcag087-B24] Fine PVA, Zapata F, Daly DC. 2014. Investigating processes of Neotropical rain forest tree diversification by examining the evolution and historical biogeography of the Protieae (Burseraceae). Evolution 68: 1988–2004. doi:10.1111/evo.12414 24689871

[mcag087-B25] Fonseca LHM, Carlsen MM, Fine PV, Lohmann LG. 2023. A nuclear target sequence capture probe set for phylogeny reconstruction of the charismatic plant family Bignoniaceae. Frontiers in Genetics 13:1085692. doi:10.3389/fgene.2022.1085692 36699458 PMC9869424

[mcag087-B26] Fonseca LHM, Lohmann LG. 2018. Combining high-throughput sequencing and targeted loci data to infer the phylogeny of the ‘*Adenocalymma-Neojobertia*’ clade (Bignonieae, Bignoniaceae). Molecular Phylogenetics and Evolution 123: 1–15. doi:10.1016/j.ympev.2018.01.023 29432850

[mcag087-B27] Fonseca LHM, Lohmann LG. 2019. An updated synopsis of *Adenocalymma* (Bignonieae, Bignoniaceae): new combinations, synonyms, and lectotypifications. Systematic Botany 44: 893–912. doi:10.1600/036364419X15710776741341

[mcag087-B28] Fonseca LHM, Lohmann LG. 2020. Exploring the potential of nuclear and mitochondrial sequencing data generated through genome-skimming for plant phylogenetics: a case study from a clade of neotropical lianas. Journal of Systematics and Evolution 58: 18–32. doi:10.1111/jse.12533

[mcag087-B29] Freyman WA, Höhna S. 2019. Stochastic character mapping of state-dependent diversification reveals the tempo of evolutionary decline in self-compatible Onagraceae lineages. Systematic Biology 68: 505–519. doi:10.1093/sysbio/syy078 30476308

[mcag087-B30] Galtier N, Daubin V. 2008. Dealing with incongruence in phylogenomic analyses. Philosophical Transactions of the Royal Society 363: 4023–4029. doi:10.1098/rstb.2008.0144 PMC2607408 18852109

[mcag087-B31] Gao C, Ren X, Mason AS, et al 2014. Horizontal gene transfer in plants. Functional & Integrative Genomics 14: 23–29. doi:10.1007/s10142-013-0345-0 24132513

[mcag087-B32] Gardner EM, Bruun-Lund S, Niissalo M, et al 2023. Echoes of ancient introgression punctuate stable genomic lineages in the evolution of figs. Proceedings of the National Academy of Sciences of the United States of America 120:e2222035120. doi:10.1073/pnas.2222035120 37399402 PMC10334730

[mcag087-B33] Glémin S, Scornavacca C, Dainat J, et al 2019. Pervasive hybridizations in the history of wheat relatives. Science Advances 5:eaav9188. doi:10.1126/sciadv.aav9188 31049399 PMC6494498

[mcag087-B34] Haffer J . 1969. Speciation in Amazonian forest birds. Science 165: 131–137. doi:10.1126/sciadv.aav91 17834730

[mcag087-B35] Hendriks KP, Kiefer C, Al-Shehbaz IA, et al 2023. Global Brassicaceae phylogeny based on filtering of 1,000-gene dataset. Current Biology 33: 4052–4068.e6. doi:10.1016/j.cub.2023.08.026 37659415

[mcag087-B36] Hennig W . 1966. Phylogenetic systematics. Chicago: University of Illinois Press.

[mcag087-B37] Hernández RM, Jordan TE, Farjat AD, Echavarría L, Idleman BD, Heynolds JH. 2005. Age, distribution, tectonics, and eustatic controls of the Paranaense and Caribbean marine transgressions in southern Bolivia and Argentina. South American Earth Sciences 19: 495–512. doi:10.1016/j.jsames.2005.06.007

[mcag087-B38] Hipp AL, Manos PS, Hahn M, et al 2020. Genomic landscape of the global oak phylogeny. New Phytologist 226: 1198–1212. doi:10.1111/nph.16162 31609470

[mcag087-B39] Hoang DT, Chernomor O, Von Haeseler A, Minh BQ, Vinh LS. 2018. UFBoot2: improving the ultrafast bootstrap approximation. Molecular Biology and Evolution 35: 518–522. doi:10.1093/molbev/msx281 29077904 PMC5850222

[mcag087-B40] Höhna S . 2014. Likelihood inference of non-constant diversification rates with incomplete taxon sampling. PLoS One 9:e84184. doi:10.1371/journal.pone.0084184 24400082 PMC3882215

[mcag087-B41] Höhna S, Freyman WA, Nolen Z, Huelsenbeck JP, May MR, Moore BR. 2019. A Bayesian approach for estimating branch-specific speciation and extinction rates. *bioRxiv*. doi:10.1101/555805.

[mcag087-B42] Höhna S, Landis MJ, Heath TA, et al 2016. RevBayes: Bayesian phylogenetic inference using graphical models and an interactive model-specification language. Systematic Biology 65: 726–736. doi:10.1093/sysbio/syw021 27235697 PMC4911942

[mcag087-B43] Hoorn C, Lohmann LG, Boschman LM, Condamine FL. 2023. Neogene history of the Amazonian Flora: a perspective based on geological, palynological, and molecular phylogenetic data. Annual Review of Earth and Planetary Sciences 51: 419–446. doi:10.1146/annurev-earth-081522-090454

[mcag087-B44] Hoorn C, Wesselingh FP, ter Steege H, et al 2010. Amazonia through time: Andean uplift, climate change, landscape evolution, and biodiversity. Science 330: 927–931. doi:10.1126/science.119458 21071659

[mcag087-B45] Huang W, Zhang L, Columbus JT, et al 2022. A well-supported nuclear phylogeny of Poaceae and implications for the evolution of C_4_ photosynthesis. Molecular Plant 15: 755–777. doi:10.1016/j.molp.2022.01.015 35093593

[mcag087-B46] Jacobs BF, Kingston JD, Jacobs LL. 1999. The origin of grass-dominated ecosystems. Annals of Missouri Botanical Garden 86: 590–643. doi:10.2307/2666186

[mcag087-B47] Jaramillo CA, Cárdenas A. 2013. Global warming and Neotropical rainforests: a historical perspective. Annual Review of Earth and Planetary Sciences 41: 741–766. doi:10.1146/annurev-earth-042711-105403

[mcag087-B48] Jaramillo CA, Rueda MJ, Mora G. 2006. Cenozoic plant diversity in the Neotropics. Science 311: 1893–1896. doi:10.1126/science.112138 16574860

[mcag087-B49] Jin ZT, Hodel RG, Ma DK, et al 2023. Nightmare or delight: taxonomic circumscription meets reticulate evolution in the phylogenomic era. Molecular Phylogenetics and Evolution 189:107914. doi:10.1016/j.ympev.2023.107914 37666378

[mcag087-B50] Johnson MG, Gardner EM, Liu Y, et al 2016. HybPiper: extracting coding sequence and introns for phylogenetics from high-throughput sequencing reads using target enrichment. Applications in Plant Sciences 4:1600016. doi:10.3732/apps.1600016 PMC4948903 27437175

[mcag087-B51] Jombart T, Balloux F, Dray S. 2010. *Adephylo*: new tools for investigating the phylogenetic signal in biological traits. Bioinformatics 26: 1907–1909. doi:10.1093/bioinformatics/btq292 20525823

[mcag087-B52] Karimi N, Grover CE, Gallagher JP, Wendel JF, Ané C, Baum DA. 2020. Reticulate evolution helps explain apparent homoplasy in floral biology and pollination in baobabs (Adansonia; Bombacoideae; Malvaceae). Systematic Biology 69: 462–478. doi:10.1093/sysbio/syz073 31693158

[mcag087-B53] Katoh K, Standley DM. 2013. MAFFT multiple sequence alignment software version 7: improvements in performance and usability. Molecular Biology and Evolution 30: 772–780. doi:10.1093/molbev/mst010 23329690 PMC3603318

[mcag087-B54] Keeling PJ, Palmer JD. 2008. Horizontal gene transfer in eukaryotic evolution. Nature Reviews: Genetics 9: 605–618. doi:10.1038/nrg2386 18591983

[mcag087-B55] Kozlov AM, Darriba D, Flouri T, Morel B, Stamatakis A. 2019. RAxML-NG: a fast, scalable and user-friendly tool for maximum likelihood phylogenetic inference. Bioinformatics 35: 4453–4455. doi:10.1093/bioinformatics/btz305 31070718 PMC6821337

[mcag087-B56] Kubatko LS, Degnan JH. 2007. Inconsistency of phylogenetic estimates from concatenated data under coalescence. Systematic Biology 56: 17–24. doi:10.1080/10635150601146041 17366134

[mcag087-B57] Landis JB, Soltis DE, Li Z, et al 2018. Impact of whole-genome duplication events on diversification rates in angiosperms. American Journal of Botany 105: 348–363. doi:10.1002/ajb2.1060 29719043

[mcag087-B58] Leal BSS, Medeiros LR, Peres EA, et al 2018. Insights into the evolutionary dynamics of Neotropical biomes from the phylogeography and paleodistribution modeling of *Bromelia balansae*. American Journal of Botany 105: 1725–1734. doi:10.1002/ajb2.1167 30324691

[mcag087-B59] Ledo RMD, Colli GR. 2017. The historical connections between the Amazon and the Atlantic Forest revisited. Journal of Biogeography 44: 2551–2563. doi:10.1111/jbi.13049

[mcag087-B60] Li H, Durbin R. 2009. Fast and accurate short read alignment with Burrows–Wheeler transform. Bioinformatics 25: 1754–1760. doi:10.1093/bioinformatics/btp324 19451168 PMC2705234

[mcag087-B61] Lohmann LG . 2006. Untangling the phylogeny of neotropical lianas (Bignonieae, Bignoniaceae). American Journal of Botany 93: 304–318. doi:10.3732/ajb.93.2.304 21646191

[mcag087-B62] Lohmann LG, Bell CD, Calió MF, Winkworth RC. 2013. Pattern and timing of biogeographical history in the Neotropical tribe Bignonieae (Bignoniaceae). Botanical Journal of the Linnean Society 171: 154–170. doi:10.1111/j.1095-8339.2012.01311.x

[mcag087-B63] Lohmann LG, Taylor CM. 2014. A new generic classification of tribe Bignonieae (Bignoniaceae). Annals of the Missouri Botanical Garden 99: 348–489. doi:10.3417/2003187

[mcag087-B64] Machado AFP, Rønsted N, Bruun-Lund S, Pereira RAS, de Queiroz LP. 2018. Atlantic forests to the all Americas: biogeographical history and divergence times of Neotropical *Ficus* (Moraceae). Molecular Phylogenetics and Evolution 122: 46–58. doi:10.1016/j.ympev.2018.01.015 29371027

[mcag087-B129] Maddison WP, Midford PE, Otto SP. 2007. Estimating a binary character's effect on speciation and extinction. Systematic Biology 56: 701. doi:10.1080/10635150701607033 17849325

[mcag087-B66] Mäder G, Zamberlan PM, Segatto ALA, Stehmann JR, Bonatto SL, Freitas LB. 2021. When phylogeography meets niche suitability to unravel the evolutionary history of a shrub from the Brazilian Atlantic Forest. Botanical Journal of the Linnean Society 195: 77–92. doi:10.1093/botlinnean/boaa073

[mcag087-B67] Magee AF, Höhna S, Vasylyeva TI, Leache AD, Minin VN. 2020. Locally adaptive Bayesian birth-death model successfully detects slow and rapid rate shifts. PLoS Computational Biology 16:e1007999. doi:10.1371/journal.pcbi.1007999 33112848 PMC7652323

[mcag087-B68] Medeiros MCM, Lohmann LG. 2015. Phylogeny and biogeography of *Tynanthus* Miers (Bignonieae, Bignoniaceae). Molecular Phylogenetics and Evolution 85: 32–40. doi:10.1016/j.ympev.2015.01.010 25659336

[mcag087-B69] Meleshko O, Martin MD, Korneliussen TS, et al 2021. Extensive genome-wide phylogenetic discordance is due to incomplete lineage sorting and not ongoing introgression in a rapidly radiated bryophyte genus. Molecular Biology and Evolution 38: 2750–2766. doi:10.1093/molbev/msab063 33681996 PMC8233498

[mcag087-B70] Minh BQ, Hahn MW, Lanfear R. 2020. New methods to calculate concordance factors for phylogenomic datasets. Molecular Biology and Evolution 37: 2727–2733. doi:10.1093/molbev/msaa106 32365179 PMC7475031

[mcag087-B71] Mirarab S . 2023. Species tree estimation using ASTRAL: practical considerations. In: Kubatko L, Knowles LL. eds. Species tree inference: a guide to methods and applications. Princeton: Princeton University Press, 43–67.

[mcag087-B72] Mirarab S, Reaz R, Bayzid MS, Zimmermann T, Swenson MS, Warnow T. 2014. ASTRAL: genome-scale coalescent-based species tree estimation. Bioinformatics 30: i541–i548. doi:10.1093/bioinformatics/btu462 25161245 PMC4147915

[mcag087-B73] Mittermeier R, Gil P, Hoffman M, et al 2005. Hotspots revisited: Earth’s biologically richest and most endangered terrestrial ecoregions. Mexico City: CEMEX.

[mcag087-B74] Morales-Briones DF, Kadereit G, Tefarikis DT, et al 2021. Disentangling sources of gene tree discordance in phylogenomic datasets: testing ancient hybridizations in Amaranthaceae sl. Systematic Biology 70: 219–235. doi:10.1093/sysbio/syaa066 32785686 PMC7875436

[mcag087-B75] Morley RL . 2000. Origin and evolution of tropical rainforests. New York: Wiley.

[mcag087-B76] Morrone JJ, Escalante T, Rodriguez-Tapia G, Carmona A, Arana M, Mercado-Gómez JD. 2022. Biogeographic regionalization of the Neotropical region: new map and shapefile. Anais da Academia Brasileira de Ciências 94:e20211167. doi:10.1590/0001-3765202220211167 35107518

[mcag087-B77] Narváez-Gómez JP, Guedes TB, Lohmann LG. 2021. Recovering the drivers of sampling bias in Bignonieae (Bignoniaceae) and identifying priority areas for new survey efforts. Biodiversity and Conservation 30: 2319–2339. doi:10.1007/s10531-021-02195-7

[mcag087-B78] Nelson BW, Ferreira CAC, Da Silva MF, Kawasaki ML. 1990. Endemism centers, refugia and botanical collection density in Brazilian Amazonia. Nature 345: 714–716. doi:10.1038/345714a0

[mcag087-B79] Nguyen LT, Schmidt HA, Von Haeseler A, Minh BQ. 2015. IQ-TREE: a fast and effective stochastic algorithm for estimating maximum-likelihood phylogenies. Molecular Biology and Evolution 32: 268–274. doi:10.1093/molbev/msu300 25371430 PMC4271533

[mcag087-B80] Nicholls JA, Ringelberg JJ, Dexter KG, et al 2025. Continuous colonization of the Atlantic coastal rain forests of South America from Amazoĉnia. Proceedings: Biological Sciences 292:20241559. doi:10.1098/rspb.2024.1559 39837505 PMC11750371

[mcag087-B81] O’Dea A, Lessios HA, Coates AG, et al 2016. Formation of the Isthmus of Panama. Science Advances 2:e1600883. doi:10.1126/sciadv.1600883 27540590 PMC4988774

[mcag087-B82] Oliveira-Filho A, Ratter JA. 1995. A study of the origin of central Brazilian forests by the analysis of plant species distribution patterns. Edinburgh Journal of Botany 52: 141–194. doi:10.1017/S0960428600000949

[mcag087-B83] Oliveira PE, Barreto AMF, Suguio K. 1999. Late Pleistocene-Holocene climatic and vegetational history of the Brazilian Caatinga: the fossil dunes of the middle São Francisco River. Palaeogeography, Palaeoclimatology, Palaeoecology 152: 319–337. doi:10.1016/S0031-0182(99)00061-9

[mcag087-B84] Olmstead RG, Zjhra ML, Lohmann LG, Grose SO, Eckert AJ. 2009. A molecular phylogeny and classification of Bignoniaceae. American Journal of Botany 96: 1731–1743. doi:10.3732/ajb.0900004 21622359

[mcag087-B85] Page RD, Charleston MA. 1997. From gene to organismal phylogeny: reconciled trees and the gene tree/species tree problem. Molecular Phylogenetics and Evolution 7: 231–240. doi:10.1006/mpev.1996.0390 9126565

[mcag087-B86] Pamilo P, Nei M. 1988. Relationships between gene trees and species trees. Molecular Biology and Evolution 5: 568–583. doi:10.1093/oxfordjournals.molbev.a040517 3193878

[mcag087-B87] Payseur BA, Rieseberg LH. 2016. A genomic perspective on hybridization and speciation. Molecular Ecology 25: 2337–2360. doi:10.1111/mec.13557 26836441 PMC4915564

[mcag087-B88] Peres EA, Pinto-da-Rocha R, Lohmann LG, Michelangeli FA, Miyaki CY, Carnaval AC. 2020. Patterns of species and lineage diversity in the Atlantic rainforest of Brazil. In: Rull V, Carnaval AC. eds. Neotropical diversification: patterns and processes fascinating life sciences. Cham: Springer International Publishing, 415–447. doi:10.1007/978-3-03031167-4_16

[mcag087-B89] Perret M, Chautems A, Araujo AO, Salamim N. 2013. Temporal and spatial origin of Gesneriaceae in the New World inferred from plastid DNA sequences. Botanical Journal of the Linnean Society 171: 61–79. doi:10.1111/j.1095-8339.2012.01303.x

[mcag087-B90] Puttick MN . 2019. MCMCtreeR: functions to prepare MCMCtree analyses and visualize posterior ages on trees. Bioinformatics 35: 5321–5322. doi:10.1093/bioinformatics/btz554 31292621

[mcag087-B91] Ree RH, Smith SA. 2008. Maximum likelihood inference of geographic range evolution by dispersal, local extinction, and cladogenesis. Systematic Biology 57: 4–14. doi:10.1080/10635150701883881 18253896

[mcag087-B92] Reginato M, Michelangeli FA. 2019. Pleistocene range expansions might explain striking disjunctions between eastern Brazil, Andes and Mesoamerica in *Leandra* s.str. (Melastomataceae). Journal of Systematics and Evolution 57: 646–654. doi:10.1111/jse.12475

[mcag087-B93] Rieseberg LH . 1997. Hybrid origins of plant species. Annual Review of Ecology, Evolution, and Systematics 28: 359–389. doi:10.1146/annurev.ecolsys.28.1.359

[mcag087-B94] Rocha DG, Kaefer IL. 2019. What has become of the refugia hypothesis to explain biological diversity in Amazonia. Ecology and Evolution 9: 4302–4309. doi:10.1002/ece3.5051 31016006 PMC6468052

[mcag087-B95] Rokas A, Williams BL, King N, Carroll SB. 2003. Genome-scale approaches to resolving incongruence in molecular phylogenies. Nature 425: 798–804. doi:10.1038/nature02053 14574403

[mcag087-B96] Romero EJ . 1993. South American paleofloras. In: Goldblatt P. eds. Biological relationships between Africa and South America. New Haven: Yale University Press, 62–85. doi:10.2307/j.ctt22726mc

[mcag087-B97] Rose JP, Toledo CA, Lemmon EM, Lemmon AR, Sytsma KJ. 2021. Out of sight, out of mind: widespread nuclear and plastid-nuclear discordance in the flowering plant genus *Polemonium* (Polemoniaceae) suggests widespread historical gene flow despite limited nuclear signal. Systematic Biology 70: 162–180. doi:10.1093/sysbio/syaa049 32617587

[mcag087-B98] Rull V . 2011. Neotropical biodiversity, timing and potential drivers. Trends in Ecology & Evolution 26: 508–513. doi:10.1016/j.tree.2011.05.011 21703715

[mcag087-B99] Ruskin BG, Dávila FM, Hoke GD, Jordan TE, Astini RA, Alonso R. 2011. Stable isotope composition of middle Miocene carbonates of the Frontal Cordillera and Sierras Pampeanas: Did the Paranaense seaway flood western and central Argentina? Palaeogeography, Palaeoclimatology, Palaeoecology 308: 293–303. doi:10.1016/j.palaeo.2011.05.033

[mcag087-B100] Schliep KP . 2011. Phangorn: phylogenetic analysis in R. Bioinformatics 27: 592–593. doi:10.1093/bioinformatics/btq706 21169378 PMC3035803

[mcag087-B101] Schliep K, Potts AJ, Morrison DA, Grimm GW. 2017. Intertwining phylogenetic trees and networks. Methods in Ecology and Evolution 8: 1212–1220. doi:10.1111/2041-210X.12760

[mcag087-B130] Schumann K . 1894. Bignoniaceae. In: Engler HGA. ed. *Die Natürlichen Pflanzenfamilien* Teil 4 (Abt. 3b). Leipzig: W. Engelmann, 189–252.

[mcag087-B102] Shen XX, Steenwyk JL, Rokas A. 2021. Dissecting incongruence between concatenation-and quartet-based approaches in phylogenomic data. Systematic Biology 70: 997–1014. doi:10.1093/sysbio/syab011 33616672

[mcag087-B103] Simon MF, Grether R, de Queiroz LP, Skema C, Pennington RT, Hughes CE. 2009. Recent assembly of the Cerrado, a neotropical plant diversity hotspot, by in situ evolution of adaptations to fire. Proceedings of the National Academy of Sciences of the United States of America 106: 20359–20364. doi:10.1073/pnas.0903410106 19918050 PMC2787167

[mcag087-B104] Slater GSC, Birney E. 2005. Automated generation of heuristics for biological sequence comparison. BMC Bioinformatics 6: 1–11. doi:10.1186/1471-2105-6-31 15713233 PMC553969

[mcag087-B105] Sobral-Souza T, Lima-Ribeiro MS, Solferini VN. 2015. Biogeography of Neotropical rainforests: past connections between Amazon and Atlantic Forest detected by ecological niche modeling. Evolutionary Ecology 29: 643–655. doi:10.1007/s10682-015-9780-9

[mcag087-B106] Solís-Lemus C . 2023. Network thinking: novel inference tools and scalability challenges. In: Kubatko L, Knowles LL. eds. Species tree inference: a guide to methods and applications. Princeton: Princeton University Press, 120–147. doi:10.2307/j.ctv2wr4wdf

[mcag087-B107] Solís-Lemus C, Yang M, Ané C. 2016. Inconsistency of species tree methods under gene flow. Systematic Biology 65: 843–851. doi:10.1093/sysbio/syw030 27151419

[mcag087-B108] Soltis PS, Soltis DE. 2009. The role of hybridization in plant speciation. Annual Review of Plant Biology 60: 561–588. doi:10.1146/annurev.arplant.043008.092039 19575590

[mcag087-B109] Steenwyk JL, Buida TJ III, Li Y, Shen XX, Rokas A. 2020. ClipKIT: a multiple sequence alignment trimming software for accurate phylogenomic inference. PLoS Biology 18:e3001007. doi:10.1371/journal.pbio.3001007 33264284 PMC7735675

[mcag087-B110] Stull GW, Pham KK, Soltis PS, Soltis DE. 2023. Deep reticulation: the long legacy of hybridization in vascular plant evolution. The Plant Journal 114: 743–766. doi:10.1111/tpj.16142 36775995

[mcag087-B111] Sun JX, Fonseca LHM, Chatrou LW, Lohmann LG. 2025. Molecular phylogenetics and historical biogeography of *Mansoa* DC. (Bignonieae, Bignoniaceae). Botanical Journal of the Linnean Societyboaf076. doi:10.1093/botlinnean/boaf076

[mcag087-B112] Suvorov A, Scornavacca C, Fujimoto MS, et al 2022. Deep ancestral introgression shapes evolutionary history of dragonflies and damselflies. Systematic Biology 71: 526–546. doi:10.1093/sysbio/syab063 34324671 PMC9017697

[mcag087-B113] Thode VA, Sanmartín I, Lohmann LG. 2019. Contrasting patterns of diversification between Amazonian and Atlantic Forest clades of Neotropical lianas (*Amphilophium*, Bignonieae) inferred from plastid genomic data. Molecular Phylogenetics and Evolution 133: 92–106. doi:10.1016/j.ympev.2018.12.021 30584919

[mcag087-B114] Tribble CM, Freyman WA, Landis MJ, et al 2022. RevGadgets: an R package for visualizing Bayesian phylogenetic analyses from RevBayes. Methods in Ecology and Evolution 13: 314–323. doi:10.1111/2041-210X.13750

[mcag087-B115] Turchetto-Zolet AC, Salgueiro F, Turchetto C, et al 2016. Phylogeography and ecological niche modelling in *Eugenia uniflora* (Myrtaceae) suggest distinct vegetational responses to climate change between the southern and the northern Atlantic Forest. Botanical Journal of the Linnean Society 182: 670–688. doi:10.1111/boj.1247

[mcag087-B116] Villaverde T, Larridon I, Shah T, et al 2023. Phylogenomics sheds new light on the drivers behind a long-lasting systematic riddle: the figwort family Scrophulariaceae. New Phytologist 240: 1601–1615. doi:10.1111/nph.18845 36869601

[mcag087-B117] Wang X, Auler AS, Edwards RL, et al 2004. Wet periods in northeastern Brazil over the past 210 Kyr linked to distant climate anomalities. Nature 432: 740–743. doi:10.1038/nature03067 15592409

[mcag087-B118] Werneck FP . 2011. The diversification of eastern South American open vegetation biomes: historical biogeography and perspectives. Quaternary Science Reviews 30: 1630–1648. doi:10.1016/j.quascirev.2011.03.009

[mcag087-B119] Westerhold T, Marwan N, Drury AJ, et al 2020. An astronomically dated record of Earth’s climate and its predictability over the last 66 million years. Science 369: 1383–1387. doi:10.1126/science.aba6853 32913105

[mcag087-B120] Wing SL, Herrera F, Jaramillo CA, Gómez-Navarro C, Wilf P, Labandeira CC. 2009. Late Paleocene fossils from the Cerejón Formation, Colombia, are the earliest record of Neotropical rainforest. Proceedings of the National Academy of Sciences of the United States of America 106: 18627–18632. doi:10.1073/pnas.0905130106 19833876 PMC2762419

[mcag087-B121] Xu Y, Wei Y, Zhou Z, et al 2024. Widespread incomplete lineage sorting and introgression shaped adaptive radiation in the *Gossypium* genus. Plant Communications 5:100728. doi:10.1016/j.xplc.2023.100728 37803827 PMC10873890

[mcag087-B122] Yang Z . 2007. PAML 4: phylogenetic analysis by maximum likelihood. Molecular Biology and Evolution 24: 1586–1591. doi:10.1093/molbev/msm088 17483113

[mcag087-B123] Yang F, Ge J, Guo Y, Olmstead R, Sun W. 2023. Deciphering complex reticulate evolution of Asian *Buddleja* (Scrophulariaceae): insights into the taxonomy and speciation of polyploid taxa in the Sino-Himalayan region. Annals of Botany 132: 15–28. doi:10.1093/aob/mcad022 36722368 PMC10550280

[mcag087-B124] Yu Y, Nakhleh L. 2015. A maximum pseudo-likelihood approach for phylogenetic networks. BMC Genomics 16:S10. doi:10.1186/1471-2164-16-S10-S10 PMC4602316 26450642

[mcag087-B125] Yu Y, Than C, Degnan JH, Nakhleh L. 2011. Coalescent histories on phylogenetic networks and detection of hybridization despite incomplete lineage sorting. Systematic Biology 60: 138–149. doi:10.1093/sysbio/syq084 21248369 PMC3167682

[mcag087-B126] Zhang C, Mirarab S. 2022. Weighting by gene tree uncertainty improves accuracy of quartet-based species trees. Molecular Biology and Evolution 39:msac215. doi:10.1093/molbev/msac215 36201617 PMC9750496

[mcag087-B127] Zhang C, Nielsen R, Mirarab S. 2025. CASTER: direct species tree inference from whole-genome alignments. Science 387:eadk9688. doi:10.1126/science.adk9688 39847611 PMC12038793

[mcag087-B128] Zhou BF, Yuan S, Crowl AA, et al 2022. Phylogenomic analyses highlight innovation and introgression in the continental radiations of Fagaceae across the Northern Hemisphere. Nature Communications 13: 1320. doi:10.1038/s41467-022-28917-1 PMC8921187 35288565

