# Supplementary material for: Atlantic forest origins and Amazon connections: the evolutionary history of Adenocalymma
Source: Ann Bot. 2026 Apr 13;138(2):488–504. doi: 10.1093/aob/mcag087 (PMC13454680; doi:10.1093/aob/mcag087)
Supplement: Supplementary file 1 [file mcag087_supplementary_data.zip › supplemental1.pdf]

Table S1 Collection information of the specimens. Sample sources are also mentioned, including the herbarium where the collection is housed.

Species	Source	Collector	Number	Country	Major area
A. ackermanii	Silica gel (SPF)	L.H.M. Fonseca	272	Brazil	Minas Gerais
A. adenophorum	Silica gel (MO)	L.G. Lohmann	30	Brazil	Amazonas
A. albiflorum	Silica gel (SPF)	Matos	1733	Brazil	Bahia
A. alboaurantiacum	Silica gel (MO)	L.G. Lohmann	681	Brazil	Espírito Santo
A. allamandiflorum	Silica gel (SPF)	B.M. Gomes	671	Brazil	Pará
A. apetiolatum	Silica gel (SPF)	L.H.M. Fonseca	431	Brazil	Espírito Santo
A. apparicianum	Silica gel (SPF)	A. Frazão	166	Brazil	Ceará
A. apurense	Silica gel (SPF)	L.H.M. Fonseca	348	Brazil	Roraima
A. aurantiacum	Silica gel (MO)	L.G. Lohmann	658	Brazil	Espírito Santo
A. biternatum	Herbarium (SPF)	M.R. Pace	521	Peru	Loreto
A. bracteatum	Silica gel (SPF)	L.H.M. Fonseca	100	Brazil	Paraná
A. bracteolatum	Silica gel (SPF)	L.H.M. Fonseca	346	Brazil	Roraima
A. bracteosum	Silica gel (SPF)	A. Frazão	209	Brazil	Pará
A. calcareum	Herbarium (SPF)	D. Sasaki	1776	Brazil	Mato Grosso
A. candolleanum	Silica gel (MO)	L.G. Lohmann	363	Brazil	Bahia
A. cauliflorum	Silica gel (SPF)	L.H.M. Fonseca	448	Brazil	Rio de Janeiro
A. cinereum	Silica gel (SPF)	L.H.M. Fonseca	220	Brazil	Minas Gerais
A. cladotrichum	Silica gel (SPF)	M.R. Pace	517	Peru	Loreto
A. comosum	Silica gel (SPF)	L.H.M. Fonseca	208	Brazil	Rio de Janeiro
A. contractum	Silica gel (SPF)	R.G. Udulutsch	2676	Brazil	Rondônia
A. coriaceum	Silica gel (SPF)	L.H.M. Fonseca	358	Brazil	Bahia
A. cristicalyx	Silica gel (MO)	L.G. Lohmann	705	Brazil	Paraíba
A. cymbalum	Silica gel (SPF)	L.H.M. Fonseca	424	Brazil	Espírito Santo
A. darwinii	Herbarium (SPF)	L. Kollmann	8228	Brazil	Espírito Santo
A. dichilum	Silica gel (SPF)	L.H.M. Fonseca	386	Brazil	Bahia
A. divaricatum	Silica gel (SPF)	L.H.M. Fonseca	281	Brazil	Bahia
A. dusenii	Herbarium (SPF)	N.M. Ivanauskas	726	Brazil	São Paulo
A. fistulosum	Herbarium (SPF)	G.S. França	341	Brazil	Minas Gerais
A. flaviflorum1	Herbarium (CEN)	M.F. Simon	1262	Brazil	Rondônia
A. flaviflorum2	Silica gel (SPF)	A. Nogueira	165	Brazil	Amazonas
A. flavum	Herbarium (SPF)	J.L. Paixão	1394	Brazil	Bahia
A. gibbosum	Silica gel (SPF)	L.H.M. Fonseca	222	Brazil	Minas Gerais
A. gracielzae	Silica gel (SPF)	L.H.M. Fonseca	409	Brazil	Para
A. grandifolium	Silica gel (SPF)	L.H.M. Fonseca	211	Brazil	Rio de Janeiro
A. hatschbachii	Silica gel (SPF)	L.H.M. Fonseca	229	Brazil	Paraná
A. hypostictum	Silica gel (SPF)	L.H.M. Fonseca	293	Brazil	Bahia
A. impressum	Silica gel (SPF)	A. Frazão	315	Brazil	Amazonas
A. juliae	Herbarium (MO)	A. Gentry	20354	Peru	Loreto
A. lineare	Silica gel (SPF)	L.H.M. Fonseca	409	Brazil	Para
A. longilineum	Silica gel (SPF)	L.H.M. Fonseca	325	Brazil	Amazonas
A. macrophyllum	Silica gel (SPF)	L.H.M. Fonseca	420	Brazil	Espírito Santo
A. magnificum	Silica gel (SPF)	L.H.M. Fonseca	392	Brazil	Pará
A. marginatum	Silica gel (SPF)	L.H.M. Fonseca	108	Brazil	Paraná
A. mirabile1	Herbarium (MO)	A. Fernandes	6056	Brazil	Maranhão
A. mirabile2	Herbarium (MO)	A. Fernandes	8439	Brazil	Maranhão
A. moringifolium	Silica gel (SPF)	L.H.M. Fonseca	329	Brazil	Amazonas
A. nervosum	Silica gel (SPF)	L.H.M. Fonseca	262	Brazil	Espírito Santo
A. nodosum	Silica gel (MO)	L.G. Lohmann	266	Brazil	Minas Gerais
A. patulum	Herbarium (MO)	A. Gentry	78473	Colombia	Bolivar

A. paulistarum	Silica gel (SPF)	L.H.M. Fonseca	355	Brazil	São Paulo
A. pedunculatum	Silica gel (SPF)	L.H.M. Fonseca	267	Brazil	Minas Gerais
A. peregrinum	Silica gel (SPF)	L.H.M. Fonseca	444	Brazil	Goiás
A. pubescens	Silica gel (SPF)	M. Kaehler	194	Brazil	Piauí
A. racemosum	Herbarium (MO)	B.G. Bordenave	8007	Suriname	Sipaliwini
A. salmoneum	Herbarium (SPF)	L. Kollmann	8141	Brazil	Espírito Santo
A. saulense1	Herbarium (CAY)	R. Jansen-Jacobs	1820	French Guiana	NA
A. saulense2	Silica gel (SPF)	A. Frazão	322	Brazil	Amazonas
A. scabriusculum	Silica gel (SPF)	M. Kaehler	186	Brazil	Ceará
A. schomburgkii1	Silica gel (SPF)	A. Nogueira	353	Brazil	Roraima
A. schomburgkii2	Silica gel (SPF)	A. Frazão	256	Brazil	Roraima
A. sessile	Herbarium (VIC)	V.V. Scudeller	536	Brazil	Minas Gerais
A. subincanum	Silica gel (SPF)	A. Frazão	319	Brazil	Amazonas
A. subsessilifolium	Silica gel (SPF)	L.H.M. Fonseca	240	Brazil	Espírito Santo
A. subspicatum	Herbarium (HRCB)	R.G. Udulutsch	2758	Brazil	Ceará
A. tanaeciicarpum	Herbarium (SPF)	A. Nogueira	353	Brazil	Amazonas
A. tephrinocalyx	Silica gel (SPF)	L.H.M. Fonseca	212	Brazil	Espírito Santo
A. trifoliatum	Silica gel (SPF)	L.H.M. Fonseca	413	Brazil	Rio de Janeiro
A. ubatubense	Herbarium (RFFP)	D. Machado	373	Brazil	Rio de Janeiro
A. uleanum	Herbarium (SPF)	G.A. Parada	954	Bolivia	Santa Cruz
A. validum	Silica gel (SPF)	A. Frazão	179	Brazil	Ceará
A. velutinum	Silica gel (SPF)	L.H.M. Fonseca	412	Brazil	Pará

Table S2 Summary statistics of sequencing success including the number of raw pair-end reads obtained, percentage of on-target reads, number of loci obtained, percentage of gene recovery, and number of paralogs.

Species	Total reads	Reads on target	Total loci	Paralogs
Adenocalymma ackermanii	18567746	77.5	729	53
Adenocalymma acutissimum	20818204	72.8	727	46
Adenocalymma adenophorum	23042624	77.7	722	43
Adenocalymma albiflorum	8893416	83.3	731	37
Adenocalymma alboaurantiacum	29301422	47.7	719	29
Adenocalymma allamandiflorum	20050530	75.9	729	61
Adenocalymma apetiolatum	23958638	79.8	731	52
Adenocalymma apparicianum	45042376	77.7	734	27
Adenocalymma apurense	18729252	73.4	726	46
Adenocalymma aurantiacum	11510466	82.2	728	37
Adenocalymma biternatum	14602906	75	722	41
Adenocalymma bracteatum	16343474	75.7	723	41
Adenocalymma bracteolatum	18940506	68.8	729	43
Adenocalymma bracteosum	10283470	80	725	38
Adenocalymma calcareum	22887096	71.8	731	44
Adenocalymma candolleanum	20502340	84.7	729	38
Adenocalymma cauliflorum	27028076	71.1	728	41
Adenocalymma cinereum	19470100	73.9	733	40
Adenocalymma cladotrichum	12408684	76	729	37
Adenocalymma comosum	35803182	78.2	734	42
Adenocalymma contractum	13600756	80.5	725	51
Adenocalymma coriaceum	28600664	79	733	54
Adenocalymma cristicalyx	21568574	79.9	724	35
Adenocalymma cymbalum	8070196	78	724	46
Adenocalymma darwinii	15750868	84.3	728	30
Adenocalymma dichilum	25923422	82.2	728	48
Adenocalymma divaricatum	11216850	78.6	728	51
Adenocalymma dusenii	22961332	71.2	726	27
Adenocalymma fistulosum	21972812	84.8	729	37
Adenocalymma flaviflorum1	25702146	77.6	726	44
Adenocalymma flaviflorum2	16953728	80	728	50
Adenocalymma flavum	36732462	85	731	38
Adenocalymma gibbosum	13286102	82.2	725	37
Adenocalymma gracielzae	16652742	81.4	726	47
Adenocalymma grandifolium	15801222	70.2	729	52
Adenocalymma hatschbachii	21958856	77.9	731	41
Adenocalymma hypostictum	22686188	79	728	48
Adenocalymma impressum	14298788	79	728	52
Adenocalymma juliae	8140496	84.9	725	33
Adenocalymma lineare	19538214	82.3	729	50
Adenocalymma longilineum	12805978	83.3	721	56
Adenocalymma macrophyllum	23602940	76.4	734	51
Adenocalymma magnificum	12144532	80.6	724	47
Adenocalymma marginatum	17418670	79.3	725	46
Adenocalymma mirabile1	17396084	87.1	731	34
Adenocalymma mirabile2	27039902	90.1	728	25
Adenocalymma moringifolium	8250838	79.9	720	42
Adenocalymma nervosum	32526560	76.4	734	53
Adenocalymma nodosum	32413318	66.4	727	54

Adenocalymma patulum	6060152	81.3	696	16
Adenocalymma paulistarum	17475052	74.9	729	43
Adenocalymma pedunculatum	22098912	81.3	728	42
Adenocalymma peregrinum	17795178	71.9	727	47
Adenocalymma pubescens	17032954	85.8	723	38
Adenocalymma racemosum	15690814	84.1	722	44
Adenocalymma salmoneum	12302434	80.2	732	40
Adenocalymma saulense1	19752008	87.3	723	33
Adenocalymma saulense2	17563780	81.4	729	48
Adenocalymma scabriusculum	26146676	81.4	727	46
Adenocalymma schomburgkii1	10372152	81.8	724	42
Adenocalymma schomburgkii2	25852722	84.1	731	39
Adenocalymma sessile	25916284	86.2	729	41
Adenocalymma subincanum	22414872	77.8	729	52
Adenocalymma subsessilifolium	52197438	80.1	733	35
Adenocalymma subspicatum	25370904	79.6	723	53
Adenocalymma tanaeciicarpum	22121254	82.1	737	50
Adenocalymma tephrinocalyx	26235832	79.3	737	49
Adenocalymma trifoliatum	31203826	79.4	732	40
Adenocalymma ubatubense	10734390	82.2	727	40
Adenocalymma uleanum	10783088	80.5	723	40
Adenocalymma validum	26158286	80.6	727	46
Adenocalymma velutinum	17371126	77.6	730	52
Anemopaegma arvense	16567304	75.4	716	34
Callichlamys latifolia	21619446	82.2	726	46
Crescentia cujete	13228134	83.3	736	35
Dolichandra cynanchoides	22126318	73.4	732	50
Fridericia speciosa	7854474	79.6	717	41
Jacaranda mimosifolia	9892098	73.6	615	51
Perianthomega vellozoi	11365240	85.5	732	37
Tanaecium jaroba	45068434	78.7	725	49

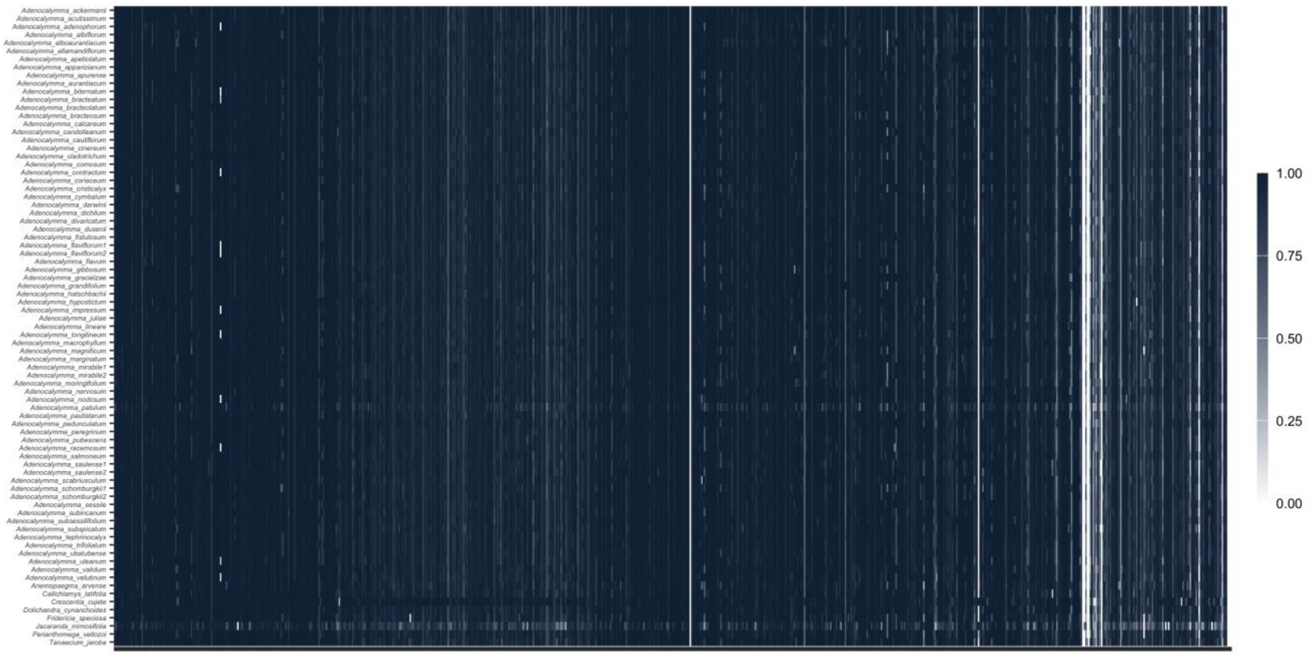

FIG. S1. Recovered sequence heatmap for all the 762 genes targeted. Each row corresponds to a different specimen sampled, and each column correspond to a gene. Colors represent the length of the recovered sequence relative to the template sequence.

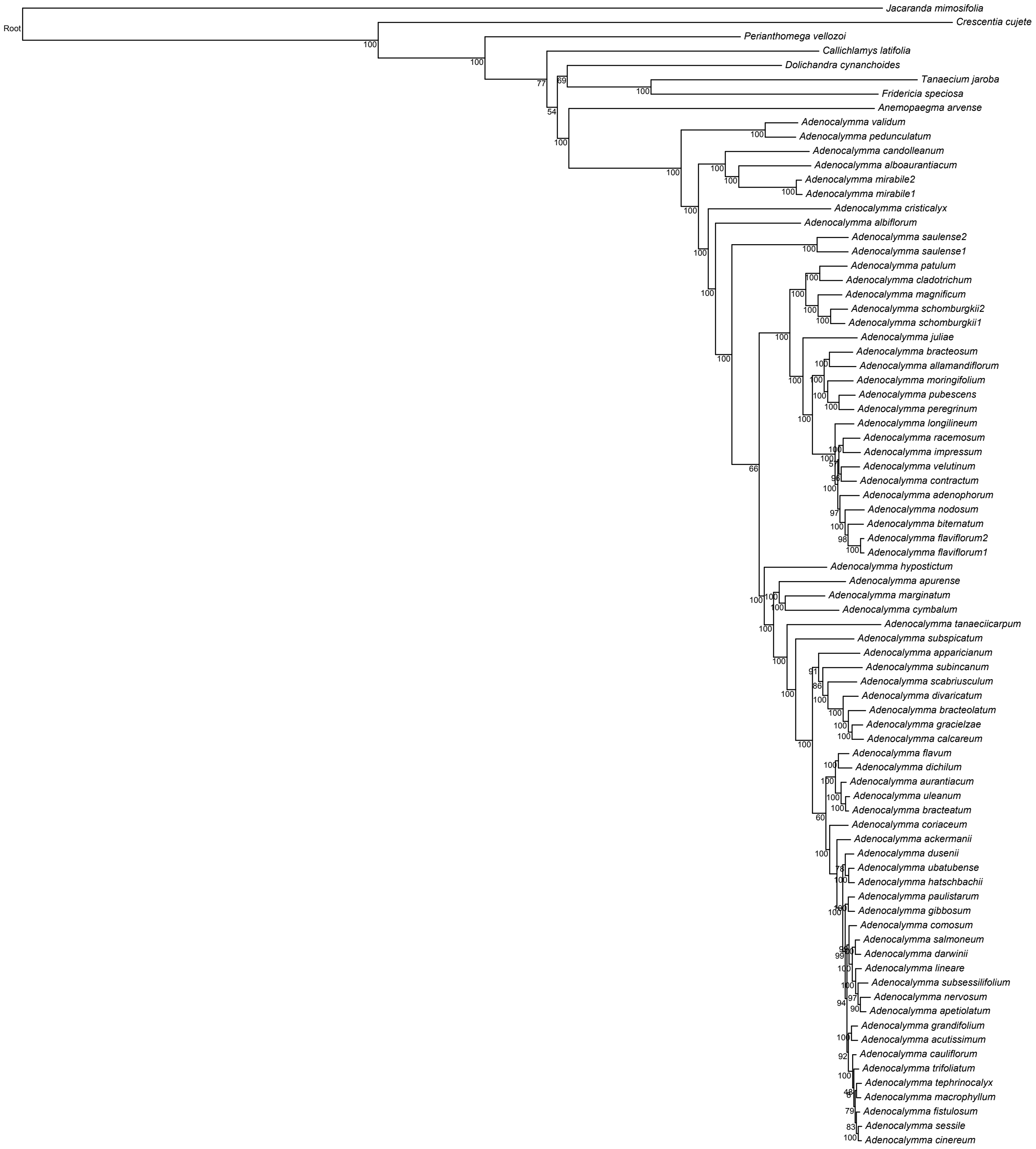

FIG. S2. Phylogenetic tree from the concatenated approach of Exon-Only dataset. The tree was inferred using IQ-TREE. Branch support are results of the UFBoot sampling.

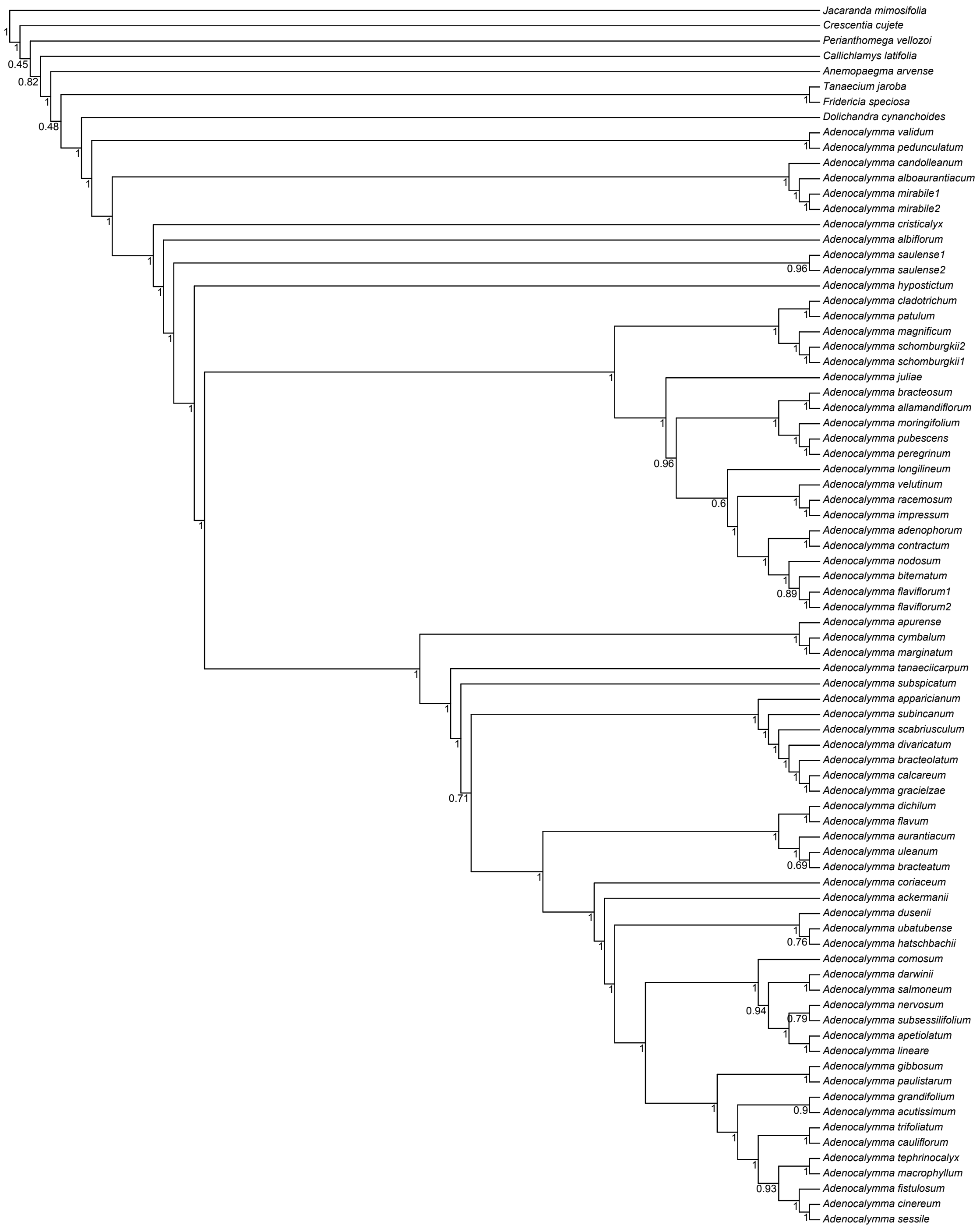

FIG. S3. Species-tree from analysis of the 661 independent trees from the Exon-Only dataset. The tree was inferred using weighted ASTRAL. Branch support are Local Posterior Probabilities.

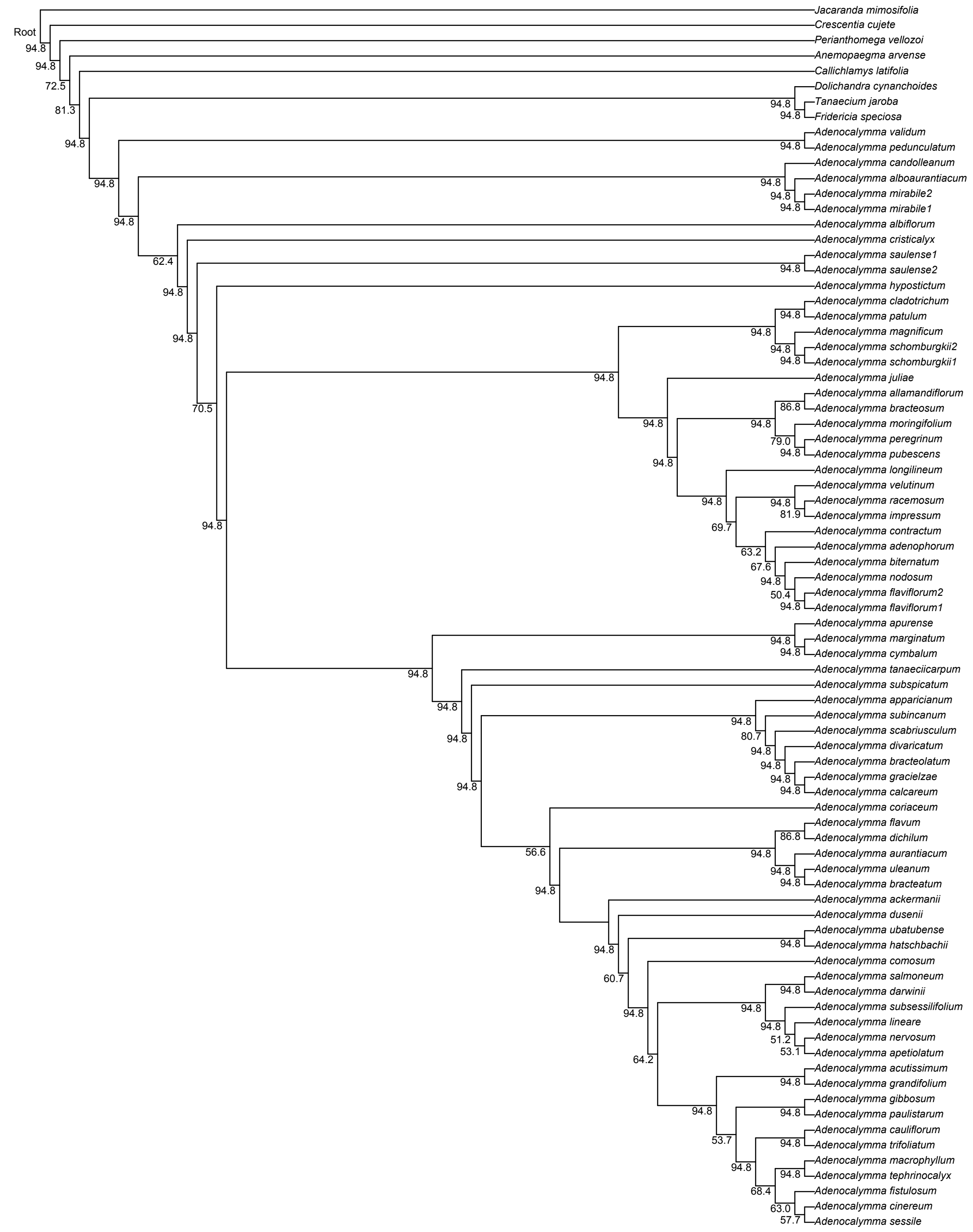

FIG. S4. Species-tree from analysis of the combined matrix of the Exon-Only dataset. The tree was inferred using CASTER-site. Branch support are Local Bootstraps.

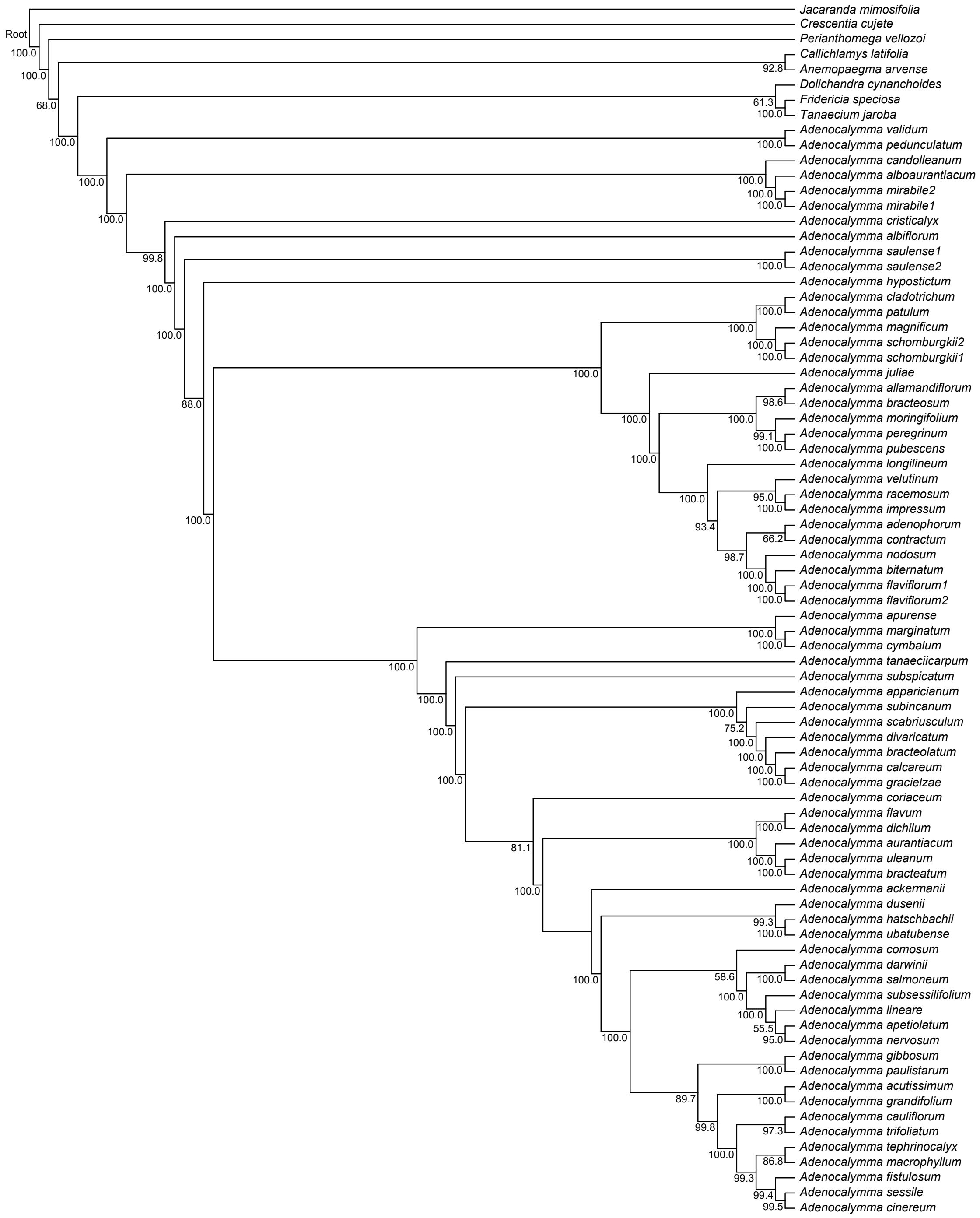

FIG. S5. Species-tree from analysis of the combined matrix of the Supercontig dataset. The tree was inferred using CASTER-site. Branch support are Local Bootstraps.

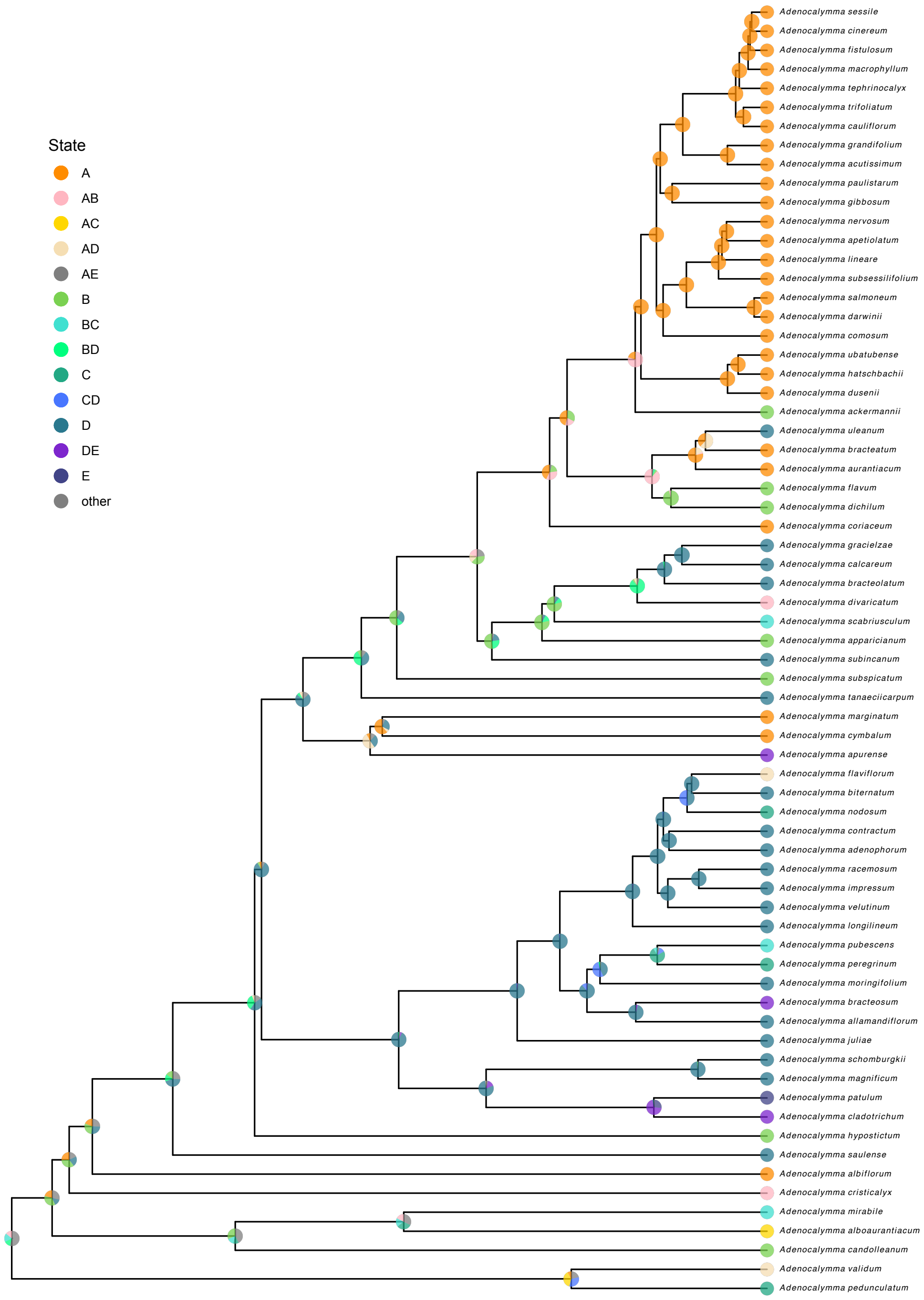

FIG. S6. Bayesian ancestral ranges inferred using RevBayes and the wASTRAL tree from the Supercontig dataset as topological constraint. Results were obtained using a simple dispersal matrix. Colored circles at tips indicate the geographical range of each extant species. Pie charts over nodes represent the relative probabilities of ancestral ranges inferred

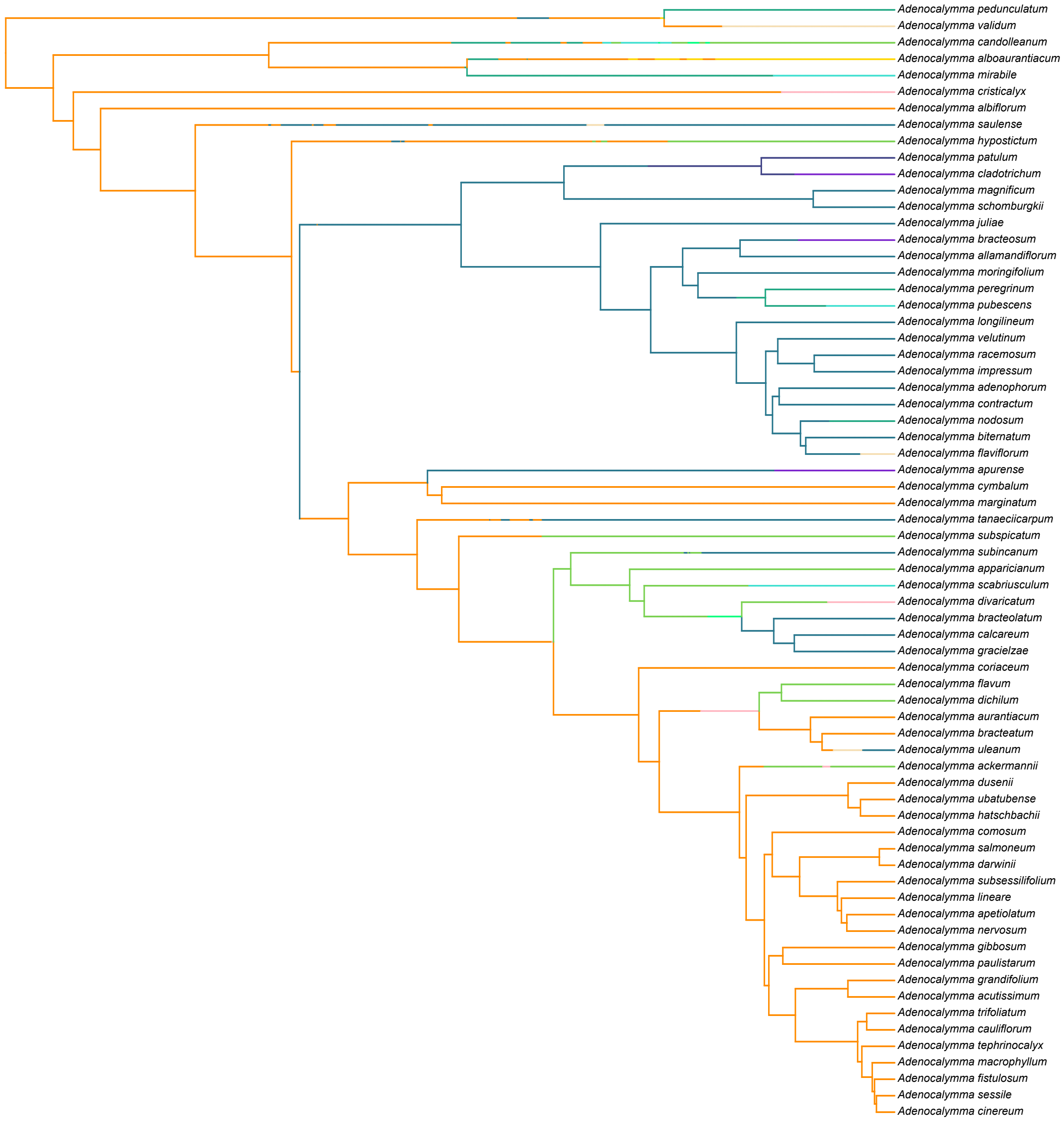

FIG. S7. Bayesian Stochastic Range Mapping inferred using RevBayes and the wASTRAL tree from the Supercontig dataset as topological constraint. Results were obtained using the time stratified dispersal matrices. Colored branches represent the result with major relative probabilities.
