# Supplementary material for: Atlantic forest origins and Amazon connections: the evolutionary history of Adenocalymma
Source: Ann Bot. 2026 Apr 13;138(2):488–504. doi: 10.1093/aob/mcag087 (PMC13454680; doi:10.1093/aob/mcag087)
Supplement: Supplementary file 1 [file mcag087_supplementary_data.zip › Supplemental2.pdf]

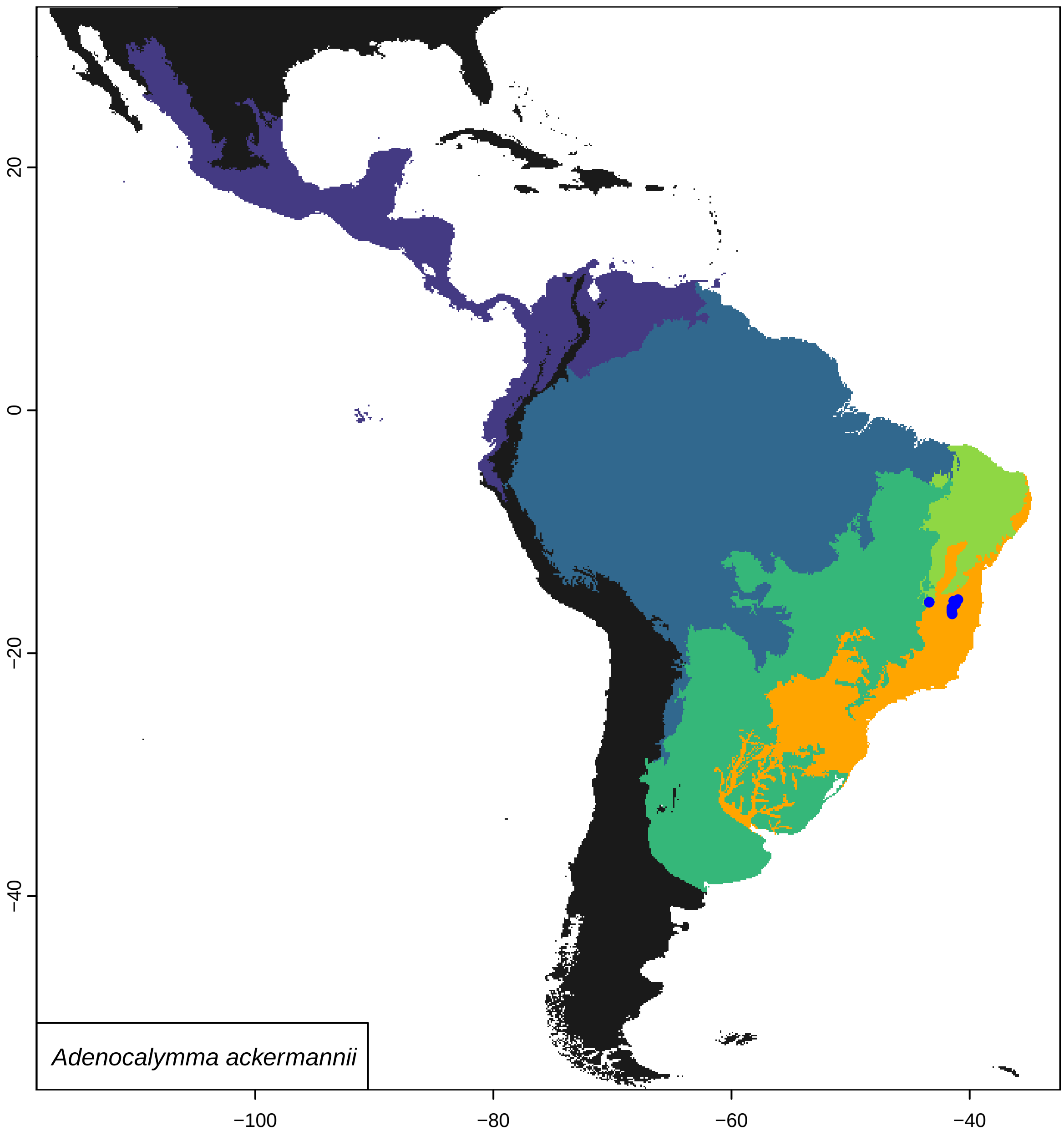
20
0
−20
−40
−100
−80
−60
−40
Adenocalymma ackermannii

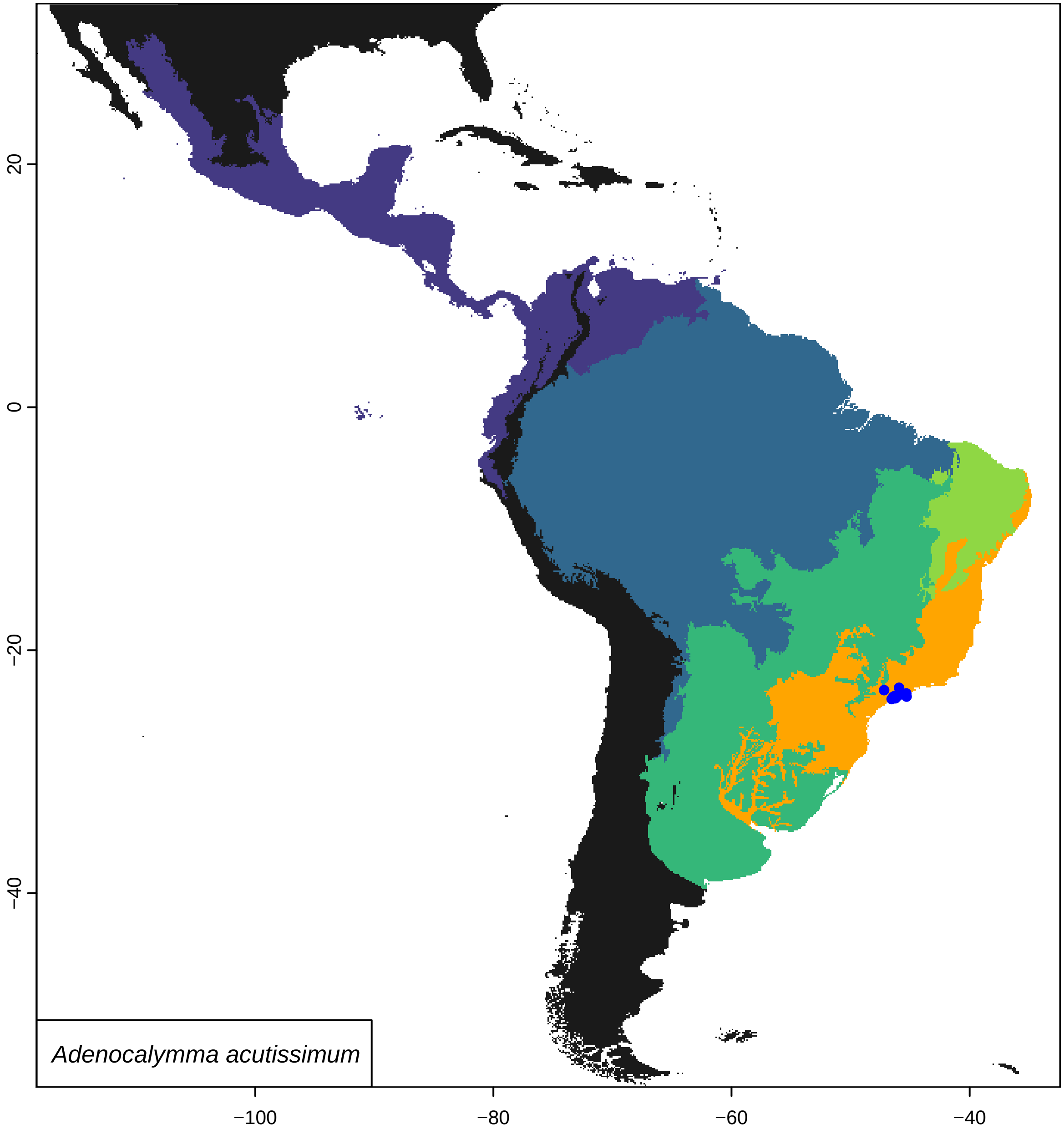

20
0
−20
−40
−100
−80
−60
−40
Adenocalymma acutissimum

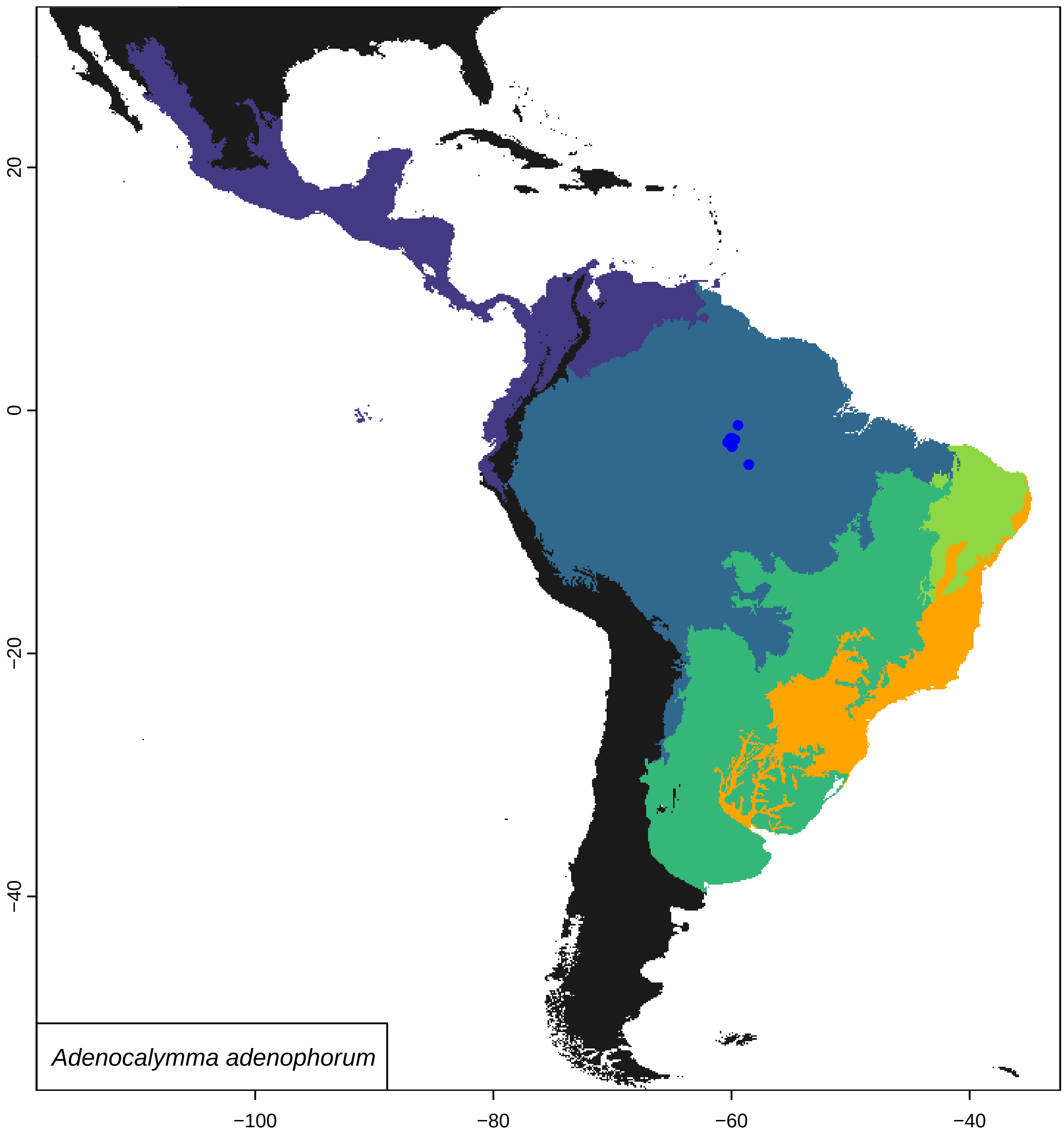

20
0
−20
−40
−100
−80
−60
−40
Adenocalymma adenophorum

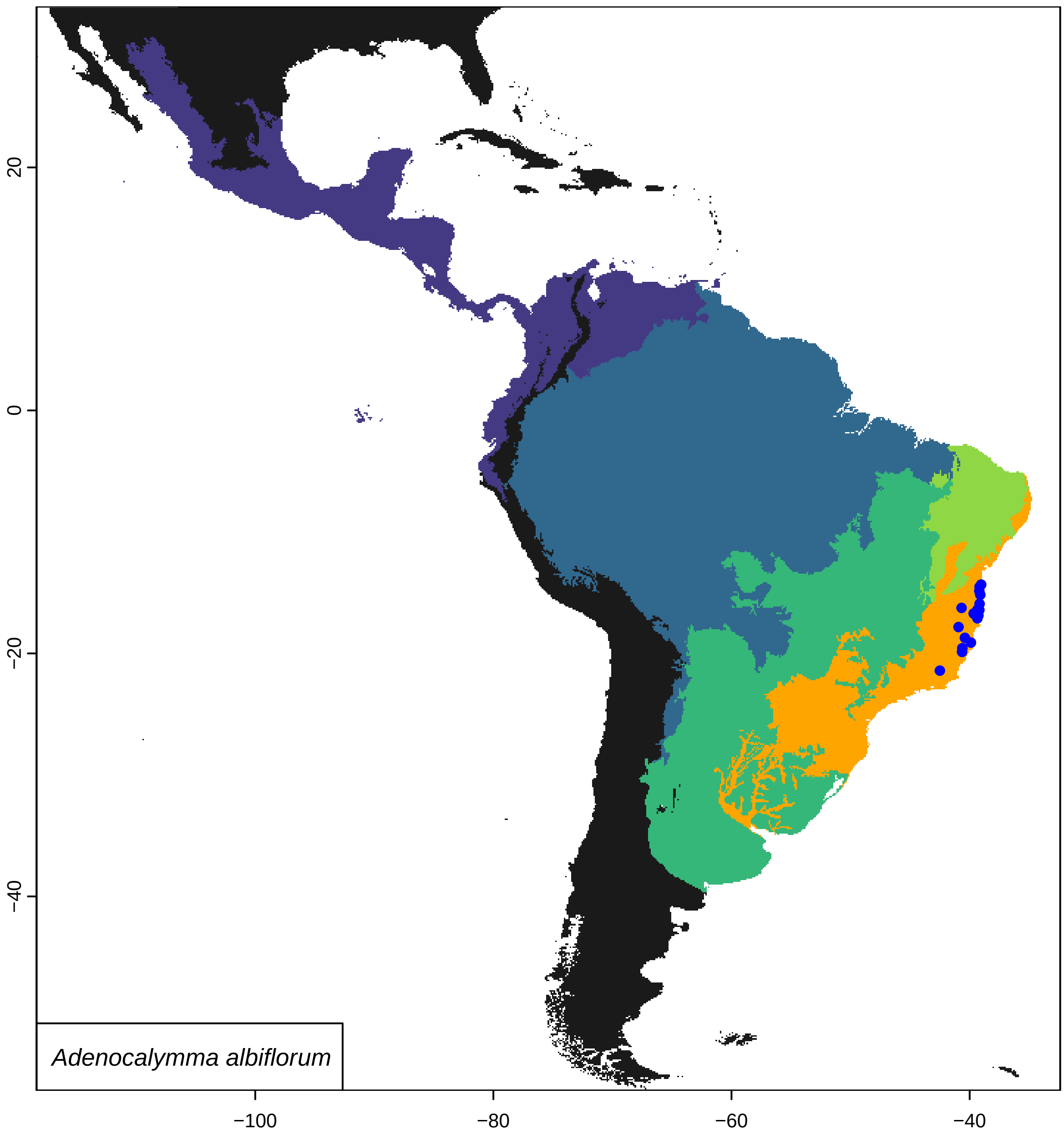
20
0
−20
−40
−100
−80
−60
−40
Adenocalymma albiflorum

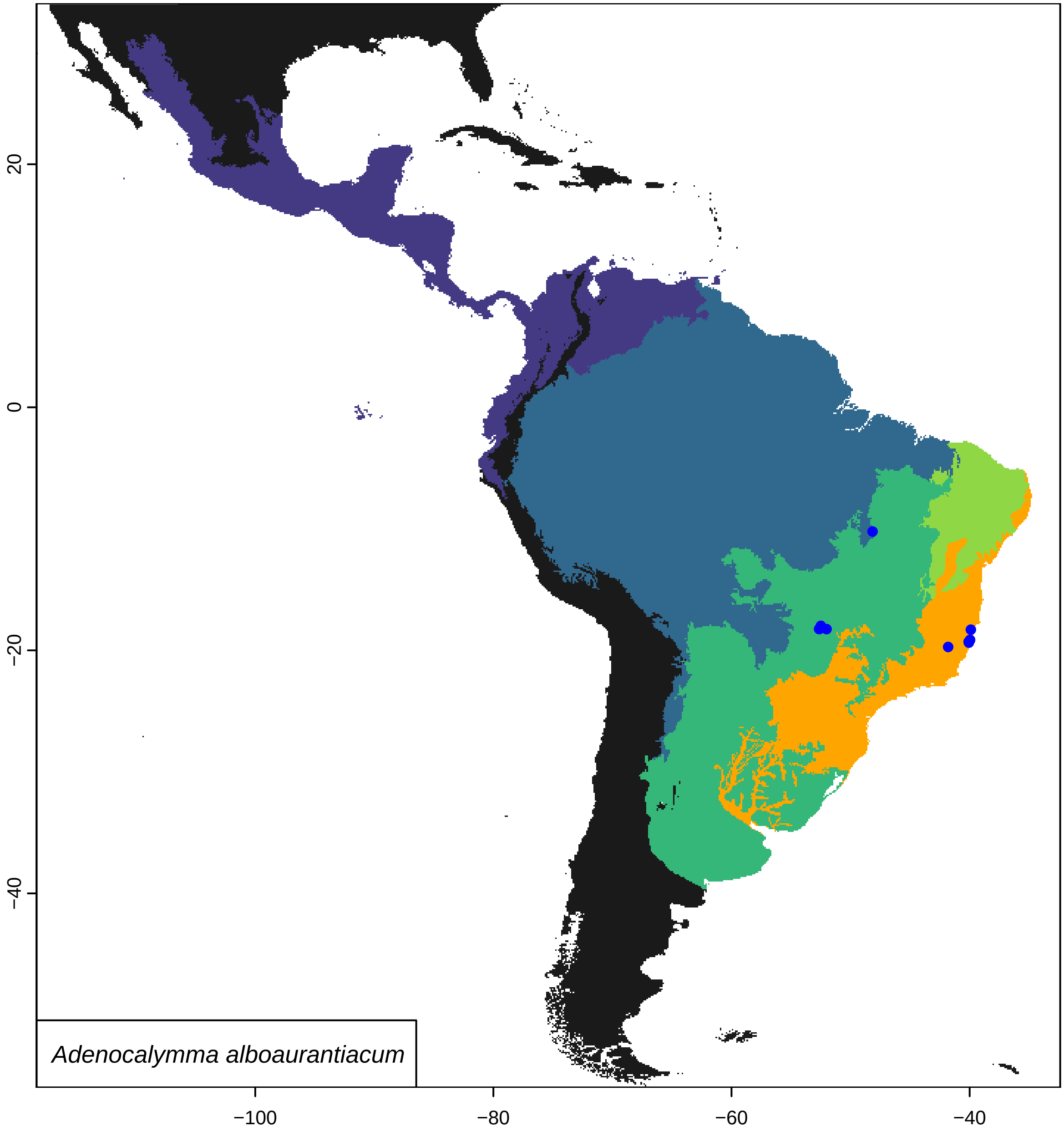

20
0
−20
−40
−100
−80
−60
−40
Adenocalymma alboaurantiacum

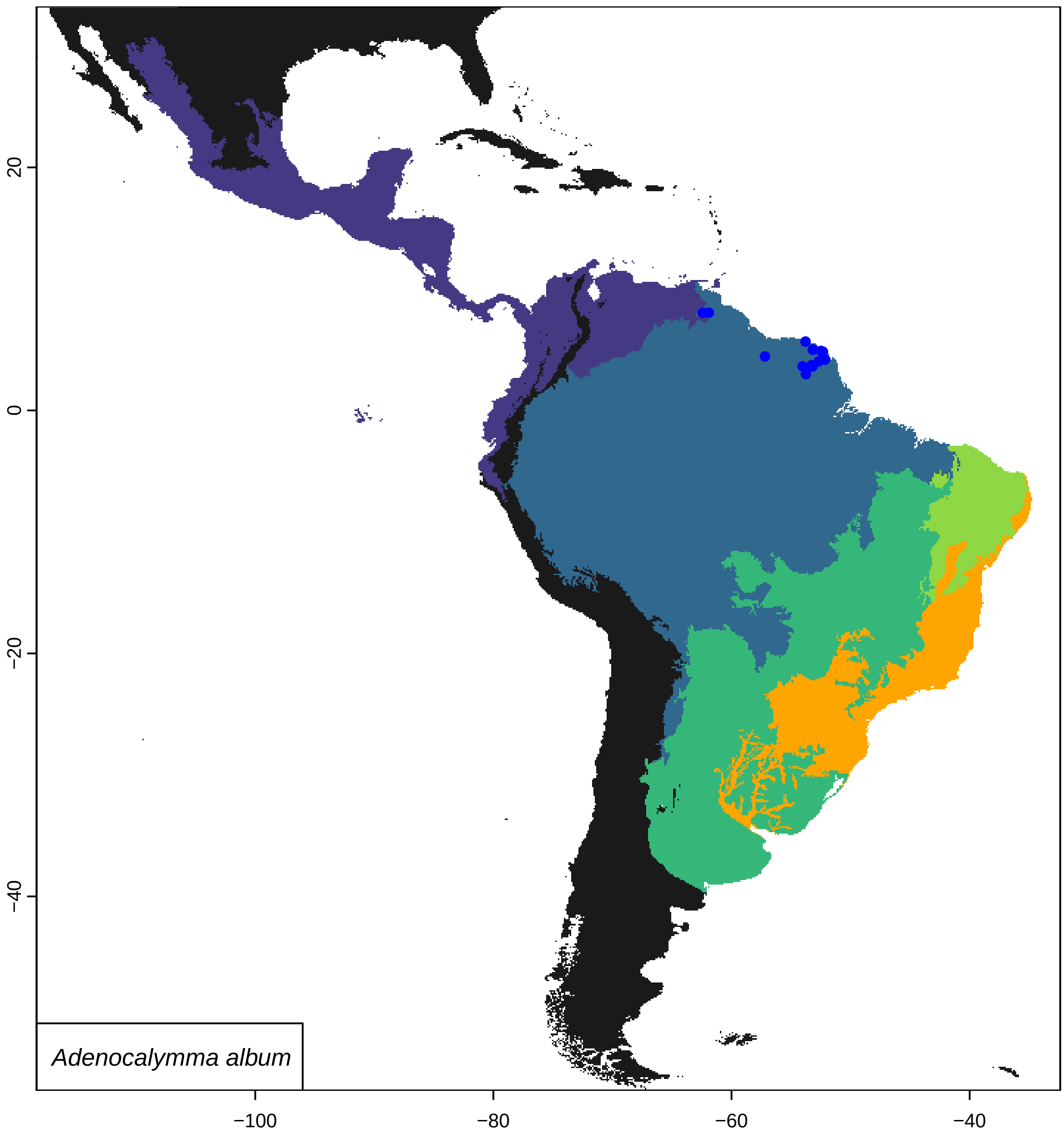

20
0
−20
−40
−100
−80
−60
−40
Adenocalymma album

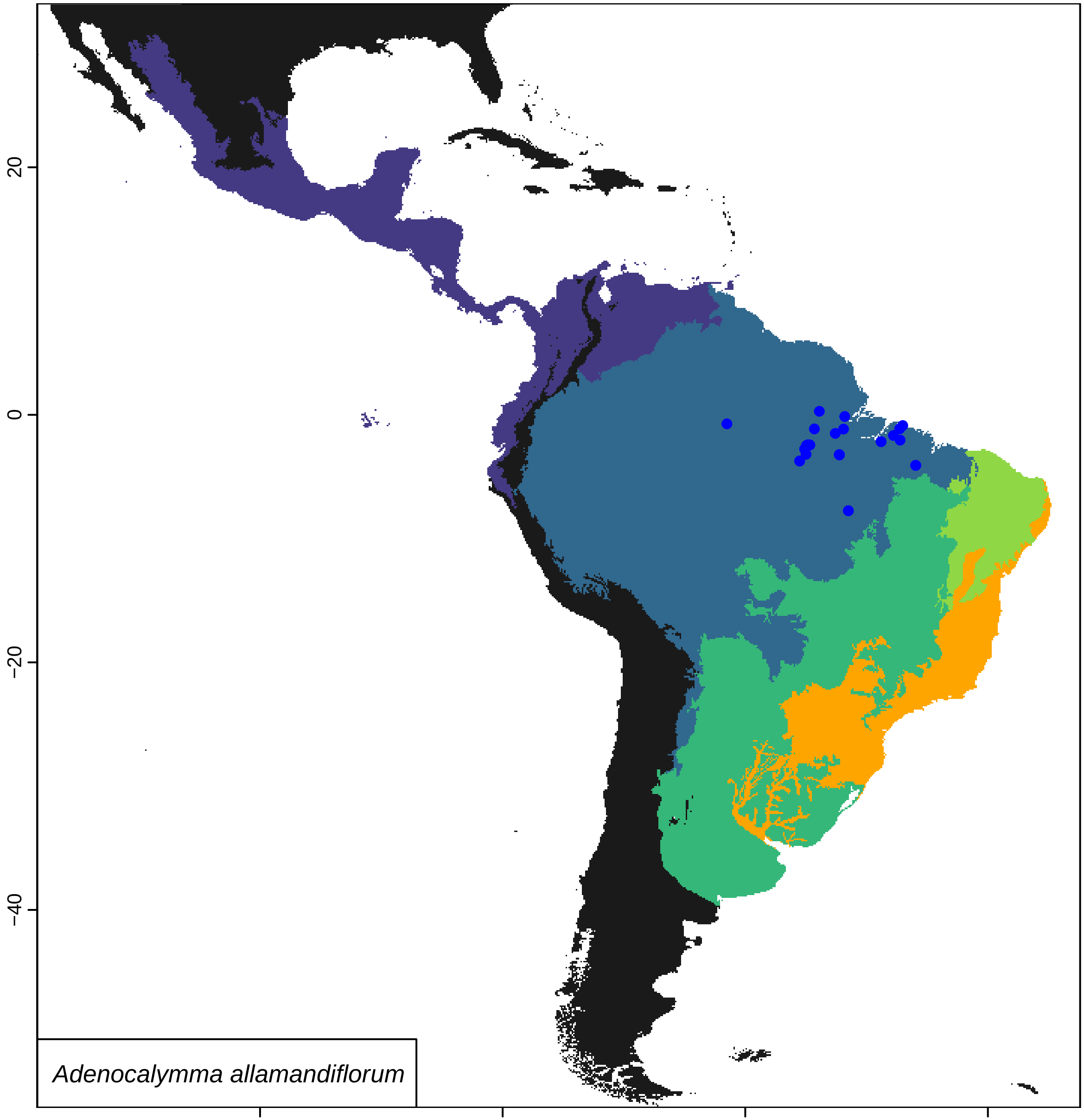

20
0
−20
−40
−100
−80
−60
−40
Adenocalymma allamandiflorum

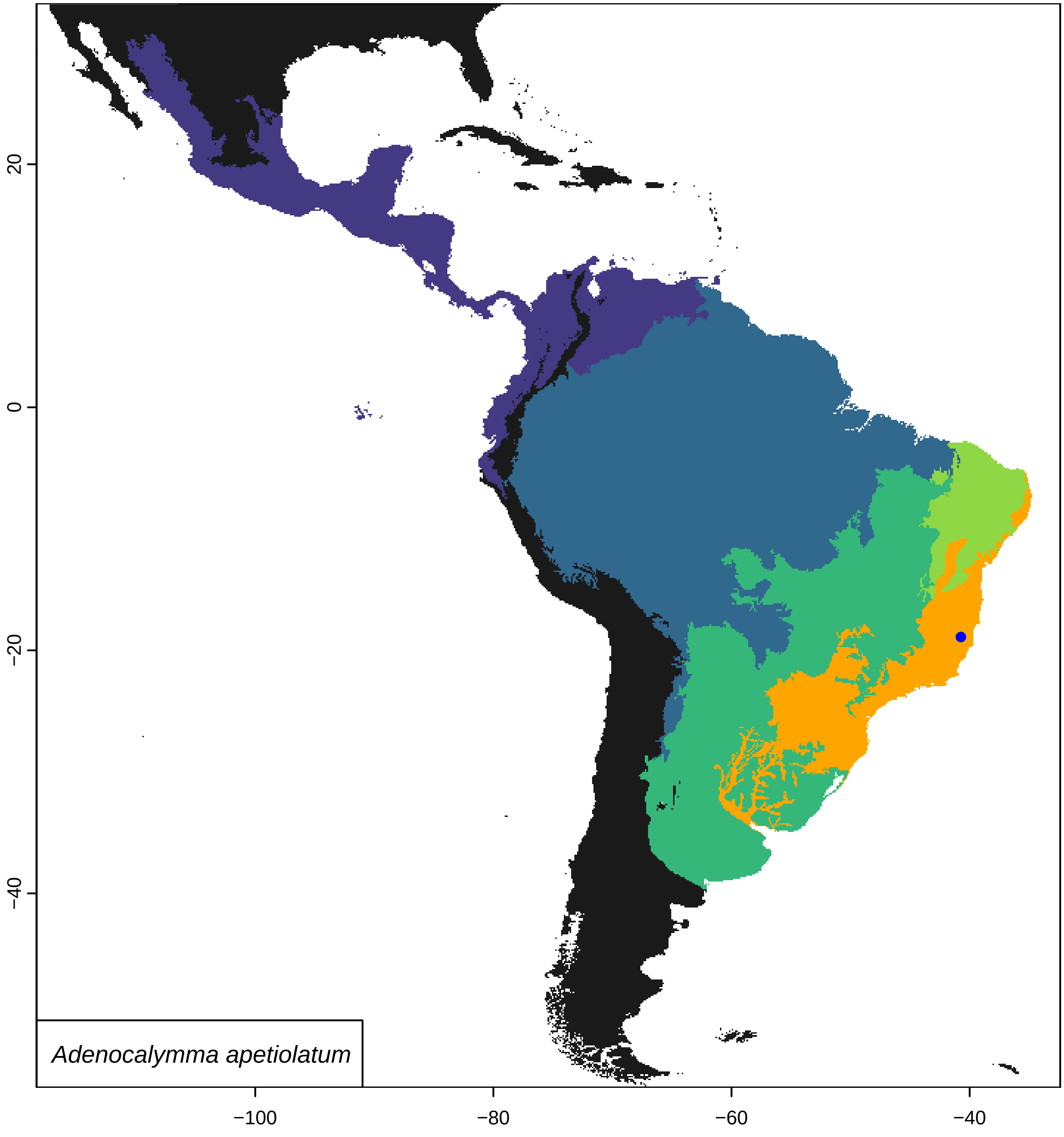
20
0
−20
−40
−100
−80
−60
−40
Adenocalymma apetiolatum

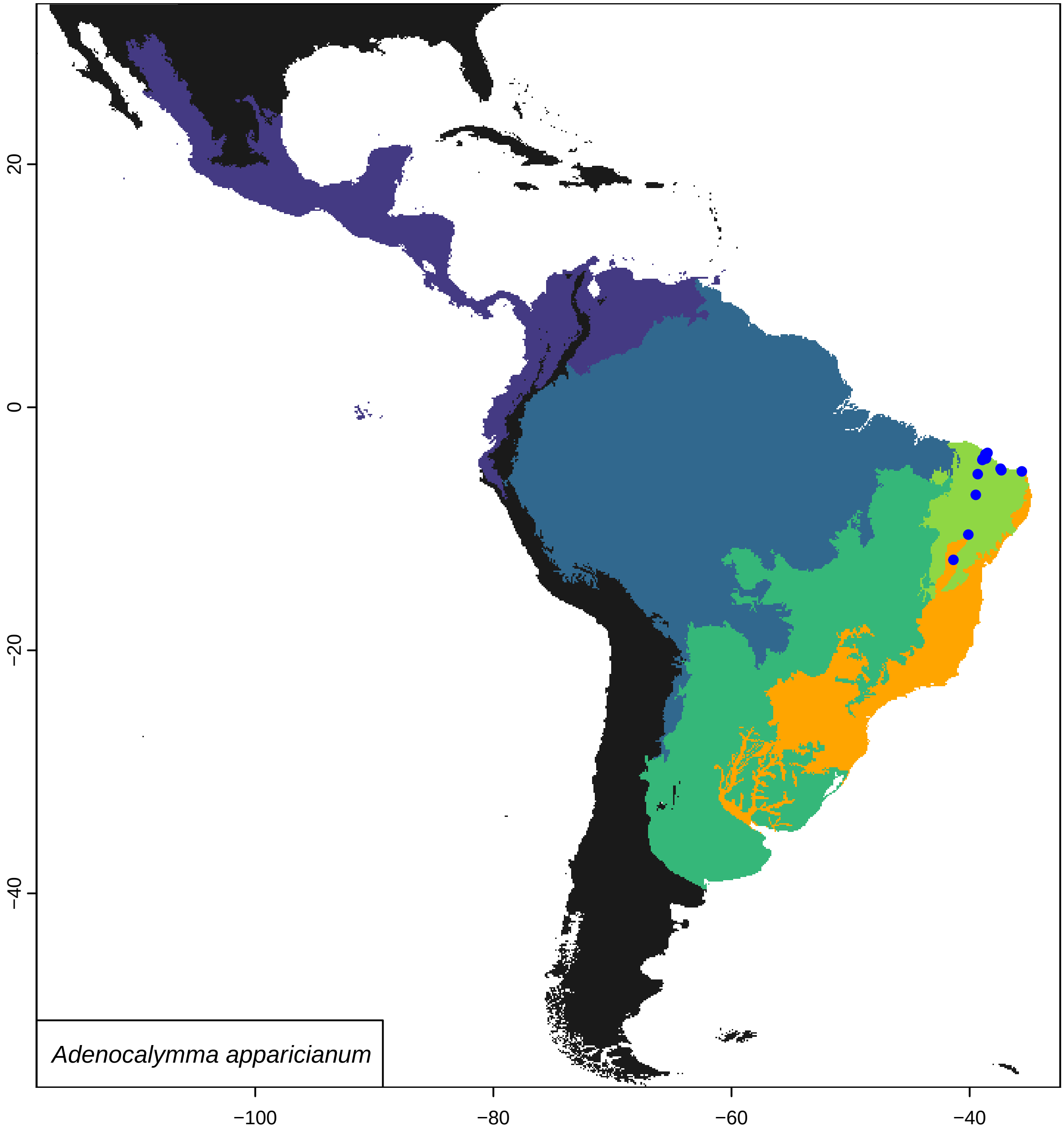

20
0
−20
−40
−100
−80
−60
−40
Adenocalymma apparicianum

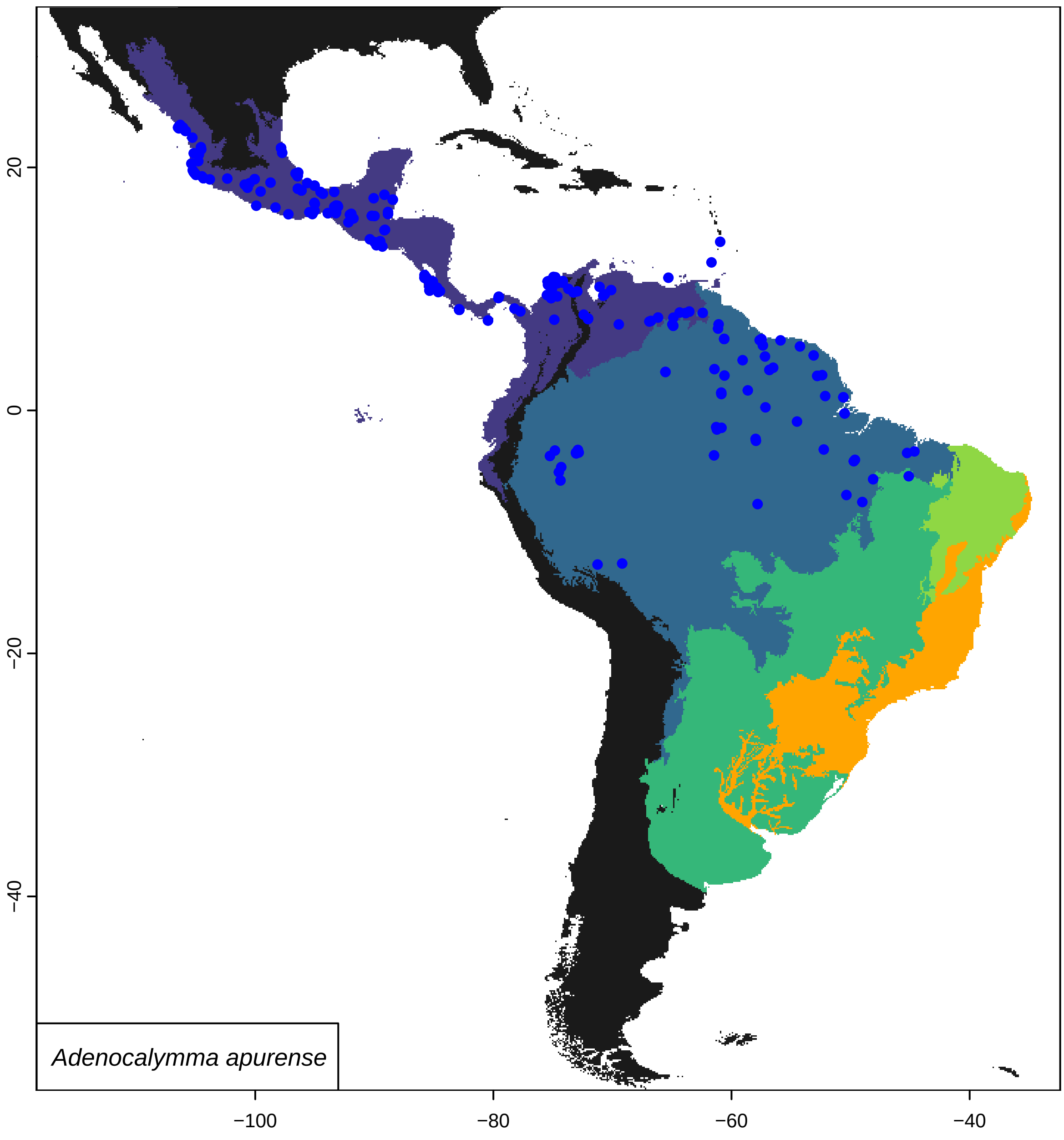

20
0
−20
−40
−100
−80
−60
−40
Adenocalymma apurense

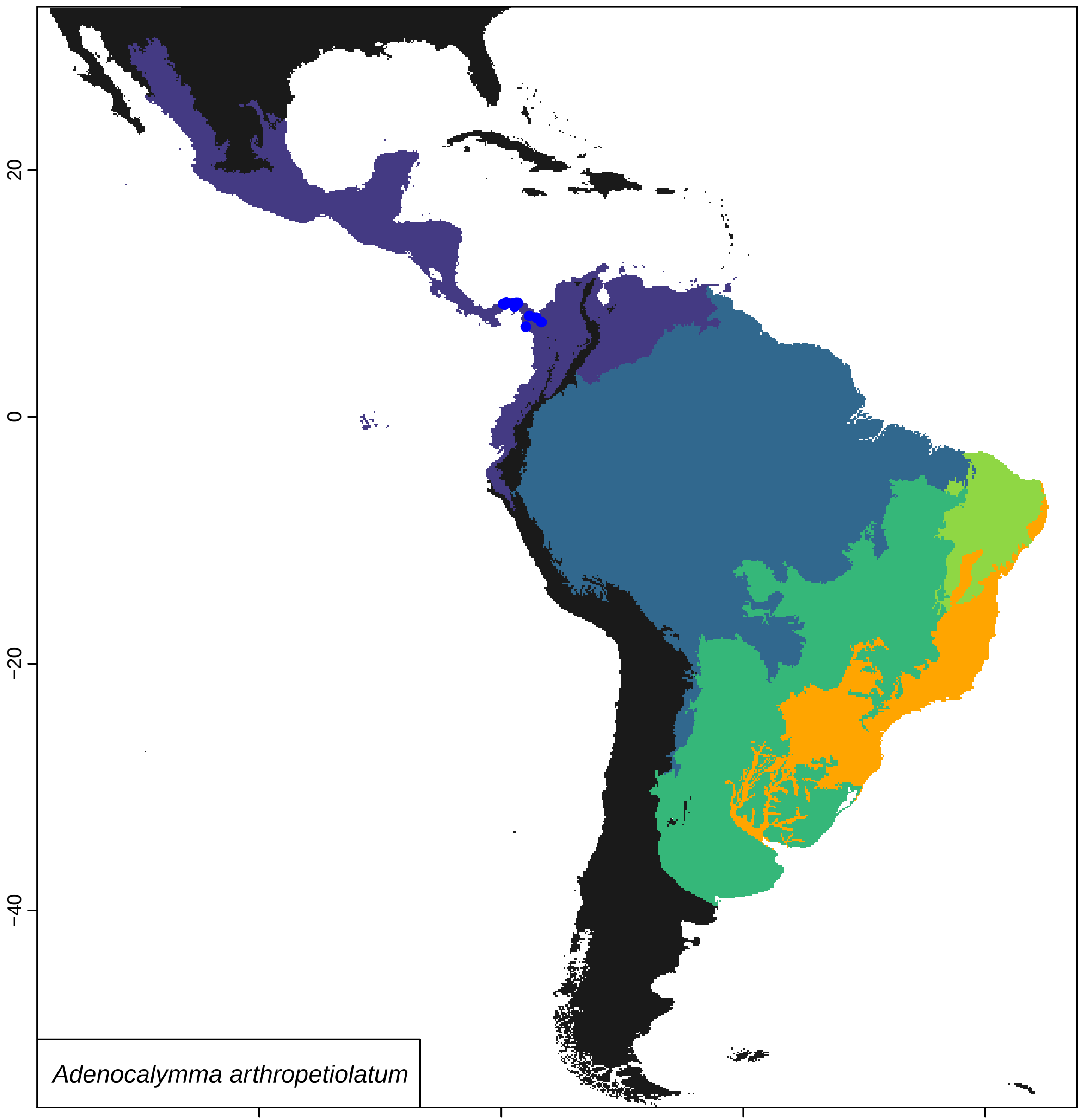

20
0
−20
−40
−100
−80
−60
−40
Adenocalymma arthropetiolatum

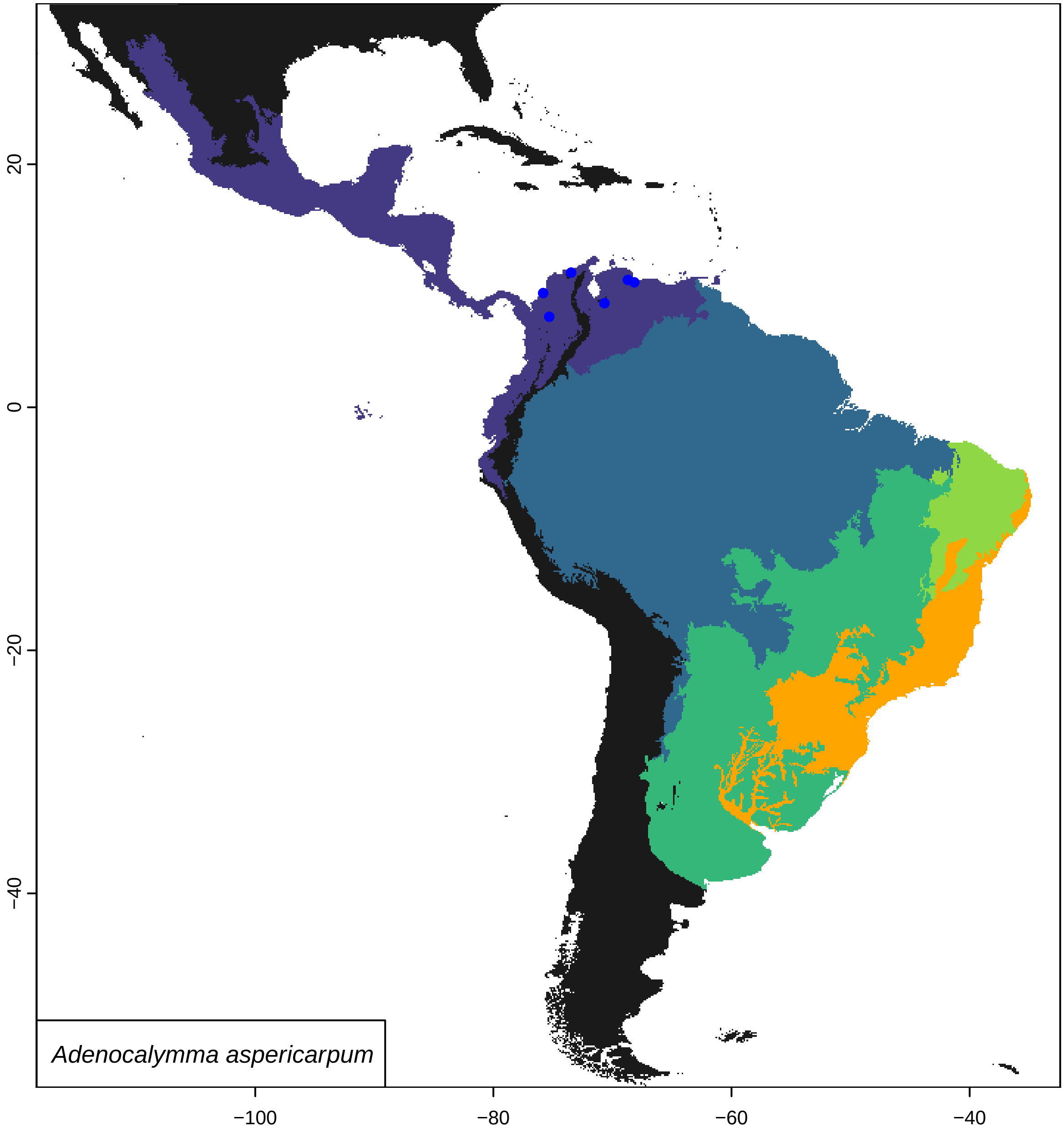

20
0
−20
−40
−100
−80
−60
−40
Adenocalymma aspericarpum

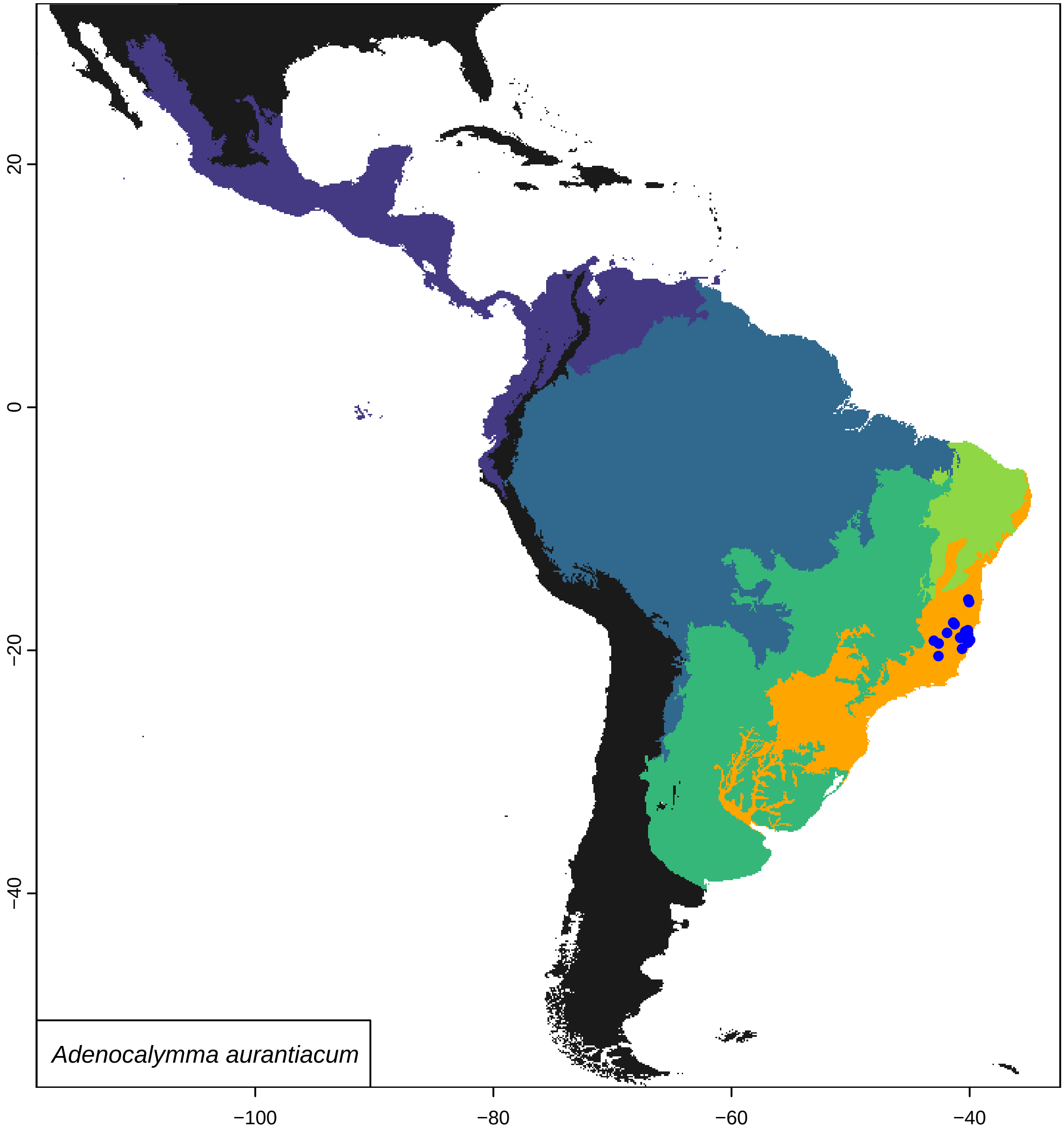

20
0
−20
−40
−100
−80
−60
−40
Adenocalymma aurantiacum

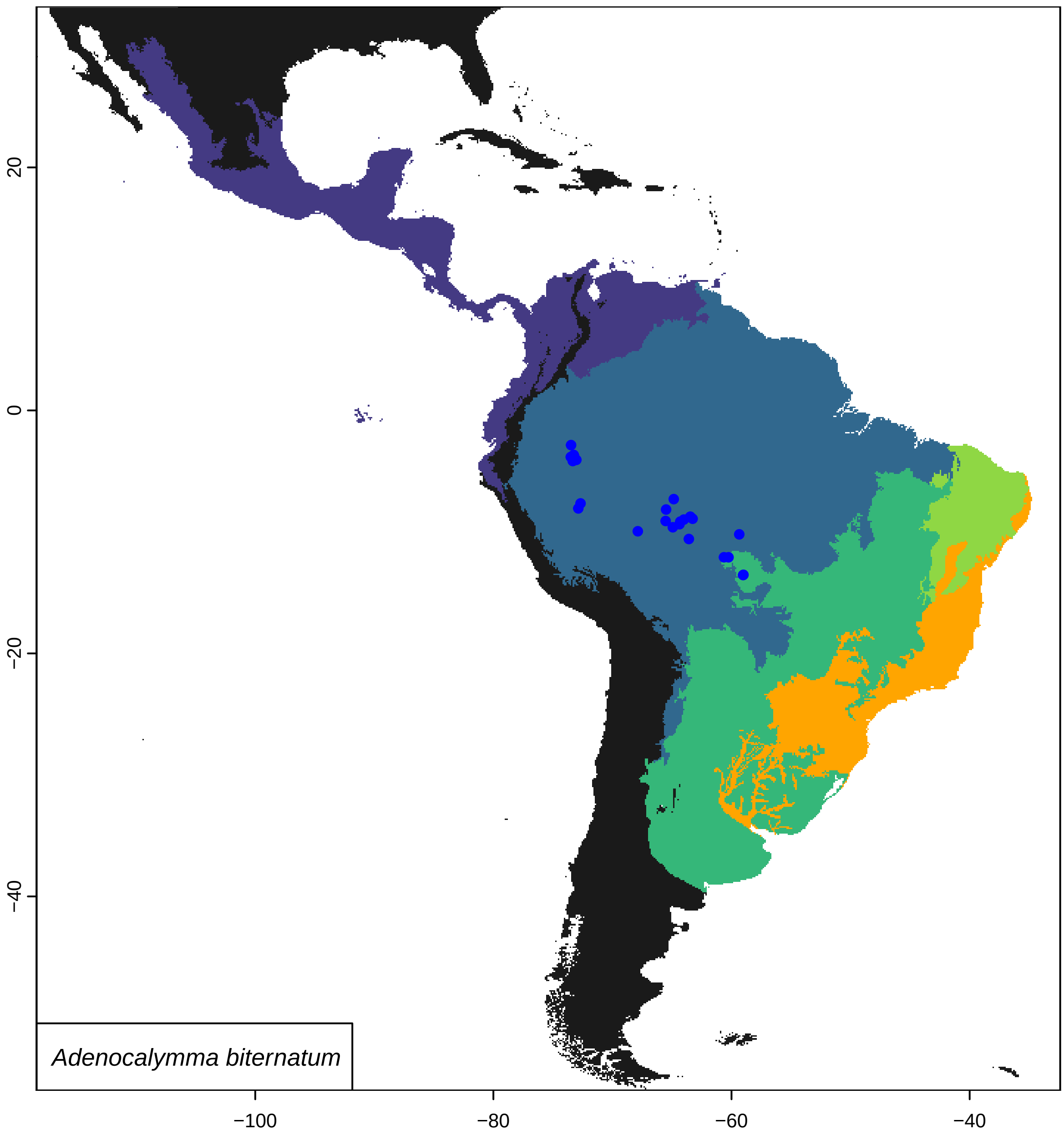

20
0
−20
−40
−100
−80
−60
−40
Adenocalymma biternatum

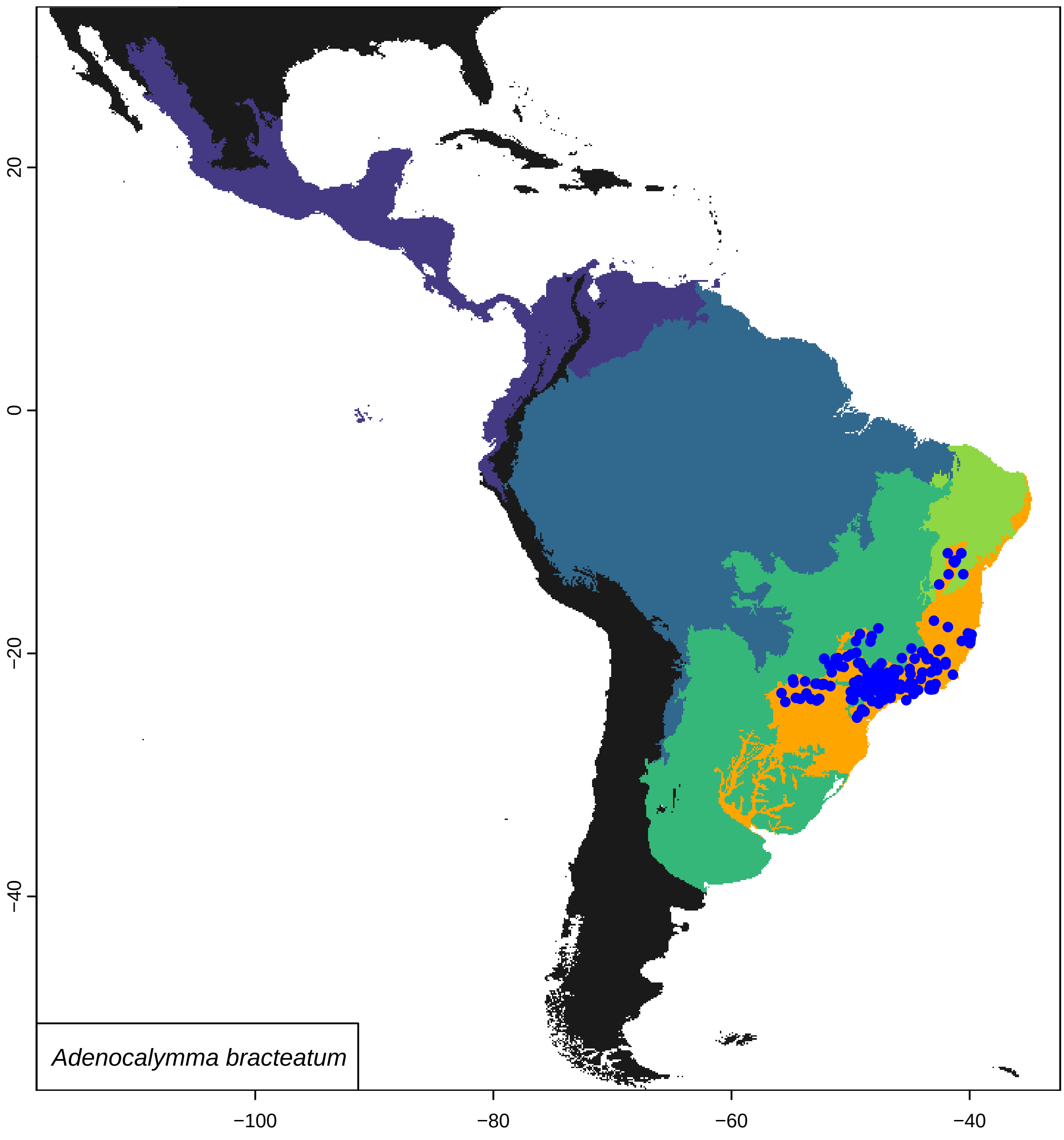

20
0
−20
−40
−100
−80
−60
−40
Adenocalymma bracteatum

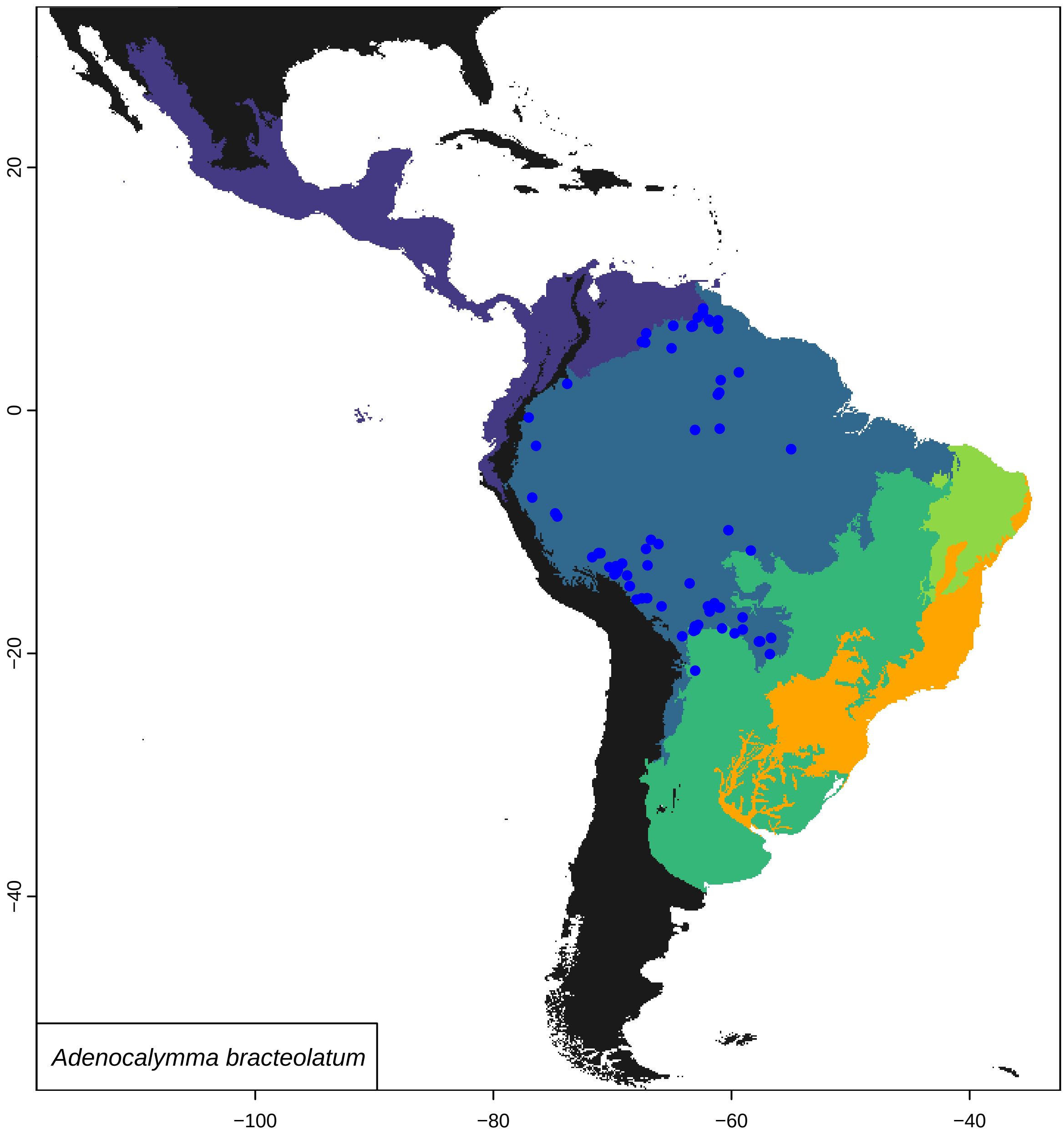

Adenocalymma bracteolatum
20
0
−20
−40
−100
−80
−60
−40

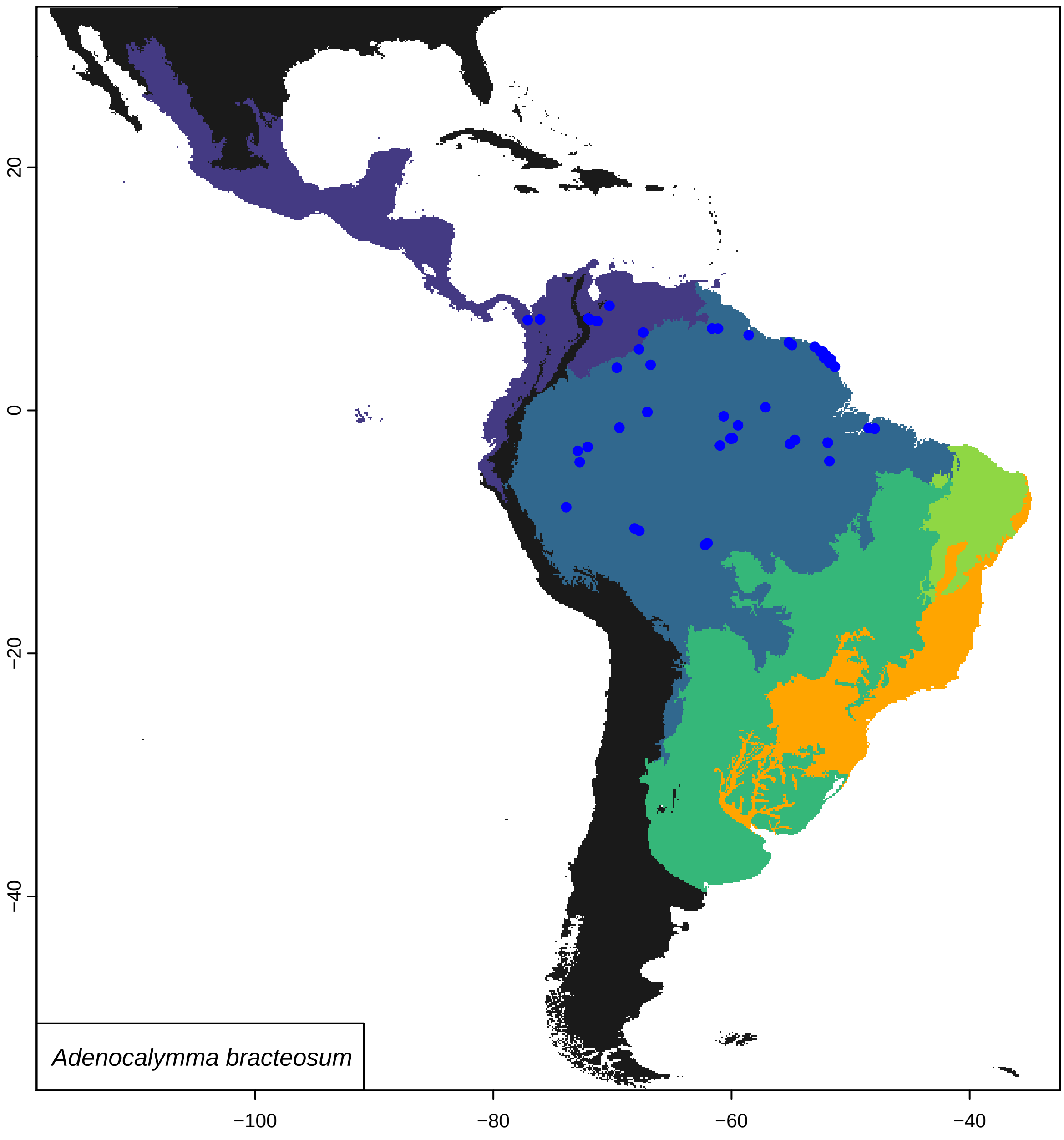
20
0
−20
−40
−100
−80
−60
−40
Adenocalymma bracteosum

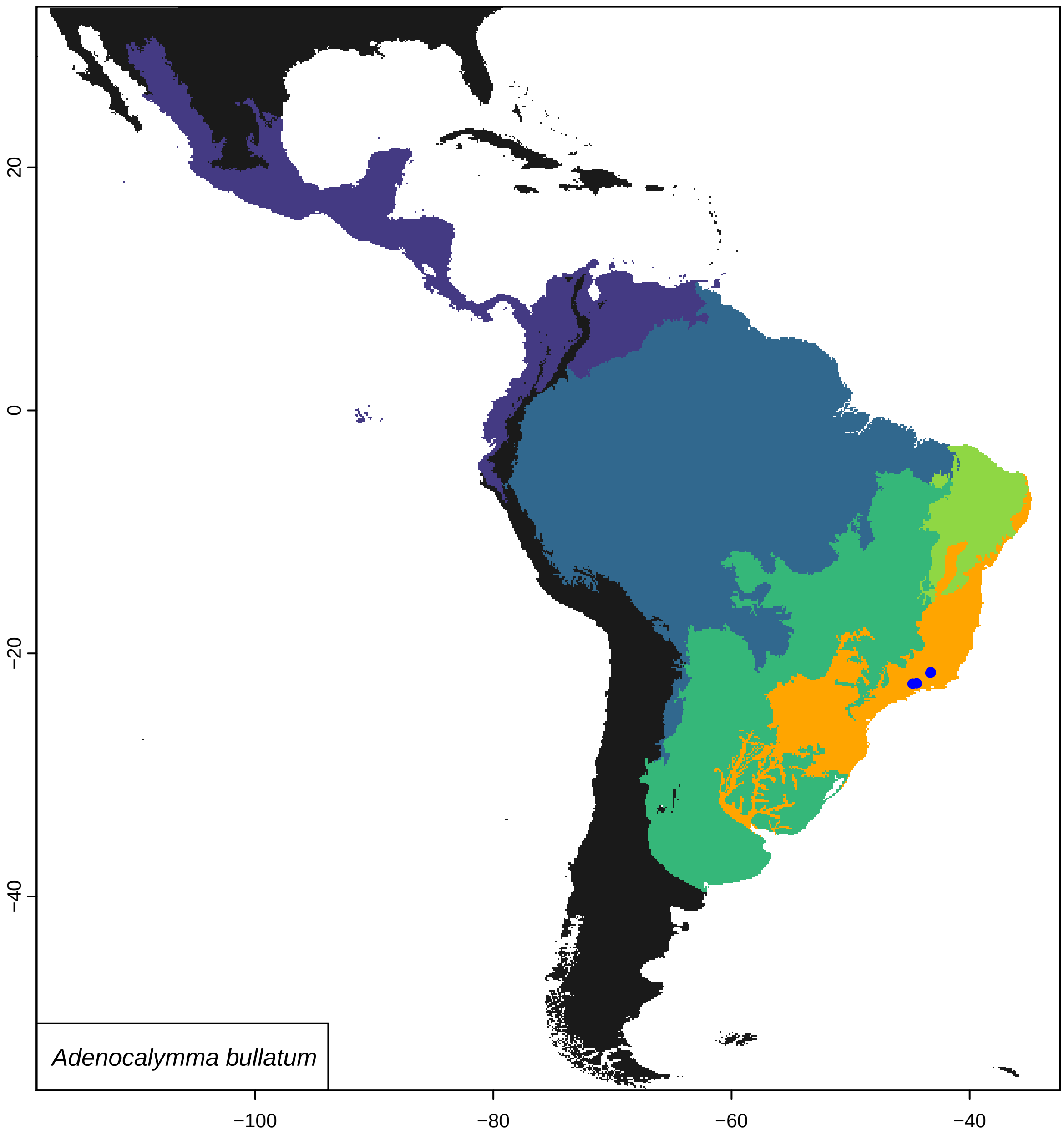
20
0
−20
−40
−100
−80
−60
−40
Adenocalymma bullatum

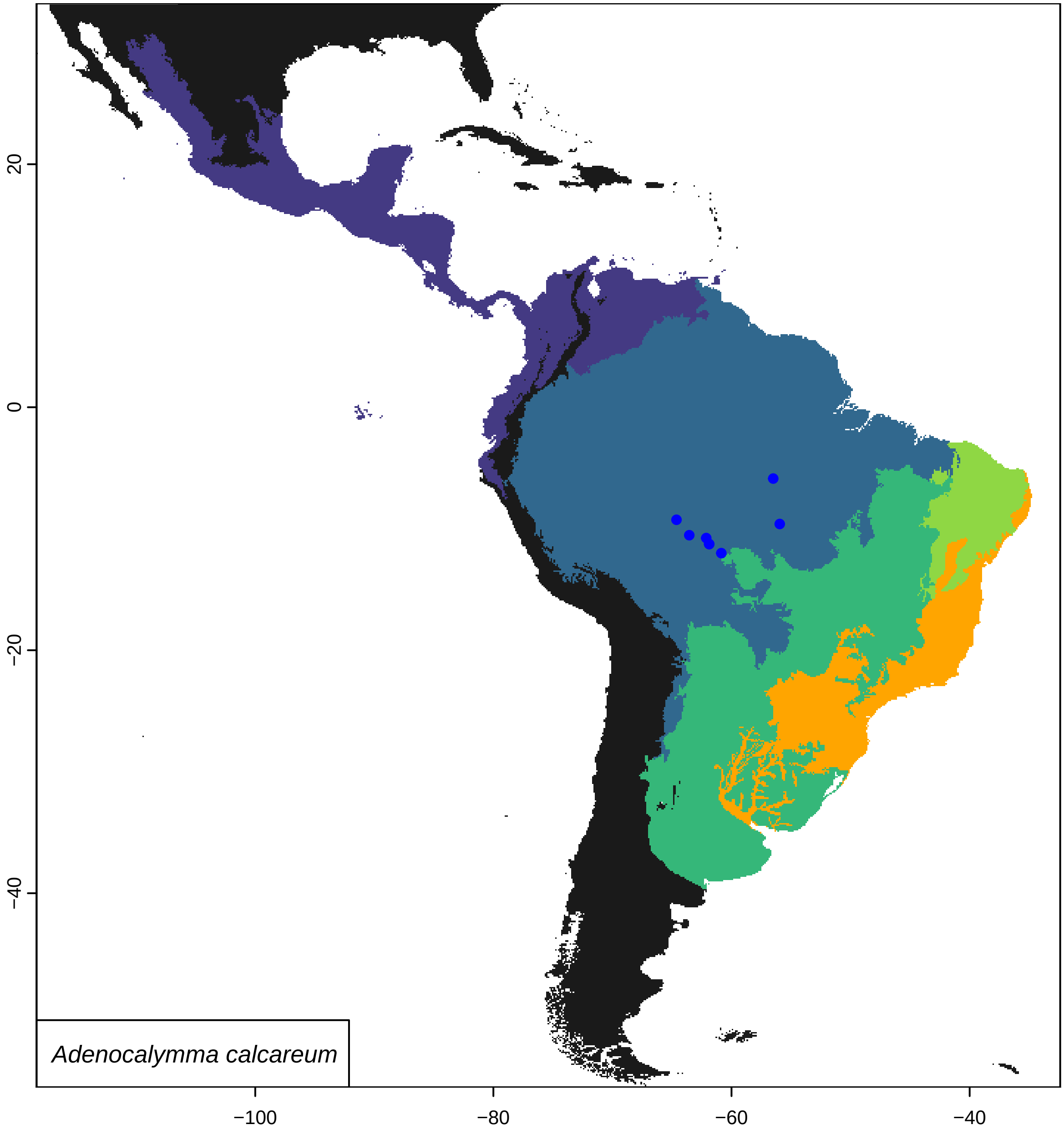

20
0
−20
−40
−100
−80
−60
−40
Adenocalymma calcareum

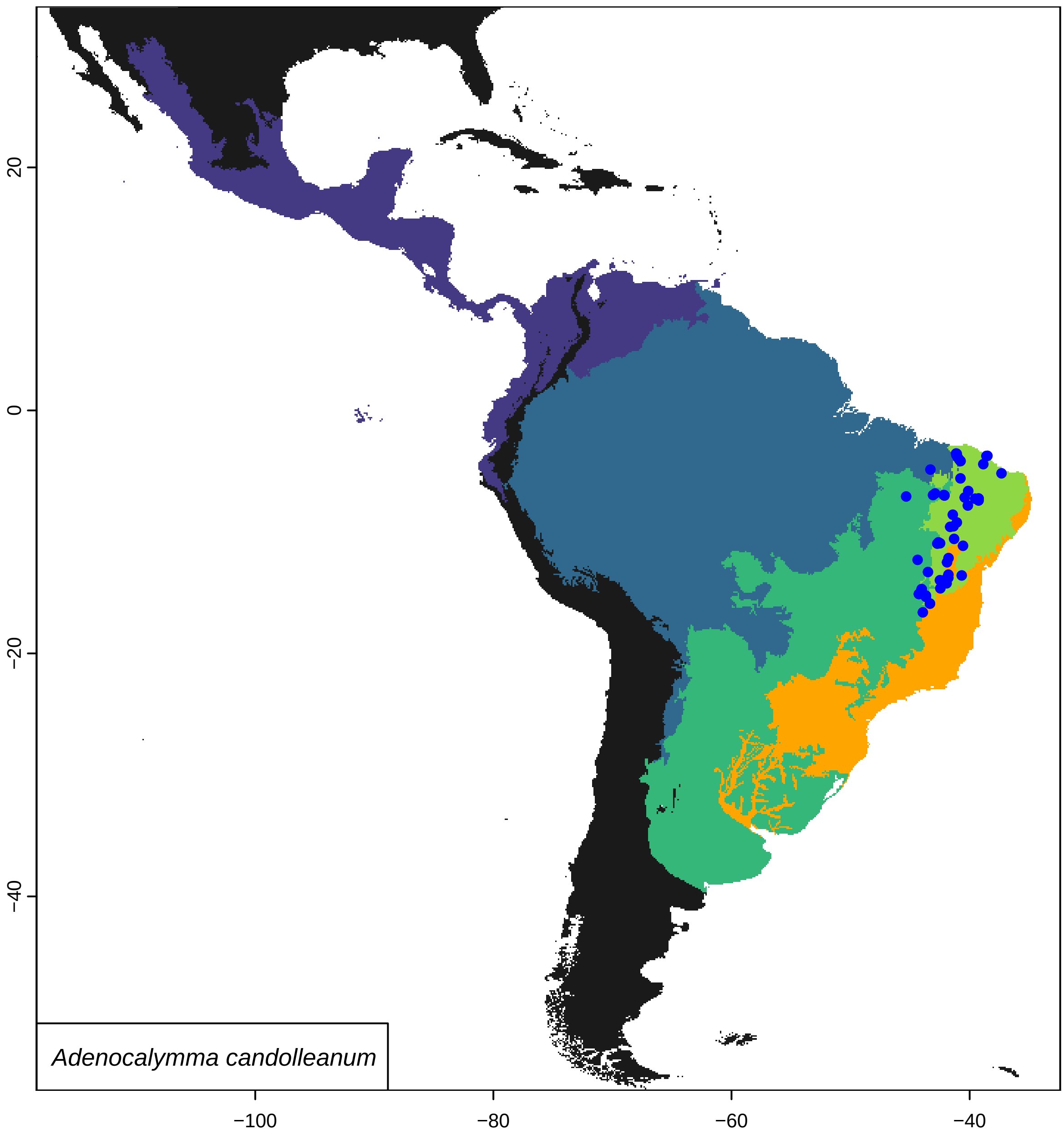

20
0
−20
−40
−100
−80
−60
−40
Adenocalymma candolleanum

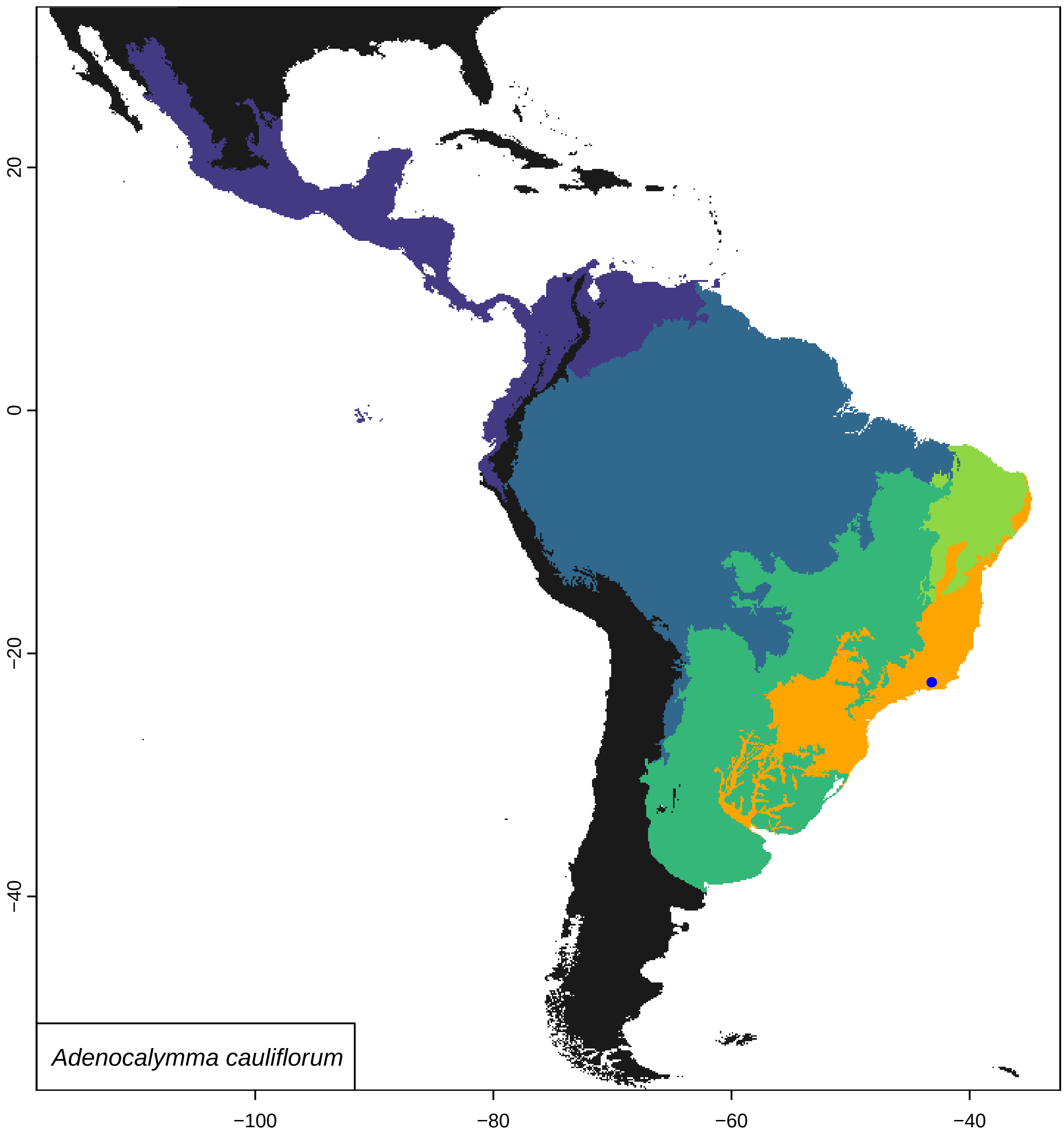
20
0
−20
−40
−100
−80
−60
−40
Adenocalymma cauliflorum

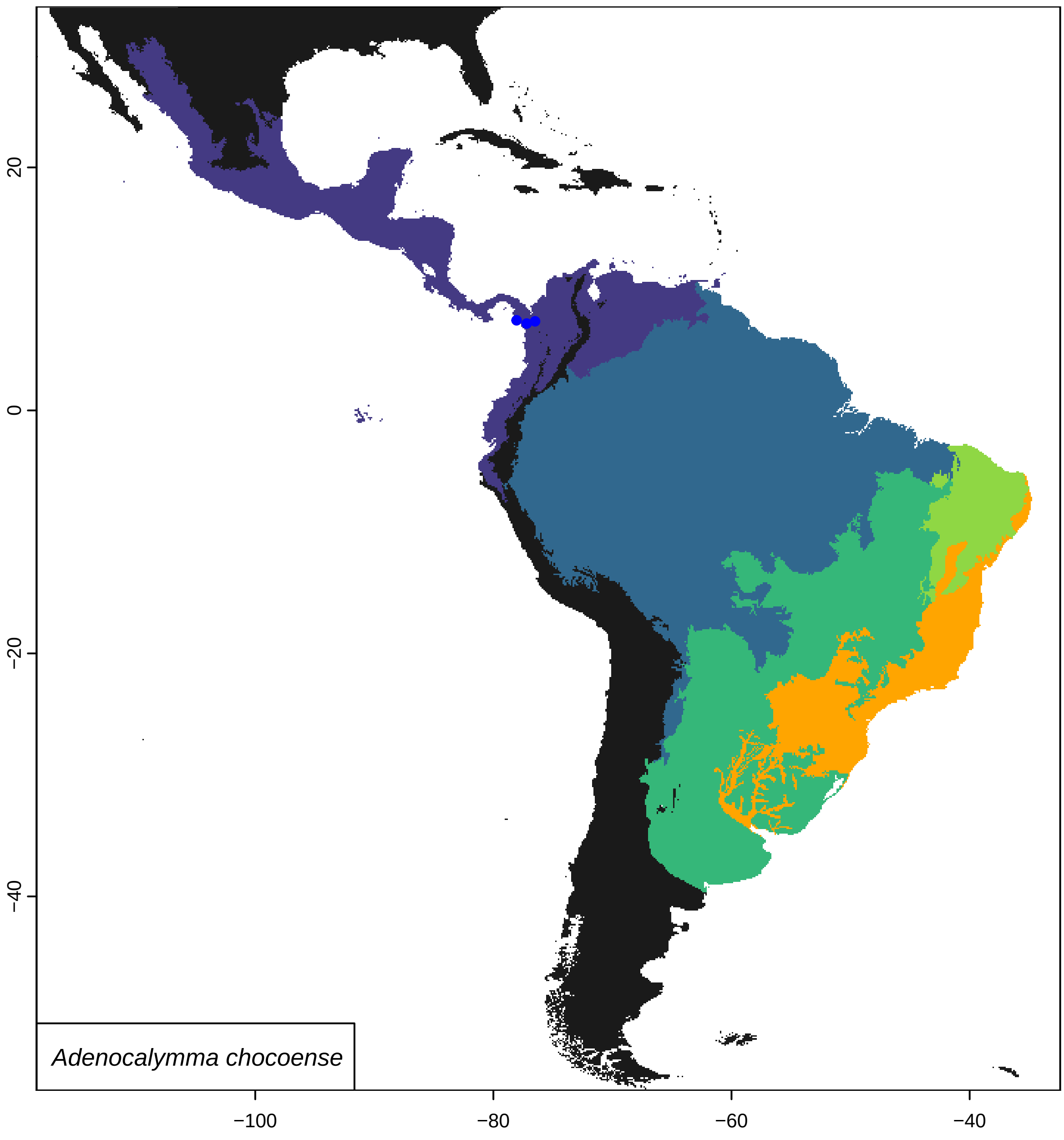

20
0
−20
−40
−100
−80
−60
−40
Adenocalymma chocoense

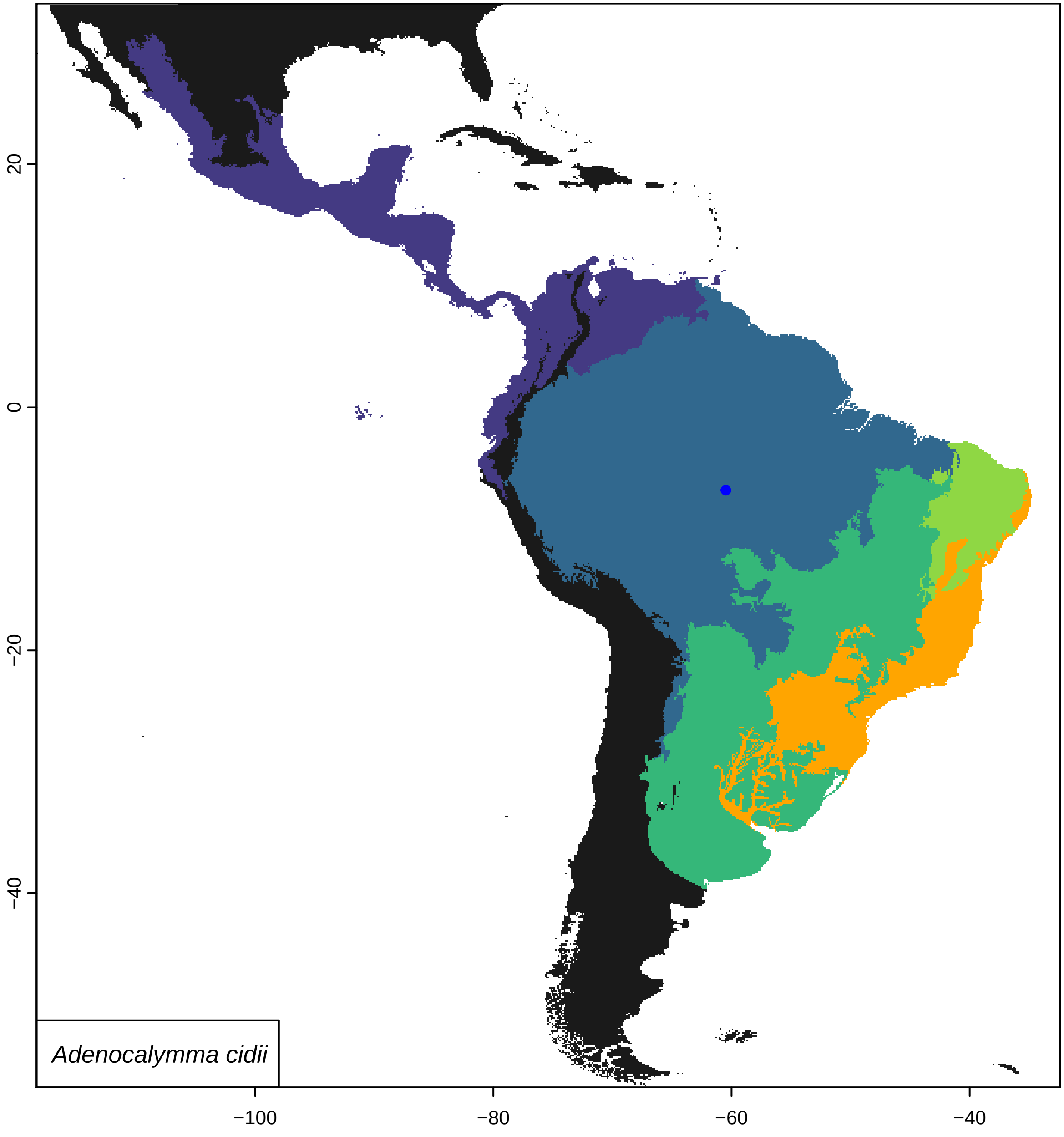

20
0
−20
−40
−100
−80
−60
−40
Adenocalymma cidii

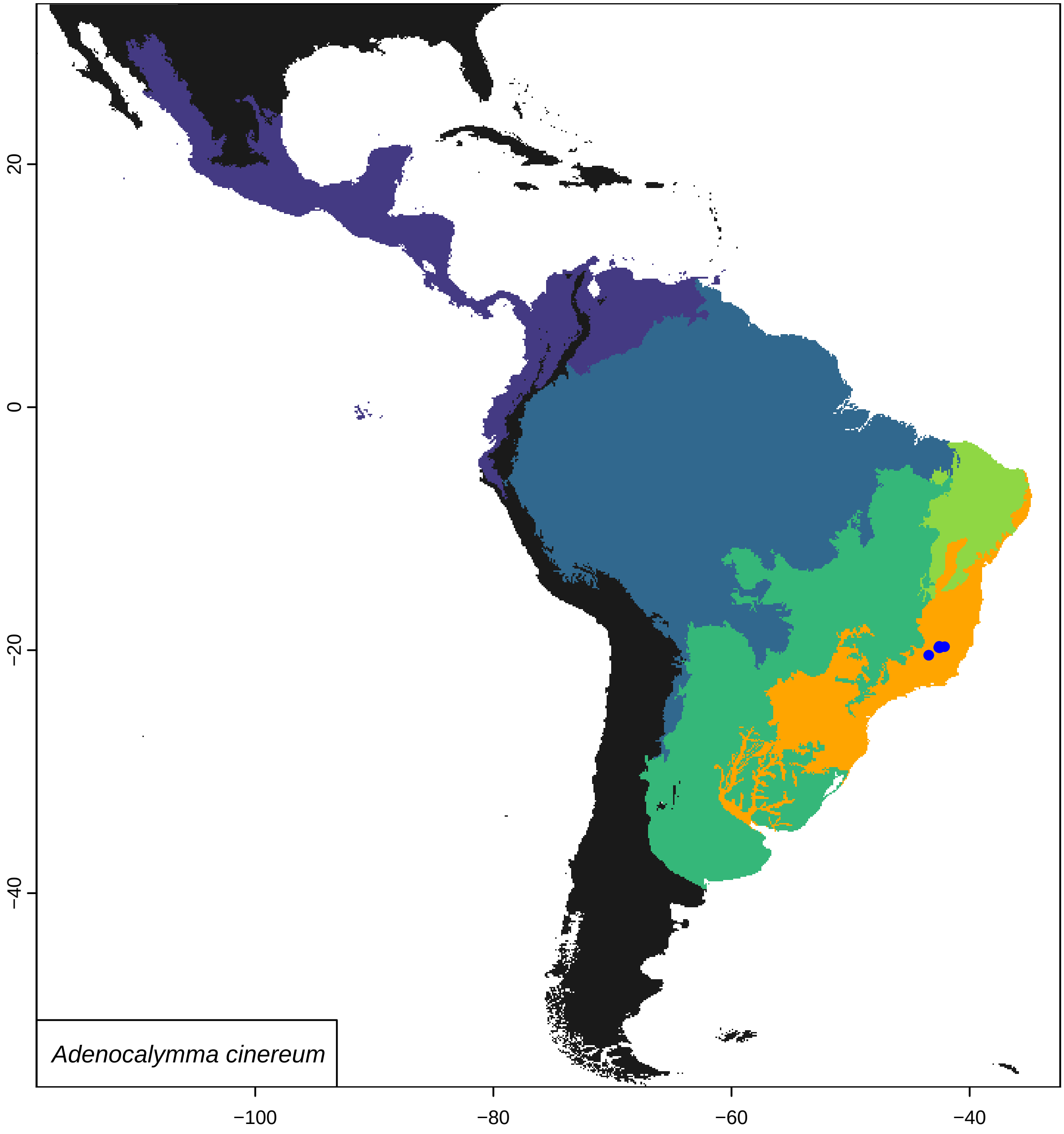

20
0
−20
−40
−100
−80
−60
−40
Adenocalymma cinereum

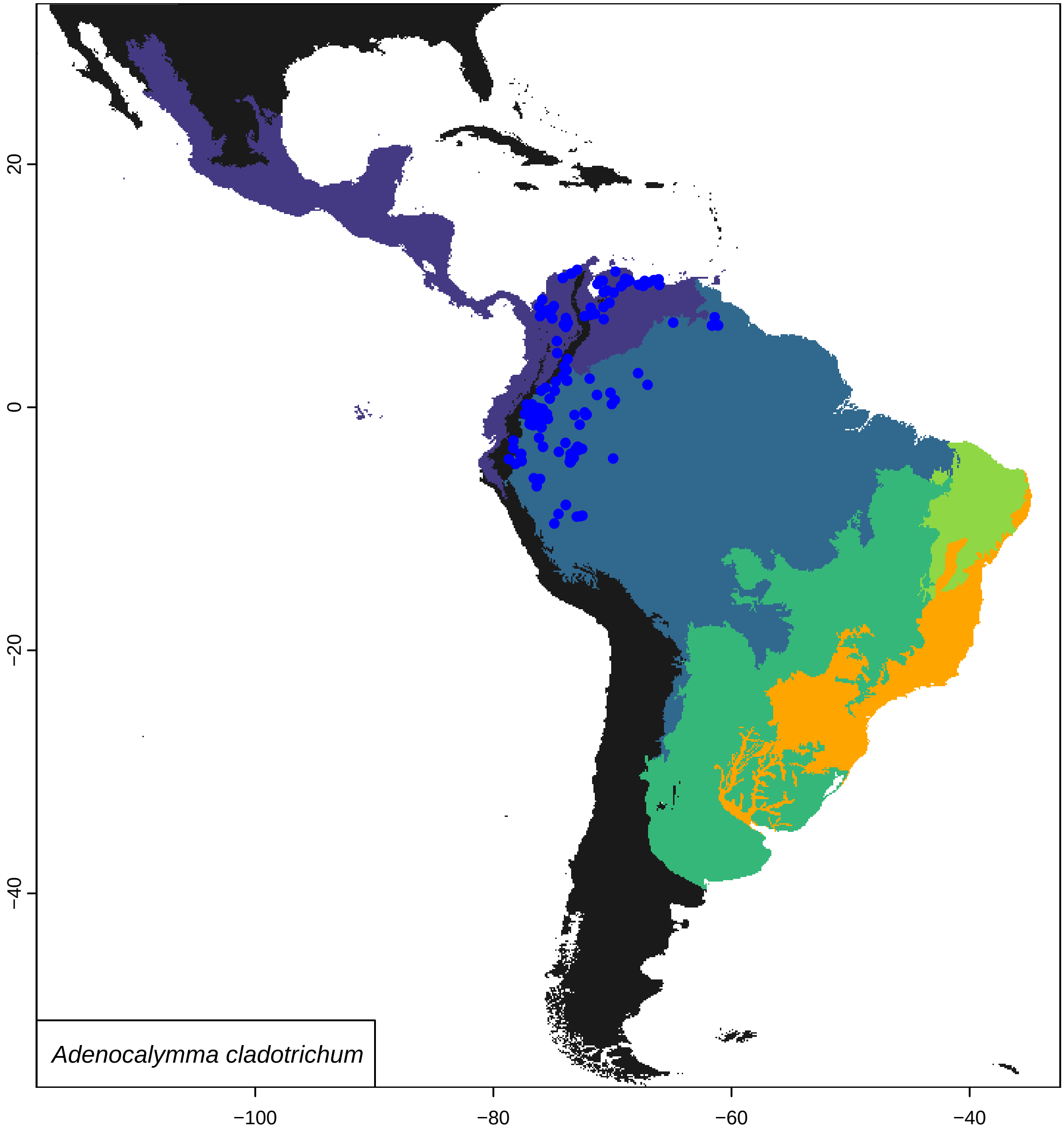

20
0
−20
−40
−100
−80
−60
−40
Adenocalymma cladotrichum

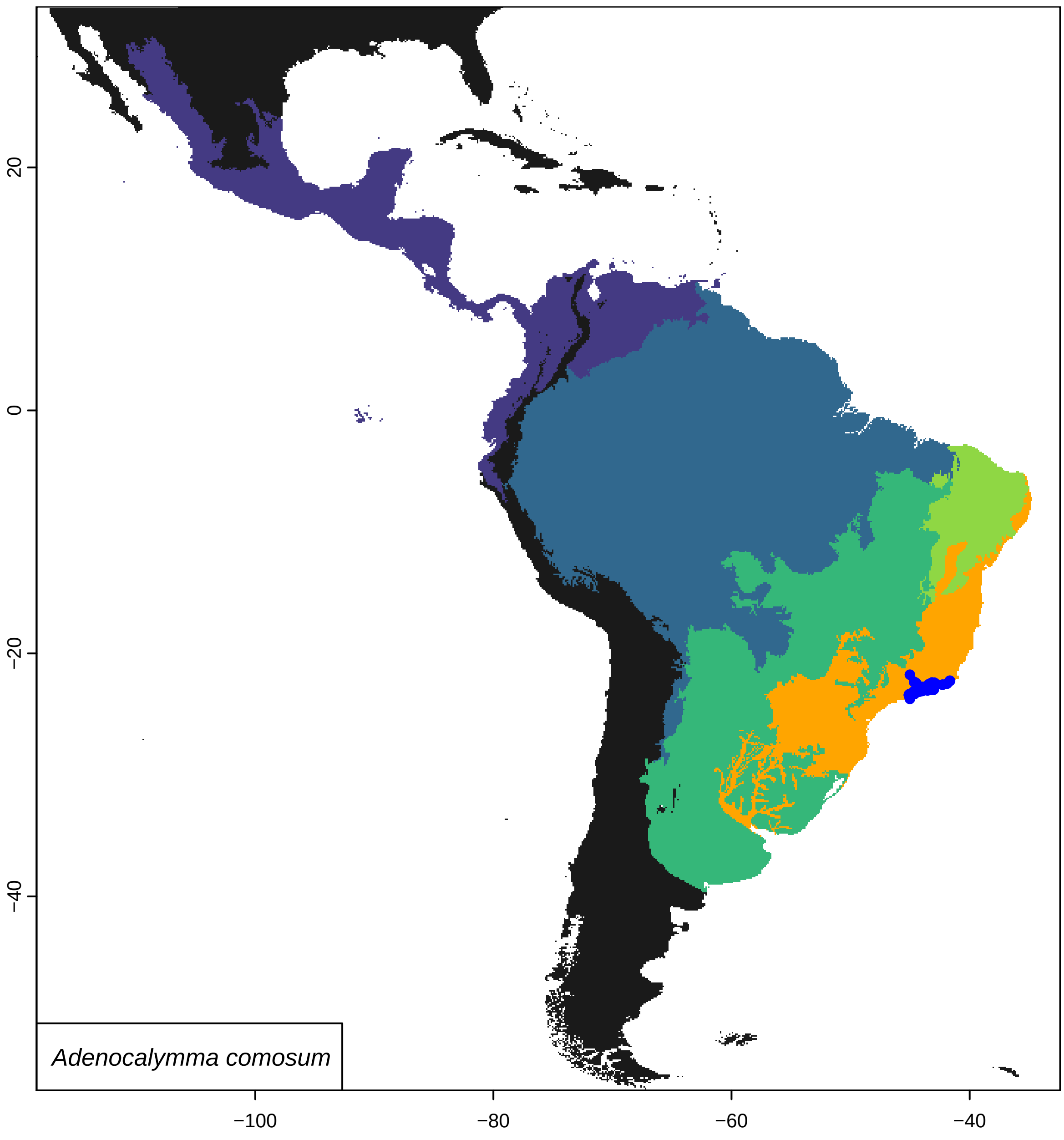
20
0
−20
−40
−100
−80
−60
−40
Adenocalymma comosum

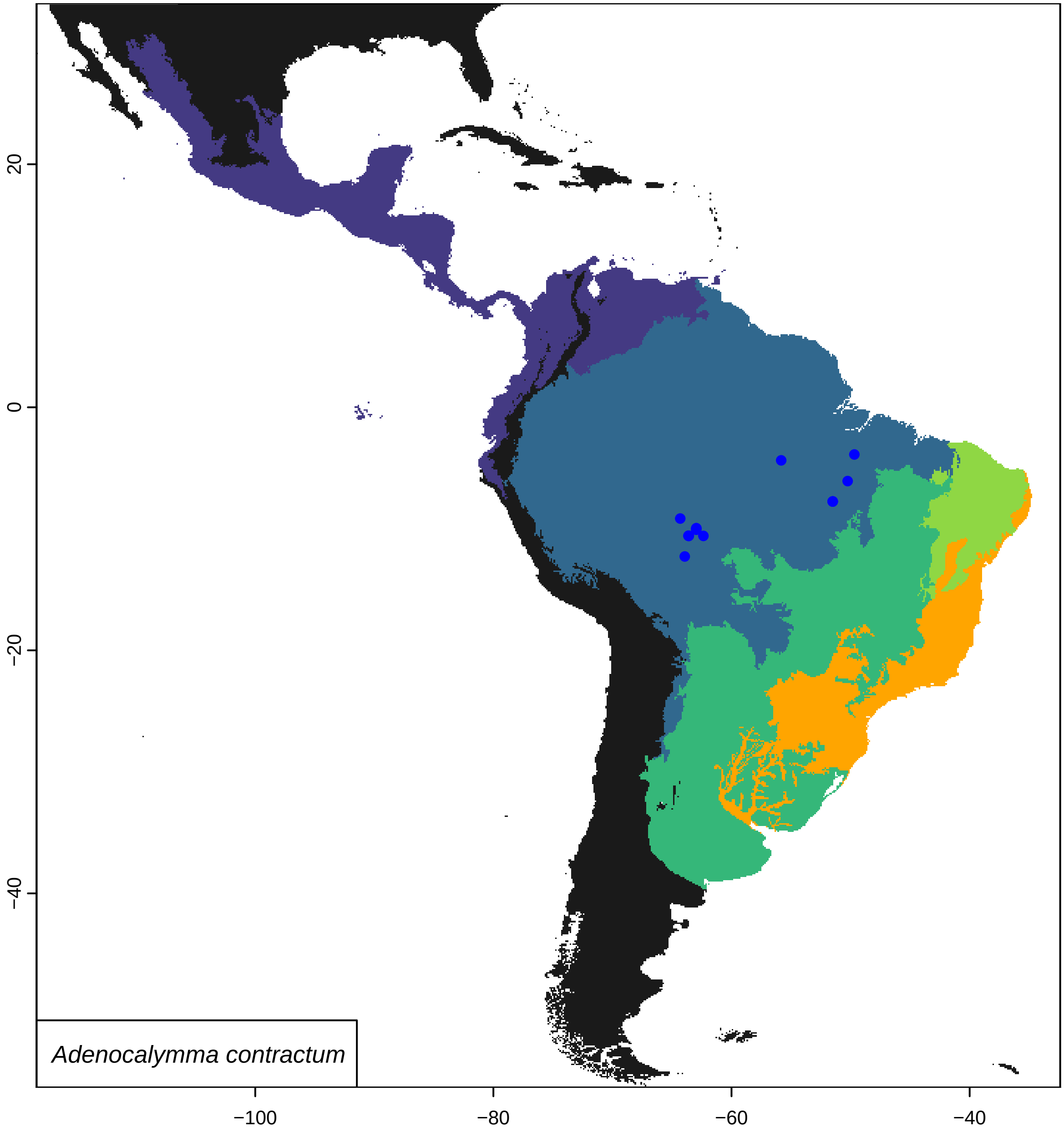

20
0
−20
−40
−100
−80
−60
−40
Adenocalymma contractum

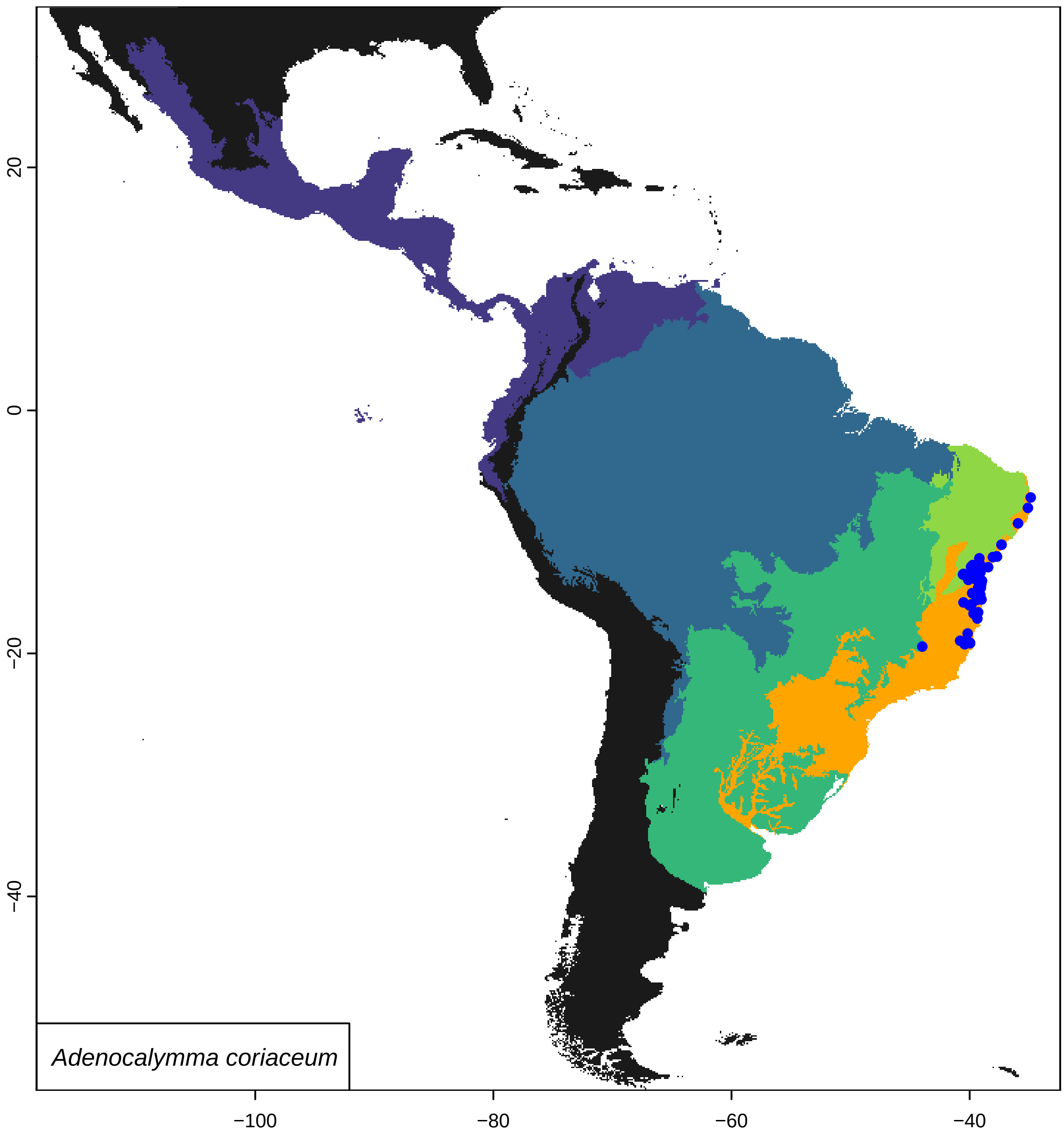

20
0
−20
−40
−100
−80
−60
−40
Adenocalymma coriaceum

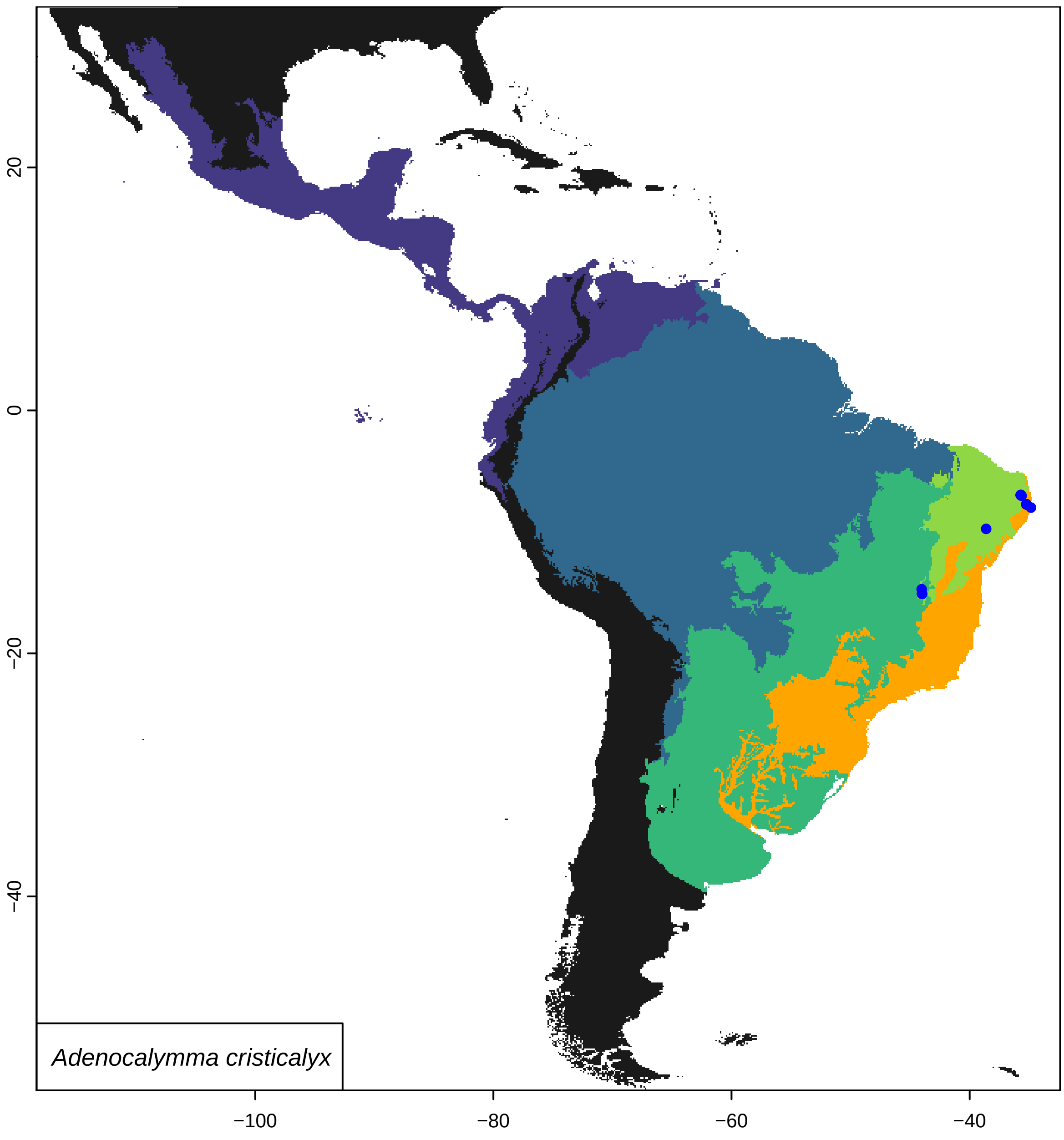
20
0
−20
−40
−100
−80
−60
−40
Adenocalymma cristicalyx

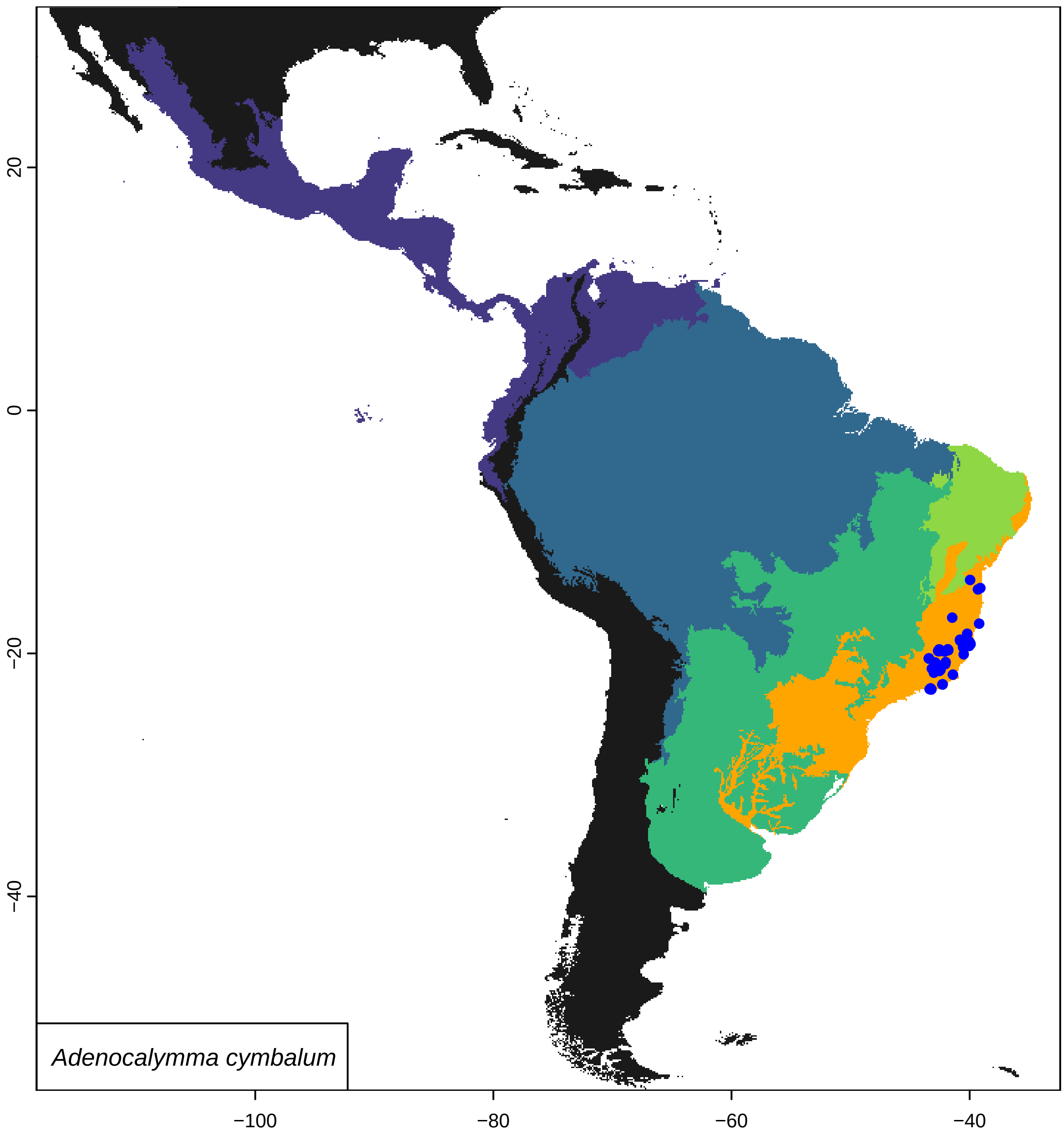

20
0
−20
−40
−100
−80
−60
−40
Adenocalymma cymbalum

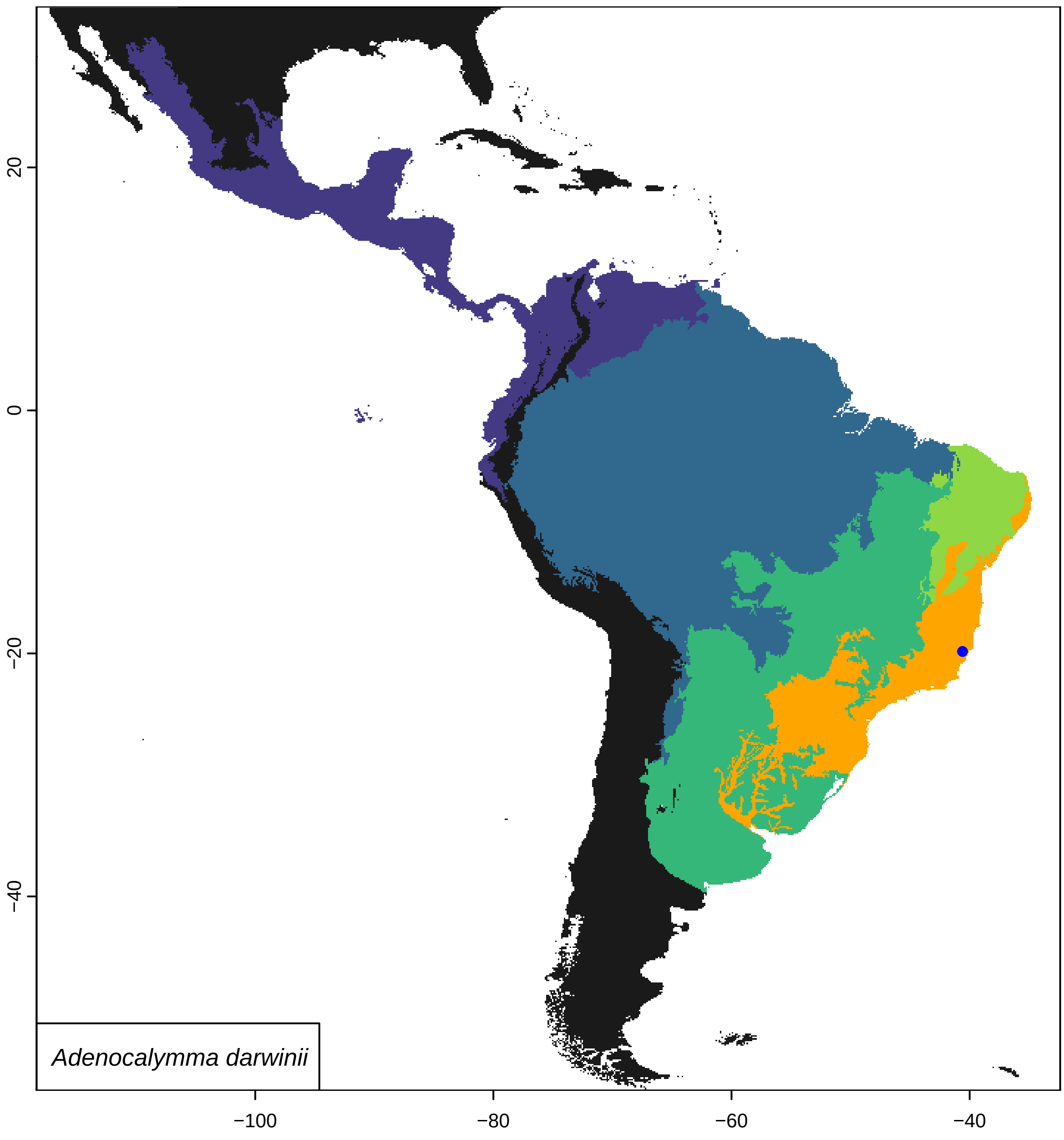

20
0
−20
−40
−100
−80
−60
−40
Adenocalymma darwinii

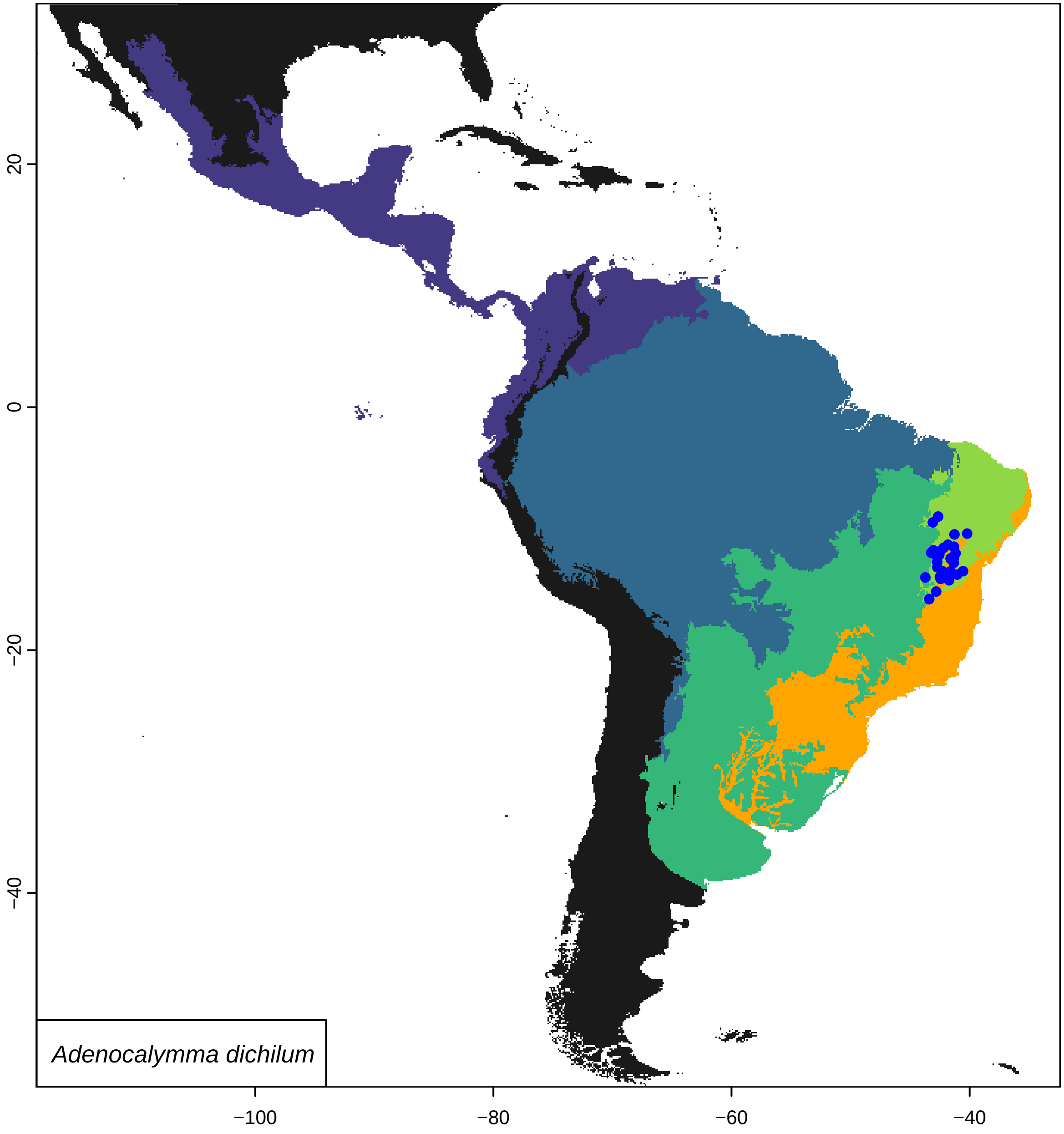

20
0
−20
−40
−100
−80
−60
−40
Adenocalymma dichilum

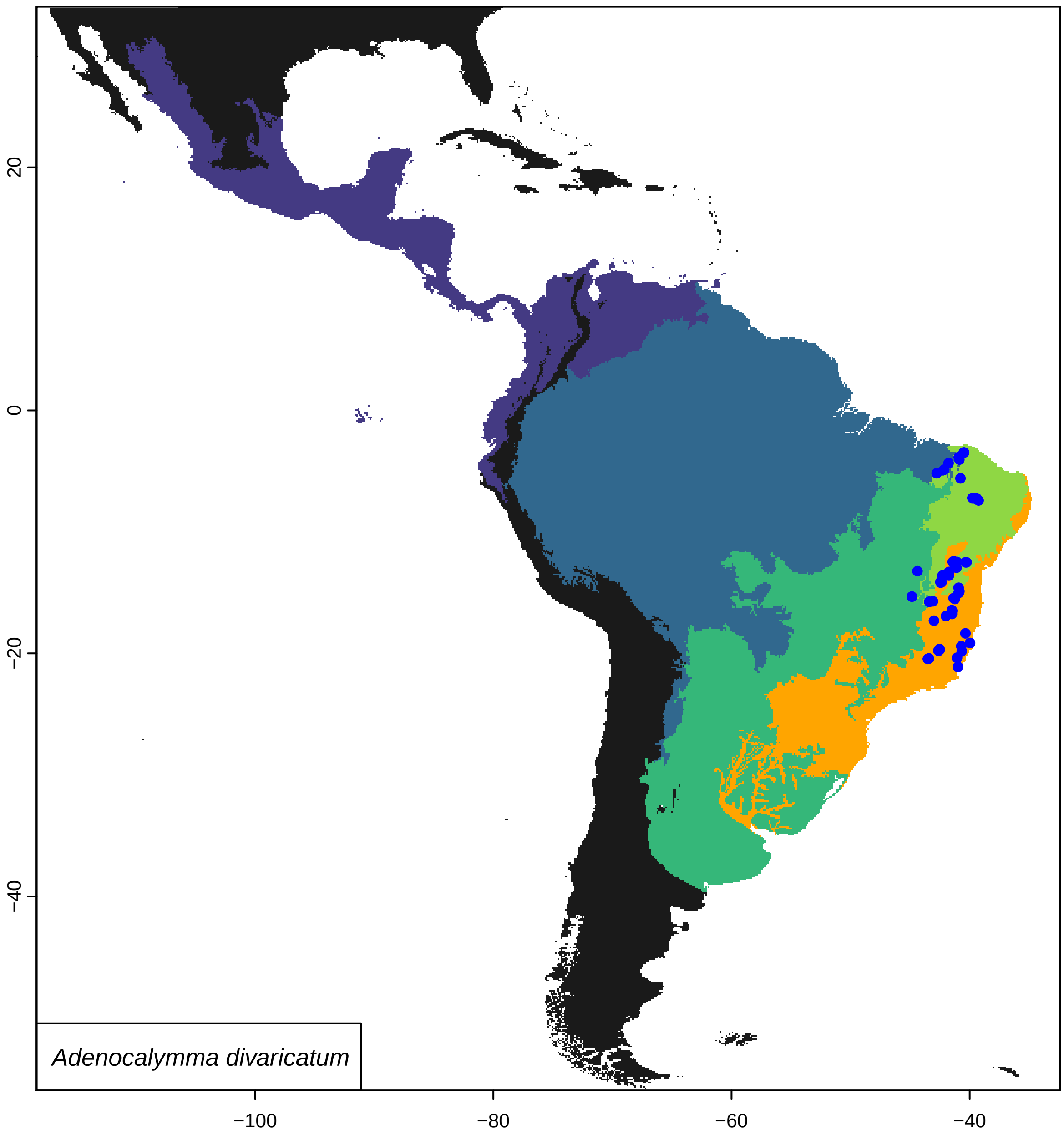
20
0
−20
−40
−100
−80
−60
−40
Adenocalymma divaricatum

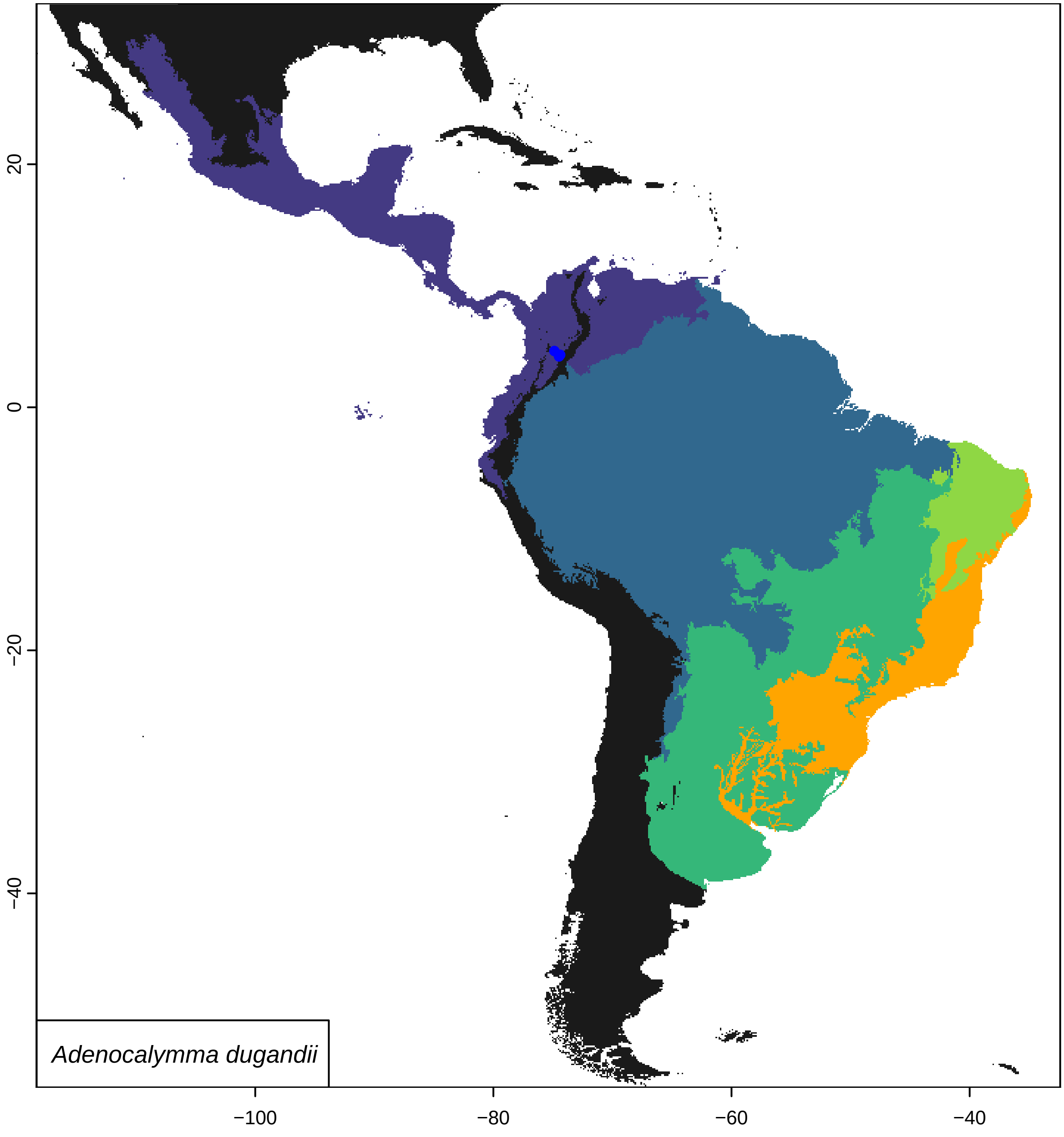

20
0
−20
−40
−100
−80
−60
−40
Adenocalymma dugandii

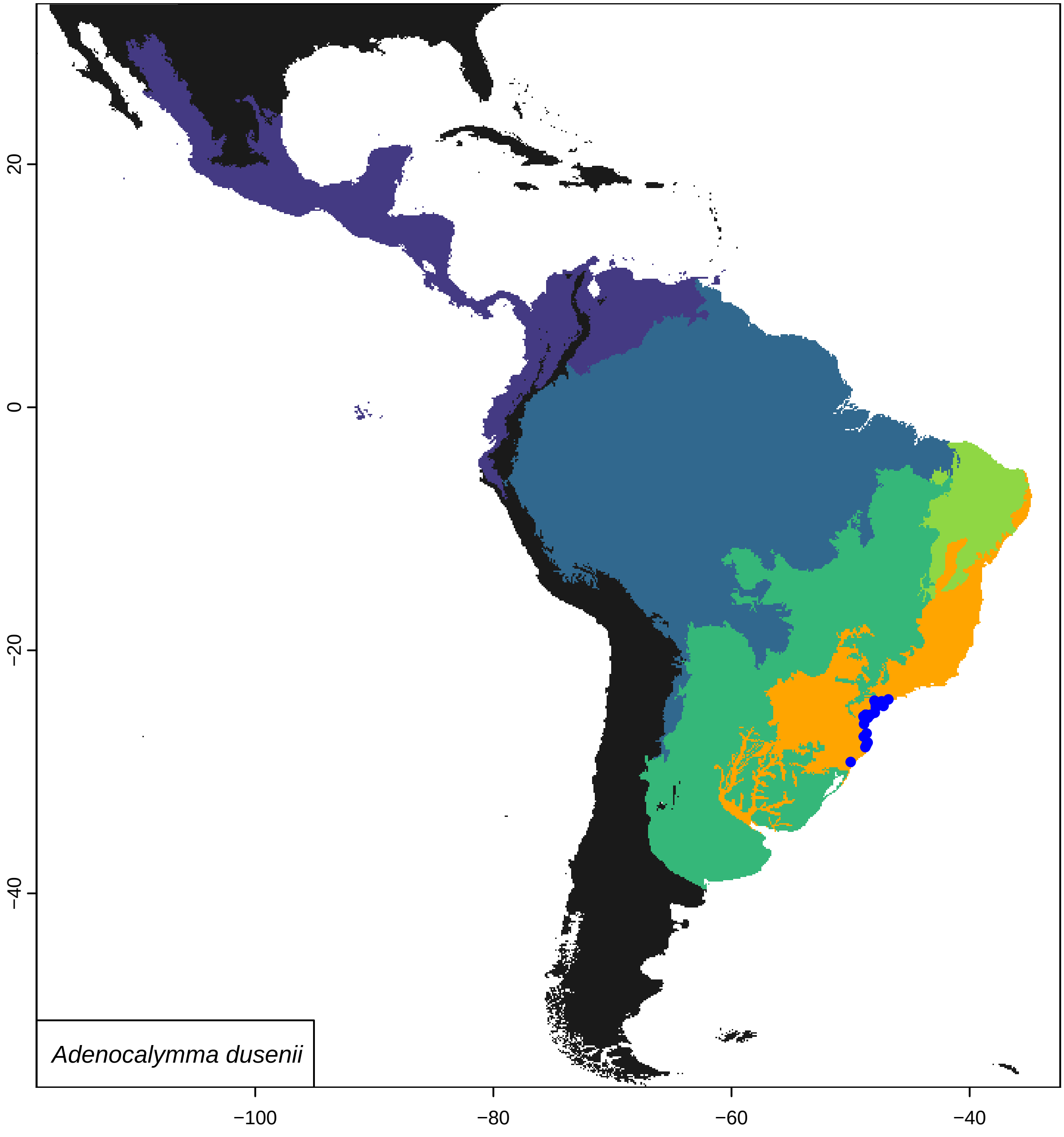

20
0
−20
−40
−100
−80
−60
−40
Adenocalymma dusenii

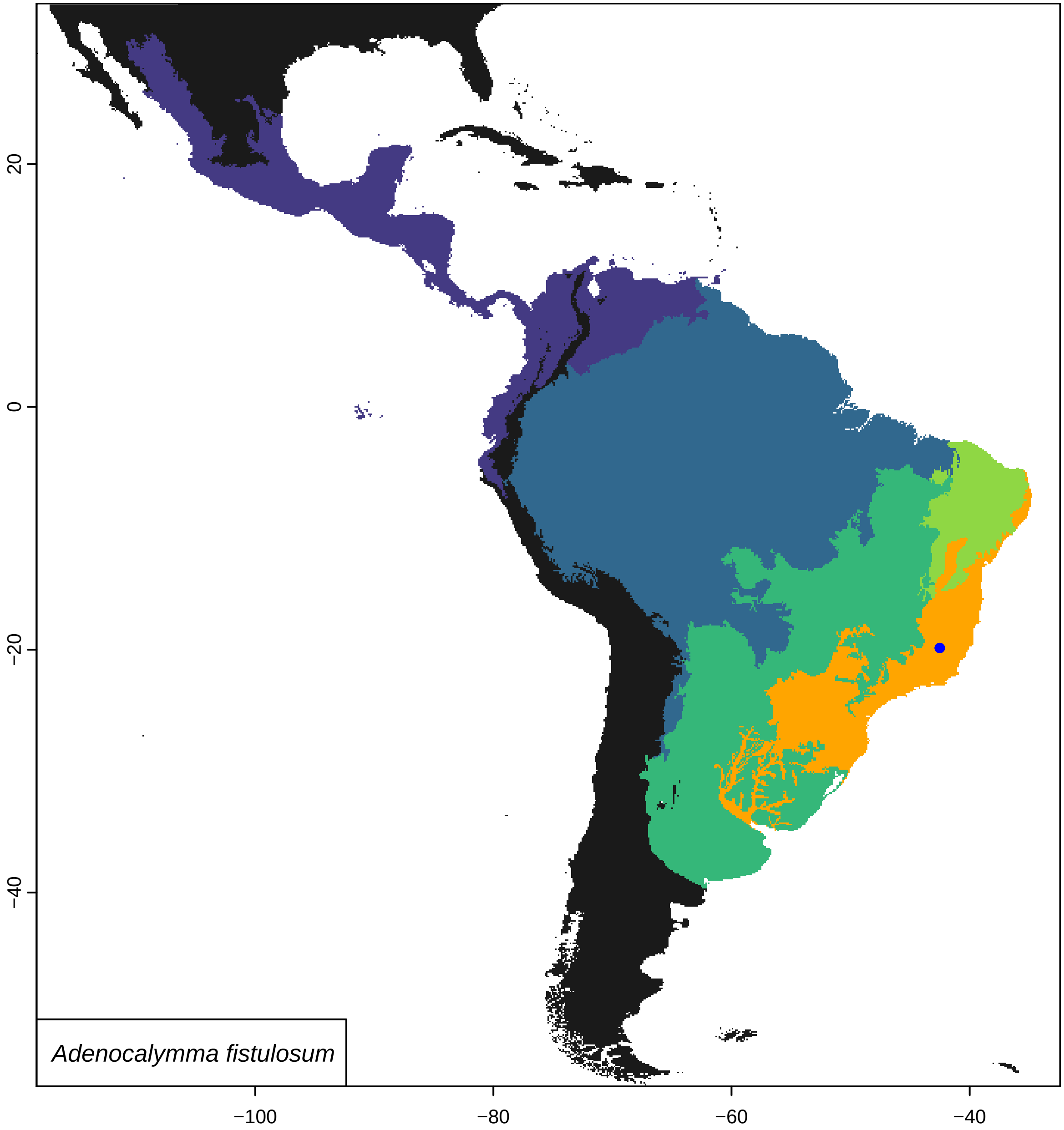

20
0
−20
−40
−100
−80
−60
−40
Adenocalymma fistulosum

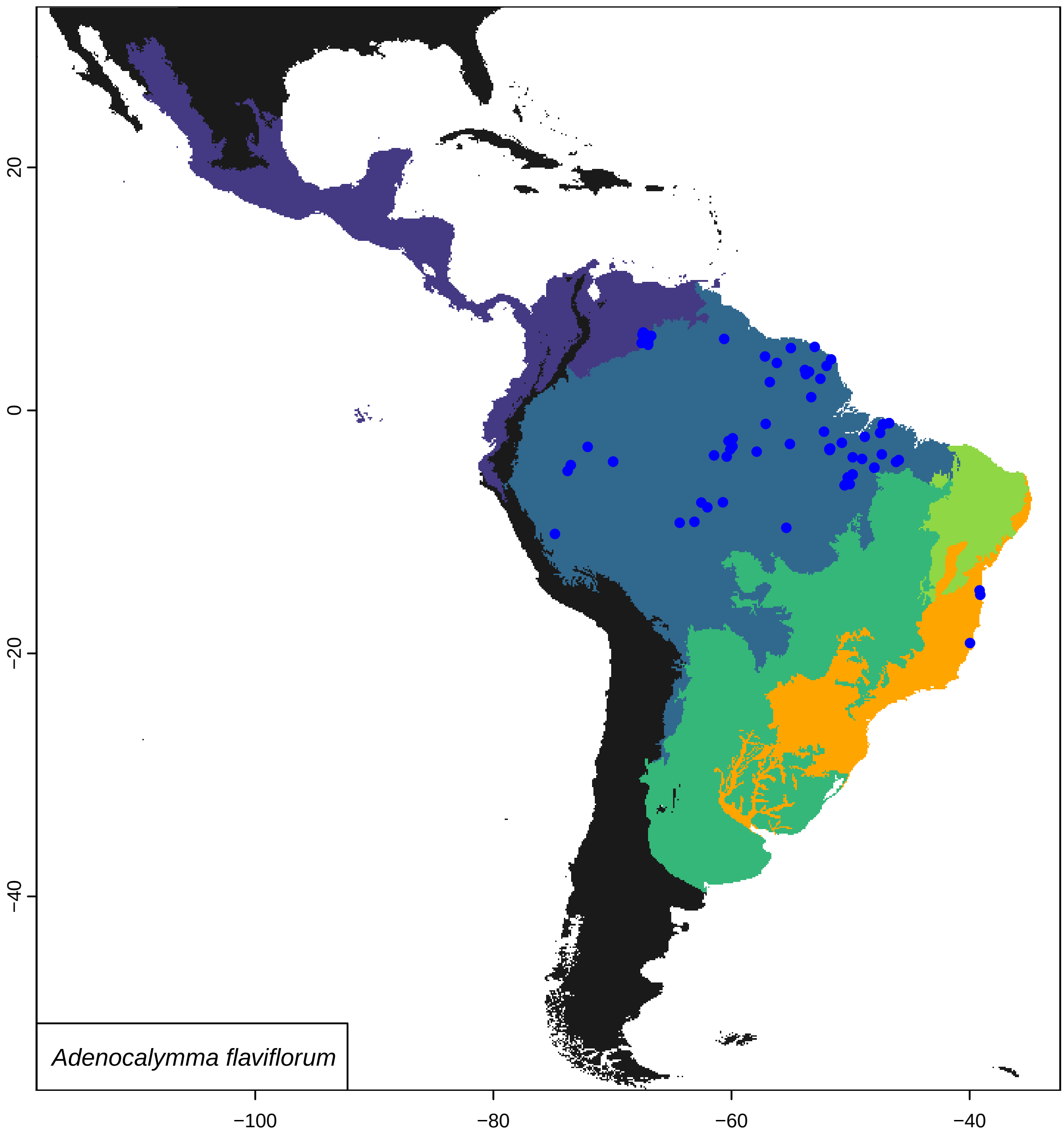

20
0
−20
−40
−100
−80
−60
−40
Adenocalymma flaviflorum

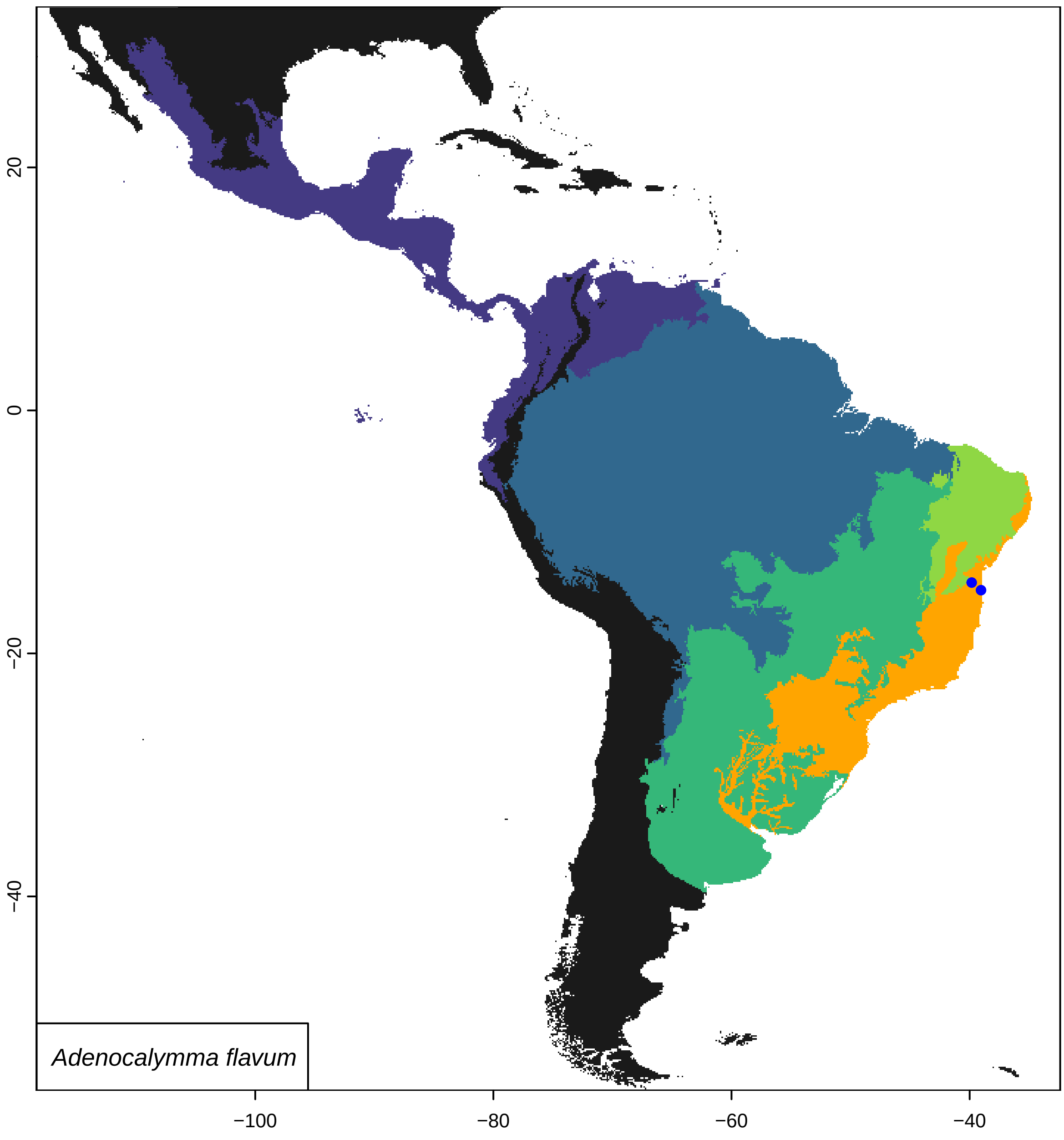

20
0
−20
−40
−100
−80
−60
−40
Adenocalymma flavum

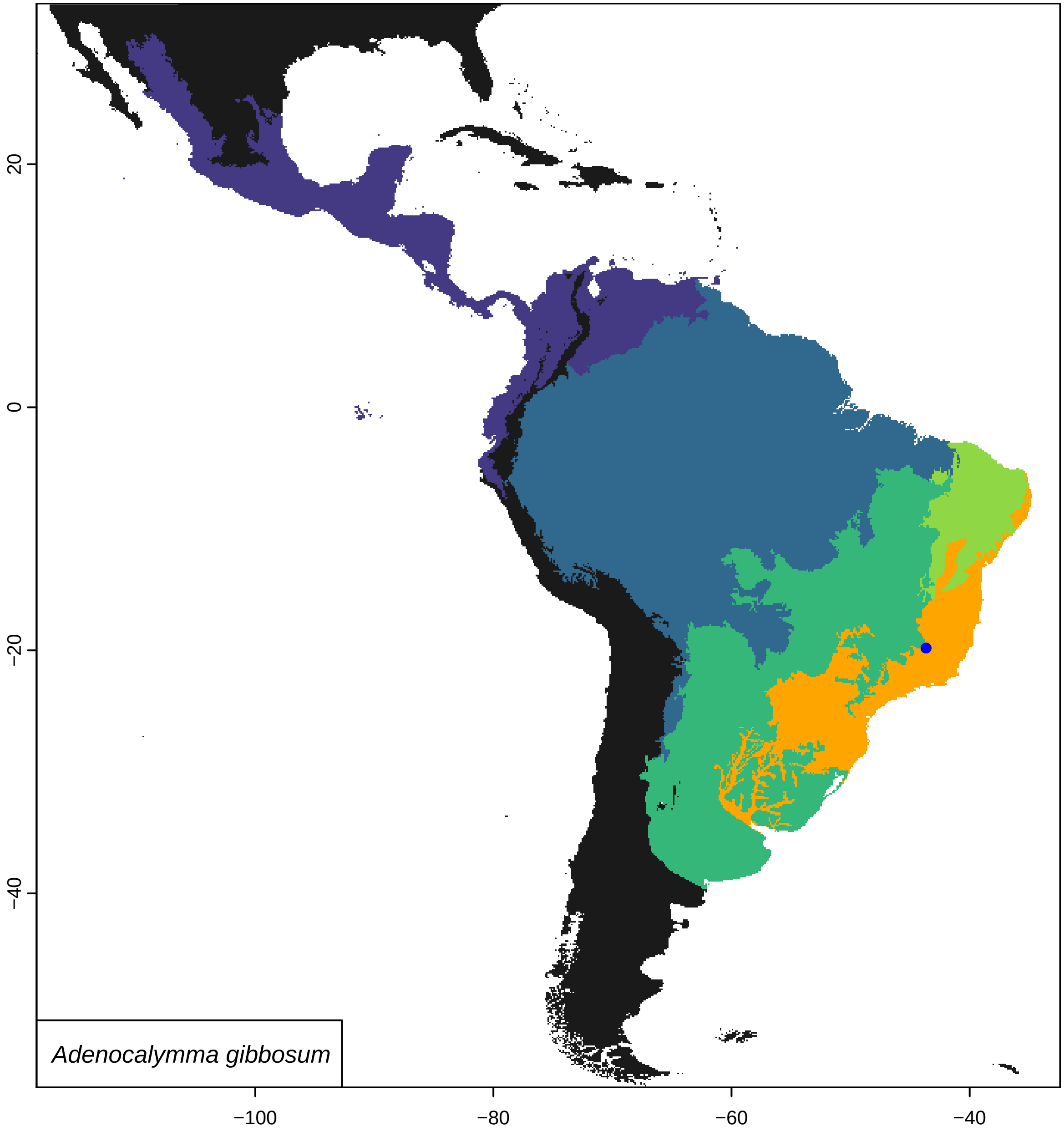

20
0
−20
−40
−100
−80
−60
−40
Adenocalymma gibbosum

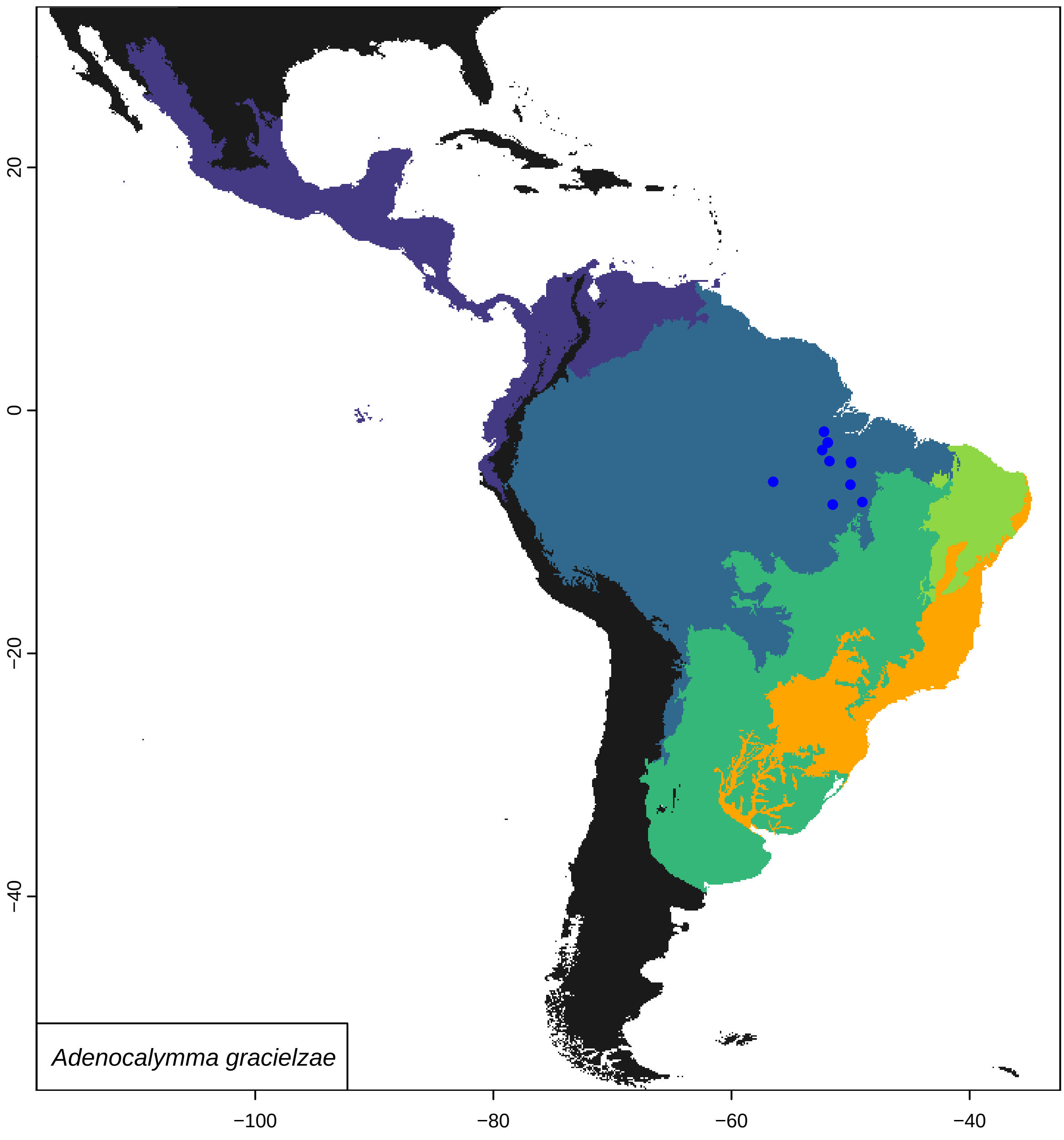

20
0
−20
−40
−100
−80
−60
−40
Adenocalymma gracielzae

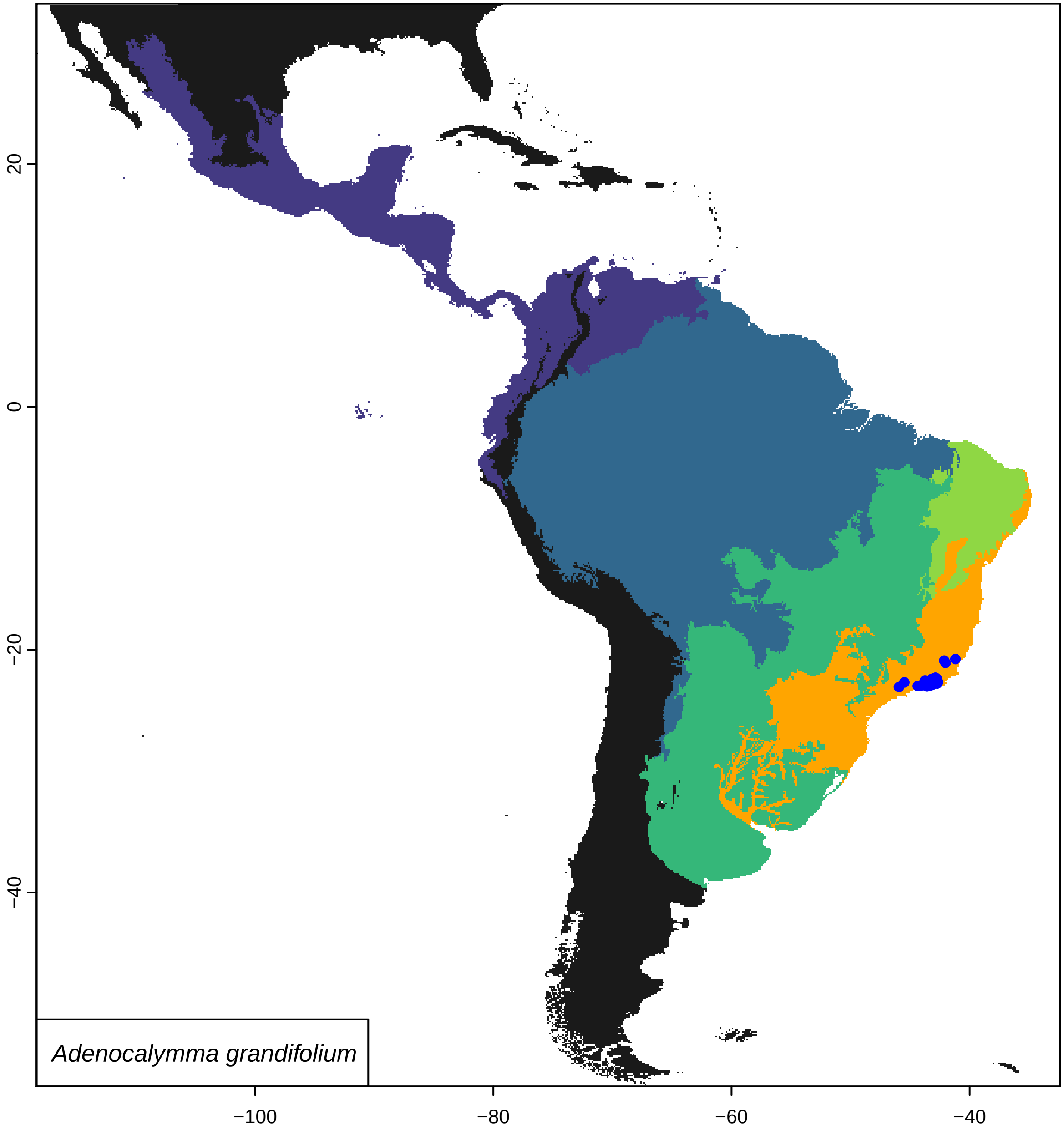

20
0
−20
−40
−100
−80
−60
−40
Adenocalymma grandifolium

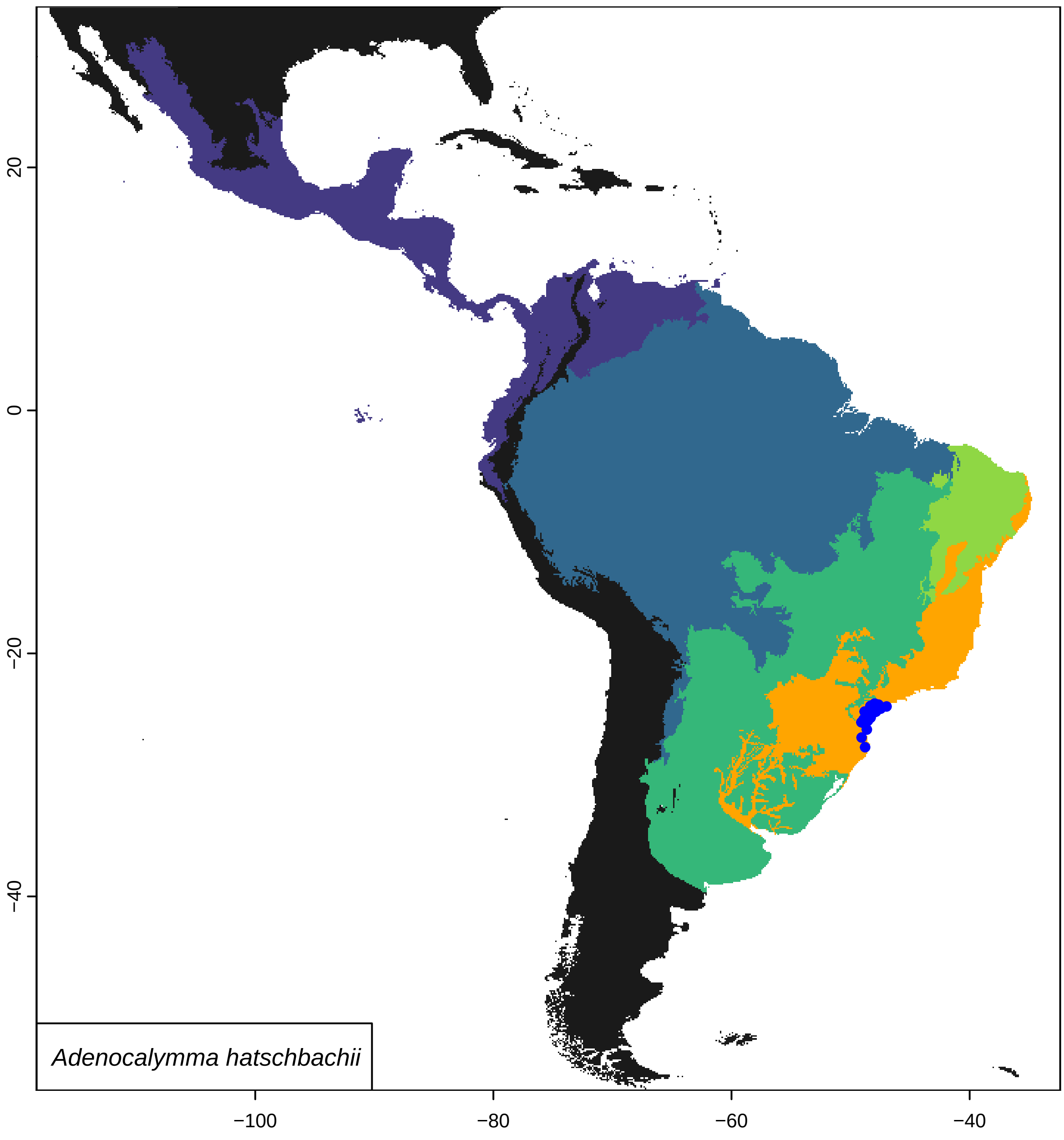

20
0
−20
−40
−100
−80
−60
−40
Adenocalymma hatschbachii

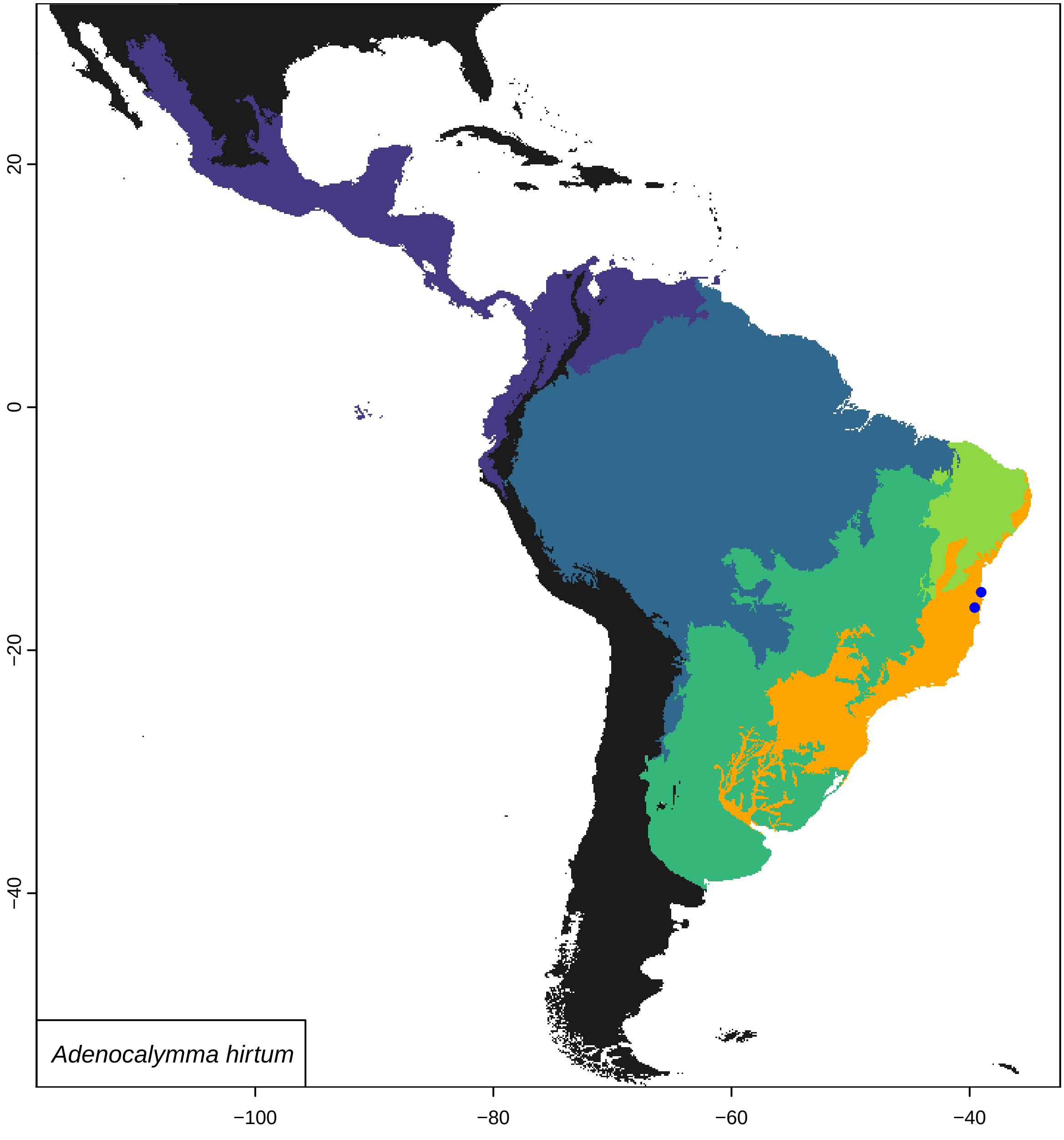
20
0
−20
−40
−100
−80
−60
−40
Adenocalymma hirtum

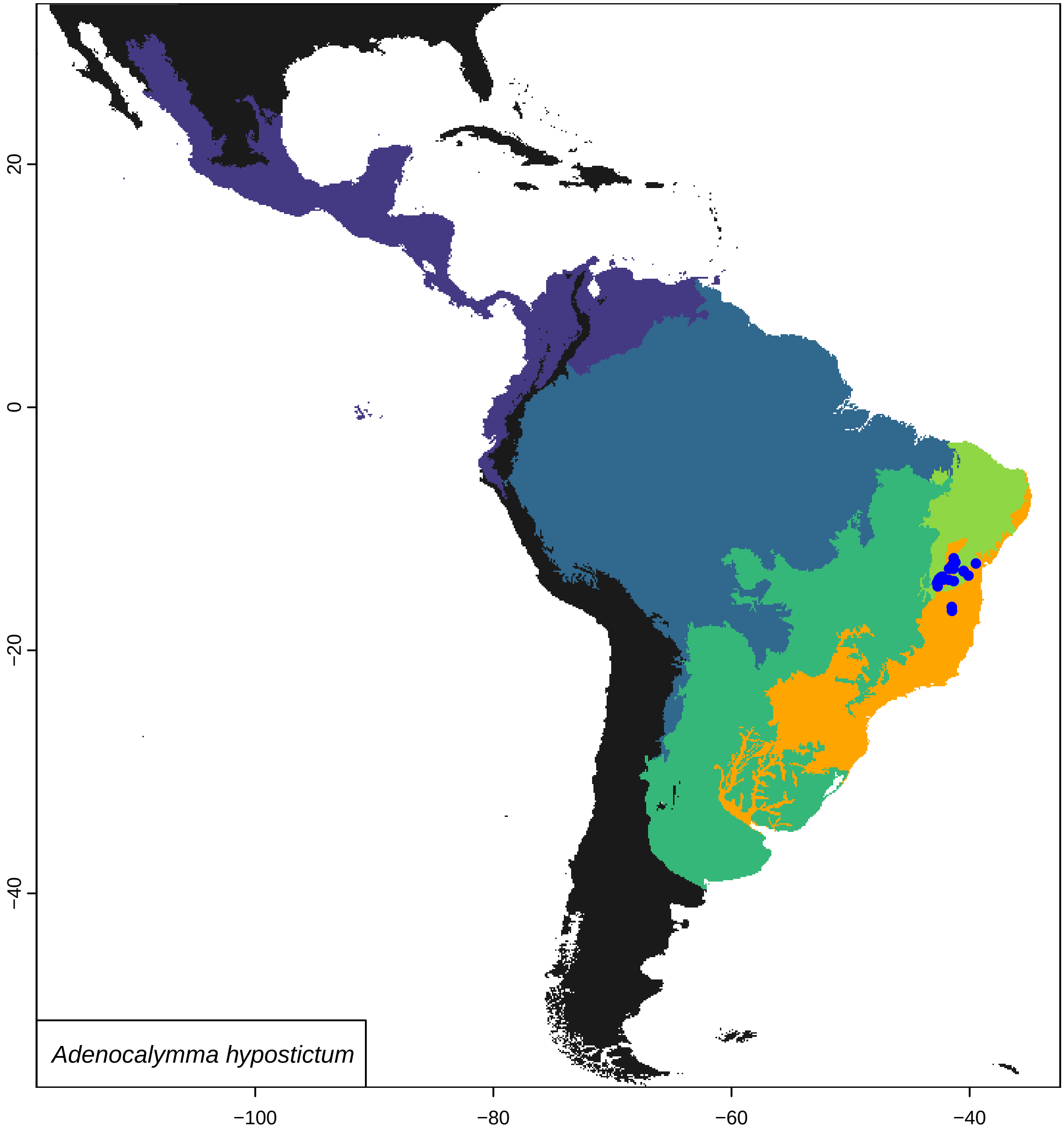

20
0
−20
−40
−100
−80
−60
−40
Adenocalymma hypostictum

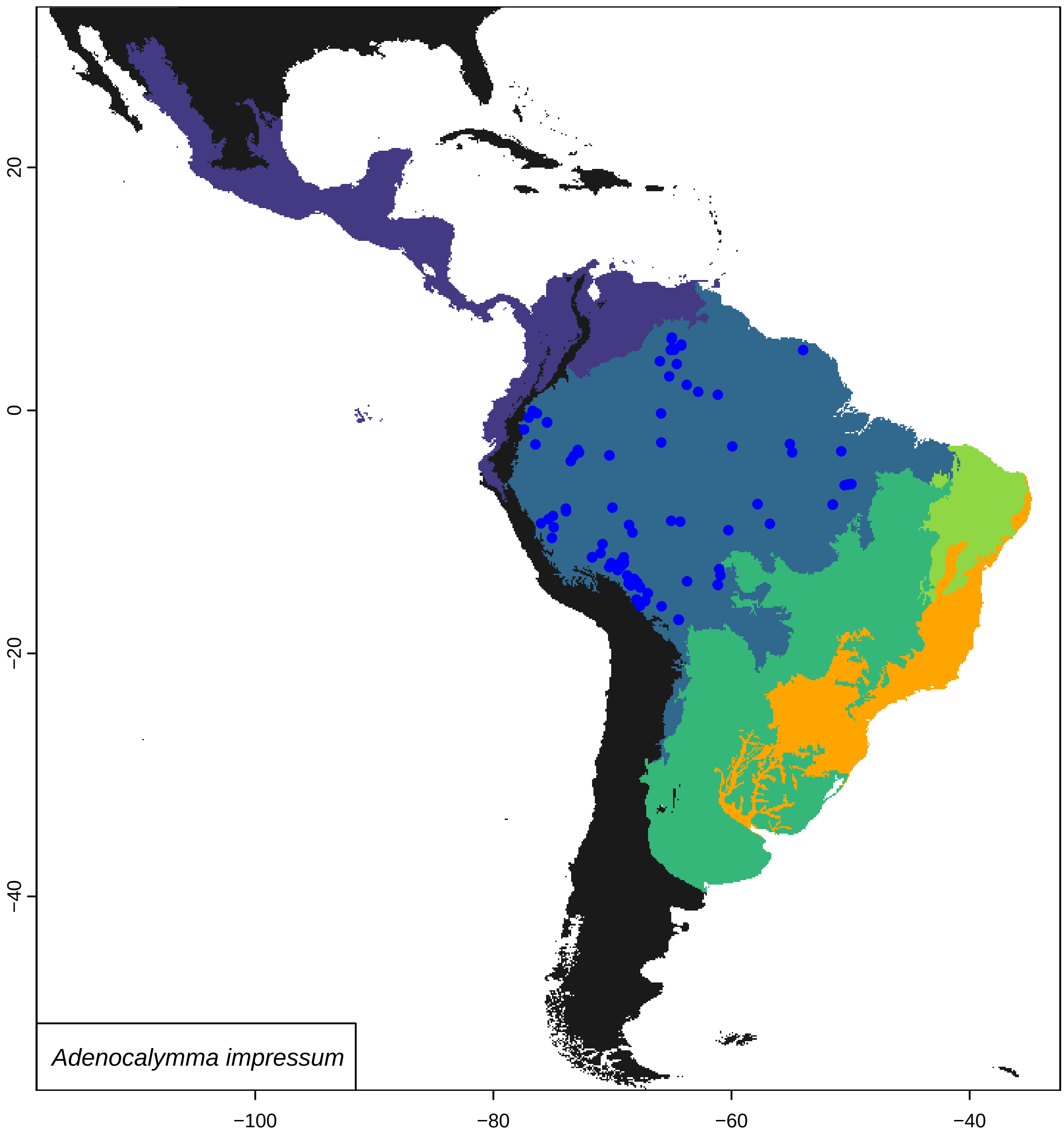

Adenocalymma impressum
20
0
−20
−40
−100
−80
−60
−40

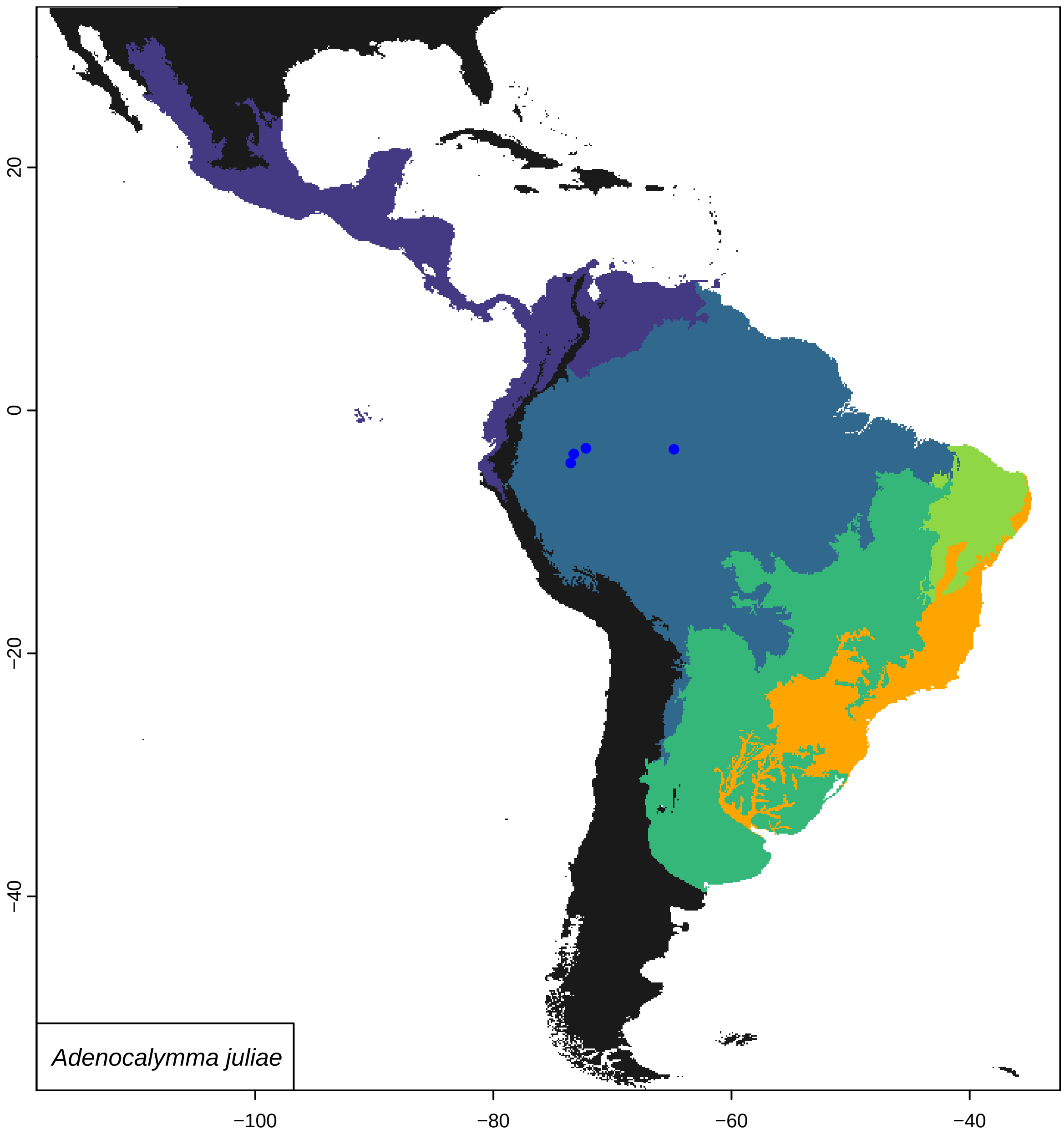

20
0
−20
−40
−100
−80
−60
−40
Adenocalymma juliae

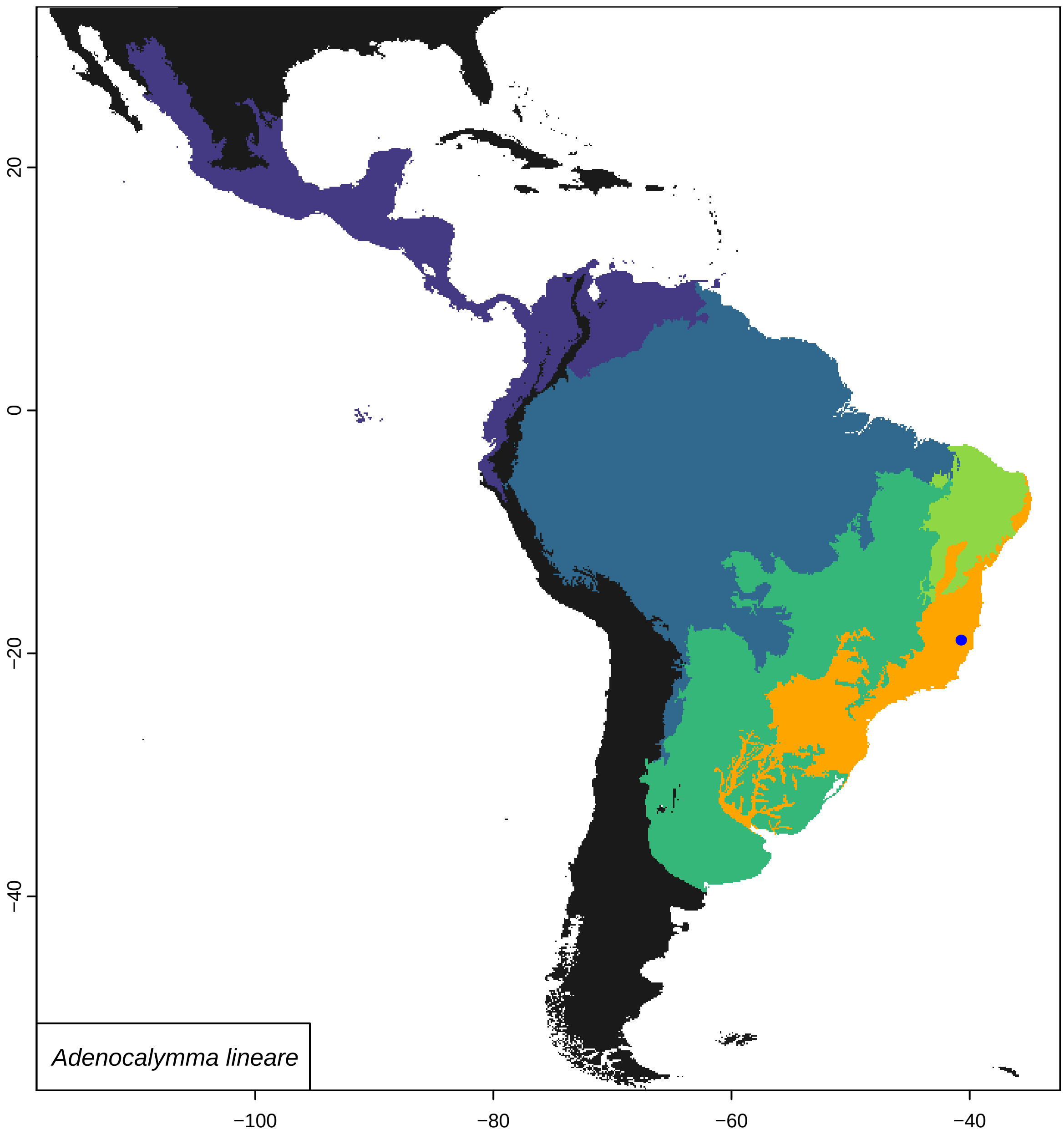
20
0
−20
−40
−100
−80
−60
−40
Adenocalymma lineare

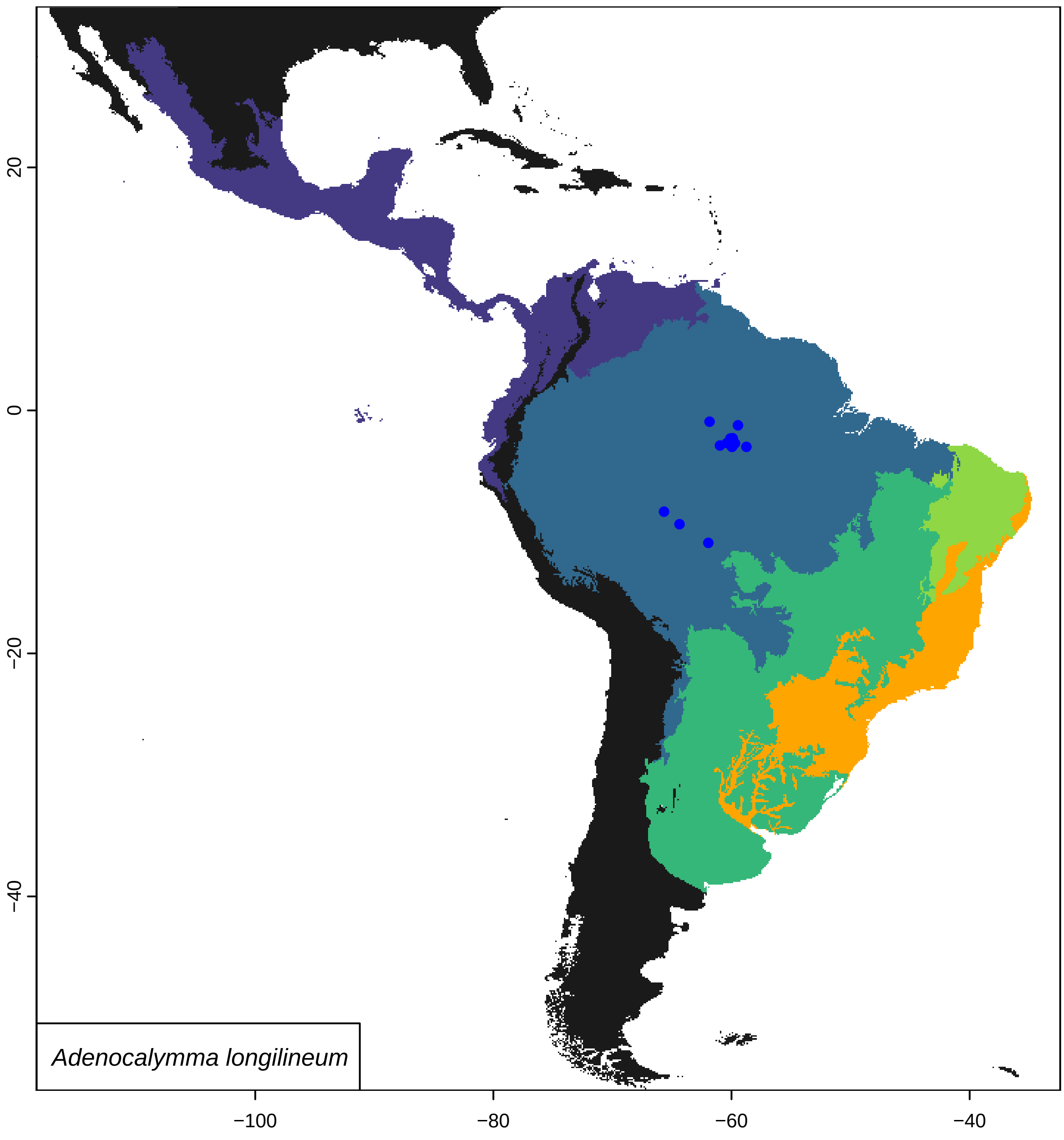

Adenocalymma longilineum
20
0
−20
−40
−100
−80
−60
−40

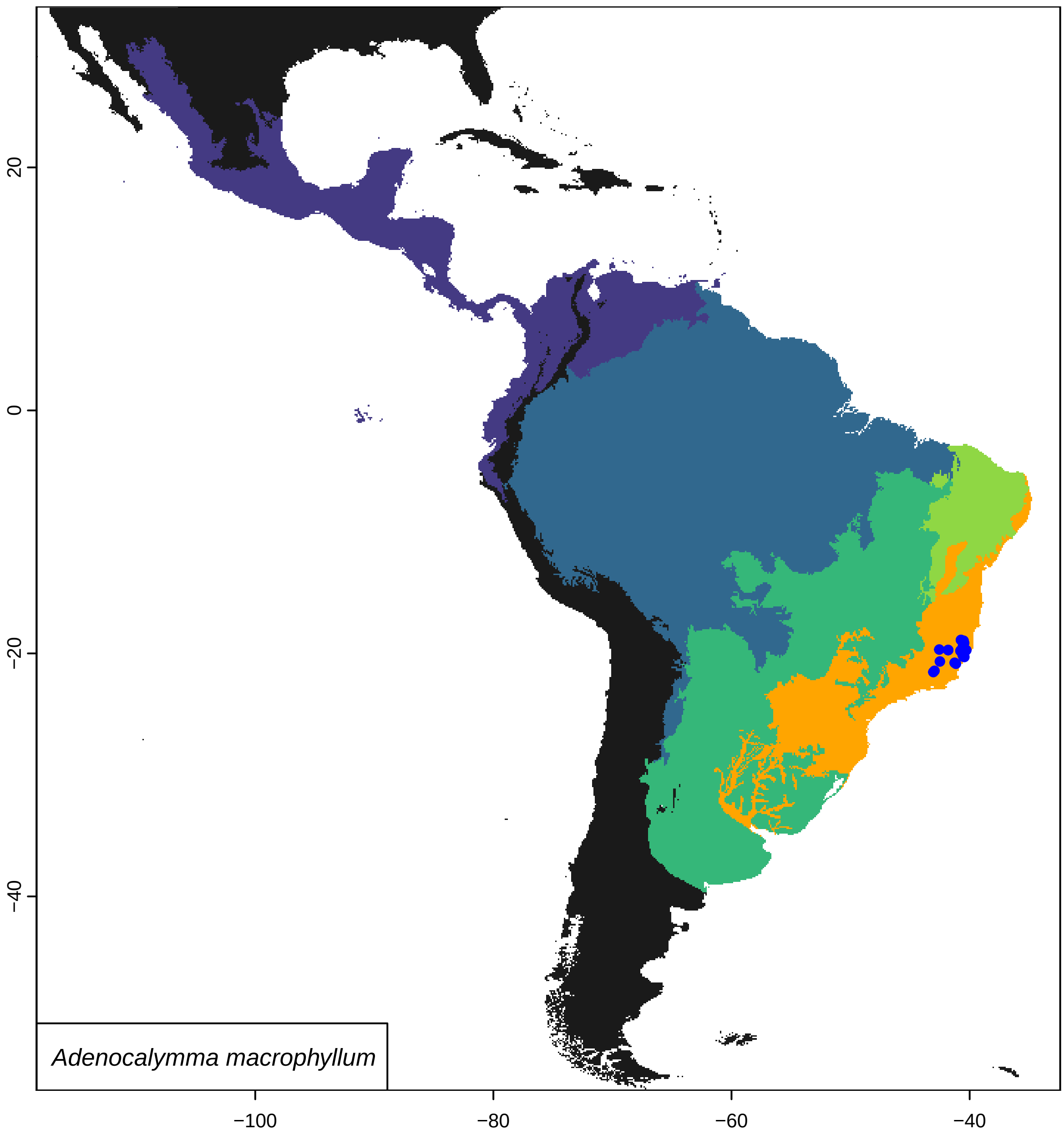
20
0
−20
−40
−100
−80
−60
−40
Adenocalymma macrophyllum

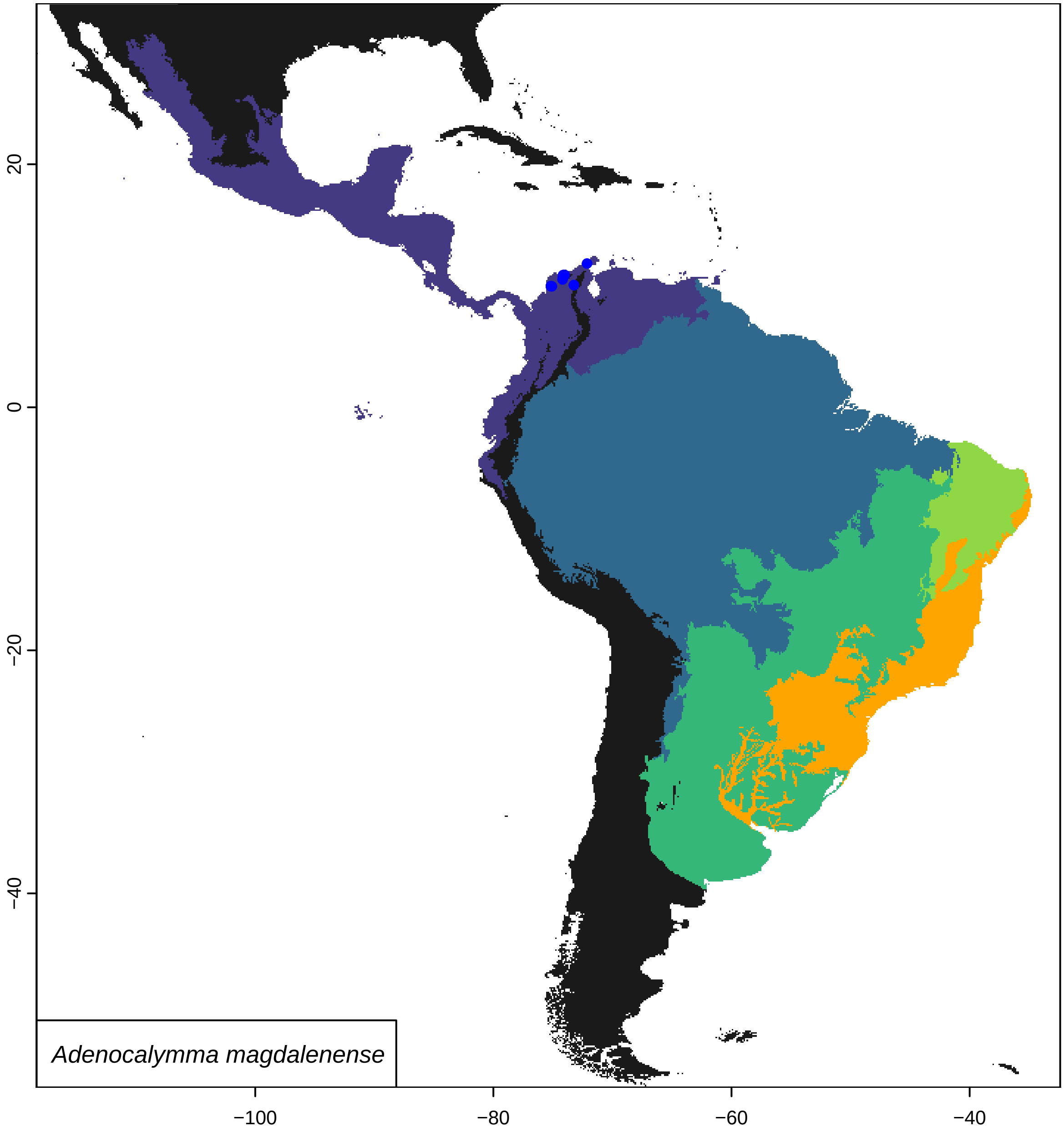
20
0
−20
−40
−100
−80
−60
−40
Adenocalymma magdalenense

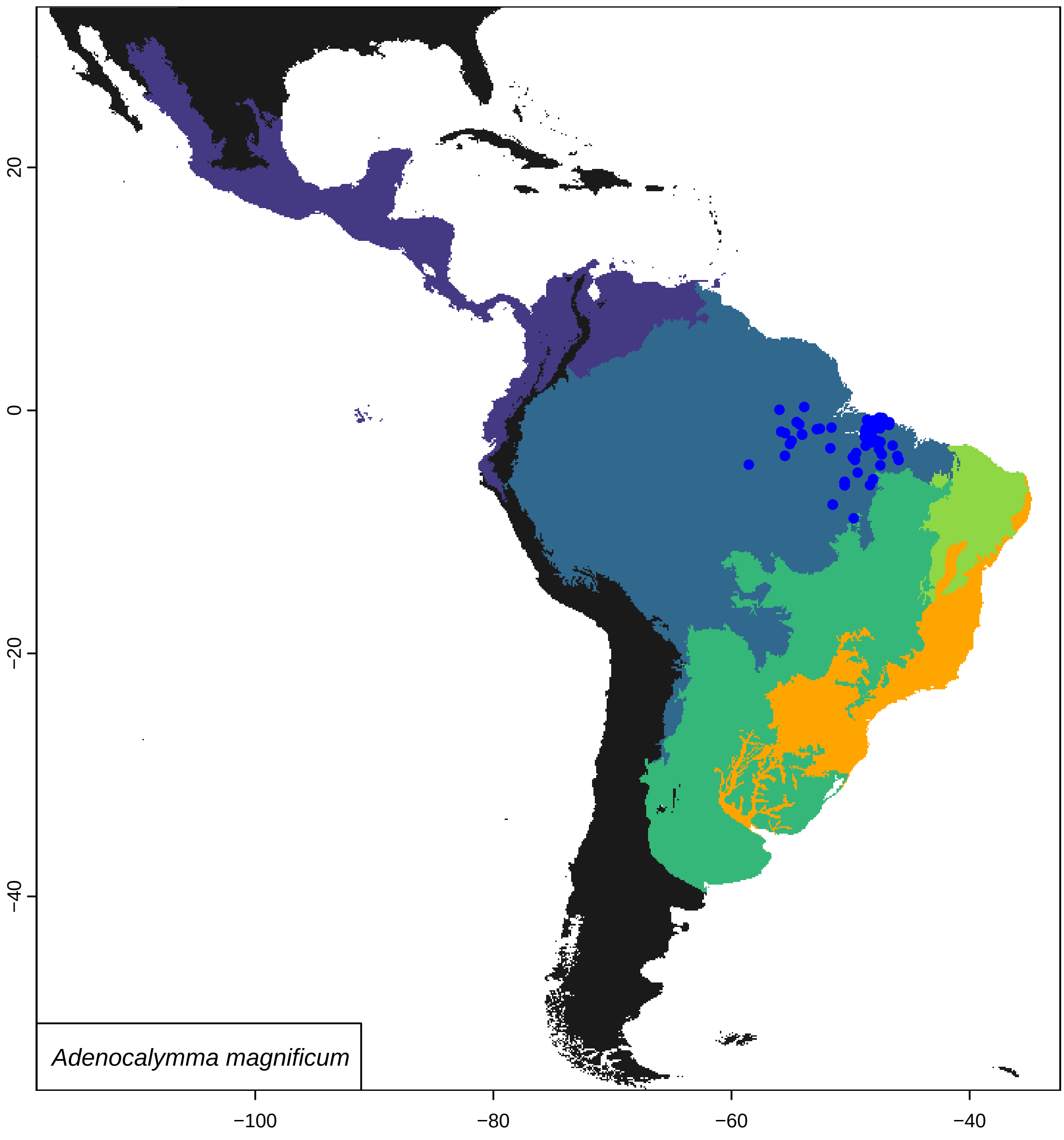

20
0
−20
−40
−100
−80
−60
−40
Adenocalymma magnificum

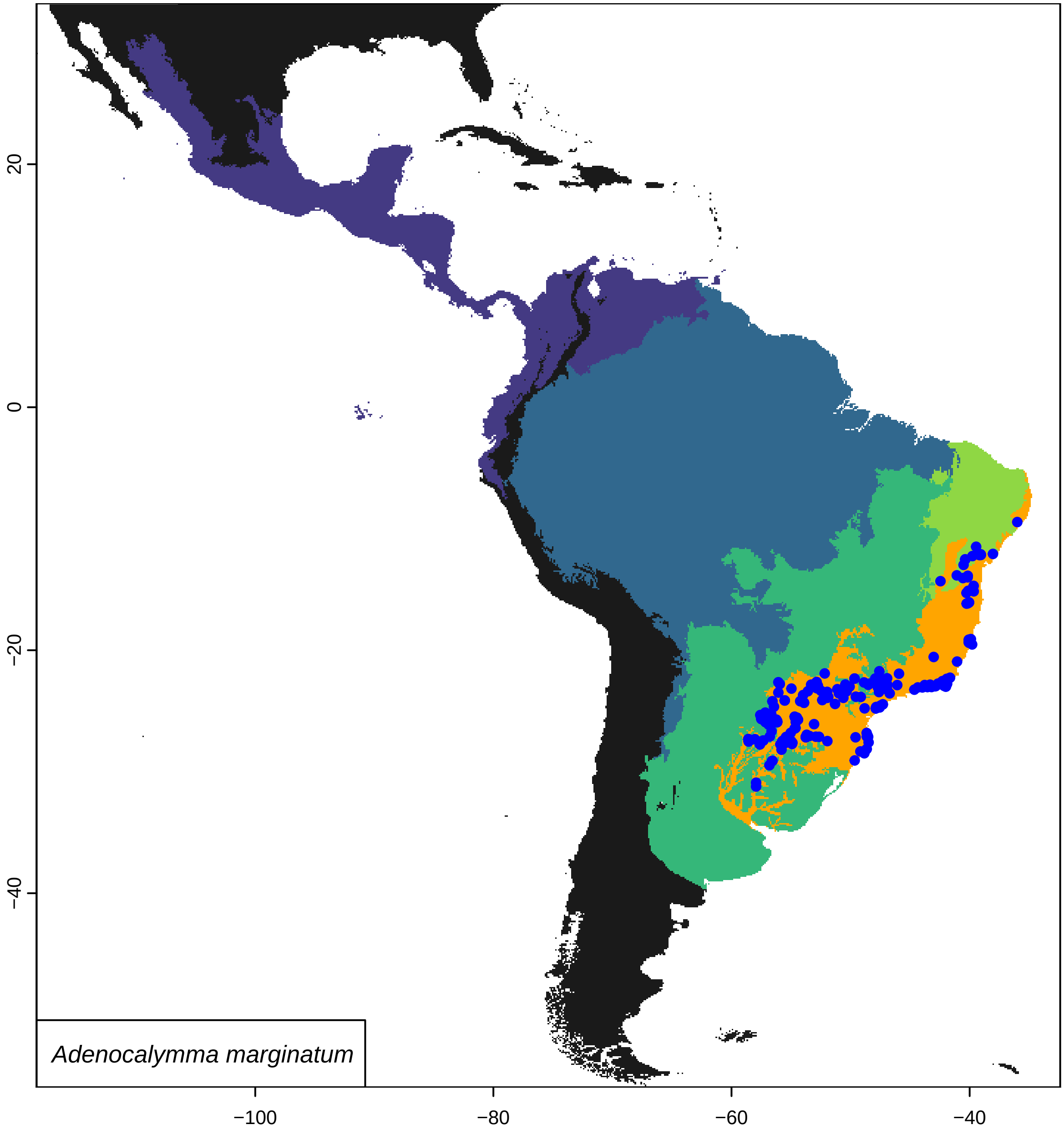
20
0
−20
−40
−100
−80
−60
−40
Adenocalymma marginatum

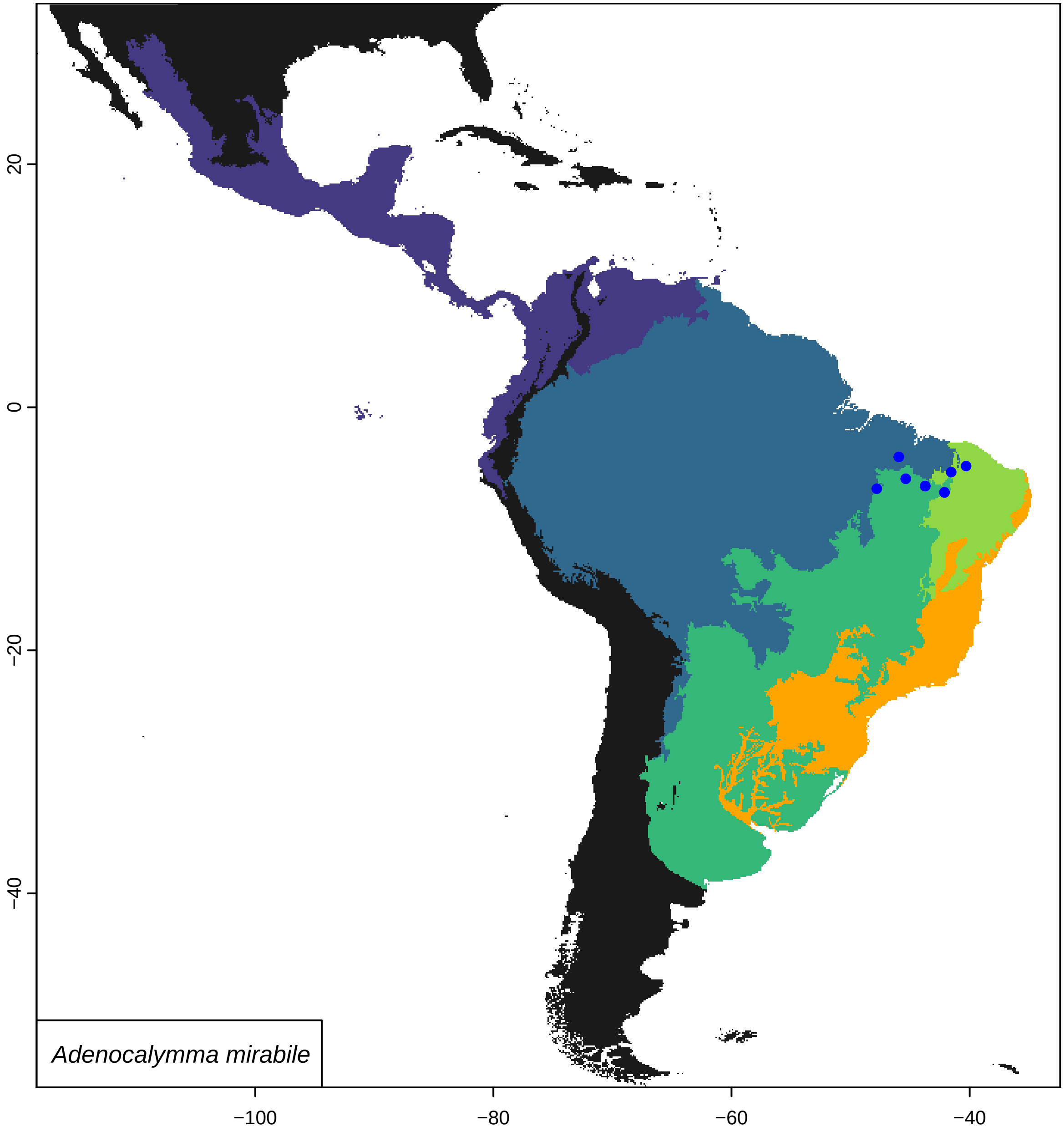

20
0
−20
−40
−100
−80
−60
−40
Adenocalymma mirabile

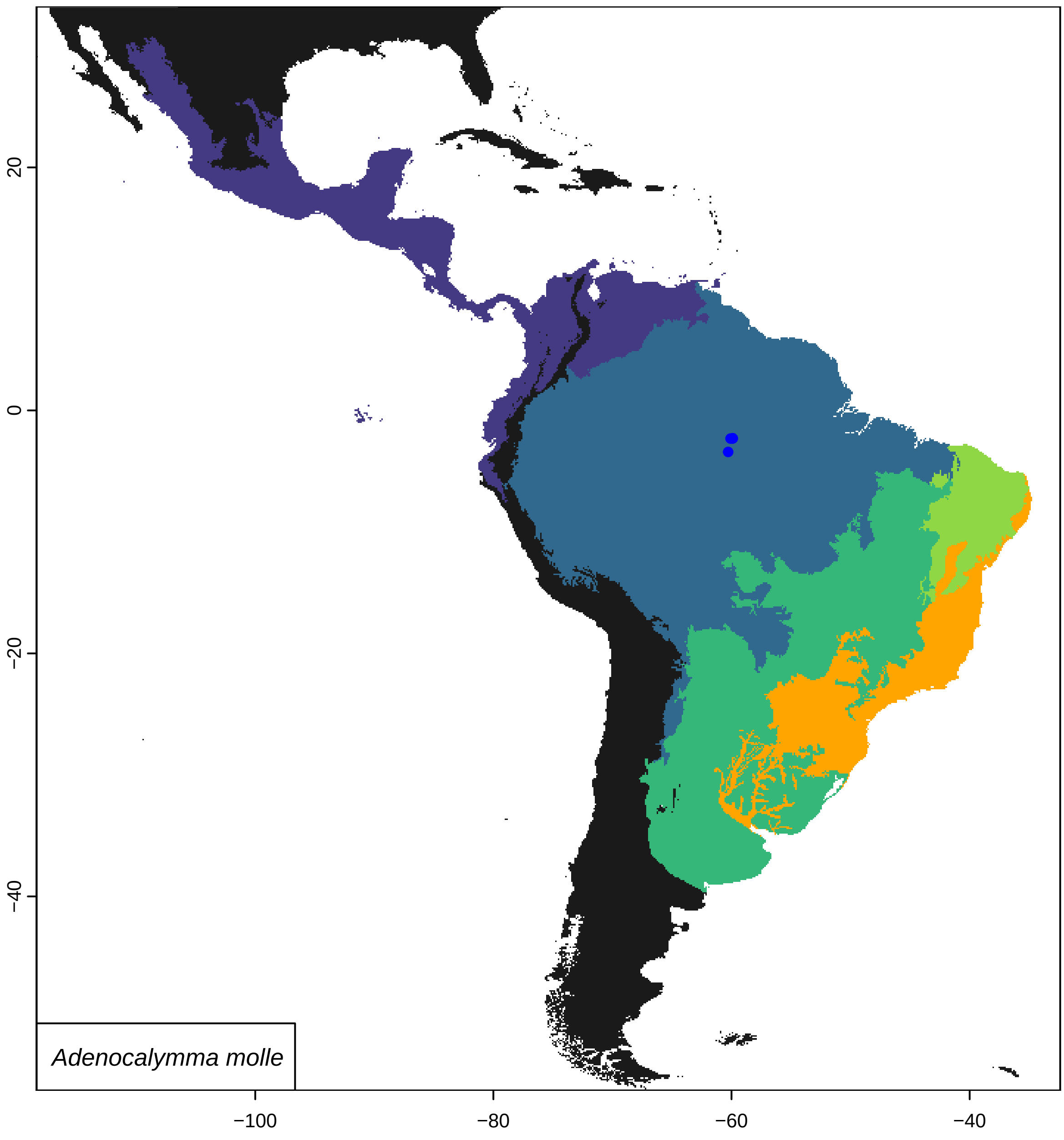
20
0
−20
−40
−100
−80
−60
−40
Adenocalymma molle

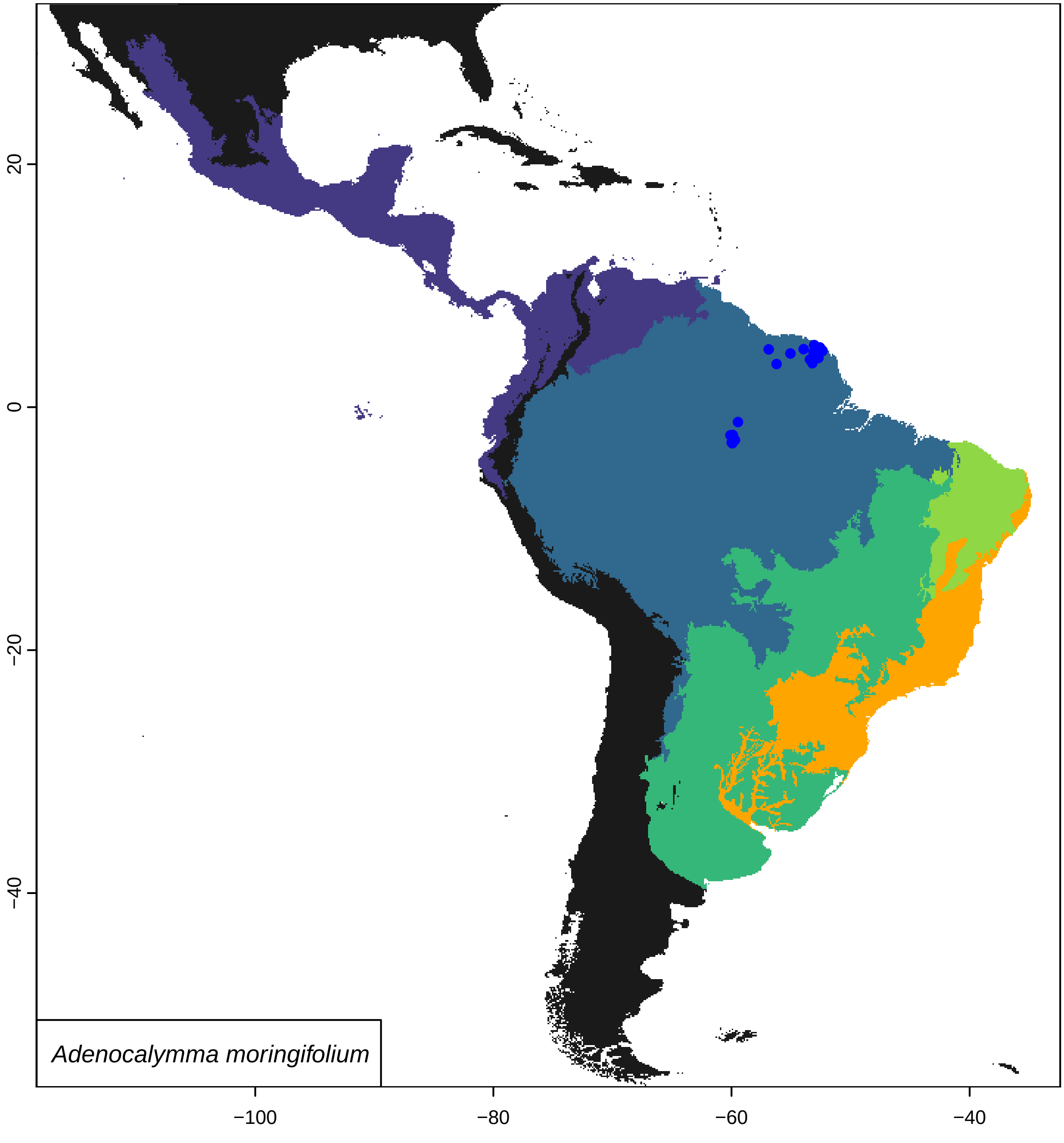

20
0
−20
−40
−100
−80
−60
−40
Adenocalymma moringifolium

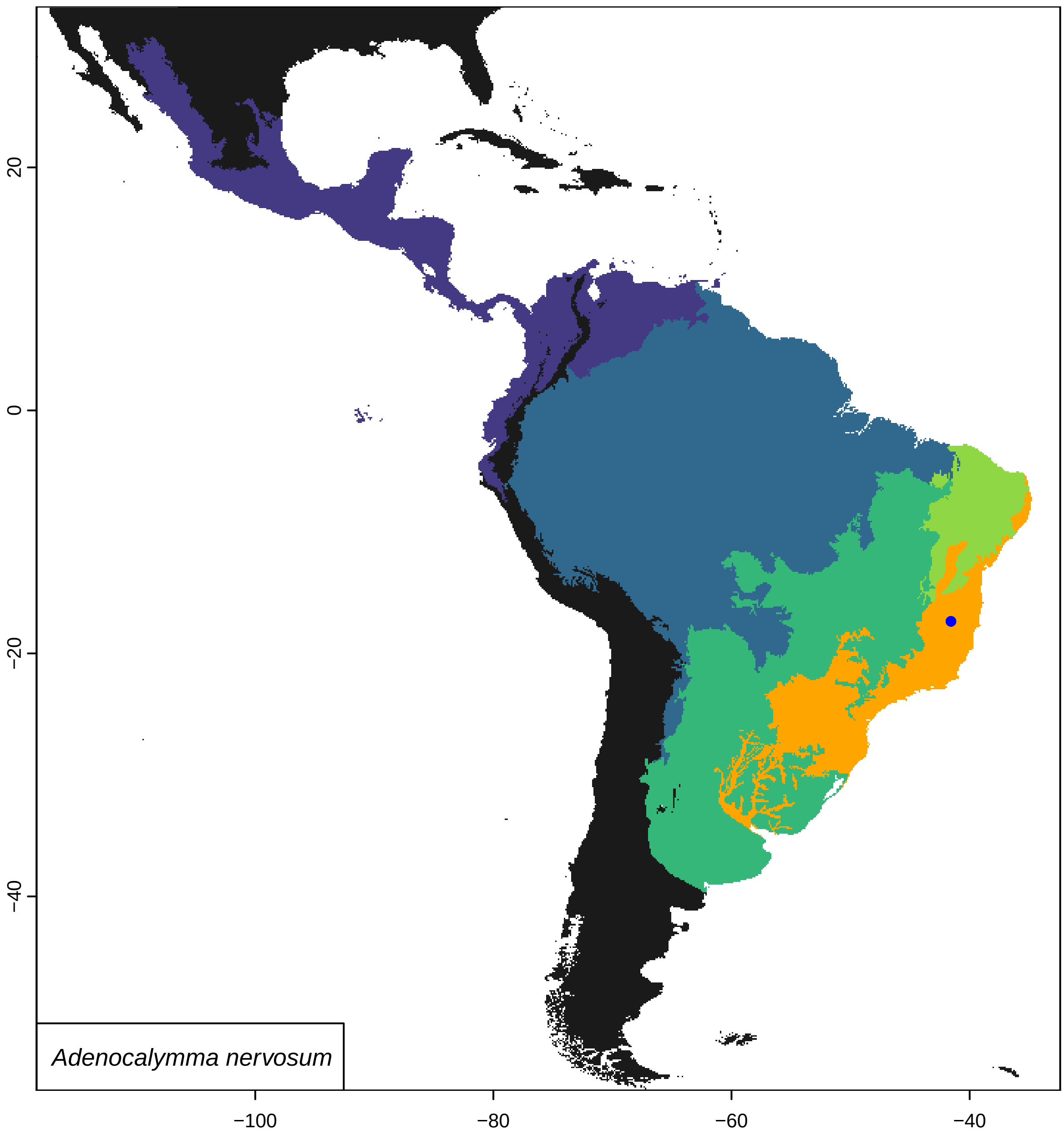

20
0
−20
−40
−100
−80
−60
−40
Adenocalymma nervosum

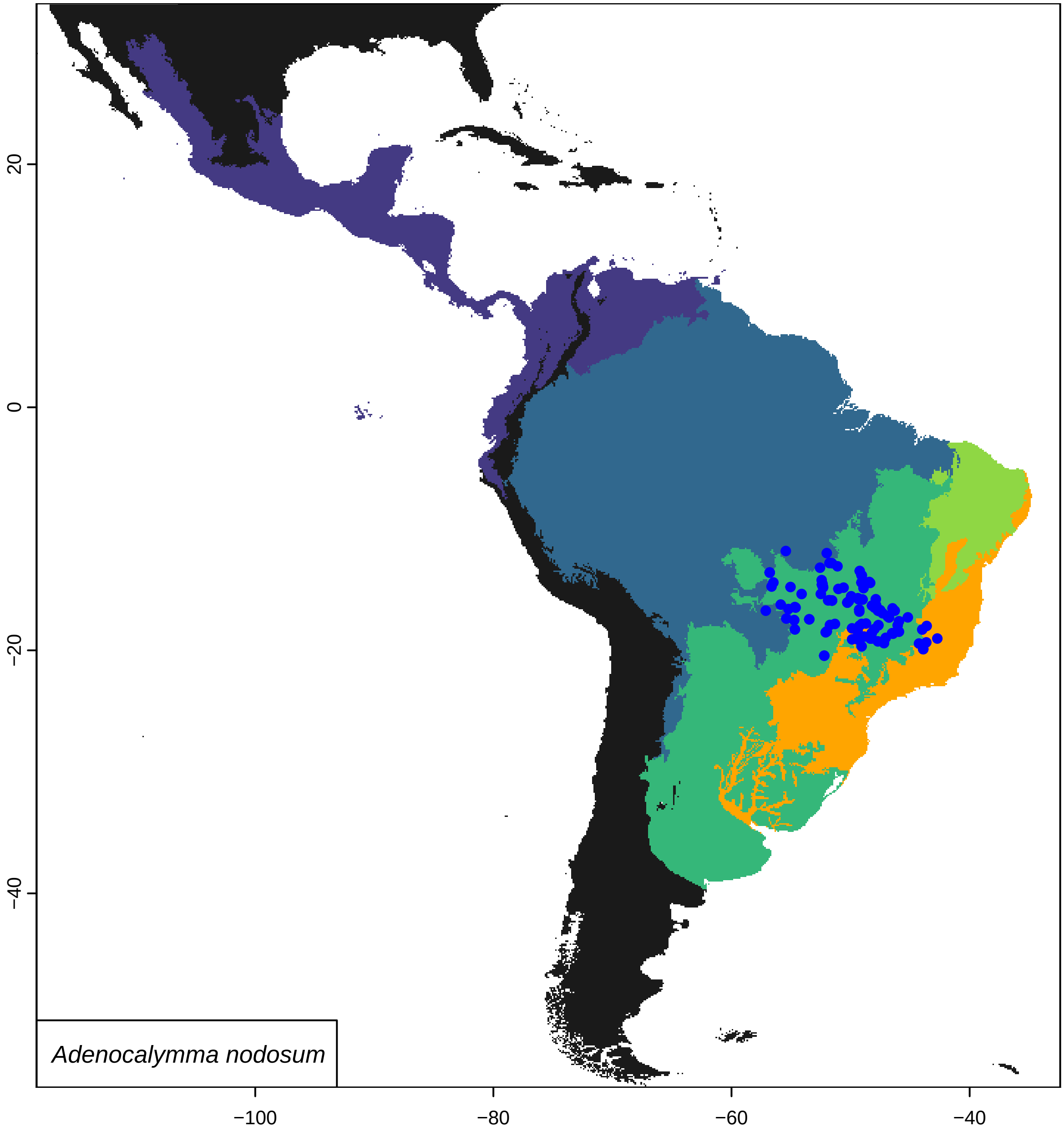

Adenocalymma nodosum
20
0
−20
−40
−100
−80
−60
−40

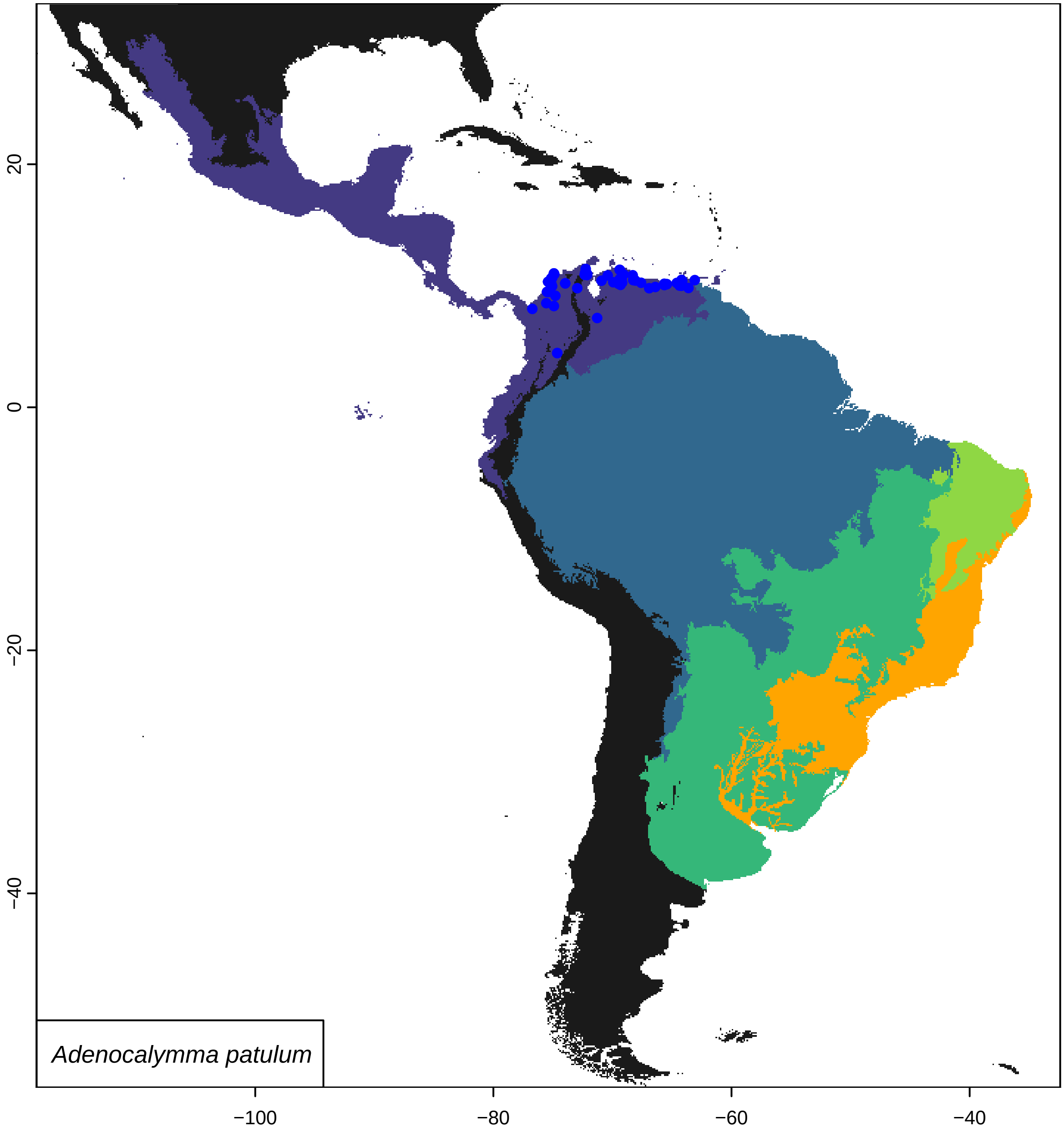

20
0
−20
−40
−100
−80
−60
−40
Adenocalymma patulum

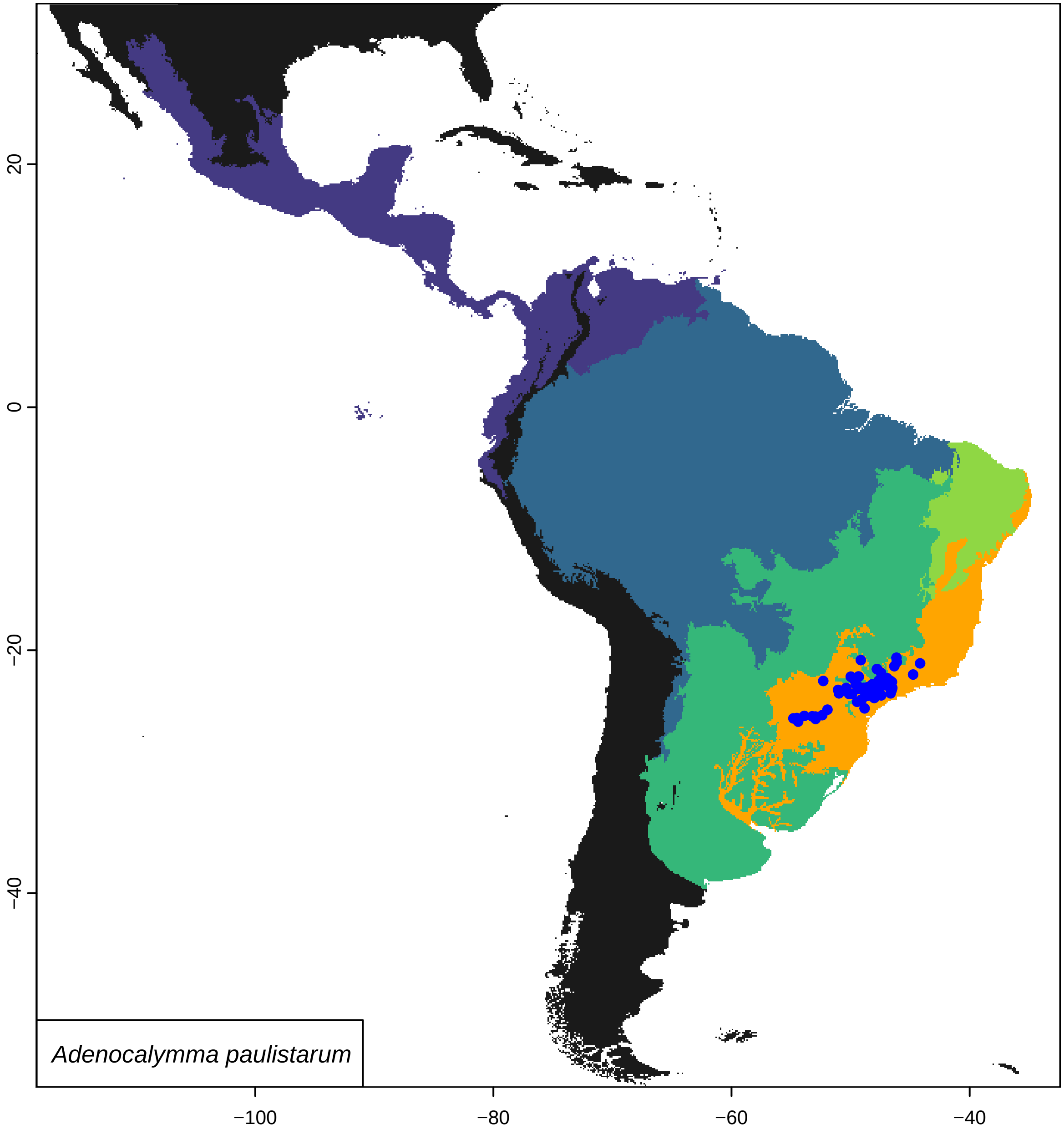
20
0
−20
−40
−100
−80
−60
−40
Adenocalymma paulistarum

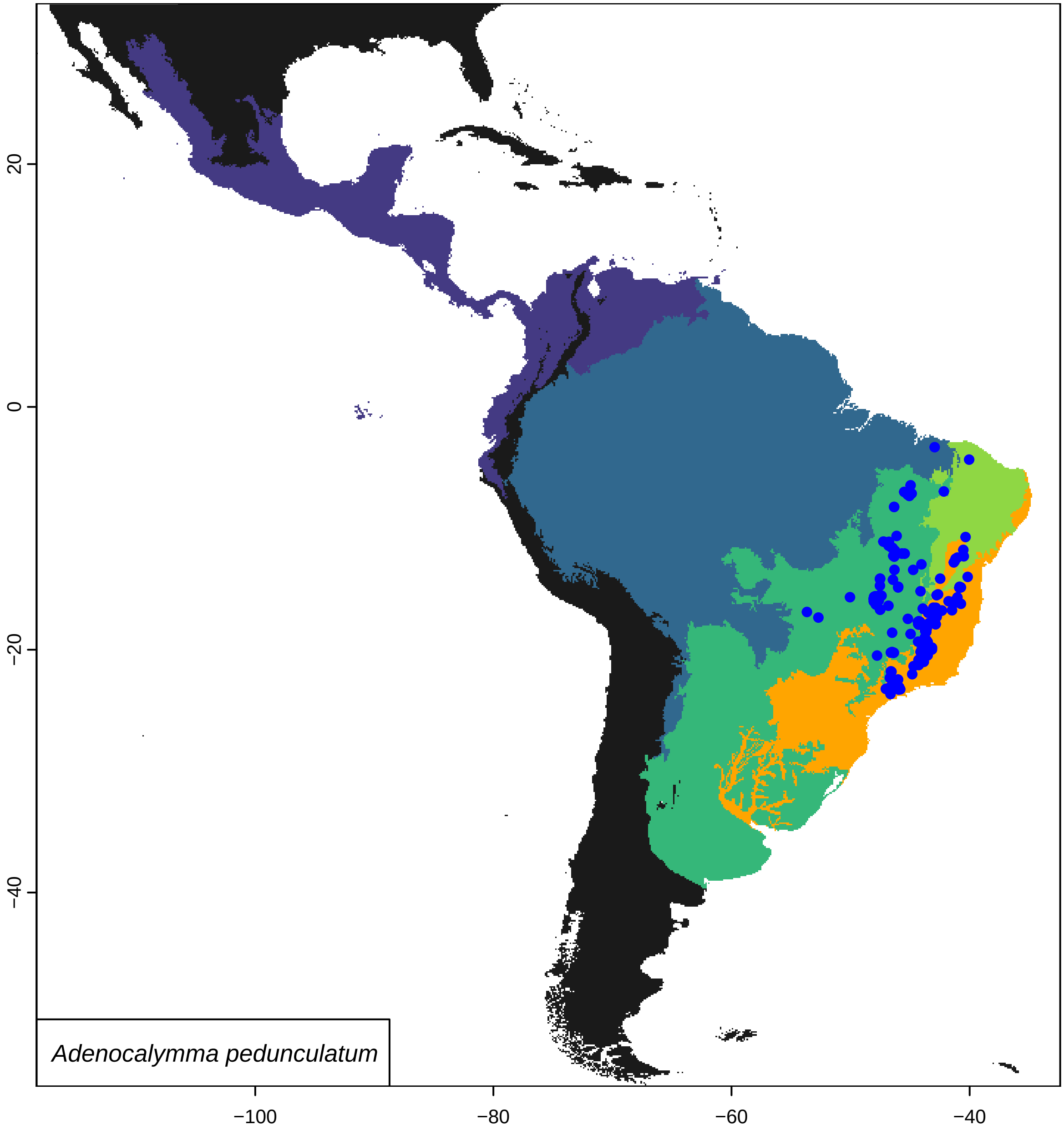
20
0
−20
−40
−100
−80
−60
−40
Adenocalymma pedunculatum

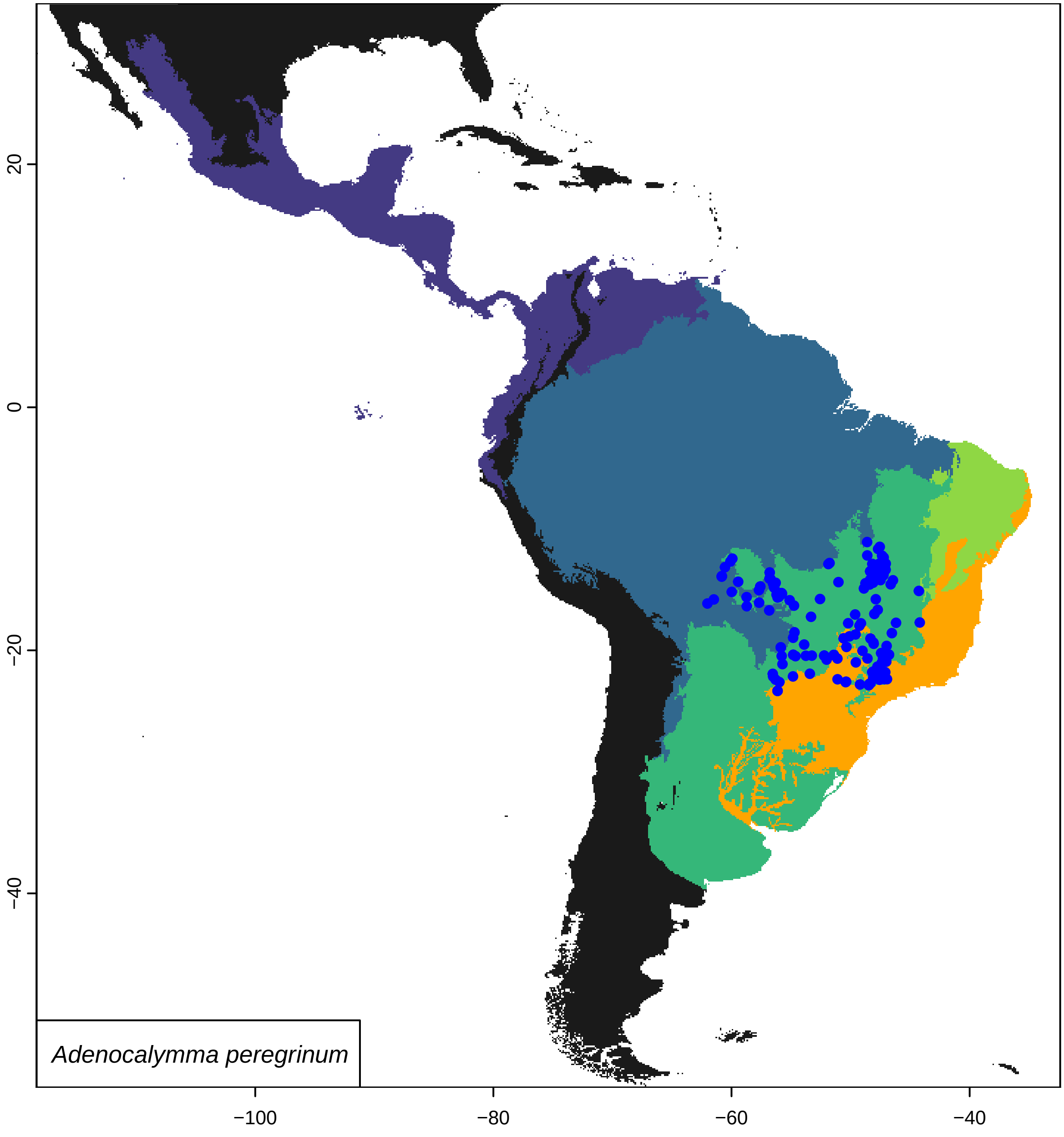

20
0
−20
−40
−100
−80
−60
−40
Adenocalymma peregrinum

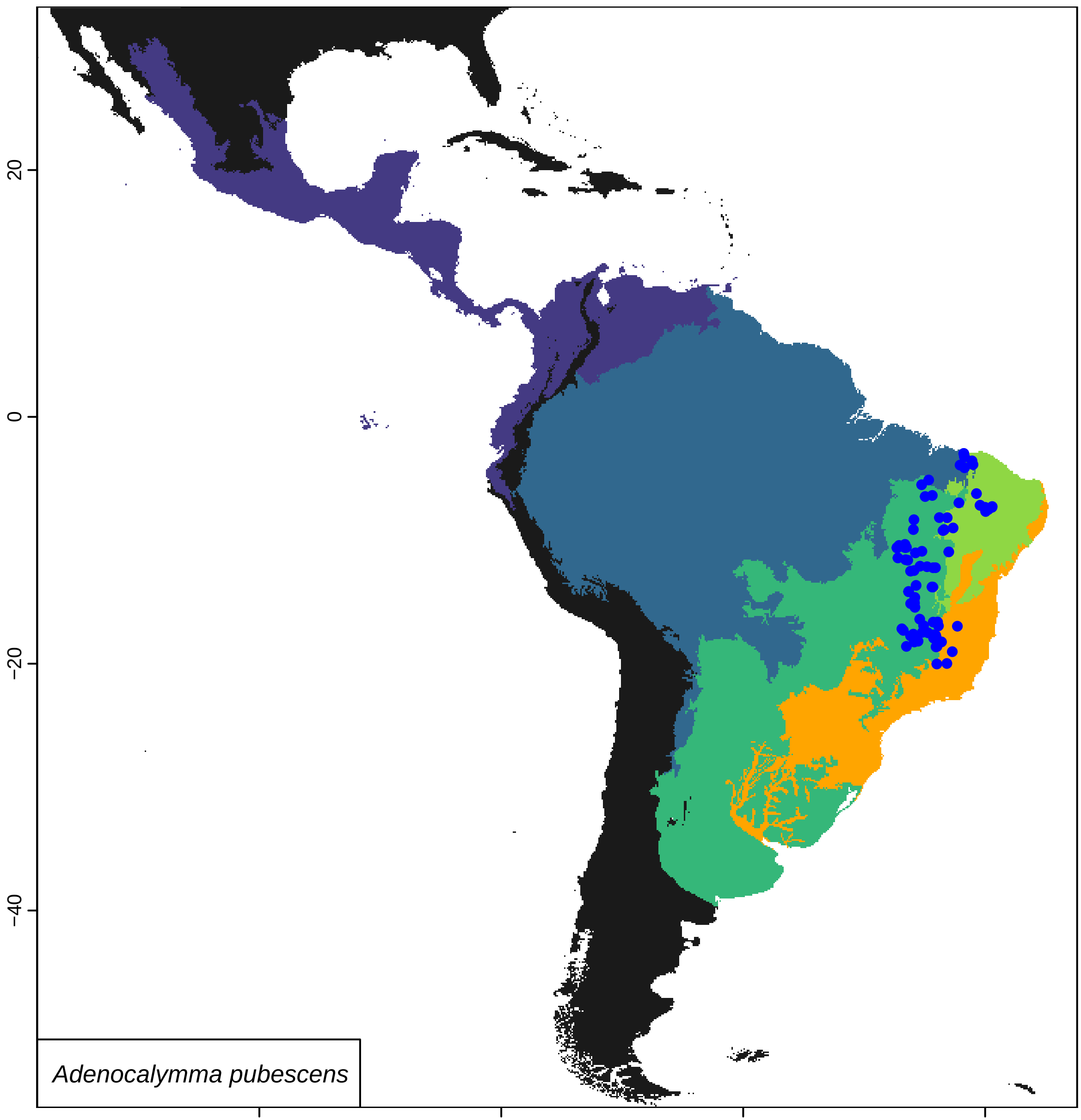
20
0
−20
−40
−100
−80
−60
−40
Adenocalymma pubescens

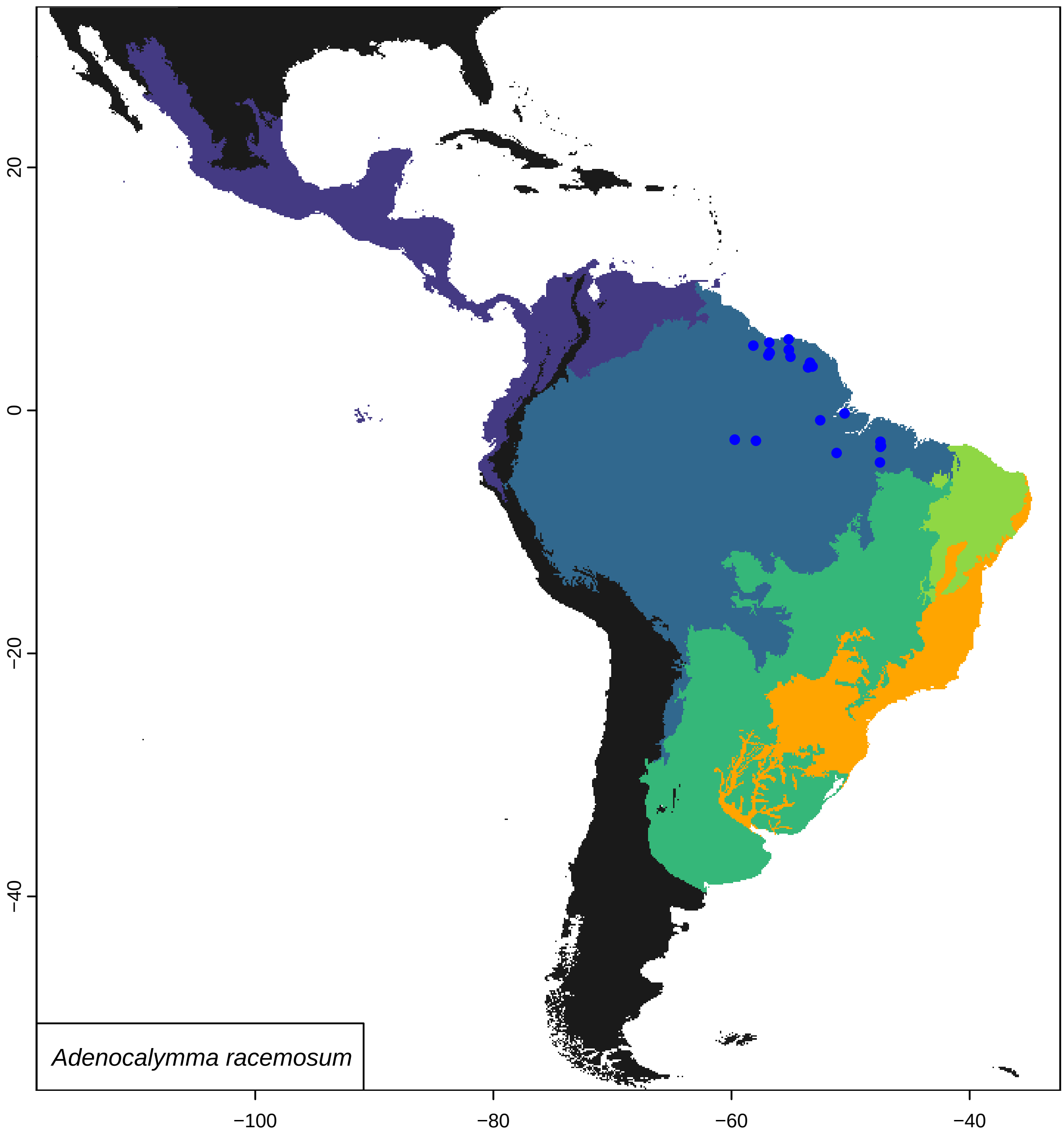
Adenocalymma racemosum
20
0
−20
−40
−100
−80
−60
−40

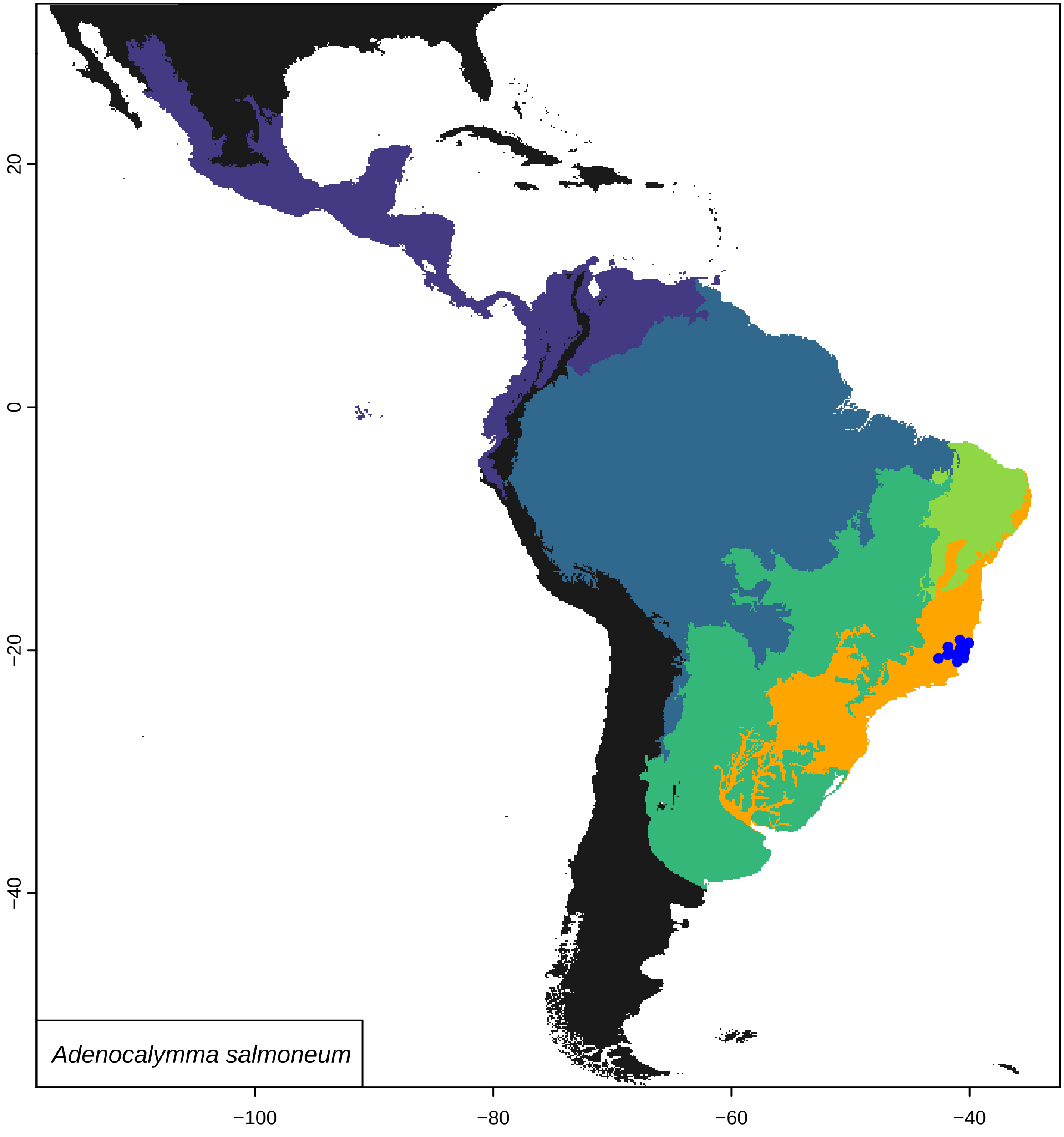

20
0
−20
−40
−100
−80
−60
−40
Adenocalymma salmoneum

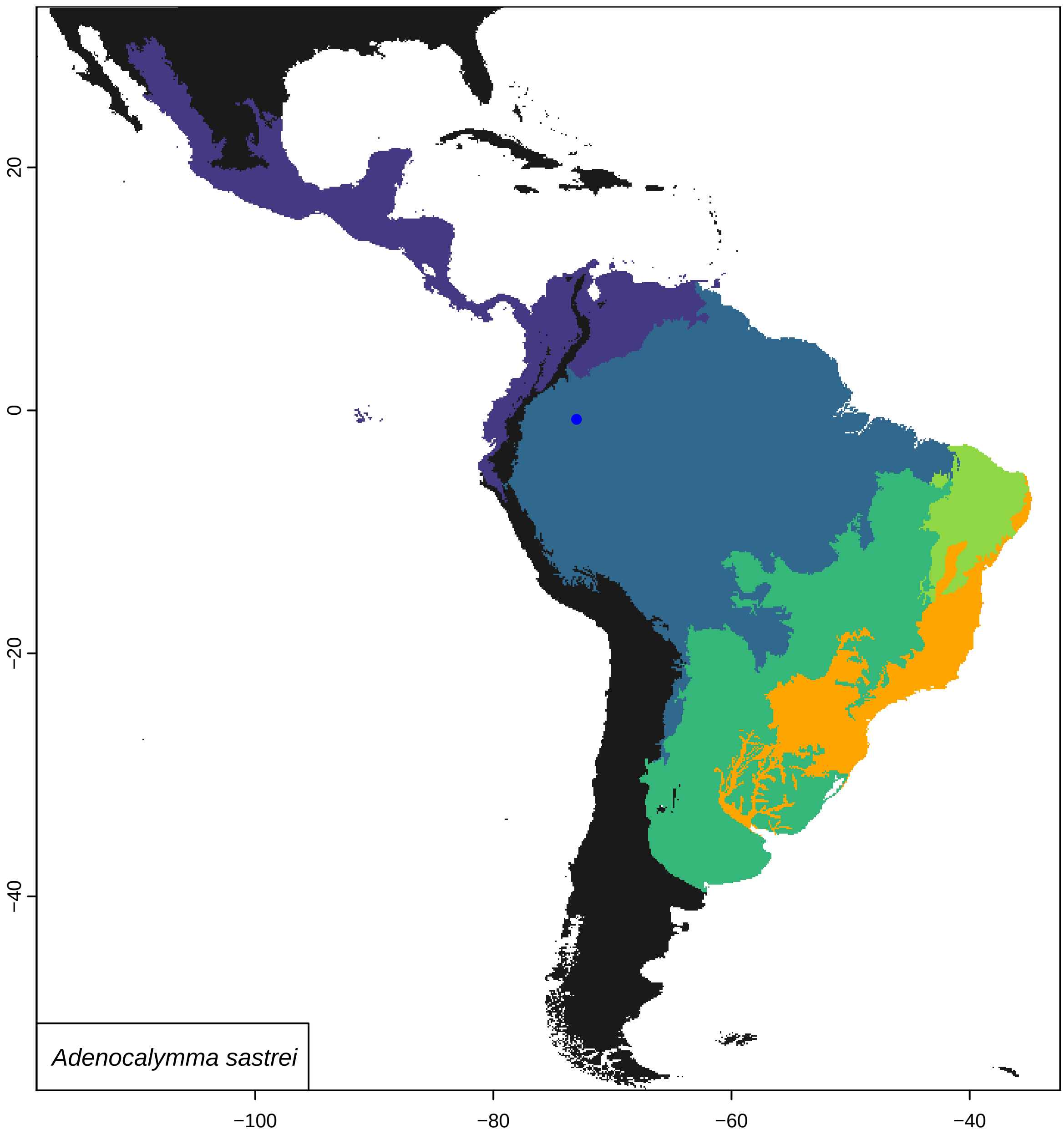

20
0
−20
−40
−100
−80
−60
−40
Adenocalymma sastrei

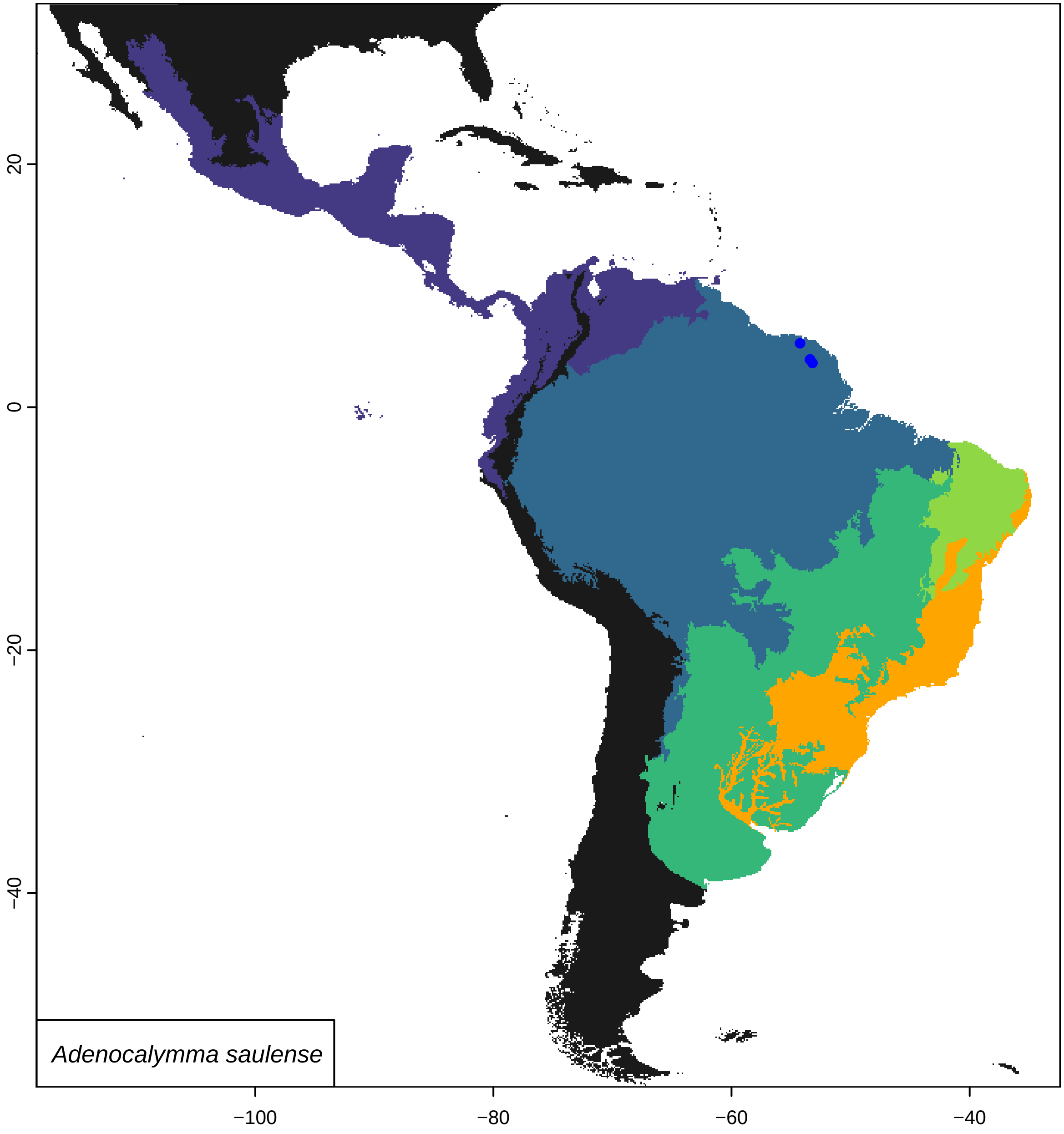

20
0
−20
−40
−100
−80
−60
−40
Adenocalymma saulense

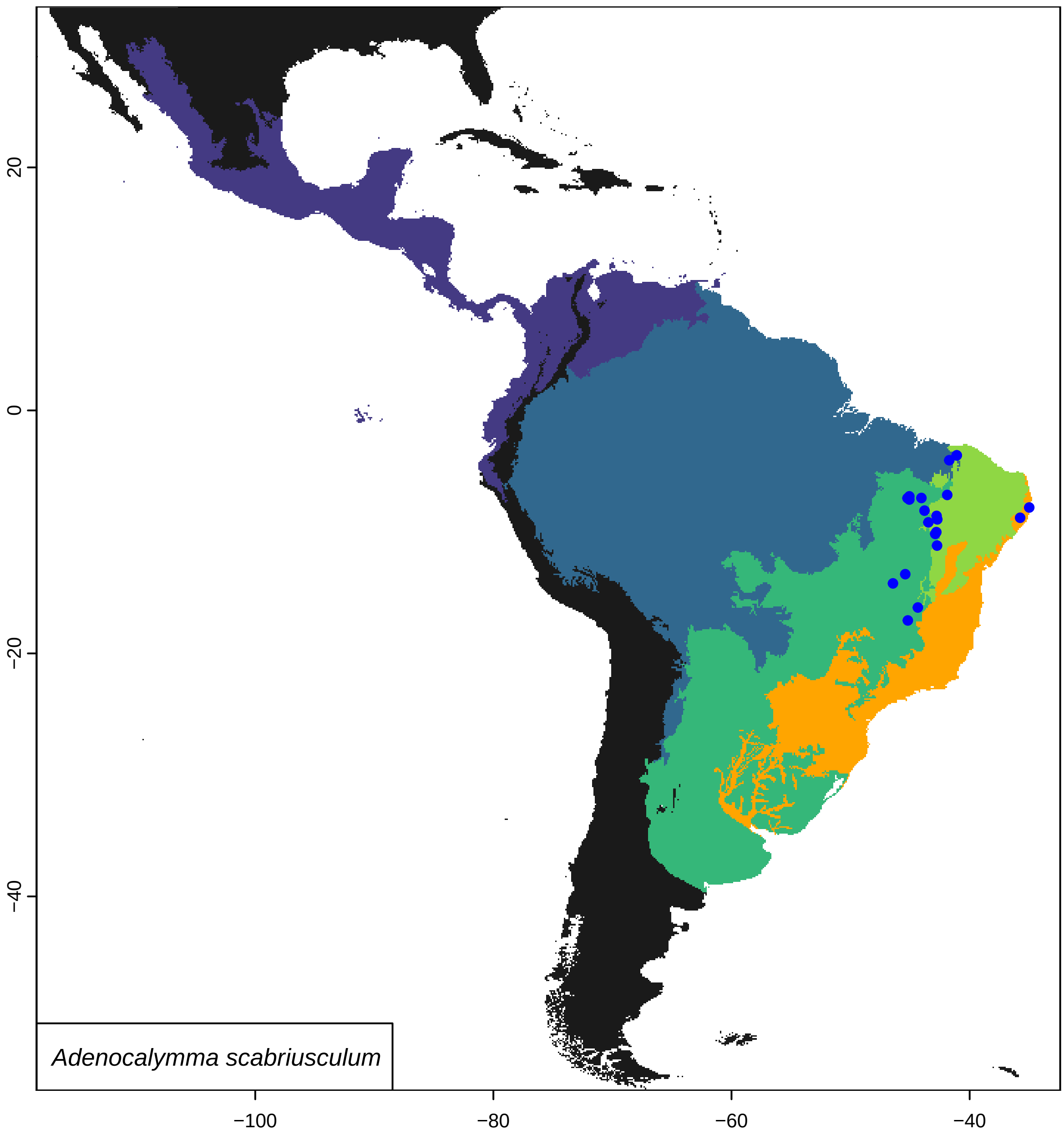

Adenocalymma scabriusculum
20
0
−20
−40
−100
−80
−60
−40

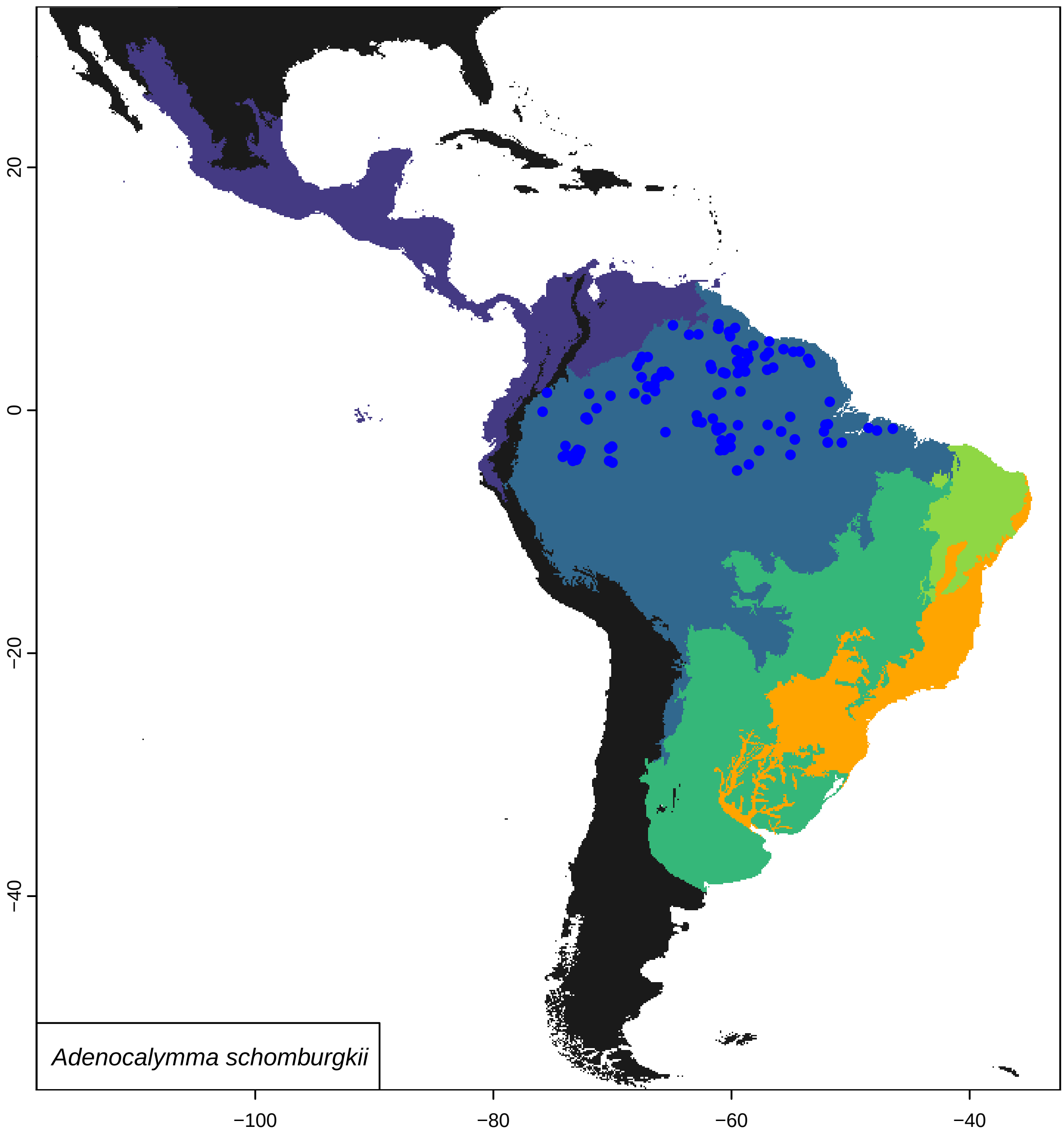
Adenocalymma schomburgkii
20
0
−20
−40
−100
−80
−60
−40

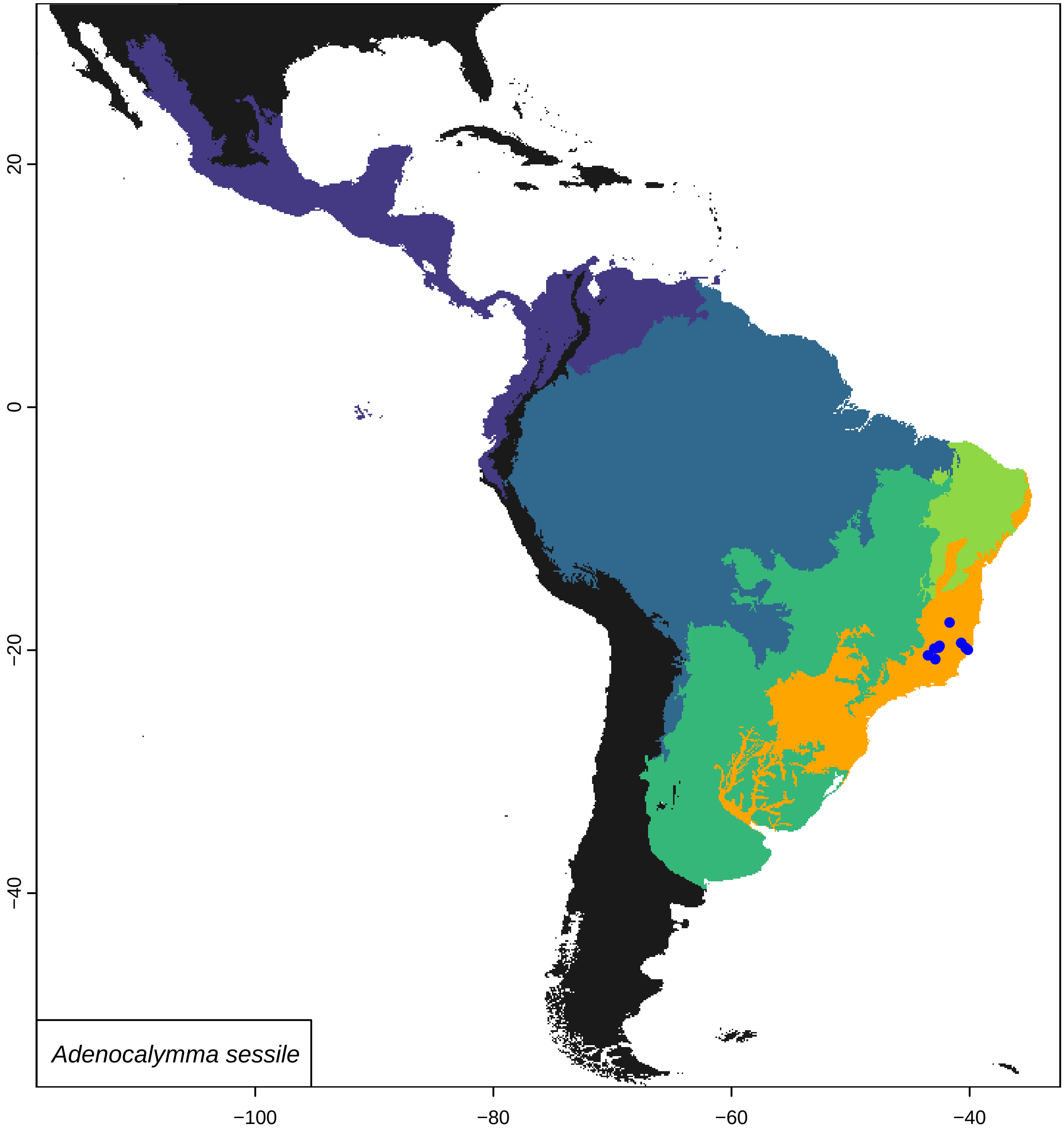
20
0
−20
−40
−100
−80
−60
−40
Adenocalymma sessile

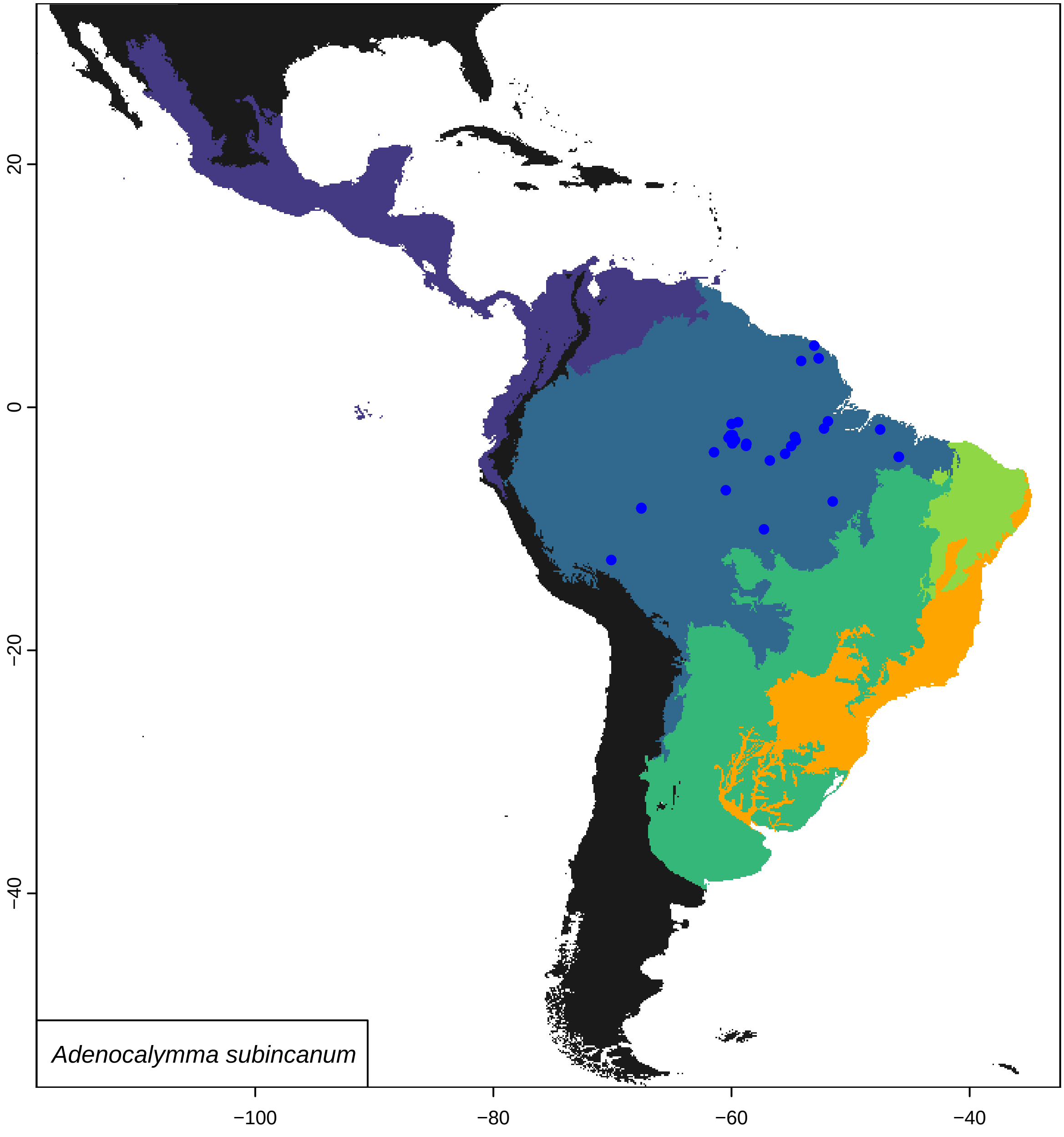
20
0
−20
−40
−100
−80
−60
−40
Adenocalymma subincanum

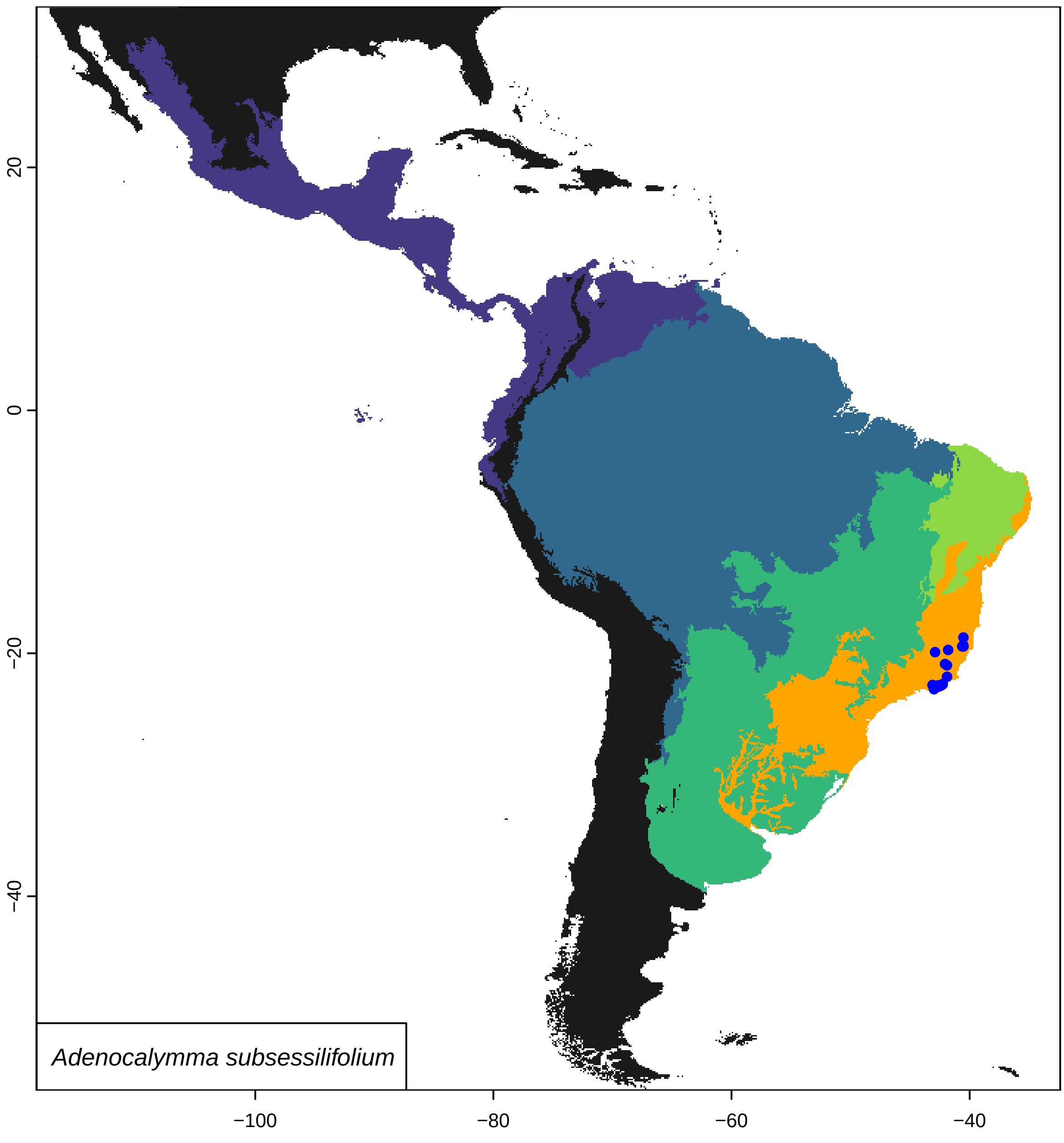

20
0
−20
−40
−100
−80
−60
−40
Adenocalymma subsessilifolium

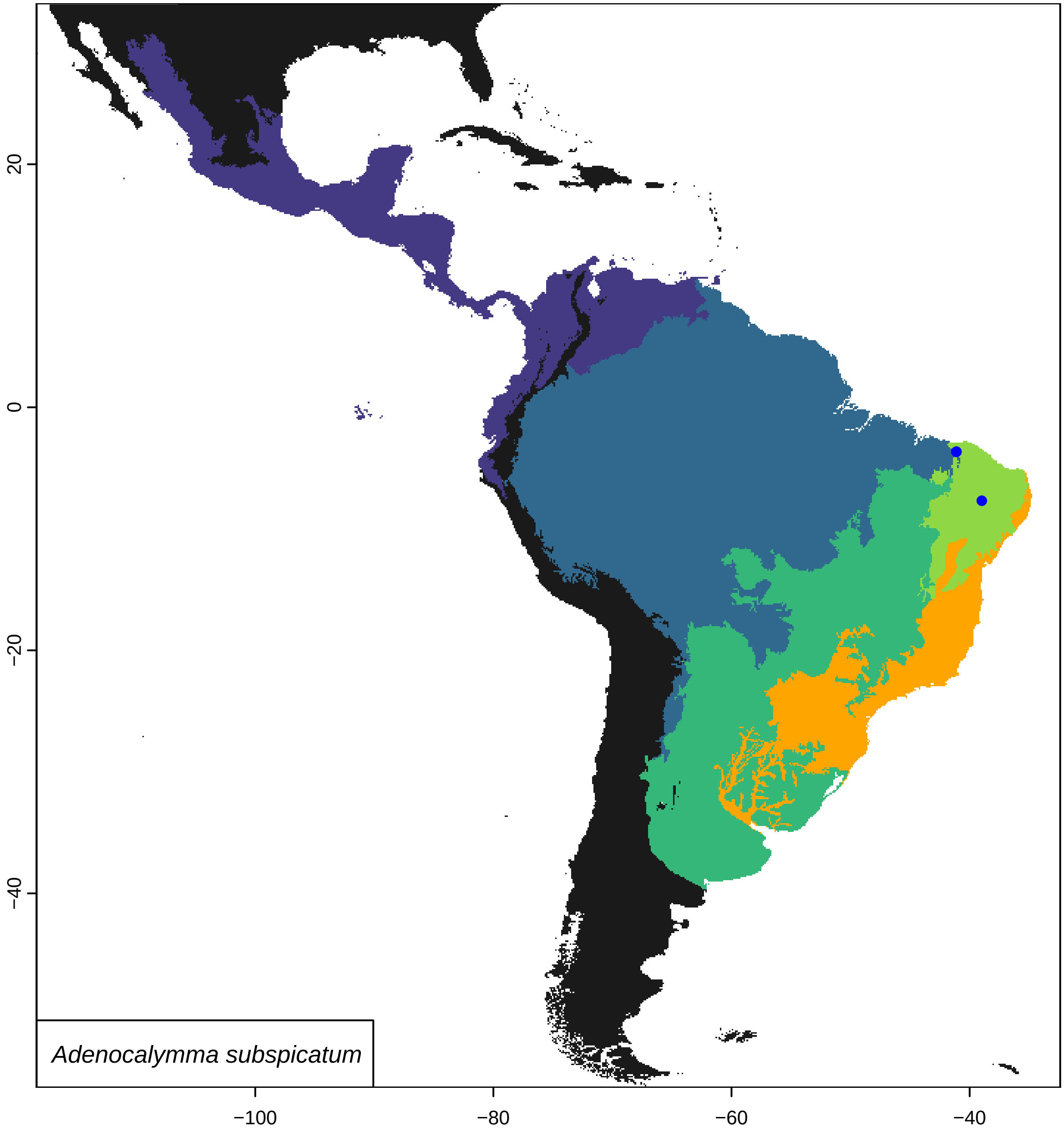

20
0
−20
−40
−100
−80
−60
−40
Adenocalymma subspicatum

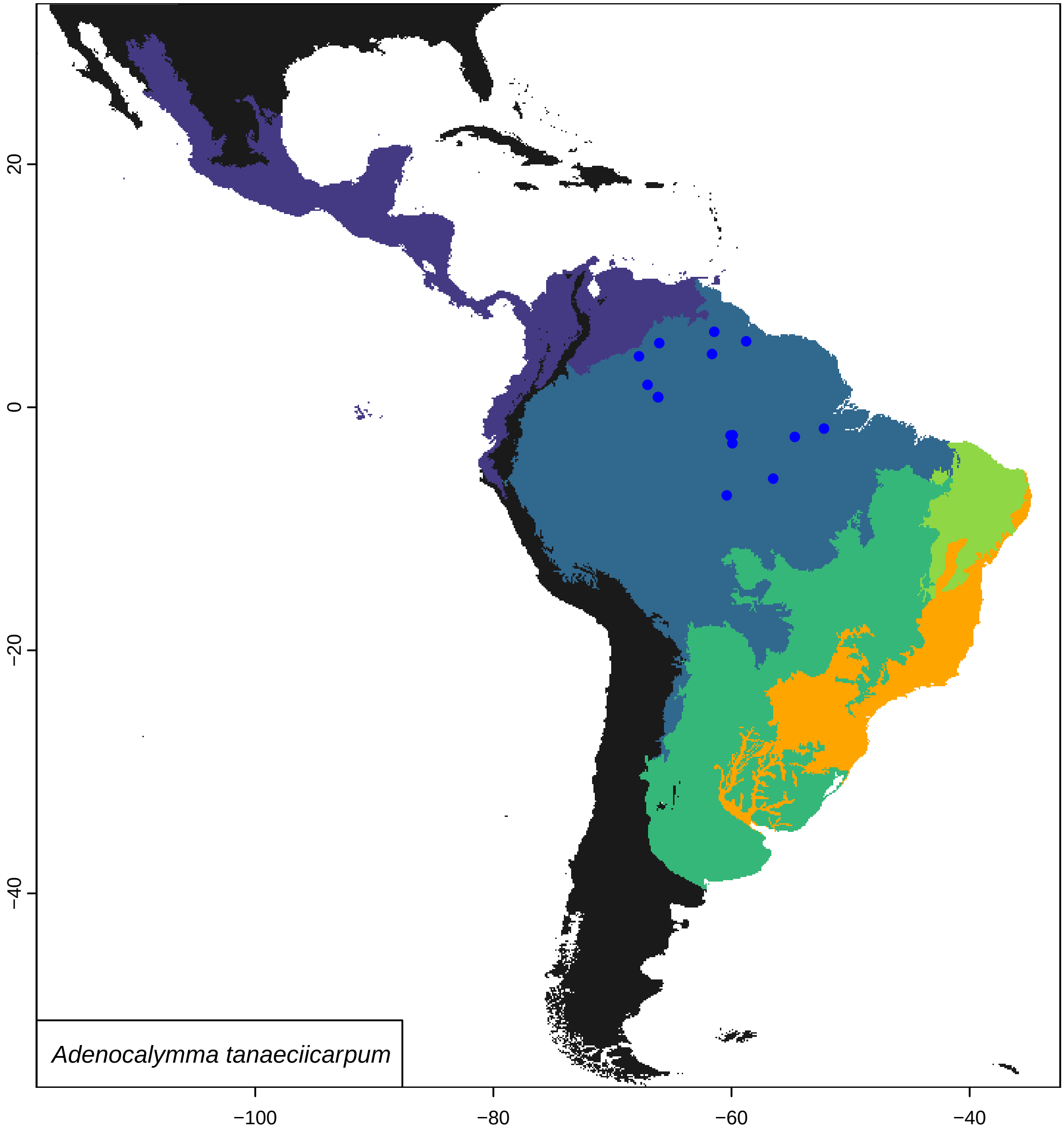

20
0
−20
−40
−100
−80
−60
−40
Adenocalymma tanaeciicarpum

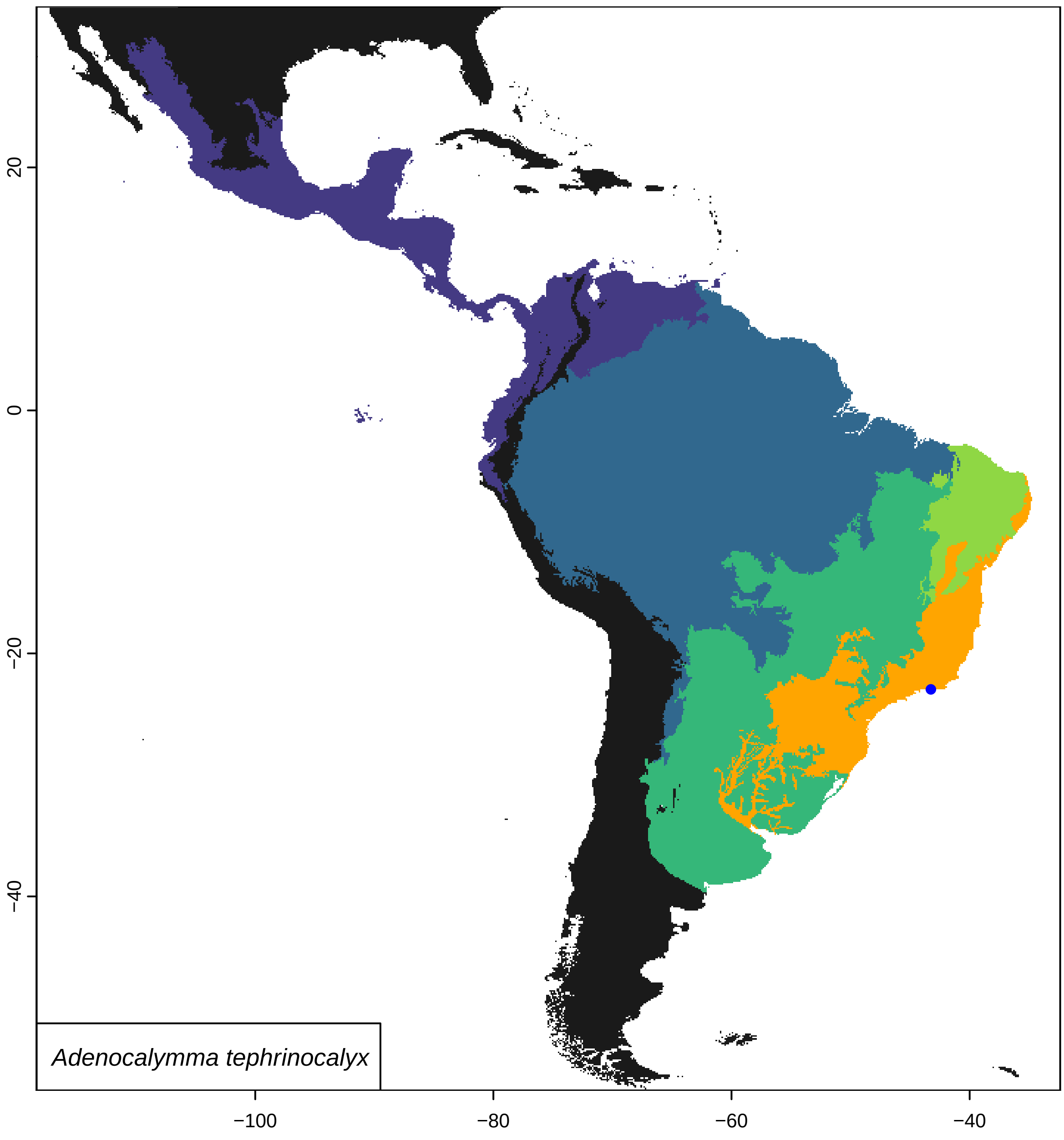
Adenocalymma tephrinocalyx
20
0
−20
−40
−100
−80
−60
−40

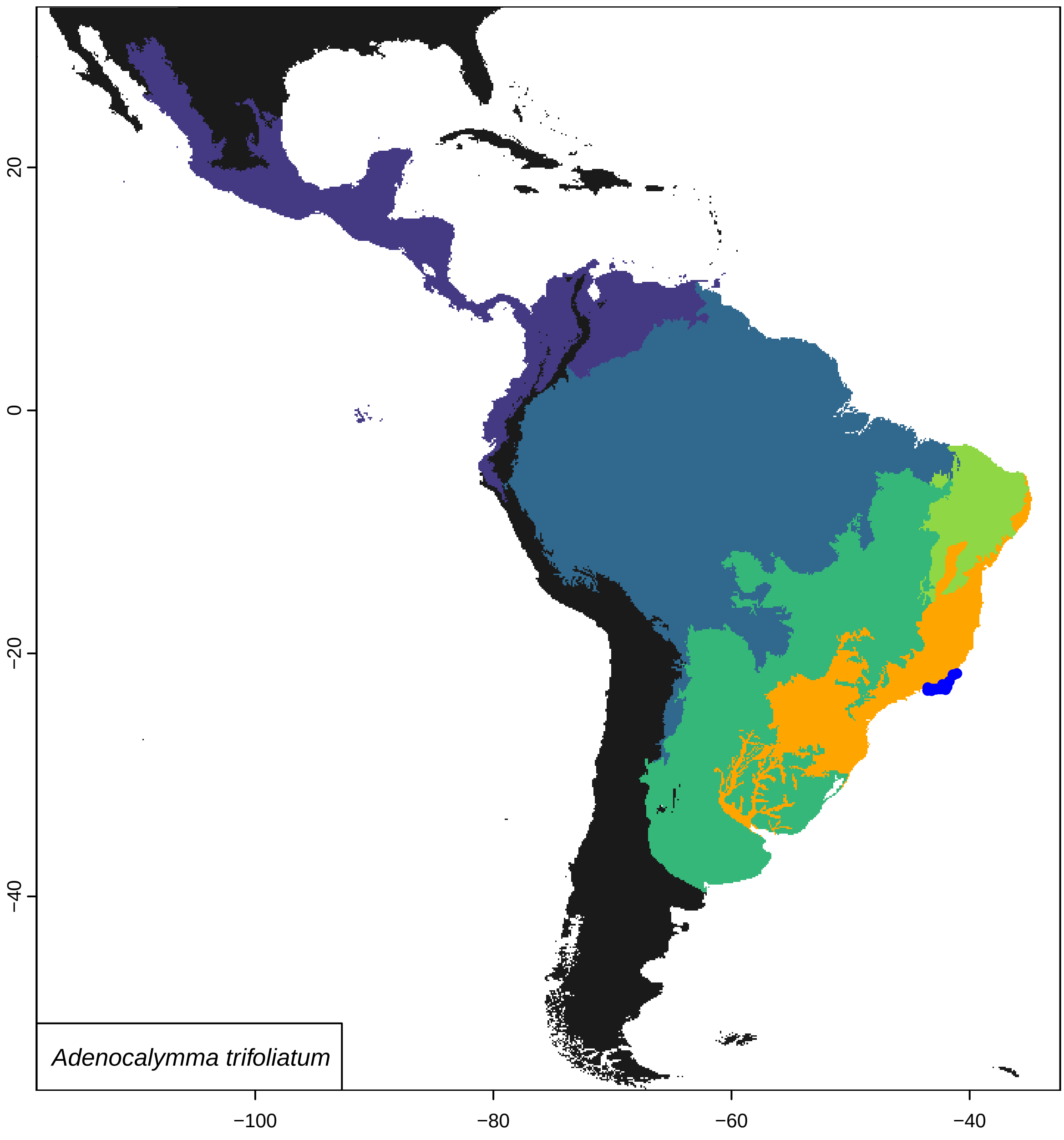

20
0
−20
−40
−100
−80
−60
−40
Adenocalymma trifoliatum

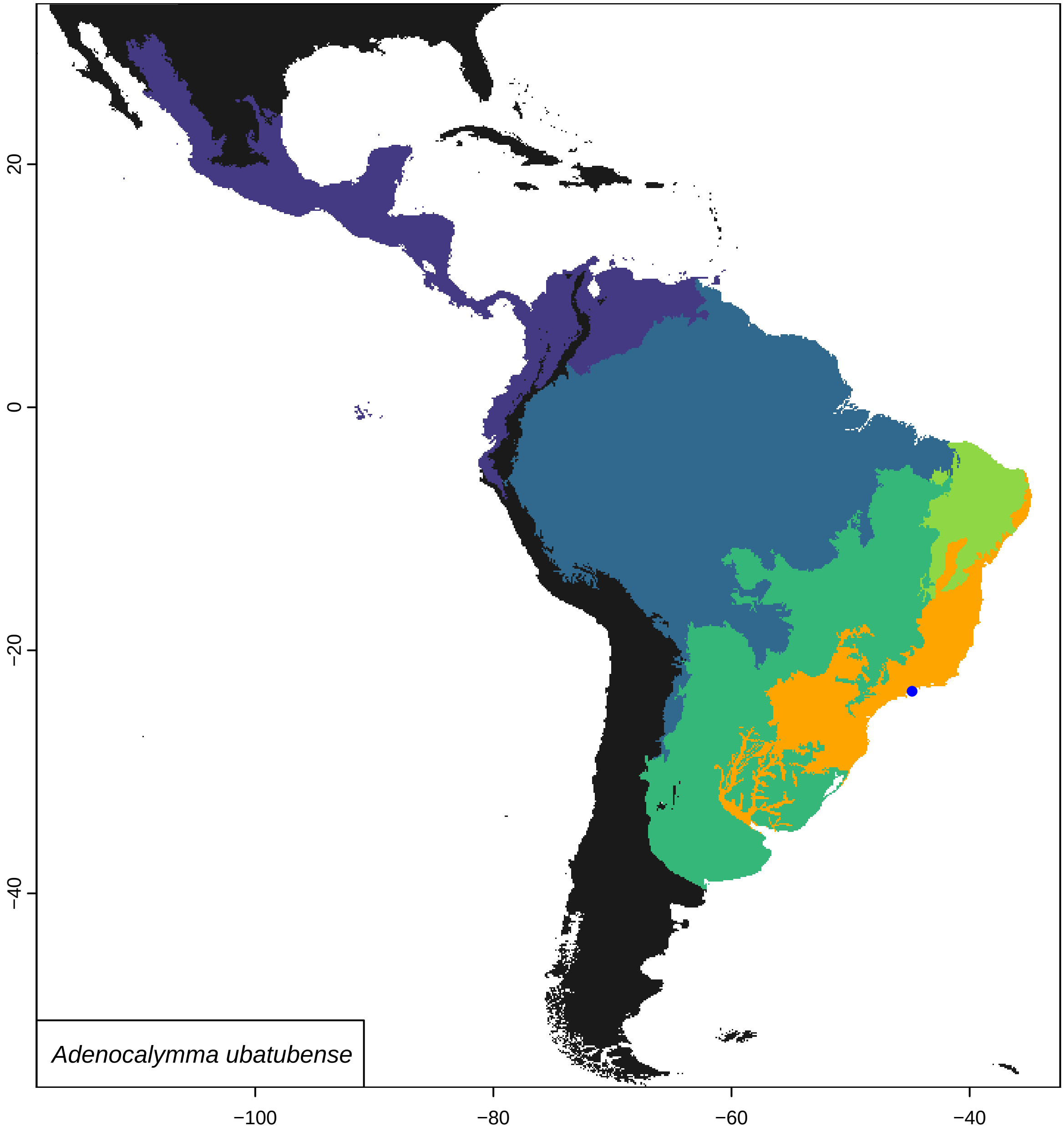

20
0
−20
−40
−100
−80
−60
−40
Adenocalymma ubatubense

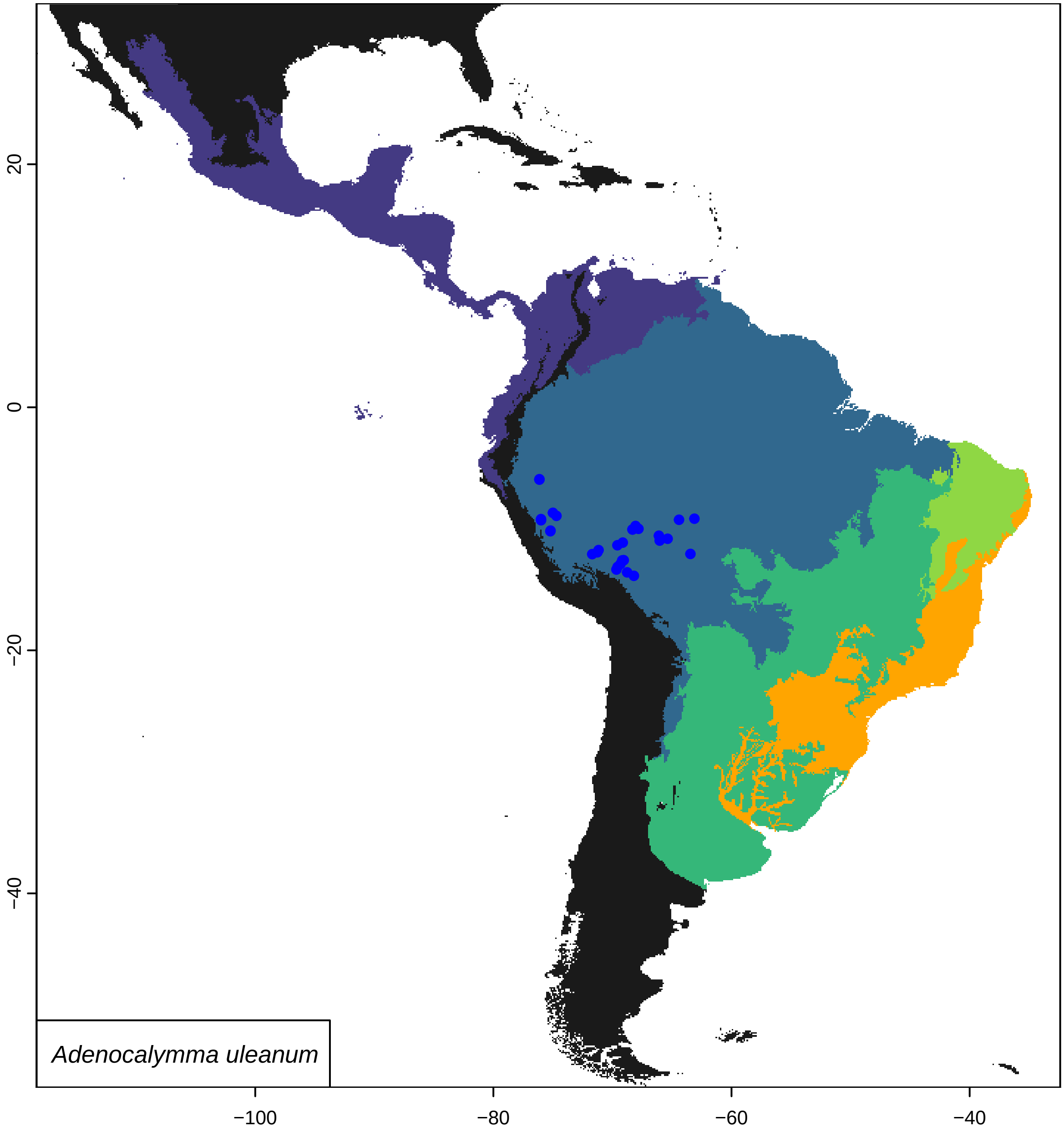
20
0
−20
−40
−100
−80
−60
−40
Adenocalymma uleanum

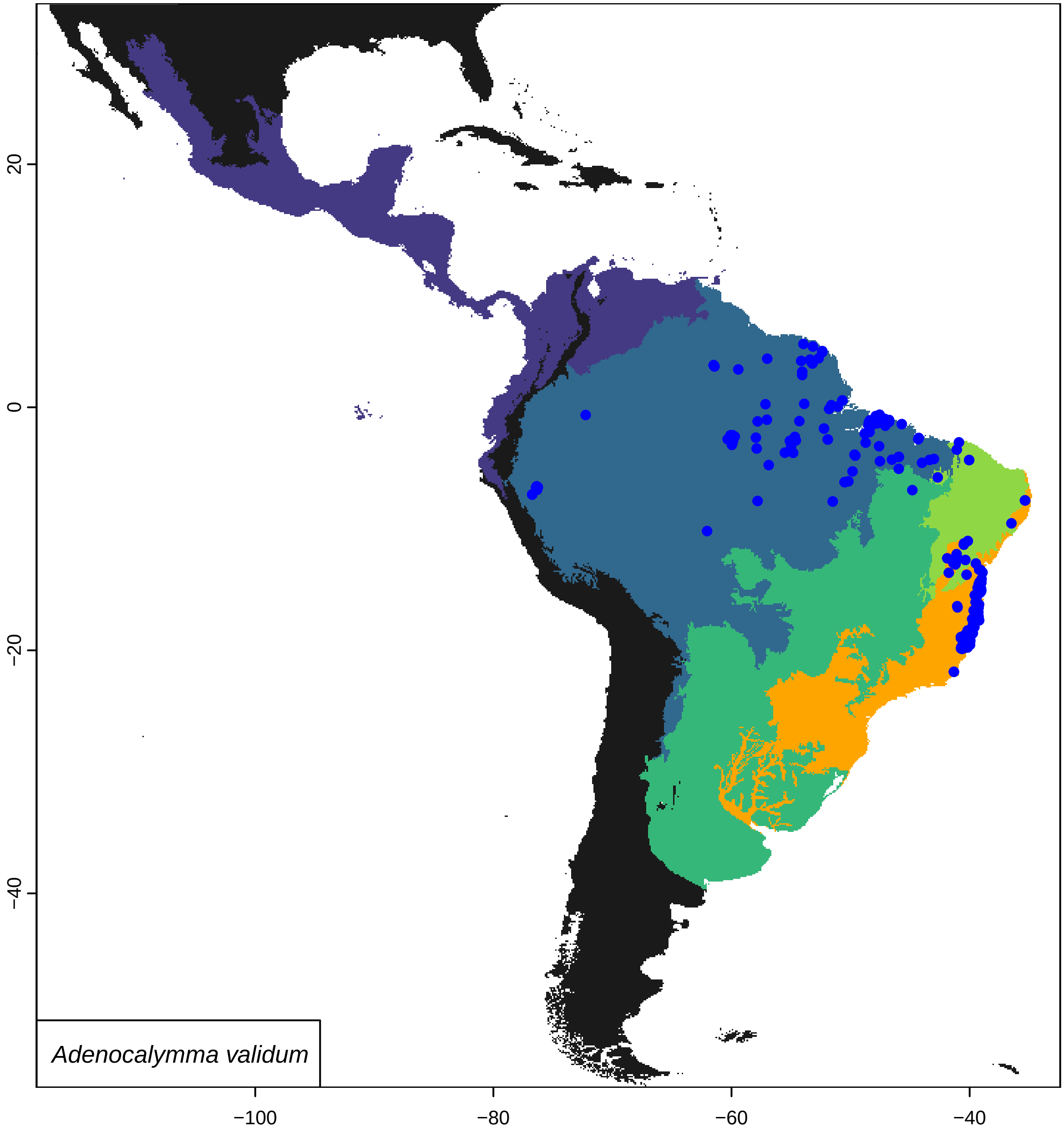

20
0
−20
−40
−100
−80
−60
−40
Adenocalymma validum

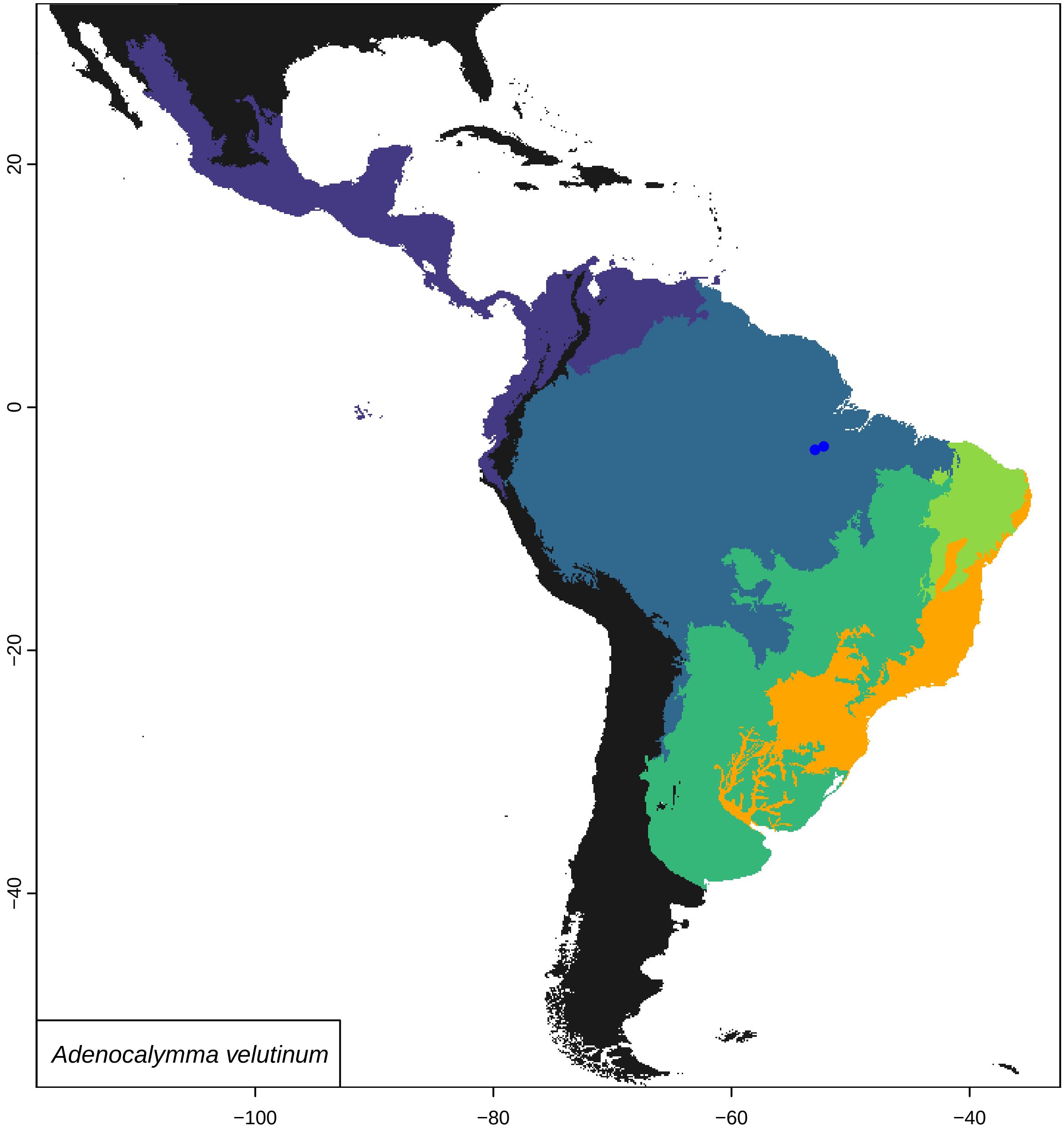

20
0
−20
−40
−100
−80
−60
−40
Adenocalymma velutinum
